# A Historical Review of Brain Drug Delivery

**DOI:** 10.3390/pharmaceutics14061283

**Published:** 2022-06-16

**Authors:** William M. Pardridge

**Affiliations:** Department of Medicine, University of California, Los Angeles (UCLA), Los Angeles, CA 90095, USA; wpardrid@ucla.edu

**Keywords:** blood–brain barrier, endothelium, receptor-mediated transport, carrier-mediated transport, genetic engineering, IgG fusion proteins, nanoparticles, liposomes

## Abstract

The history of brain drug delivery is reviewed beginning with the first demonstration, in 1914, that a drug for syphilis, salvarsan, did not enter the brain, due to the presence of a blood–brain barrier (BBB). Owing to restricted transport across the BBB, FDA-approved drugs for the CNS have been generally limited to lipid-soluble small molecules. Drugs that do not cross the BBB can be re-engineered for transport on endogenous BBB carrier-mediated transport and receptor-mediated transport systems, which were identified during the 1970s–1980s. By the 1990s, a multitude of brain drug delivery technologies emerged, including trans-cranial delivery, CSF delivery, BBB disruption, lipid carriers, prodrugs, stem cells, exosomes, nanoparticles, gene therapy, and biologics. The advantages and limitations of each of these brain drug delivery technologies are critically reviewed.

## Contents

1. Introduction  1.1. Blood–Brain Barrier and Blood–CSF Barrier  1.2. History of the Blood–Brain Barrier  1.3. History of Brain Drug Delivery2. Invasive Drug Delivery to Brain  2.1. CSF Delivery    2.1.1. CSF Microcirculation and Microcirculation    2.1.2. Drug Transfer from CSF to Blood    2.1.3. Lumbar CSF Delivery    2.1.4. Ventricular CSF Delivery  2.2. Intra-Cerebral Delivery    2.2.1. Intra-Cerebral Implants    2.2.2. Convection-Enhanced Diffusion3. Trans-Nasal Drug Delivery to Brain  3.1. Drainage of CSF from Brain to Nose  3.2. Drug Delivery from Nose to Brain  3.3. Clinical Trials of Trans-Nasal Drug Delivery to Brain4. Brain Drug Delivery with Blood–Brain Barrier Disruption (BBBD)  4.1. BBBD Following Intra-Carotid Arterial Infusion    4.1.1. BBBD with Intra-Arterial Hyper-Osmolar Solutions    4.1.2. BBBD with Intra-Arterial Bradykinin Analogs  4.2. BBBD with Intravenous Microbubbles/Focused Ultrasound  4.3. Miscellaneous forms of BBBD    4.3.1. BBBD with Tight Junction Modulators    4.3.2. BBBD with Adenosine Analogs    4.3.3. BBBD with Anti-Bacterial Antibodies    4.3.4. BBBD with Intra-Arterial Polycations    4.3.5. BBBD with Intra-Arterial Amphipathic Agents    4.3.6. BBBD and Free Radicals    4.3.7. BBBD and Electromagnetic Radiation5. Cell-Mediated Transport  5.1. Stem Cells for Brain Drug Delivery  5.2. Exosomes for Brain Drug Delivery6. Brain Drug Delivery of Small Molecules  6.1. Lipid-Mediated Transport of Small Molecules    6.1.1. Approved Small Molecules for the CNS    6.1.2. Mechanism of Small Molecule Diffusion through the BBB    6.1.3. Lipid-Soluble Pro-Drugs    6.1.4. Conjugation of Hydrophilic Drugs to Hydrophobic Carriers  6.2. Carrier-Mediated Transport of Small Molecules    6.2.1. GLUT1 Glucose Carrier    6.2.2. LAT1 Large Neutral Amino Acid Carrier    6.2.3. CAT1 Cationic Amino Acid Carrier    6.2.4. MCT1 Monocarboxylic Acid Carrier    6.2.5. CNT2 Purine Nucleoside Carrier and Adenine Carrier    6.2.6. CTL1 Choline Carrier    6.2.7. Vitamin Carriers    6.2.8. Thyroid Hormone Carriers    6.2.9. Organic Cation Carrier  6.3. Active Efflux Transport of Small Molecules    6.3.1. Brain-to-Blood Efflux    6.3.2. ABC Efflux Transporters    6.3.3. SLC Efflux Transporters7. Absorptive-Mediated Transport of Cationic Proteins or Lectins  7.1. Cationic Proteins    7.1.1. Cationized Proteins    7.1.2. Endogenous Cationic Proteins    7.1.3. Cell-Penetrating Peptides  7.2. Lectins  7.3. Toxicity of Cationic Proteins and Lectins    7.3.1. Toxicity of Cationic Proteins    7.3.2. Toxicity of Lectins8. Receptor-Mediated Transport of Peptides and Monoclonal Antibodies  8.1. Receptor-Mediated Transporters at the Blood–Brain Barrier    8.1.1. Insulin Receptor    8.1.2. Transferrin Receptor    8.1.3. IGF Receptor    8.1.4. Leptin Receptor    8.1.5. LRP1 Receptor    8.1.6. LDL Receptor    8.1.7. Nicotinic Acetylcholine Receptor    8.1.8. Basigin/CD147    8.1.9. Miscellaneous Receptors  8.2. Trojan Horse Delivery Via Blood–Brain Barrier Receptor-Mediated Transport (RMT)    8.2.1. Peptide-Based RMT Trojan Horses    8.2.2. Monoclonal Antibody-Based RMT Trojan Horses  8.3. IgG Fusion Proteins for Blood–Brain Barrier Delivery of Biologics    8.3.1. Lysosomal Enzymes    8.3.2. Neurotrophins    8.3.3. Decoy Receptors    8.3.4. Bispecific Antibodies  8.4. Avidin-Biotin Technology    8.4.1. Peptide Radiopharmaceuticals for Brain Imaging    8.4.2. Antisense Radiopharmaceuticals for Brain Imaging    8.4.3. IgG–Avidin Fusion Proteins9. Nanoparticles  9.1. Nanoparticle Formulations  9.2. Polymer-Based Nanoparticles    9.2.1. Polymeric Nanoparticles    9.2.2. Dendrimers    9.2.3. Micelles    9.2.4. Albumin Nanoparticles  9.3. Lipid-Based Nanoparticles    9.3.1. Liposomes    9.3.2. Solid Lipid Nanoparticles  9.4. Non-Polymeric Nanoparticles    9.4.1. Carbon Nanotubes    9.4.2. Graphene Oxide, Fullerenes, and Quantum Dots    9.4.3. Metallic Nanoparticles  9.5. Mediated Blood–Brain Barrier Delivery of Functionalized Nanoparticles    9.5.1. Carrier-Mediated Transport of Nanoparticles    9.5.2. Absorptive-Mediated Transport of Nanoparticles    9.5.3. Receptor-Mediated Transport of Nanoparticles    9.5.4. Brain Delivery of Nanoparticles with BBB Avoidance Strategies  9.6. Nanoparticle Clinical Trials for the Brain  9.7. Nanoparticle Neurotoxicology10. Gene Therapy of the Brain  10.1. Viral Gene Therapy    10.1.1. Lentivirus-Transfected Stem Cells    10.1.2. Adenovirus    10.1.3. Herpes Simplex Virus    10.1.4. Adeno-Associated Virus  10.2. Non-Viral Gene Therapy of Brain    10.2.1. Cationic Liposomes and Cationic Polyplexes    10.2.2. Pegylated Liposomes    10.2.3. Trojan Horse Liposomes11. Blood–Brain Barrier Transport Methodology  11.1. Physiologic Model of Free Drug in Brain and Plasma  11.2. Free Drug in Plasma and Role of Plasma Protein Binding  11.3. Measurement of Free Drug in Brain    11.3.1. CSF as a Measure of Free Drug in Brain    11.3.2. Free Drug in Brain with Cerebral Microdialysis    11.3.3. Free Drug in Brain In Vitro with Brain Slices or Homogenates  11.4. Measurement of PS^influx^    11.4.1. Brain Uptake index Method    11.4.2. Internal Carotid Artery Perfusion Method    11.4.3. Capillary Depletion Method    11.4.4. Intravenous Injection Methods  11.5. Measurement of PS^efflux^    11.5.1. Brain Uptake index Method    11.5.2. Brain Efflux index Method  11.6. Measurement of Drug Sequestration in Brain In Vivo  11.7. In Vitro BBB Models    11.7.1. Isolated Brain Microvessels    11.7.2. In Vitro Models of BBB Transport in Cell Culture  11.8. BBB Transport Methods from Perspective of Pharmaceutical Industry12. Summary13. PerspectiveAbbreviationsReferences

## 1. Introduction

**Figure 1:**
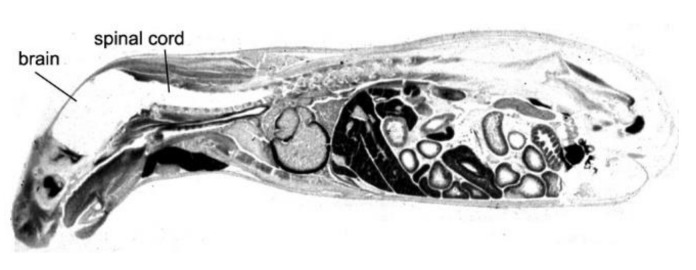
**The blood–brain barrier to small molecules.** Whole body autoradiography of mouse following the IV injection of [^14^C]-histamine shows the lack of transport of this small molecule drug into brain and spinal cord. Reprinted with permission from [2], Copyright© 1986 American College of Physicians.

The driving force in the evolution of brain drug delivery technology is the blood–brain barrier (BBB) and the limitation this barrier creates in the development of new drugs for the brain. More than 98% of small molecule drugs do not cross the BBB [1], as illustrated in Figure 1, which shows the selective organ uptake in the mouse of histamine, a small molecule drug with a molecular weight (MW) of just 111 Daltons (Da).

Following intravenous (IV) administration, histamine penetrates all of the organs of the body except for the brain and spinal cord (Figure 1). The fraction of large molecule biologics that do not cross the BBB is essentially 100%. Therefore, brain drug development, in the absence of brain drug delivery technology, is limited to the <2% of small molecules that penetrate the BBB via lipid-mediated free diffusion [1]. In order to develop new drugs for brain disease from either water-soluble small molecule drugs, or from biologics (recombinant proteins or nucleic acid pharmaceuticals), a multitude of brain drug delivery technologies have emerged over the last 40 years. These technologies can be broadly classified as:

- *Invasive brain drug delivery*: the BBB is circumvented by drug injection into either the cerebrospinal fluid (CSF) following intrathecal or trans-nasal administration, or by trans-cranial direct injection of drug into brain tissue by either intra-cerebral implants or convection-enhanced diffusion (CED).
- *BBB disruption brain drug delivery*: the brain capillary endothelial tight junctions that form the BBB are disrupted by either the intra-arterial infusion of noxious agents, or by the intravenous injection of micro-bubbles followed by sonication of brain.
- *Trans-vascular brain drug delivery*: the non-disrupted brain capillary endothelial barrier is traversed following the re-engineering of the pharmaceutical so as to gain access to multiple carrier-mediated transporters (CMT) for small molecules, or receptor-mediated transporters (RMT) for biologics. This category also includes the development of co-drugs that inhibit active efflux transporters (AET) at the BBB, such as p-glycoprotein (P-gp), as well as the free diffusion of lipid-soluble small molecules.

**Figure 2:**
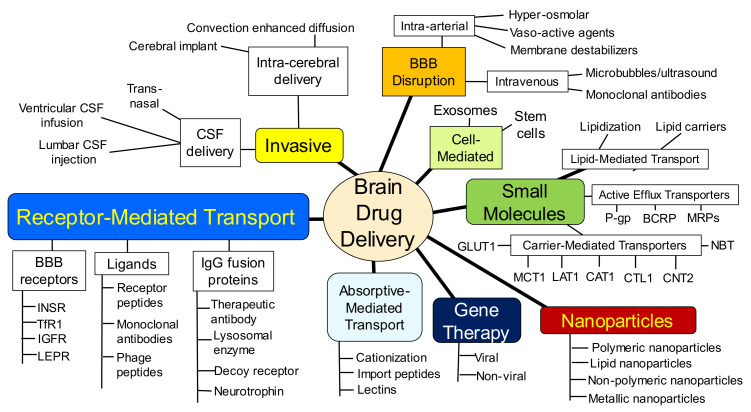
**Overview of brain drug delivery technologies.** These multiple delivery technologies can be broadly classified into 3 categories: (i) invasive brain drug delivery, which includes trans-cranial intra-thecal drug delivery into CSF, intra-cerebral implants, or convection-enhanced diffusion; (ii) BBB disruption brain drug delivery, which includes either the intra-carotid arterial infusion of noxious agents, or the intravenous injection of microbubbles coupled with focal external sonication of the brain; (iii) trans-vascular brain drug delivery, which includes receptor-mediated transport, carrier-mediated transport, active efflux transport, and lipid-mediated transport.

Within each of these 3 spheres, different parallel approaches have emerged to the point where brain drug delivery science has now evolved into a complex maze of competing technologies. This maze is nearly impenetrable by the artisan who practices outside the field of brain drug delivery, or even within a competing brain delivery area. The complexity of modern brain drug delivery science is illustrated by the outline in Figure 2.

Prior to an analysis of each of the brain drug delivery technologies shown in Figure 2, the different anatomic locations of the BBB, at the brain capillary endothelium, and the blood–CSF barrier, at the choroid plexus, are reviewed. The presence of a barrier between blood and brain was discovered in 1900, and the limitation this barrier plays on brain drug delivery can be dated to 1914.

### 1.1. Blood–Brain Barrier and Blood–CSF Barrier

**Figure 3:**
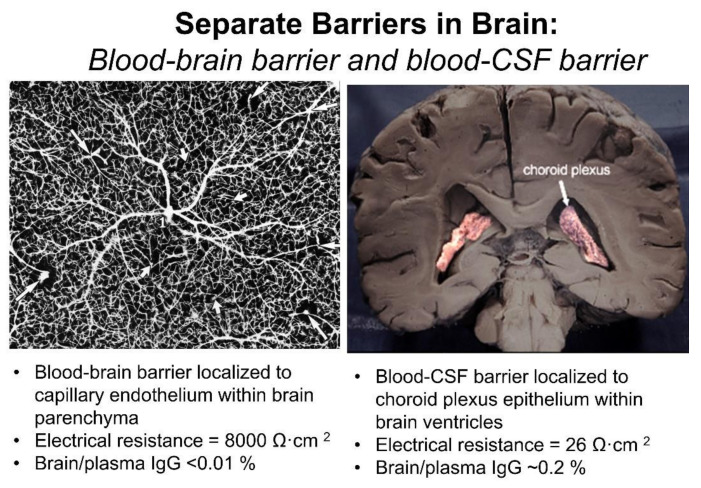
**Blood–brain barrier vs. blood–CSF barrier.** (**Left**) Inverted India ink labeling of microvasculature of human cerebral cortex, which is from [14] with permission, Copyright© 1981 Elsevier. (**Right**) Coronal section of human brain showing the choroid plexus lining the floor of both lateral ventricles. Adapted from [15], Copyright© 2020 licensed under Creative Commons Attribution License (CC-BY).

The BBB and the blood–CSF barrier are functionally and anatomically distinct barriers within the brain. The different anatomical locations of the BBB and the blood–CSF barrier are viewed in Figure 3. The BBB, at the brain microvascular endothelium, is shown in the left panel, and the blood–CSF barrier, at the choroid plexus, is shown in the right panel of Figure 3. The BBB at the brain capillary is formed by endothelial high resistance tight junctions that eliminate any paracellular pathway of solute movement from blood-to-brain extracellular space (ECS) [3]. Minimal pinocytosis within brain capillary endothelium removes any non-specific transcellular pathway of solute transport from blood to brain [4]. The blood–CSF barrier is formed by the epithelial cells of the choroid plexus [5], which lines the floor of each of the 4 cerebral ventricles, including both lateral ventricles shown in Figure 3 (right panel). The blood–CSF barrier is leaky relative to the BBB, as reflected by the electrical resistance across these two barriers. The electrical resistance across the choroid plexus epithelial barrier is only 26 ohm·cm^2^ [6]. In contrast, the electrical resistance across pial vessels on the surface of the brain is 1600 ohm·cm^2^ [7]. However, pial vessels express tight junctional complexes less developed than those in parenchymal vessels, and pial vessels are more permeable than parenchymal capillaries [8,9]. The electrical resistance across the endothelium of capillaries within brain parenchyma is estimated to be 8000 ohm·cm^2^ [10], which is 300-fold higher than the resistance across the blood–CSF barrier [11]. Owing to the relative leakiness of the blood–CSF barrier, serum proteins readily move from plasma to CSF, as reflected by the high CSF/plasma ratio of IgG, which is ~0.2% [12]. In contrast, the brain/plasma IgG ratio in for the parenchyma of brain is <0.01% [13].

The brain capillary endothelium, which forms the permeability barrier between blood and brain parenchyma, is buttressed on the blood side by the endothelial glycocalyx, and on the brain side by the capillary basement membrane and the astrocyte endfeet that terminate on the basement membrane [16]. The thickness of the endothelium is 300 nm from the luminal to the abluminal endothelial membranes [17]. The thickness of the glycocalyx ranges from 100 nm, as measured by electron microscopy [18], to 400 nm, as measured by two-photon microscopy [19]. The glycocalyx covers about 40% of the surface area of the endothelial luminal membrane [20]. The capillary basement membrane covers the entire abluminal endothelial membrane and has a thickness ranging from 20 nm to 200 nm [21]. The basement membrane invests both the abluminal surface of the endothelium and the pericyte, which sits on the abluminal surface of the endothelium. Astrocyte endfeet terminate on the capillary basement membrane [21]. Electron microscopy of cryo-fixed brain shows the astrocyte endfeet cover about 63% of the basement membrane surface [22]. As discussed below, electron microscopy of brain shows that horseradish peroxidase (HRP), a 40 kDa protein, after injection into the brain, moves freely through the brain extracellular space (ECS), through the astrocyte endfeet, and through the capillary basement membrane to reach the abluminal surface of the capillary endothelium [3].

### 1.2. History of the Blood–Brain Barrier

The first known recognition of a restrictive permeability of the blood vessels in brain was reported by Ridley in 1695, as reviewed by Liddelow [23] and Thakur et al. [24]. The restricted uptake of acidic vital dyes by brain as compared to peripheral organs was demonstrated by Ehrlich in the 19th century [23]. Acidic vital dyes were systemically injected in rabbits and all the organs were stained by the dye with the exception of the central nervous system (CNS). However, these observations were attributed to lack of adsorption of the dyes to brain tissue, and not to any barrier between blood and brain. In 1900, Lewandowsky reported experiments on the intravenous and intrathecal injection of sodium ferrocyanide, as reviewed by Liddelow [23] and Macinowski [25]. Lewandowsky observed ferrocyanide effects on the CNS following intrathecal injection but not after intravenous administration, and first used the term, *blut-thirn-schranke*, or blood–brain barrier, to characterize the selective permeability properties of the cerebral capillaries. In 1913, Goldman repeated Ehrlich’s observations that the brain was not stained by acidic dyes following intravenous injection in rabbits, but observed the brain was stained by the dye following intrathecal administration, and Goldman’s findings were summarized in the English literature by Mott in 1913 [26]. At this time, the prevailing view was that nutrients in blood passed first into the CSF and then into brain. Within this view, any barrier between blood and brain must necessarily lie at the choroid plexus, as reflected by Mott’s commentary on Goldman’s experiments:

- “Vital stains possess an affinity for the nervous system, and specially for the ganglion cells. If they are introduced by means of subcutaneous or intravenous injections, they are kept back by the plexus.”
- “From the plexus choroideus the cerebro-spinal fluid receives important metabolic products, which are carried to the nerve substance by the fluid.”

However, in 1916, McIntosh and Fildes [27] reported their findings on the intravenous injection of basic vital dyes, methylene blue and neutral red, which do cross the BBB. They observed the brain stained with no parallel staining of the CSF, and made the following conclusions:

- “Certain dye substances can pass directly from the blood to the brain substance proper without being found in the cerebrospinal fluid, while others fail to penetrate into the brain.”
- Certain substances “do not possess the necessary solubility to allow them to pass from the blood-vessels into the brain substance. Their relative inefficiency has nothing to do with their absence from the cerebrospinal fluid”.

By 1916, McIntosh and Fildes [27] clearly localized the BBB to the brain capillary, not the choroid plexus, and recognized that CSF was not an intermediate compartment between blood and brain.

The ambiguity in regard to location of the BBB, i.e., brain capillary vs. choroid plexus, was reinforced by Stern working in the 1920s, who used the term, *barrier-hemato-encephalique*, or BBB, but concluded the BBB was localized to the choroid plexus [28]. However, by the 1940s, workers such as Broman in 1941 [29], and Friedemann in 1942 [30] observed that the location of the BBB was clearly at the brain capillary wall, and not the choroid plexus. Friedemann [30] wrote, “this paper deals exclusively with the distribution of substances between blood and CNS. As will be shown, distribution between blood and CSF is an entirely different problem and remains outside the scope of this review.” In 1946, Krough [31] observed that Broman had shown the BBB was localized to the brain capillary endothelium.

Consensus on the location of the BBB was elusive, as Hassin [32] wrote in 1948 that “the cerebrospinal fluid represents the tissue fluids of the brain”, and that the “hemato-encephalique barrier (if one must consider such) is the Virchow-Robin spaces”. Hassin, in 1948, reinforced the 1913 view of Mott [26] that CSF was an intermediate compartment as nutrients passed from blood to brain. The reluctance to even accept a specific location of the BBB was presented by Dobbing in 1961 [33], who disputed the concept of a specific BBB, and proposed the use of the term, “brain barrier system”. This concept of ‘brain barrier systems’ is still used today [34], so as to lump together the BBB and blood–CSF barrier as a single system.

**Figure 4:**
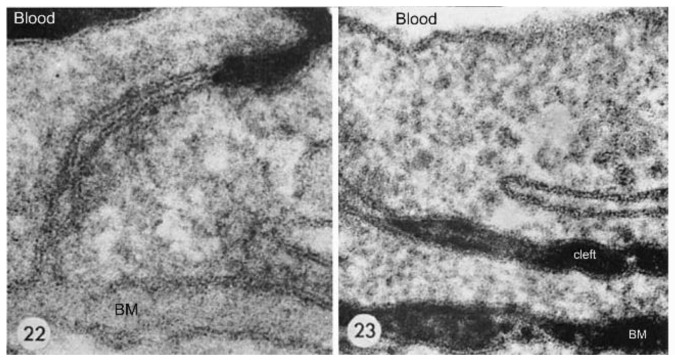
**Brain capillary endothelial tight junctions.** (**Left**) Electron microscopy of mouse brain following the intravenous injection of lanthanum, which is retained in the blood volume of brain at the top of the figure. (**Right**) Electron microscopic histochemistry of mouse brain following the intrathecal injection of HRP. This 40 kDa protein moves freely through the brain ECS, through the astrocyte endfeet, through the capillary basement membrane (BM), and into abluminal clefts formed between adjacent endothelial cells. Reproduced from [3], Copyright© 1969 under Creative Commons Attribution License (Share Alike 4.0 Unported).

The anatomical location of the BBB was unambiguously localized to the brain capillary endothelium by the 1969 work of Brightman and Reese [3]. The brain was examined with electron microscopic histochemistry following the intravenous or intrathecal administration of horseradish peroxidase (HRP), a protein of 40 kDa, or lanthanum, an electron dense trivalent cation [3]. Following intravenous injection, the transport of lanthanum from blood to brain was blocked by the endothelial tight junctions on the luminal side of the brain capillary endothelium, as shown in Figure 4 (left panel).

The endothelial tight junctions eliminated any para-cellular pathway for solute-free diffusion across the endothelium. In addition, no lanthanum was observed within intracellular vesicles, indicating the pinocytotic transcellular pathway found in endothelia of peripheral tissues is nearly eliminated within the brain capillary endothelium [4]. Following the intrathecal administration of HRP, the 40 kDa protein was observed to move freely through the brain ECS, and to traverse the microvascular astrocyte endfeet and capillary basement membrane (Figure 4, right panel). However, further passage of HRP was blocked by the endothelial tight junction at the abluminal side of the capillary (Figure 4, right panel). After decades of controversy, this seminal work finally clarified unequivocally the location of the BBB as residing in the capillary endothelial cells, as suggested by several authors decades before. The anatomic basis of the endothelial barrier was the presence of high resistance tight junctions between endothelial cells. A total of 98% of all listings in PubMed under the search term, ‘blood–brain barrier,’ has been generated since the 1969 publication of Brightman and Reese [3].

### 1.3. History of Brain Drug Delivery

The first indication that the BBB would be a problem in brain drug development occurred in 1914, at the beginning of the synthetic pharmaceutical era. In 1913, Ehrlich described the production of salvarsan and neosalvarsan, which were the first commercial anti-microbial agents, and were marketed by Hoechst for the treatment of syphilis [35]. Salvarsan was a mixture of dimer and trimer complexes of neosalvarsan, which was a polar organic arsenical compound [36]. The first organo-arsenical compound, atoxyl, was synthesized in 1859, and used to treat trypanosomiasis [37]. Ehrlich determined the structure of atoxyl, and he and his colleague, Hata, synthesized salvarsan, and the more soluble, less toxic neosalvarsan, for the treatment of syphilis [37]. However, the syphilitic spirochete invades the brain to cause neurosyphilis, as described by Wile in 1916 [38]. Within a year of Ehrlich’s publication, McIntosh and Fildes [39], in 1914, showed that salvarsan and neosalvarsan do not enter brain from blood in the rabbit following IV administration. They made the following observations:

- “After intravenous injections of salvarsan and neosalvarsan in man and animals no arsenic can be found in the brain.”
- “This phenomenon is not due to a lack of affinity between the brain and the drugs, but to an inability on the part of the drugs to penetrate into the substance of the brain.”

Therefore, in 1914, the problem of the blood–brain barrier and brain drug delivery was born. The most serious effect of syphilis, neurosyphilis, could not be treated by neosalvarsan, owing to the lack of transport of this drug across the BBB.

By the 1950s, drugs such as tricyclic antidepressants and chlorpromazine were developed for affective disorders of the brain [40,41]. These drugs crossed the BBB by free diffusion owing to high lipid solubility and low MW, in the range of 280–320 Da, as discussed in Section 6.1. The role of lipid solubility in BBB transport of small molecules was demonstrated by Oldendorf, in 1972, with the description of the comparative brain uptake of heroin, codeine, and morphine [42]. While lipid-soluble, low-MW drugs crossed the BBB and could be developed for certain brain disorders; drugs that lacked these characteristics were not effective, owing to lack of penetration of the BBB. This was exemplified by methotrexate, which was developed as a treatment for leukemic infiltration of the meninges. Methotrexate was not effective in the CNS following IV administration, so the drug was delivered directly into the CSF compartment by lumbar CSF injection [43].

The first brain drug delivery technology was developed by Ommaya in 1963 [44], which was an implantable reservoir for catheter infusion of drug into the CSF of a lateral ventricle. Ommaya developed the reservoir to facilitate chronic treatment of bacterial meningitis with intrathecal antibiotic [44]. However, the Ommaya reservoir was not widely adopted, owing to the technical issues related to device implantation and maintenance. The next brain drug delivery system that was developed, albeit inadvertently, was the treatment of Parkinson’s disease (PD) with L-DOPA, as reviewed by Hornykiewicz in 1966 [45]. It was known that PD was associated with striatal dopamine deficiency, and that treatment of PD with dopamine, per se, was not effective. However, the dopamine precursor, L-DOPA, which is a large neutral amino acid, was effective in the treatment of PD. L-DOPA acted as a prodrug, as it was converted into dopamine in brain following the enzymatic action of aromatic amino acid decarboxylase (AAAD). The use of L-DOPA was an ‘accidental’ brain drug delivery approach, as the efficacy of L-DOPA was not immediately linked to a BBB transport mechanism [45]. Nearly 10 years later, in 1975, Wade and Katzman [46], using the Brain Uptake Index (BUI) technique of Oldendorf [47,48], demonstrated that brain uptake of L-DOPA was mediated by a BBB neutral amino acid transport system. BBB transport of L-DOPA was saturable, and was inhibited by other large neutral amino acids [46]. The next brain drug delivery technology was introduced in 1979, which aimed to deliver drugs to brain following BBB disruption. The intra-carotid arterial infusion of hyperosmolar 25% (1.4 M) mannitol enhanced brain uptake of methotrexate in dogs [49]. Trans-nasal drug delivery to CSF was introduced as a means to bypass the BBB in 1982. Progesterone was administered to monkeys by intra-nasal or IV administration, and CSF levels of the steroid were reported to be higher following intra-nasal administration [50].

Over the 20-year period of 1980–2000, multiple brain drug delivery approaches were developed. Trans-cranial approaches were developed by 1994, and used intra-cerebral implants, including polymers [51] or genetically engineered fibroblasts [52], or convection-enhanced diffusion [53]. Cationic vectors were developed including cationized albumin [54], and cationic cell-penetrating peptides (CPP), such as tat [55] or penetratin [56]. Lipid carriers, such as docosahexaenoic acid (DHA), were developed [57]. Receptor-mediated transcytosis of receptor ligands through the BBB was proposed in 1986 [58], followed by the development of monoclonal antibodies (MAbs) targeting either the BBB transferrin receptor [59,60] or insulin receptor [61]. The model active efflux transporter (AET) is p-glycoprotein (Pgp), and the high expression of Pgp at the brain capillary was demonstrated in 1989 [62]. Nanotechnology for the brain was introduced with liposomes in 1990 [63], nanoparticles in 1995 [64], and dendrimers in 2004 [65]. BBB disruption with the IV administration of microbubbles coupled with focused ultrasound (FUS) was developed in 2001 [66], and exosomes were introduced for brain drug delivery in 2011 [67].

A literature search in PubMed, using the keyword, ‘brain drug delivery’ yielded a total of 19,087 citations, and over 80% of these citations cover the 20 areas in Table 1.

The PubMed search was refined with the search term, ‘brain drug delivery and keyword’, where 20 different keywords were used, as listed in Table 1. The brain drug delivery technologies are ranked according to the number of citations in PubMed, and range from 124 citations for CED, to 4160 citations for nanoparticles (Table 1). These top 20 keywords account for 81% of the 19,807 citations for brain drug delivery. Nanoparticles, ultrasound, cerebral implants, and nasal delivery account for 42% of all brain drug delivery citations. The remainder of this review will discuss these 20 brain drug delivery technologies listed in Table 1. The relative efficacy and toxicity of each technology will be reviewed, as well as the extent to which, despite decades of experimentation, the technology has failed to lead to FDA approval, or even clinical trials for brain diseases in humans.

## 2. Invasive Drug Delivery to Brain

### 2.1. CSF Delivery

#### 2.1.1. CSF Macrocirculation and Microcirculation

There is 140 mL of CSF in the human CNS, and this fluid is produced at the choroid plexus that lines the four cerebral ventricles (four lateral ventricles, third ventricle, fourth ventricle) [11]. The CSF is absorbed into the peripheral blood, primarily across the arachnoid villi into the superior sagittal sinus of the venous system [68]. This ‘macrocirculation’ of the CSF is relatively rapid and turns over every ~5 h or ~5 times each day in the human brain [11]. The CSF production rate in the 2 g rat brain is 3.4 uL/min [69], and in the 1400 g human brain, it is 350 uL/min [70]. There is also a ‘microcirculation’ within the interstitial fluid (ISF) of brain, as originally described by Cserr et al. [71]. Following the intra-cerebral injection of either 900 Da polyethylene glycol (PEG) or the 68 kDa albumin, both molecules exited the brain at a flow rate of 0.2 uL/min in the rat [71]. Since the rate of clearance was independent of MW, the mechanism of exodus was convection via peri-vascular pathways [71]. Ultimately, the tracers were transferred to blood via intermediate compartments composed of either CSF or the lymphatics. Qualitatively, the ISF microcirculation could provide a conduit for drug entry into brain parenchyma from the CSF. However, quantitatively, the ISF microcirculation is slow compared to the CSF macrocirculation. The rate of fluid flow in brain via the CSF macrocirculation, 3.4 uL/min in the rat [69], is nearly 20-fold higher than the rate of fluid flow via the ISF microcirculation, 0.2 uL/min, in the rat [71]. The comparative kinetics of the CSF microcirculation and the ISF microcirculation comport with the results of many studies that show solutes move from CSF to brain parenchyma slowly by diffusion, and not via more rapid convection pathways, as discussed below.

Transfer of solute between CSF and brain parenchyma via fluid convection is also called the glymphatic pathway. Early work in support of the convection pathway was reported by Wagner et al. in 1974 [72] and Rennels et al. in 1985 [73], and these investigations suggested the ISF microcirculation may proceed at high rates such that this pathway could provide for rapid transfer of solutes in CSF to the deep parenchyma of brain. In these studies, HRP was injected into the lateral ventricle of a rat [72] or cat [73], and brain was removed just 10 min after the intra-cerebroventricular (ICV) injection. Histochemistry showed broad distribution of the HRP deep into brain parenchyma. However, these findings appear to be artifacts following the ICV injection of very large volumes of the HRP solution. In the rat study [72], a volume of 35 μL of 7% HRP was injected into one lateral ventricle as a bolus. This volume is 300% greater than the volume of the lateral ventricle in the rat, which is 12 μL [74]. In the cat study [73], a volume of 1000 μL of 4% HRP was injected. This volume is 800% greater than the volume of the lateral ventricle in the cat, which is 130 μL [75]. In both studies, it was necessary to cannulate either the contralateral ventricle [72], or the cisterna magna [73], to reduce the high pressure introduced by these high-volume injections. As discussed below, multiple studies on the passage of drugs from CSF to brain parenchyma do not support a quantitatively significant role of the convection pathway under conditions of normal pressure in the CSF compartment.

In support of the convection pathway for drug delivery from CSF to brain parenchyma, extensive distribution of a therapeutic MAb into brain parenchyma of the primate was observed following ICV administration of the antibody [13]. However, this study actually supports the classical diffusion pathway. The MAb against the beta secretase-1 was continuously infused 24/7 for 42 consecutive days into the left lateral ventricle of a primate at a rate of 0.4 mL/day. At the end of the 42-day continuous infusion, the brain was removed and the MAb concentration was measured in multiple regions of brain by ELISA. Immunohistochemistry (IHC) showed the MAb was distributed to both sides of the brain, but MAb entry into brain parenchyma was confined to gray matter, with no MAb visible in white matter [13]. The lack of MAb penetration into white matter is inconsistent with a convection pathway, as perivascular flow occurs preferentially in white matter [76]. The MAb concentration in the contralateral motor cortex, which is near the CSF surface, is nearly 30-fold greater than the MAb concentration in the contralateral putamen, a deep parenchyma structure. If convection into brain was the prominent pathway, then the concentration in motor cortex and putamen should be comparable. Quantitative considerations indicate that diffusion, not convection, is the principal mechanism of MAb distribution from CSF to brain parenchyma following ICV infusion for 42 consecutive days. Given a MAb diffusion coefficient in brain of 0.6 × 10^−6^ cm^2^/s [77], and a time for diffusion of 42 days (3.6 × 10^6^ s), the diffusion diameter is 30 mm. The width of the primate brain is 40 mm [11]. Therefore, diffusion alone would be expected to cover 75% of the primate brain following a 42-day constant ICV infusion.

**Figure 5:**
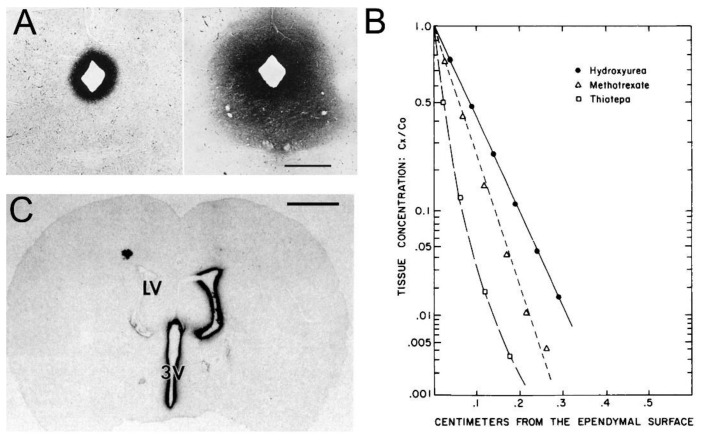
**Limited drug delivery to brain via the ventricular CSF.** (**A**) Peroxidase histochemistry of mouse brain removed at either 10 min (**left**) or 90 min (**right**) after ICV administration of HRP. The magnification bar is 0.7 mm. Reproduced from [3], Copyright© 1969 under Creative Commons Attribution License (Share Alike 4.0 Unported). (**B**) Brain concentrations of hydroxyurea (MW = 76 Da), methotrexate (MW = 454 Da), and thiotepa (MW = 189 Da) at 1–4 mm, removed from ependymal surface at 60 min following drug injection into the lateral ventricle of the Rhesus monkey. Reproduced with permission from [78], Copyright© 1975 Am. Soc. Pharm. Exp. Ther. (**C**) Film autoradiography of a coronal section of rat brain removed 24 h after injection into one lateral ventricle (LV) of [^125^I]-BDNF. The magnification bar is 2 mm; 3V = third ventricle. Reproduced with permission from [79], Copyright© 1994 Elsevier.

Support for the classical diffusion pathway of solute movement from CSF to brain comes from a variety of studies. In 1969, Brightman and Reese [3] injected HRP into the lateral ventricle of the mouse brain, and removed the brain at either 10 min or 90 min for histochemistry. The use of different fixation protocols showed the movement of HRP into brain tissue occurred in vivo, and was not a post-mortem artifact. The distribution of HRP at 10 min and 90 min is shown in Figure 5A.

The HRP diffused 0.2 mm and 0.7 mm into peri-aqueductal brain at 10 min and 90 min, respectively (Figure 5A). Given a brain diffusion coefficient for HRP of 0.6 × 10^−6^ cm^2^/s, it is expected that HRP would diffuse 0.2 mm and 0.7 mm in 10 min and 90 min, respectively [11]. Therefore, the distribution of HRP into brain shown in Figure 5A can be accounted for solely on the basis of diffusion. In 1970, Levin and colleagues showed the concentration of inulin was decreased 10-fold at just 1–2 mm from the CSF surface in rabbits, cats, dogs, and monkeys [80]. In 1975, Blasberg et al. [78] injected small molecules (thiotepa, hydroxyurea, methotrexate) into the lateral ventricle of the Rhesus monkey, and removed the brain 60 min after ICV injection. Drug concentration was measured at 1 mm intervals removed from the CSF surface. Drug concentration in brain decreases logarithmically, and is just 1% of the CSF concentration at 1–2 mm removed from the ependymal surface (Figure 5B). The logarithmic decline in brain drug concentration is consistent with a diffusion model, not a convection model, of drug distribution from CSF to brain. Moreover, diffusion is inefficient as a drug delivery mechanism, and the drug concentration in brain is decreased 99% at just 1–2 mm from the ependymal surface (Figure 5B). In 1994, Yan et al. [79] injected [^125^I]-brain-derived neurotrophic factor (BDNF) into the lateral ventricle of the rat. The brain was removed 24 h later, and coronal sections were analyzed with film autoradiography. The study shows that BDNF distributes only to the ependymal surface of the lateral ventricle ipsilateral to the injection and to the third ventricle. BDNF diffusion into brain parenchyma from the CSF compartment is limited to a distance of only 0.2 mm. The failure to observe BDNF in the contralateral brain is due to the unidirectional flow of CSF from the lateral ventricle to the third ventricle to the fourth ventricle and into the spinal canal and over the convexities of the cerebrum. The only path for distribution to the contralateral ventricle following a ICV injection in one lateral ventricle is reflux through the foramen of Monro from the third ventricle to the contralateral lateral ventricle. There may be minimal reflux during diastole [81], but this does not result in significant drug distribution to the contralateral ventricle as shown in Figure 5C. The distribution in brain of [^125^I]-insulin-like growth factor (IGF)-1 following an ICV injection of the peptide was also determined by film autoradiography, and a result identical to that shown in Figure 5C was reported [82]. In summary, the data in Figure 5 show that drug movement from CSF to brain parenchyma is limited by diffusion, which is fundamentally incompatible with a quantitatively significant role for the convection or glymphatic pathway.

#### 2.1.2. Drug Transfer from CSF to Blood

The paradox of intrathecal drug delivery to brain, i.e., drug injection into the CSF compartment, is that this route delivers drug to blood, not to brain parenchyma. The studies described in Figure 5 show that the ICV route only delivers drug to the ependymal surface of brain lining the CSF flow tracts. In parallel with the slow entry of drug into brain, there is a rapid movement of drug from CSF to blood following ICV drug administration. This fast CSF-to-blood transfer occurs as the entire CSF volume is absorbed into the venous blood ~5 times per day. In 1965, Fishman and Christy [83] studied the distribution of corticosteroids in blood following an intrathecal injection, and they concluded, “the intraspinal route of administration of free steroid is, in effect, equivalent to no more than a prolonged intravenous injection”. Additionally, in the 1960s, Reed and Woodbury [84] showed the plasma profile of iodide in rats was identical within 5 min of administration either as an IV injection or as an intrathecal injection in the cisterna magna. In 1984, Aird [85] showed that the dose of barbiturate that induced anesthesia in dogs was identical whether the drug was administered by injection into the blood or the CSF of the cisterna magna. After CSF injection, the drug rapidly moved to the blood, and then entered brain following transport across the BBB. Aird [85] concluded, “the relative effectiveness of intrathecal agents should be evaluated by comparing maintenance doses for a given central effect, when produced by both intrathecal and IV route”. That is, a clinical trial testing the CNS efficacy of a drug following intrathecal injection should include a control group wherein the drug was administered by IV injection. This point is illustrated for an Ommaya reservoir clinical trial discussed in Section 2.1.4. Other examples of the rapid movement of drug from CSF to blood include:

- The intrathecal injection of an interferon resulted in drug distribution to the surface of the brain, and to the blood, but not into brain parenchyma [86].
- The effect of intrathecal cholecystokinin (CCK) on food intake was found to be caused by CCK action in peripheral organs following CCK transfer from CSF to blood [87].
- Drug was injected into CSF in rats implanted with an intra-cerebral dialysis fiber; however, the drug did not appear in the dialysate of brain following ICV administration [88].
- Liver glycosaminoglycans (GAG) were reduced in the Type IIIB Mucopolysaccharidosis (MPSIIIB) mouse following the intrathecal injection of N-acetyl-α-glucosaminidase (NAGLU), the enzyme that is mutated in MPSIIIB [89], owing to enzyme movement from CSF to liver via the blood.
- The rapid movement of a monoclonal antibody (MAb) from CSF to liver, via the blood, was demonstrated by positron emission tomography (PET) in humans following the administration of the [^124^I]-8H9 MAb via an Ommaya reservoir. Whole body PET scans at 24 h after intrathecal injection showed the antibody was present in liver, but not within the parenchyma of brain [90].

#### 2.1.3. Lumbar CSF Drug Delivery

Some drugs are FDA approved for CNS conditions following drug injection into the lumbar CSF. As noted by Aird [85], intrathecal drug delivery can be effective for conditions that affect the *surface* of the brain or spinal cord, which is contiguous with the CSF flow tract. Intrathecal morphine is effective for pain [91], because opioid receptors are expressed on the surface of the spinal cord [92]. Intrathecal baclofen is used to treat spinal spasticity [93], as gamma aminobutyric acid (GABA)-B receptors are expressed on the surface of the spinal cord [94].

Lumbar injection of nusinersen is FDA approved for treatment of spinal muscular atrophy (SMA) [95]. Nusinersen is a 2′-O-methoxyethyl phosphorothioate antisense oligodeoxynucleotide (ASO), which modulates alternative splicing of the survival motor neuron (SMN)-2 gene [96]. SMA is a disease of spinal cord motor neurons, and these neurons lie near the surface of the spinal cord [97]. Nusinersen is not representative of drug distribution in the spinal cord following intrathecal administration. Nusinersen has a very long residence time in CSF with a T_1/2_ of 191 days in the mouse [96]. The molecular basis for this long residence time in CSF is not clear but appears to be related to the sulfur moiety of the phosphorothioate ASO. A phosphorodiamidate ASO, which is a sulfur-free ASO, is less effective in vivo, although both the phosphorothioate ASO and the phosphorodiamidate ASO are equally effective in cell culture [96]. Based on the FDA approval of intrathecal nusinersen for a disease of the surface of the spinal cord, other ASOs entered CNS clinical trials for treatment of the parenchyma of brain or spinal cord by drug injection into the lumbar CSF. Tominersen is an ASO targeting the huntingtin mRNA of Huntington’s disease (HD), and tofersen is an ASO targeting the superoxide dismutase 1 (SOD1) mRNA of SOD1 dependent amyotrophic lateral sclerosis (ALS) [98]. Since these ASOs do not cross the BBB, and since no antisense BBB delivery technology was developed by the drug sponsors, both tominersen and tofersen were delivered to brain by intrathecal injection into the lumbar CSF [98]. The phase 3 trials of both tominersen and tofersen ended in clinical failures, which is attributed to the poor penetration of drug into brain parenchyma following drug injection into CSF. The nusinersen model for treatment of the surface of the spinal cord by lumbar CSF injection could not be replicated for treatment of the parenchyma of brain by lumbar CSF injection.

In an effort to treat the brain in genetic lysosomal storage disease, the recombinant lysosomal enzyme was delivered to brain by intrathecal injection into the lumbar CSF. Injection of recombinant iduronate 2-sulfatase (IDS), the enzyme that is mutated in MPSII (Hunter syndrome), into the lumbar CSF resulted in a reduction in CSF GAGs, but had no improvement on cognitive function [99]. Chronic injection of N-sulfoglucosamine sulfohydrolase (SGSH), the enzyme mutated in MPSIIIA (Sanfilippo A syndrome), caused a reduction in CSF heparan sulfate [100], but had no effect on cognitive function in this disease, and the clinical trial was terminated [101].

Drug development for a brain disease, which is not restricted to the surface of the brain or spinal cord, by intrathecal drug delivery to brain is a futile effort, because drug is only distributed to the surface of the brain following drug injection into CSF (Figure 5). The futility arises not from the process of CNS drug discovery, but rather from the use of an ineffective brain drug delivery technology.

#### 2.1.4. Ventricular CSF Drug Delivery

The Ommaya reservoir was developed in 1963 [44] as an alternative to repeat intrathecal injections. A reservoir is implanted in the subcutaneous tissue of the skull and a catheter connects the reservoir to the CSF compartment of one lateral ventricle. An Ommaya reservoir delivery approach can be expected to treat diseases of the surface of the brain, which are contiguous with the CSF flow tract, such as meningitis, or meningeal infiltration in acute leukemia, and the first application of the Ommaya reservoir was the treatment of cryptococcal meningitis [44]. In 1975, Shapiro et al. [102] compared the CSF concentration of the chemotherapeutic agent, methotrexate, in CSF following IV administration, injection in the lumbar CSF, or injection in the ventricular CSF using an Ommaya reservoir. Administration of methotrexate via an Ommaya reservoir connected to the lateral ventricle provided for a more consistent delivery of methotrexate to the ventricular CSF than was afforded by drug injection into the lumbar CSF [102]. Previously, in 1962, Rieselbach et al. [103] showed in primates that the lumbar injections of large volumes, e.g., 10% of the CSF volume, were necessary in order to achieve consistent drug distribution into the subarachnoid space around both cerebral hemispheres. The injection of chemotherapeutic agents into the ventricular CSF with an Ommaya reservoir is still current practice, particularly for childhood brain tumors [104].

The Ommaya reservoir was originally designed to treat acute diseases of the surface of the brain following injection of the antibiotic or chemotherapeutic agent into the ventricular CSF. However, given the legacy misconception that CSF is equivalent to the ECS of brain, as discussed in Section 1, it was natural to broaden the application of the Ommaya reservoir to the treatment of chronic disease of the brain parenchyma. Setting aside the invasive nature, and clinical complications of this delivery system [105], the physiology of drug transfer from CSF to brain would argue against the viability of treating intra-parenchymal brain disease by chronic ICV drug administration. First, investigations over many decades show that drug in CSF distributes only to the CSF surface of the brain as illustrated in Figure 5, and discussed in Section 2.1.1. Second, drug injected into the CSF rapidly moves to the peripheral blood, where the drug can exert pharmacologic actions in peripheral organ, which could be falsely attributed to a CNS site of action, as discussed in Section 2.1.2. As originally emphasized by Aird [85] in 1984, any examination of the pharmacologic effect of intrathecal drug administration should include a side-by-side evaluation of drug effects following IV infusion. Predictably, with one exception discussed below, ICV drug administration has not achieved FDA approval for the treatment of brain parenchyma of chronic disease. Patients with acquired immune deficiency syndrome (AIDS) and multi-focal leukoencephalopathy were treated with cytarabine, a highly polar small molecule, by weekly injections into the ventricular CSF with an Ommaya reservoir, but without a clinical benefit [106]. Glial-derived neurotrophic factor (GDNF) was administered to PD patients by the ICV route, but without a clinical effect on the disease [107]. A potential toxicity may arise from the ICV administration of neurotrophic factors. This mode of brain drug delivery produces a very high drug concentration at the ependymal surface of brain, as shown in Figure 5C. The repeat ICV injection of basic fibroblast growth factor (bFGF) causes a reactive astrogliosis along the ependymal surface [108]. The chronic ICV infusion of nerve growth factor (NGF) stimulates axonal sprouting and Schwann cell hyperplasia within the pial-arachnoid surface of brain [109].

In 2017, the FDA approved the first, and only, treatment of parenchymal brain disease where the drug is administered with a chronically implanted Ommaya reservoir in a lateral ventricle. Recombinant tripeptidyl tripeptidase 1 (TPP1, cerliponase alfa) was approved for the treatment of Ceroid Lipofuscinosis 2 (CLN2) disease following ICV enzyme infusion in a lateral ventricle [110]. CLN2 disease is a lysosomal storage disorder caused by mutations in the TPP1 gene, and is characterized by childhood neurodegeneration, language delay, motor abnormalities, seizures, blindness, and early death [111]. The cDNA encoding for human TPP1 was cloned and expressed in CHO cells in 2001 [112]. However, intravenous Enzyme Replacement Therapy (ERT) with recombinant TPP1 was not initiated for CLN2 disease, because TPP1 does not cross the BBB [113]. So as to develop a treatment of the brain in CLN2 disease, the TPP1 proenzyme was infused in children with CLN2 disease into a lateral ventricle with a chronically implanted Ommaya reservoir every 2 weeks at a dose of 300 mg of enzyme in a volume of 10 mL over a 4 h period [110]. This infusion volume exceeds the entire volume of the lateral ventricle, which is 8.5 mL in adult humans, as discussed in Section 10.1.4. The control group in this pivotal clinical trial was not intravenous ERT, but rather historical controls [110]. The trial should have been designed with an intravenous ERT treatment group, because the TPP1 enzyme in CSF rapidly is exported to blood [114]. TPP1, similar to other lysosomal enzymes, is mannose 6-phosphorylated (M6P), and is a ligand for the M6P receptor (M6PR) [112], which is widely expressed in peripheral tissues [115]. Owing to the high expression of the M6PR in peripheral tissues, recombinant TPP1 is rapidly taken up by peripheral tissues, and is cleared from plasma with a T_1/2_ of just 12 min [113]. Lipofuscin granules, the lysosomal inclusion bodies that accumulate in CLN disease, are formed in peripheral organs including skeletal muscle [116]. Therefore, following the ICV injection, the TPP1 enzyme moves from CSF to plasma followed by uptake into peripheral organs via the M6PR. This process could contribute to the improved motor function of children with CLN2 disease as compared to historical controls that expressed no TPP1 enzyme [110]. Such speculation would have been obviated by a clinical trial design that compared ICV drug delivery with intravenous drug delivery, as opposed to historical controls [110]. The admonitions of Fishman and Christy in 1965 [83], and of Aird in 1984 [85], that an intrathecal drug injection is similar to an IV infusion, were not heeded in the trial design of TPP1 in CLN2 disease [110]. To date, recombinant TPP1 for CLN2 is the only treatment that is FDA approved for any chronic CNS disease of brain parenchyma wherein the drug is infused in a chronically implanted Ommaya reservoir in a lateral ventricle [117].

### 2.2. Intra-Cerebral Delivery

#### 2.2.1. Intra-Cerebral Implants

An alternative to intrathecal drug delivery to brain is a trans-cranial intra-cerebral injection of drug encapsulated in a polymer or released from a genetically engineered cell line. However, similar to intrathecal drug delivery, the limiting factor in intra-cerebral delivery is diffusion. The brain concentration of drug that enters the parenchyma via diffusion decreases logarithmically with each mm of diffusion distance [118], as illustrated in Figure 5. The maximal effective diffusion distance for small or large molecules in brain is 0.2–2 mm, and this is irrespective of the mechanism of delivery including intra-cerebral implants, ICV administration, intra-cerebral micro-dialysis or intra-cerebral micro-infusion [118].

There is an FDA-approved treatment for brain cancer, carmustine or Gliadel^®®^, which is an intra-cerebral implant form of brain drug delivery. Carmustine is a dime-sized wafer of a water-soluble polymer embedded with a small molecule chemotherapeutic alkylating agent, 1,3-bis(2-chloroethyl)-1-nitroso urea (BCNU) [51,119]. The polymer is 20% 1,3-bis(p-carboxyphenoxy) propane and 80% sebacic acid, which is a C-8 dicarboxylic acid found in castor oil. The carmustine polymeric/BCNU wafer is placed in the brain cavity created by the neurosurgical extirpation of the bulk of the cancer, and was first tested in recurrent malignant glioma [120], followed by trials that placed the wafer in the brain cavity at the first surgical resection for malignant glioma [121,122]. Statistical analysis showed the carmustine wafer increases survival in malignant glioma by 10 weeks from 11.6 months to 13.9 months [123]. Subsequent to the 1996 FDA approval of carmustine, no similar intra-cerebral implants for brain cancer, or any other brain disease, have reached regulatory approval. This intra-cerebral implant approach to brain drug delivery cannot escape the physical limitations of diffusion, and the fact that brain 2 mm or more away from the implant is exposed to very little drug released from the wafer [118,119].

The intra-cerebral implant method of brain drug delivery has also been tested following the intra-cerebral injection of genetically modified cells. Rat fibroblasts permanently transfected with a lentivirus encoding for prepro brain-derived neurotrophic factor (BDNF) were implanted in the substantia nigra of rats 1 week prior to the intra-striatal injection of the neurotoxin, 1-methyl-4-phenylpyridinium (MPP+) [52]. MPP+ injection creates an experimental model of PD, which involves neurodegeneration of the nigral-striatal tract in brain. The intra-cerebral implant of the BDNF secreting cells doubled the number of surviving neurons in the nigral-striatal tract [52]. The C2C12 mouse myoblast line was permanently transfected with a gene encoding for human GDNF and encapsulated in a 5 mm rod composed of poly(ethyl-sulfone), followed by implantation in the striatum of the rat, and the diffusion of GDNF from the rod was followed by immunohistochemistry [124]. GDNF was detected within 2 mm of the rod [124]. This distance, 2 mm, may be significant for the 2 g rat brain, but would not cover much volume in the 1400 g human brain. Neural stem cells have been permanently transfected with a variety of neurotrophic factors, and intra-cerebral cell-mediated drug delivery to brain has been reviewed [125].

In an attempt to counter the limited drug distribution in brain following diffusion from a single intra-cerebral injection depot, multi-pronged catheter bundles were described in 1988 [126]. A bundle of four catheters was developed, and 17–25 such bundles were implanted in the brain of patients with malignant glioma for infusion over 10–14 days of the alkylating agent, cisplatin. With this approach, a total of 68–100 sites of infusion in the brain was created [126]. There has been renewed interest in the multiple catheter approach to intra-cerebral brain drug delivery, as reviewed below for convection-enhanced diffusion.

#### 2.2.2. Convection-Enhanced Diffusion

Convection-enhanced diffusion (CED) was developed to overcome the limitations imposed by diffusion on intra-cerebral drug delivery [53]. A catheter is implanted in the brain and fluid flow through the catheter is driven by an external pump, which is implanted in the abdomen. The intent was for drug to move through the brain ECS by convection, rather than diffusion. In the initial evaluation, [^111^In]-transferrin (Tf) was infused bilaterally by CED in the corona radiata in the cat [53]. The catheter was placed in the white matter of the corona radiata as it was believed that bulk flow in brain would take place preferentially in parallel to the myelin tracts of white matter. Film autoradiogaphy showed a mean radial spread of the Tf though brain of 3 mm [53], which would be equivalent to a volume of ~100 mm^3^. In contrast, the volume of the putamen of the human brain is 6000 mm^3^ on each side of the brain [127]. These early findings with CED foretold a potential problem in adequate drug distribution to brain in human clinical trials using CED to deliver neurotrophins to the striatum, as discussed below.

CED was used for brain delivery of cationic liposomes encapsulating herpes simplex virus (HSV)-1 encoding thymidine kinase in patients with recurrent glioblastoma multiforme (GBM) [128]. Patients were administered IV ganciclovir for 2 weeks starting 4 days after the HSV1 administration by CED. This small open label trial of eight patients did not advance to a phase 3 trial [129]. A phase 3 double-blind, placebo-controlled randomized clinical trial (RCT) was performed with the bilateral administration of recombinant GDNF to the putamen of patients with PD [130]. A total of 34 patients were randomized to CED groups that received either GDNF or placebo. The pump was placed in the abdomen and a subcutaneous catheter terminated in the posterior dorsal putamen on both sides of the brain [130]. The trial was unblinded after determining the Unified Parkinson Disease Rating Scale (UPDRS) in all patients after 6 months of CED. There was no significant difference between the UPDRS scores of the GDNF or placebo treated subjects with CED brain drug delivery [130].

**Figure 6:**
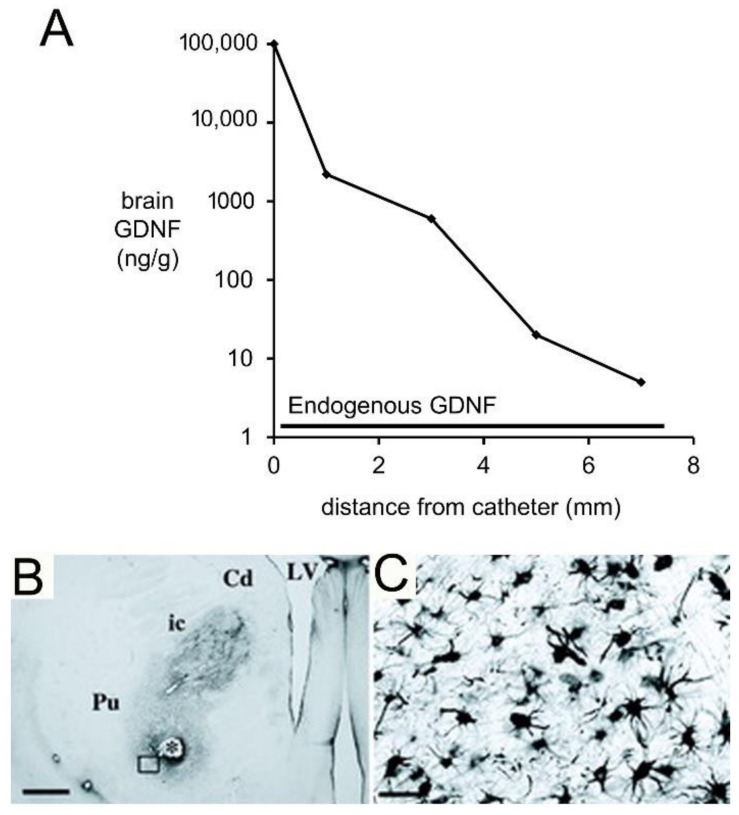
**Brain drug delivery by convection-enhanced diffusion.** (**A**) Concentration of GDNF in primate brain at 1–7 mm removed from the CED catheter. Derived from data reported by Salvatore et al. [131] and reproduced with permission from [132], Copyright© 2010 Taylor and Francis. Original GDNF concentrations, in pg per mg protein, were converted to ng per gram brain, based on 100 mg protein per gram brain [133]. (**B**,**C**) Glial fibrillary acidic protein (GFAP) immunohistochemistry of monkey brain following CED administration of GDNF. Magnification bar is 1 mm in (**B**) and 50 microns in (**C**). Reproduced with permission [134], Copyright© 2003 John Wiley & Sons.

A Rhesus monkey study using CED delivery of GDNF to brain illustrated the limitations of the CED approach for brain drug delivery [131]. GDNF (14 μg/day) was infused into the right putamen of adult Rhesus monkeys at a rate of 144 μL/day for 7 consecutive days. The CED catheter was connected to a pump implanted subcutaneously in the abdomen [131]. After 7 days of CED, the brain was removed, and the distribution of GDNF in brain was determined by immunohistochemistry (IHC) and by ELISA. The IHC was performed on serial sections of brain to compute the volume of distribution of GDNF in brain following CED in the primate. These results showed the neurotrophin distributed in a brain volume ranging from 87–360 mm^3^ [131]. This volume of distribution is comparable to the volume of distribution of transferrin following CED in the cat, which was ~100 mm^3^ [53]. As discussed above, this distribution volume is small compared to the volume of the putamen, which is 6000 mm^3^ on each side of the human brain [127]. The brain concentration of GDNF was measured by ELISA for each mm of distance removed from the CED catheter [131]. The brain concentration of GDNF decreases exponentially with each mm of distance removed from the CED catheter, which indicates the neurotophin is penetrating brain tissue by diffusion, not convection. The brain GDNF concentrations are shown in Figure 6A.

The data in Figure 6A show the region of brain most proximal to the catheter is exposed to GDNF concentrations that are log orders higher than the endogenous concentration of GDNF. High concentrations of GDNF may cause aberrant neuronal sprouting in brain [135]. CED of GDNF in the primate brain causes a local astrogliosis, as shown by GFAP immunohistochemistry (Figure 6B,C). It is not clear if this astrogliosis is due to the high local GDNF concentration, or if it is due to the CED delivery system.

CED was evaluated in a multi-centered randomized clinical trial of recurrent GBM treated with either a post-operative placement of carmustine wafer or the post-operative CED administration of cintredexin besudotox, which is a fusion protein of interleukin-13 and a mutated truncated form of the *Pseudomonas aeruginosa* exotoxin A [136]. CED of the toxin provided no clinical benefit and the target of a 2 cm penumbra around the CED catheter was met in only 20% of the patients [136]. The majority of infusions in the patients did not produce a significant coverage of the affected area [137]. A total of 15 CED clinical trials have been performed as of 2019 [129] without any advancement to drug approval. In an attempt to increase drug distribution in brain following CED, a variety of new approaches have been proposed, including the use of CED together with ultrasound [138], CED with newly designed catheters to enable the infusion of high volumes [139], the use of special catheters that infuse fluid simultaneously through 4 parallel ports [140], and the concurrent use of CED with pulsed electric currents applied to brain [141]. Real time MRI has been useful for the identification of reflux along the cannula, leakage of the infusate, and ventricular compression associated with CED [142].

## 3. Trans-Nasal Drug Delivery to Brain

The first report of drug movement from the nasal cavity to CSF was described 40 years ago [50]. Since then, over 1000 publications have evaluated drug delivery to brain and CSF via the nasal route (Table 1). However, to date, there is not a single FDA-approved drug for treatment of the brain parenchyma that is administered by the intra-nasal route [143]. To understand why 40 years of research on nasal delivery in animals has not translated to humans, it is useful to consider species differences in the anatomy of the nasal-cribriform plate. Species differences in the nasal cavity anatomy are consistent with the much greater role of olfaction in animals as compared to humans, as reflected in the number of olfactory receptor (OR) genes. OR genes comprise the largest multi-gene family in the mammalian genome, and constitute 4–5% of the mammalian proteome [144]. There are about 2000 OR genes in the rat [145], about 1000 OR genes in the mouse, but only about 400 OR genes in humans [144]. The olfactory region of the nasal mucosa, which is the site where drug must penetrate the nasal mucosa to enter olfactory CSF, constitutes 50% of the nasal cavity surface area in the rat, but only 3% of the nasal cavity surface area in humans [146]. Another factor limiting the translation of nasal drug delivery from animals to humans is the fact that the vast majority of preclinical investigations on nasal drug delivery to brain are performed with experimental designs that produce local nasal injury and membrane disruption, owing to the nasal instillation of very large volumes of drug. The introduction of >100 μL in the human nostril causes local injury to the nasal mucosa [147,148]. The volume of the nasal cavity in humans is 20 mL, but is only 0.4 mL in the rat, and only 0.03 mL in the mouse [148]. Therefore, by extrapolation, the nasal administration of a volume greater than 1% of the volume of the nasal cavity can induce local injury. A nasal administration volume of 1% of the nasal cavity volume would be 4 μL in the rat and 0.3 μL in the mouse. A review of the literature discussed below shows that the nasal administration volumes in preclinical nasal drug delivery research are 1–2 log orders higher than these injury related volume thresholds.

### 3.1. Drainage of CSF from Brain to Nose

**Figure 7:**
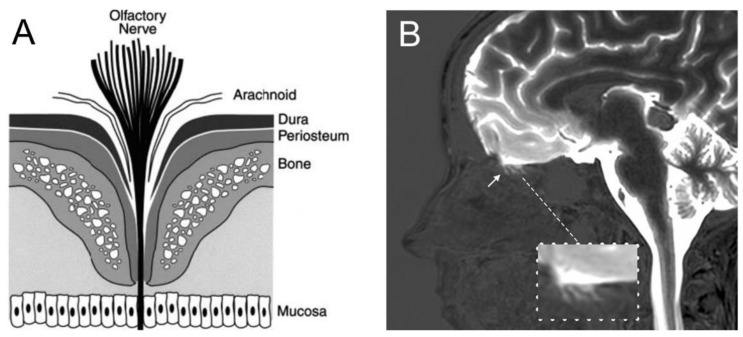
**Anatomy of the cribriform plate separation of the cranial and nasal cavities.** (**A**) The bipolar olfactory sensory neurons, which express the olfactory receptors, pass from the olfactory bulb to the nasal mucosa through the fenestrations of the cribriform plate. Reproduced with permission from [149], Copyright© 2003 JNS Publishing Group. (**B**) Magnetic resonance imaging (MRI) of the human head between 3–6 h following the injection into the lumbar CSF of gadobutrol, a macrocyclic gadolinium contrast agent. The gadolinium is visualized throughout the cerebral subarachnoid space within the convexities of the cerebrum, around the spinal cord, and is observed to penetrate into the superior regions of the fenestrations of the cribriform plate, which is magnified in the inset. No gadolinium passes into the inferior regions of the cribriform fenestrations, or into the nasal mucosa in humans (inset). Reproduced from [150], Copyright© 2020 licensed under Creative Commons Attribution License (CC-BY).

There is evidence from animal models that CSF drains from the subarachnoid space of brain into the nasal mucosa, and then to the lymphatic system. The anatomy of the olfactory nerves and arachnoid, cribriform plate, and nasal mucosa is shown in Figure 7A.

The evidence for the existence of a pathway of fluid flow from the CSF compartment of brain to the nose is less convincing for living humans. In 1937, Faber [151] observed in rabbits the movement of radiographic contrast agent from the CSF of the cisterna magna to the nasal cavity. The mechanism of this transfer was clarified by the injection of radio-iodinated albumin into the lateral ventricle of the rabbit [152]. A fraction of the radioactivity was recovered in the peripheral lymph, and this transfer to lymph was blocked by sealing the cribriform plate. Albumin, or at least the radioactivity, was demonstrated to pass from the CSF compartment of brain through the cribriform plate to the interstitial space of the olfactory submucosa or lamina propria [152]. The anatomical features enabling movement of olfactory CSF from above the cribriform plate to below the cribriform plate were examined at autopsy for the post-mortem human brain [149], as shown in Figure 7A. The olfactory CSF within the subarachnoid space of the olfactory bulb moves in parallel with the invaginations of the olfactory arachnoid membrane and dura around the olfactory nerves, which pass from the olfactory bulb to the nasal mucosa through the fenestrations of the neural foramina of the cribriform plate. The arachnoid membrane peels away from the olfactory nerve at 1–2 mm into these foramina, where the dura becomes continuous with the periosteum of the cribriform plate [149].

In the late embryonic rat, there are ‘olfactory bulb holes’, or disruptions of the arachnoid membrane at the cribriform fenestrations, which allow for drainage of olfactory CSF into the nasal mucosa [153]. However, the ultrastructural details of the junctions between the arachnoid, dura, and olfactory nerve at the proximal part of the cribriform fenestrations, and confirmation of holes in the olfactory arachnoid are not available for humans. The arachnoid membrane has high resistance tight junctions [149], similar to the endothelial junctions that form the BBB (Figure 4). The extent to which tight junctional complexes exist within the proximal part of the cribriform fenestrations is not known. An MRI study [150] in humans shows a gadolinium contrast agent that is injected into the CSF enters the proximal part of the cribriform fenestrations, but does not complete passage though these fenestrations, nor enter into the nasal submucosa (Figure 7B). This recent study in living humans does not confirm the hypothesis generated in animal investigations that CSF passes from the olfactory CSF into the nasal mucosa. Such passage of CSF from the olfactory region to the nasal submucosa would be a form of chronic subclinical CSF rhinorrhea, a condition associated in humans with local trauma to the cribriform plate [154].

### 3.2. Drug Delivery from Nose to Brain

Drug transport from nose to olfactory CSF involves drug transfer across 2 epithelial barriers, both of which are membrane barriers with tight junctions, and these barriers are the nasal epithelium and the arachnoid membrane. Therefore, drug delivery from nose to olfactory CSF is governed by the same principles that determine BBB transport. As discussed below is Section 6.1, lipid-soluble small molecules with a MW < 450 Da traverse these barriers by free diffusion. Any drug with a MW > 450 Da can only traverse the barrier by either (a) carrier- or receptor-mediated transport, or (b) membrane disruption, e.g., by local injury to the nasal mucosa. Similar to the BBB, the transport of small molecules from the nasal cavity to CSF is proportional to lipid solubility [155]. In 1982, progesterone was administered trans-nasally in primates, and the area under the concentration curve (AUC) in CSF was about twice as high as the AUC in CSF following IV administration [50]. These results were interpreted as evidence for a pathway of drug delivery from nose to CSF. However, the diluent injected in the nose in this study was 30% ethanol/30% propylene glycol [50], which may have had a solvent effect at the nasal mucosa. In a study in the rat, progesterone was administered via the nasal route in a saline diluent, and the AUC in CSF of progesterone was identical after IV or intra-nasal delivery [156]. Similarly, melatonin [157] and vitamin B12 [147] were administered via the intra-nasal and IV routes, and the AUC in CSF was identical for either form of delivery. If the drug passed directly from the nose to the olfactory CSF, then the AUC in the CSF should be higher after the nasal route as compared to the IV route. Conversely, the finding of a comparable AUC in CSF after either the IV or the nasal route indicates the drug passes across the nasal epithelium, enters blood, and then traverses the choroid plexus at the blood–CSF barrier, to enter the CSF compartment. A 1990 study on the potential delivery of a novel cognitive enhancer for Alzheimer’s disease (AD) via the trans-nasal route showed the brain AUC of the drug was the same following either IV or trans-nasal administration [158]. In 1991, the brain concentration of dextromethorphan was shown to be the same whether this drug was administered by the IV or the trans-nasal route [159]. In 2008, it was shown that the ratio of brain AUC/plasma AUC for diazepam was comparable following drug administration via either the IV or the trans-nasal routes [160].

Despite the early work showing the trans-nasal route conferred no selective delivery of drug to CSF, the number of investigations of the trans-nasal route grew exponentially, and 98% of the 1024 publications on trans-nasal brain drug delivery (Table 1) were published after 2000. Trans-nasal drug delivery to CSF succeeded once large volumes of drug were applied to the nasal cavity, particularly in rats and mice, where the olfactory region constitutes 50% of the nasal cavity surface area [146]. These large volumes induced local injury and disruption of the nasal barriers, similarly to the attempts to enhance brain drug delivery via BBB disruption as reviewed in Section 4. The instillation of drug volumes greater than 100 μL per naris in humans causes local injury [147] and the preferred volume for nasal administration in humans is as low as 25 μL per naris [161]. These volumes are <1% of the nasal cavity in humans. However, in studies in rats and mice the volume of drug introduced into the nose is typically 25–50 μL, which is very large compared to the volume of the nasal cavity in the rat, 400 μL, or the mouse, 30 μL [148]. The introduction of large volumes into the naris can lead to drug loss into the oral cavity via drainage through the naso-palatine duct in rodents, although this duct is a vestigial organ in adult humans. Therefore, the naso-palatine duct was blocked prior to the nasal introduction of 50 μL in the rat [162]. In this 1999 study, the intra-nasal administration of a volume of 50 μL in the rat, which is 12% of the nasal cavity volume, produced a higher CSF concentration of 5-fluorouracil than the CSF drug concentration produced by IV administration [162].

The administration of 5 μL in the naris of the mouse, which is a volume that is 15% of the nasal cavity in the mouse, resulted in the delivery of dopamine to the olfactory bulb of brain [163]. In a similar study, a 5 μL volume of picolinic acid in the naris of the mouse resulted in drug delivery to the olfactory bulb [164]. Drug delivery to brain of either dopamine or picolinic acid was measured by film autoradiography, which showed the olfactory bulb was the only region of brain penetrated by the drug following the instillation of large volumes into the mouse nose [163,164]. In a recent study, insulin was injected into the naris of a mouse in a volume of 25 μL [165], which is nearly equal to the entire nasal cavity in the mouse. Conversely, no delivery of IGF-1 to brain was produced following the intra-nasal administration of the peptide in a large volume, 35 μL, in the mouse [166]. In addition to the introduction of large volumes into the nasal cavity, other means of membrane destabilization have been employed to enhance drug delivery across the nasal barriers. Carbamazepine was administered by either the IV or trans-nasal routes in the rat; for nasal administration the drug was co-administered in an unspecified volume with 50 mg of a gel composed of the Carbopol 974P bioadhesive [167]. Other nasal enhancers that have been employed include 0.5% peppermint oil [168] and 2% polysorbate-80 [169].

In keeping with the analogy of drug delivery across the nasal barrier and the BBB, a preferred approach is not membrane disruption, but rather modification of the drug so that the drug becomes a ligand for endocytosis. As discussed in Section 7, cationic agents may undergo endocytosis into the brain capillary endothelium via absorptive-mediated endocytosis. Lectins, such as wheat germ agglutinin (WGA), can also undergo absorptive-mediated endocytosis into the brain endothelium [170]. A conjugate of WGA and HRP, but not unconjugated HRP, distributes to the olfactory lobe in rats following the intranasal administration of 25–50 μL into each naris of a 1% solution [171]. Cationization of a biologic also facilitates entry into CSF following intra-nasal administration. The lysosomal enzyme, iduronidase (IDUA), was cationized by conjugation of guanidinylated neomycin (Gneo) to the enzyme [172]. Guanidinylation of neomycin converts all 6 amino groups to positively charged guanidine moieties. Gneo is endocytosed by cells via absorptive-mediated endocytosis, similar to other polycations, such as poly-arginine [173]. Gneo-IDUA was infused into the nose in a volume of 50 μL in IDUA-null mice, and CSF enzyme activity was measured [172]. Gneo-IDUA entered the CSF, which peaked at 1 h and over 90% of the enzyme was cleared from CSF by 4 h after injection [172]. IDUA was also detected in the olfactory bulb, and the enzyme activity in this region of brain was >10-fold higher than any other region of brain [172].

Drug distribution following trans-nasal administration has been investigated by PET. The glucose analog, [^18^F]-fluorodeoxyglucose (FDG), was administered in the rat in volumes of 5 μL [174]. FDG entered only the inferior part of the turbinates of the cribriform plate and did not reach the superior part of the turbinate, and did not enter into the olfactory bulb [174]. In another PET study in the rat, [^11^C]-orexin A, a 33-amino acid peptide that is a potential treatment of narcolepsy, was injected in the nose in an unspecified volume using a Precision Olfactory Delivery system [175]. PET imaging showed the only part of brain exposed to the peptide was the olfactory bulb ipsilateral to the injected naris [175].

In summary, when low volumes of drug are instilled into the nose, and local injury to the nasal mucosa is not produced, then there is little evidence for drug delivery to the olfactory CSF, much less the brain following trans-nasal drug delivery. Predictably, clinical trials in humans of trans-nasal drug delivery have failed to demonstrated selective drug distribution into CSF via the nasal route, as discussed in the next section.

### 3.3. Clinical Trials of Trans-Nasal Drug Delivery to Brain

The peptide, oxytocin, was administered by the nasal route in patients with autism, in a double-blind placebo-controlled randomized clinical trial (RCT), and this treatment showed no clinical efficacy in either autism [176] or early psychosis [177]. In a second autism trial of intra-nasal oxytocin, there was a significant effect on overt emotion salience, but the drug had no dose response effect on this symptom [178]. After these negative clinical trials, the distribution of oxytocin into the CSF of primates was measured, and identical distribution of peptide into CSF was observed with either the IV or trans-nasal route of administration [179]. A follow-up RCT of nasal oxytocin in autism showed no clinical benefit [180]. A RCT of trans-nasal delivery of insulin for AD showed no clinical benefit [181]. In this trial, the insulin was delivered as a nebulized stream through a nosepiece left in the nostril for 20 s, and 40 units of insulin was delivered daily for 12 months. Treatment caused no increase in CSF insulin [181]. To date, no RCT has shown a clinical benefit in a CNS disease using the trans-nasal route of brain drug delivery. A recent review of the literature shows the trans-nasal route results in no consistent increase in brain delivery of small molecules in animal models [182].

## 4. Brain Drug Delivery with Blood–Brain Barrier Disruption (BBBD)

### 4.1. BBBD Following Intra-Carotid Arterial Infusion

#### 4.1.1. BBBD with Intra-Arterial Hyper-Osmolar Solutions

The disruption of the BBB following the intra-arterial infusion of hypertonic solutions was first demonstrated by Broman in 1945 [183] and by Rapoport in 1970 [184]. In 1973, Brightman et al. [185] showed the disruption of the BBB following the intra-carotid arterial infusion of hypertonic urea caused the shrinkage of brain endothelial cells in association with opening of endothelial tight junctions. In 1979, three groups used hyperosmolar BBBD to enhance brain uptake of therapeutics such as methotrexate [49,186], or an enzyme [187], following the intra-arterial infusion of a poorly diffusible hypertonic monosaccharide, mannitol or arabinose. BBB delivery of a drug across the disrupted BBB was shown to be dependent on MW, which is consistent with a pore mechanism associated with opening of tight junctions [188]. Hypertonic BBBD caused an increase in brain ornithine decarboxylase (ODC) activity and increased brain polyamines, and the disruption of the BBB was blocked by an ODC inhibitor, α-difluoromethylornithine (DFMO) [189]. BBBD mediated by arterial hyperosmolarity requires an intact BBB to establish the osmotic gradients across the endothelium. Consequently, the BBB in normal brain is disrupted to a greater extent than in a brain tumor, where there may be a pre-existing disruption of the BBB [190]. This reduces the therapeutic index of the BBBD, as the toxic effect in normal brain is greater than the therapeutic effect in the brain tumor [190]. These findings in an experimental rat brain tumor model [190] were replicated in humans with a malignant glial tumor [191]. The BBB permeability–surface area (PS) product, also called the K_1_, was measured for 82-rubidium with PET scans of the brain. Intra-carotid arterial infusion of 25% (1.4 M) mannitol was performed for 30 s at high flow rates (360–720 mL/min) under anesthesia and seizure prophylaxis with phenobarbital or phenytoin. The rubidium K_1_ increased 17-fold in normal brain, but was unchanged in the tumor brain. The T_1/2_ for return to normal BBB permeability was 8 min [191]. In contrast to hyper-osmolar BBBD, which preferentially effects normal brain, as compared to brain tumor, the intra-arterial infusion of vasoactive agents, such as bradykinin analogues, preferentially opens the BBB in tumor as compared to normal brain [192], as discussed in the next section.

Hyperosmolar BBBD, in combination with intra-arterial methotrexate, was demonstrated to enhance survival in patients with primary CNS lymphoma (PCNSL) [193]. At that time, PCNSL was known to be treatable with high dose methotrexate (HD-MTX) with leucovorin (folinic acid) rescue [194]. The efficacy of BBBD with hypertonic mannitol and intra-arterial methotrexate for PCNSL was confirmed in a larger cohort of patients [195], and is still in practice today [196]. There still is no FDA market approval for this approach, as a controlled clinical trial comparing intra-arterial mannitol/methotrexate vs. intra-arterial methotrexate has apparently not been performed in PCNSL.

Intra-carotid arterial hyperosmolar mannitol (ICAHM) administration is an aggressive intervention that is not without toxic sequelae. When the BBB is disrupted for the purpose of chemotherapeutic delivery to brain, virtually all substances in plasma, at least up to the size of the 420,000 Da fibrinogen, may also escape from blood to the brain ECS. Albumin is toxic to astrocytes, which can trigger a glial scar [197], and is pro-inflammatory [198]. Fibrinogen activates oligodendrocyte progenitor cells, which can lead to suppression of myelin production [199]. ICAHM induces a sterile inflammatory response (SIR) in brain, which is associated with activated astrocytes and microglia, an up-regulation of cytokines, chemokines, and trophic factors, and these changes are observed in the contralateral hemisphere, as well as the cerebral hemisphere ipsilateral to the infusion [200]. A similar SIR is observed with ultrasound-induced BBBD [201], as discussed below. Hyperosmolar BBBD causes vascular changes in brain [202], and chronic neuropathologic changes [203], owing to the brain uptake of plasma proteins that are normally excluded from brain by the BBB [204]. Peri-operative effects occur following ICAHM with a 13% incidence of seizures [205], despite pre-operative administration of anticonvulsants [206]. Electroencephalogram (EEG) abnormalities are used to monitor BBBD intra-operatively [207].

#### 4.1.2. BBBD with Intra-Arterial Bradykinin Analogs

The topical application of bradykinin (BK) to the pial surface of brain causes BBBD to small molecules such as fluorescein but not to large molecules such as albumin [208]. Since topical application of BK to the surface of the brain induces a pharmacologic effect, it is inferred that the BK receptor is expressed on the abluminal side of the BBB. It is not practical to use BK as an agent to induce BBBD as this peptide is rapidly degraded and has a plasma T_1/2_ of about 15 s, owing to first pass inactivation in the lung by angiotensin converting enzyme (ACE) [209]. A more metabolically stable analogue of BK was developed, RMP-7 [210], as an agent to induce biochemical BBBD, where RMP = receptor-mediated permeabilizer. Intra-carotid arterial administration of RMP-7 caused selective BBBD in an experimental brain tumor, as compared to normal brain [211]. This effect is opposite of hyperosmolar BBBD, which opens the BBB in normal brain to a much greater extent than in a brain tumor [191]. Capillaries perfusing the core of brain tumors are often leaky, which enables intra-arterial BK analogues to access the BK receptors on the abluminal side of the BBB in brain tumors. The carotid arterial infusion dose of RMP-7 was 0.1 μg/kg, as higher doses, 1 μg/kg, caused hypotension [211]. In humans with malignant glioma, intra-arterial RMP-7, at a dose of 10–300 ng/kg, caused an increase in the PS product of [^68^Ga]-EDTA of 46 ± 42% as determined by PET [212]. The cause of the wide variability in the opening of the blood–tumor barrier (BTB) is not clear, but may be related to differences in the degree of disruption of the BTB in brain tumors. Intra-arterial RMP-7 caused no BBBD in normal brain in humans [212], which is consistent with an abluminal expression of the BK receptor. Intravenous administration of RMP-7 caused no BBBD in dogs [213] or rats [214]. Intravenous RMP-7 did not increase the clinical efficacy of carboplatin in a RCT of malignant glioma [215]. The applications of RMP-7 proved to be limited to the intra-carotid arterial infusion route in conditions with a pre-existing BBBD, e.g., malignant gliomas. Given this limited scope of clinical applications, the drug development of RMP-7 was terminated in the 1990s.

### 4.2. BBBD Following Intravenous Microbubble/Focused Ultrasound

Focused ultrasound (FUS) of the brain was described by Lynn et al. in 1942 in cats and dogs [216]. In 1960, Ballantine et al. [217] reviewed FUS of the brain and concluded that BBBD may be introduced without lesions of the surrounding parenchyma. However, high intensity FUS of brain was shown in 1968 to cause focal lesions in the CNS including vascular occlusion [218]. In 1995, attempts were made to modulate the sonication parameters so as to separate BBBD from parenchymal damage in brain [219]. FUS was combined with the IV administration of an ultrasonic contrast agent, which is composed of 2–4.5 micron microbubbles (MB) of Optison [66]. The Optison microbubble, as well as Definity microbubble, contains a gaseous interior of octafluropropane. The FUS-MB treatment produced BBBD in rabbits [66]. The anatomical basis of the BBBD caused by microbubbles/FUS was shown by electron microscopy to be both opening of tight junctions and enhanced vesicular transport [220,221]. The BBBD caused by the FUS-MB method enabled brain penetration of 3 kDa and 70 kDa dextran, but not 2000 kDa dextran, and the brain penetration of 3 kDa dextran exceeded that for 70 kDa dextran [222]. The brain uptake of 3 kDa, 70 kDa, 500 kDa, and 2000 kDa dextran was measured following the administration of 3 different FUS-MB protocols that varied the acoustic power from 0.31 Mpa, 0.51 Mpa, and 0.84 Mpa, where Mpa = mega Pascals [223]. There was minimal entry of any dextran with an acoustic pressure of 0.31 mPa. At an acoustic pressure of 0.51 mPa, the 3 kDa dextran entered the brain to an extent greater than the 70 kDa dextran, and the 500 kDa and 2000 kDa dextrans did not enter brain. At the acoustic pressure of 0.84 Mpa, all dextrans entered the brain, although the entry of the 500 kDa and 2000 kDa dextrans was nearly background [223]. The gyration radii of 4 kDa, 70 kDa, 500 kDa, and 2000 kDa dextran are 2.2 nm, 9 nm, 10.5 nm, and 58 nm, respectively [224]. Therefore, the diameter of the pore created by FUS-MB treatment at an acoustic pressure of 0.51 Mpa is about 20 nm, which is of sufficient size for entry of plasma proteins and therapeutic antibodies, which have a diameter of 10–11 nm [225]. The opening of the BBB following FUS-MB is on the order of hours, and the BBBD is resolved by 6–24 h [226]. The extent to which the BBB is disrupted is a function of both the acoustic power applied to brain, and the injection dose (ID) of the microbubbles. The higher the acoustic power, and the higher the ID of the microbubble, the greater the disruption of the BBB [227]. If the acoustic power is increased from 0.53 Mpa to 0.64 Mpa, and the ID of the microbubbles is increased from 0.1 to 0.5 mL/kg, then even a 200 nm pegylated liposome enters the brain [227]. However, as the ID of the microbubble is increased from 0.15 mL/kg to 0.4 mL/kg, neuropathologic effects on brain are observed including neuronal apoptosis and intra-cerebral hemorrhage [228], and the neurotoxicity of FUS-MB treatment is discussed below.

Th FUS-MB approach to BBBD has entered into phase 1–2 clinical trials. Patients with recurrent GBM were treated with a FUS dose escalation from 0.5 mPa to 1.1 Mpa, in conjunction with an ID of 0.1 mL/kg of SonoVue microbubbles, which contain a hexafluoride gas [229]. The FUS was administered by an ultrasonic transducer implanted in the skull. No clinical efficacy was evaluated in this trial as no therapeutic was co-administered with the BBBD. In a phase 1 trial of recurrent malignant glioma in 5 patients, the FUS-MB-induced BBBD was performed in conjunction with doxorubicin/pegylated liposomes in 1 patient and temozolomide in 4 patients [230]. The ID of the Definity microbubble was up to 0.02 mL/kg. The FUS was administered by a helmet following shaving of the head and in the presence of stand-by anesthesia. The sonicated area was determined by contrast MRI of the head. The volume of the sonicated volume area of brain was about 0.5 mL, and a biopsy was performed of the sonicated and non-sonicated peri-tumor tissue. BBBD caused no increase in the brain concentration of either doxorubicin or temozolomide in this pilot study [230]. A phase 1 trial of FUS-MB treatment was performed in 6 subjects with recurrent glioblastoma [231]. In this acoustic power dose escalation study, the IV dose of microbubbles was 0.1 mL/kg. No drug was co-administered in this trial [231]. Clinical trials of the FUS-MB method of BBBD are being extended from life threatening focal disease of brain, such as malignant glioma, to chronic neurodegenerative disease of brain including Alzheimer’s disease (AD) [232] and amyotrophic lateral sclerosis (ALS) [233]. In these phase 1 trials, no therapeutic was co-administered with the FUS-MB. To date, no clinical trial has demonstrated any clinical efficacy of the FUS-MB method of BBBD in human CNS disease.

The neuropathology of hyperosmolar BBBD is discussed in Section 4.1.1, and a similar profile of neuropathology is caused by the FUS-MB method of BBBD. In both cases, the brain parenchyma is exposed to serum protein, which enter brain following BBBD. The FUS-MB treatment causes cell uptake of albumin, which is associated with activation of astrocytes and microglia in brain [234]. Similar to the sterile inflammatory response (SIR) caused by hyperosmolar BBBD [200], the FUS-MB form of BBBD also causes an SIR in brain [201]. Albumin entry into the parenchyma of brain induces neuroinflammation triggered by the NFκB pathway [201]. This SIR response, which is similar to that observed in cerebral ischemia or traumatic brain injury [201], is associated with the up-regulation of >1000 genes within 6–24 h of the FUS-MB treatment [235]. A recent review [236] suggests that optimization of ultrasound parameters may allow for “safe” BBBD. However, the FUS-MB method of BBBD seems to be an example of a therapy with a therapeutic index of 1. If the BBB is disrupted, so as to allow the entry into brain of a therapeutic, the parallel entry of plasma proteins, which are toxic to brain, is inescapable.

### 4.3. Miscellaneous Forms of BBBD

BBBD via intra-carotid arterial hyperosmolar mannitol (ICAHM), or via focused ultrasound-microbubbles (FUS-MB), are aggressive approaches to brain drug delivery that require treatment in the operating room either under anesthesia, in the case of ICAHM, or with standby anesthesia, in the case of FUS-MB. Attempts have been made to produce BBBD biochemically, which gave rise to the development of RMP-7. However, RMP-7 administration required an interventional radiologist for intra-carotid arterial administration, and only worked in conditions with a pre-existing BBBD such as advanced malignant gliomas [212]. A kind of ‘holy grail’ in brain pharmaceutics is the development of a non-invasive form of BBBD following IV administration. The effort to disrupt the BBB as a therapeutic intervention is paradoxical, because the BBB “allows for maintenance of homeostasis of the CNS milieu” [237]. If this is true, then would not BBBD have serious toxicity in the brain? Indeed, the most developed forms of BBBD, ICAHM and FUS-MB, both cause a non-infectious inflammation in brain called a sterile inflammatory response (SIR) [200,201,235]. ICAHM was demonstrated over 30 years ago to cause vascular pathology in brain [202], and chronic neuropathologic effects in brain [203]. One of the earliest forms of BBBD described was the intra-carotid artery injection of micellar forming concentrations of common neuropsychiatric drugs, nortriptyline and chlorpromazine [238]. However, this arterial drug-induced BBBD was clearly a toxicologic effect of very high concentrations of these drugs. The miscellaneous forms of BBBD described below are also toxicologic, not therapeutic, approaches to brain drug delivery.

#### 4.3.1. BBBD with Tight Junction Modulators

Both ICAHM and FUS-MB disrupt the BBB by opening tight junctions [185,221]. Alternative approaches to opening tight junctions have been proposed, and these can be classified as to whether the agent is administered by the intravenous or the intra-arterial route. The Ser-His-Ala-Val-Ser (SHAVS) pentapeptide includes the HAV tripeptide sequence from the extracellular domain (ECD) of E-cadherin, a cell adhesion molecule involved in tight junction formation. A cyclic version of the pentapeptide was more stable in plasma [239]. The cyclic pentapeptide affected epithelial resistance in cell culture at high peptide concentration of 0.5–1 mM, and IV administration of the cyclic peptide in mice increased the brain uptake of gadolinium on MRI [239]. The co-administration of the linear form of the SHAVS peptide and peroxiredoxin-1, an anti-oxidant enzyme, reduced tumor growth in a mouse medulloblastoma tumor model [240]. The intra-arterial infusion of a high concentration, 1 mM, of the linear form of the SHAVS peptide increases mannitol uptake by brain [241].

A monoclonal antibody (MAb) against claudin-5 (CLDN5), a tight junction protein, caused BBBD in a cell culture model [242]. IV administration of this MAb in primates at an injection dose (ID) of 3 mg/kg caused increased uptake of fluorescein in the CSF. However, the MAb has a narrow therapeutic index, as a dose of 6 mg/kg of the CLDN5 MAb induced convulsions in monkeys [243].

Angubindin-1 is a 200-amino acid fragment derived from the *Clostridium perfringens* iota-toxin, and it binds tricellulin, a component of tricellular tight junctions [244]. Angubindin-1 was expressed as a glutathione S-transferase (GST) fusion protein, and was administered IV to mice at an ID of 10 mg/kg. This treatment increased the brain uptake of an antisense oligodexoynucleotide [244].

The intra-carotid arterial infusion of membrane active agents can also induce BBBD by interference with tight junctions. Arterial infusion of 30 mM caproic acid, a 10-carbon monocarboxylic acid, for 30 s causes enhanced transport of mannitol across the BBB; however, 45–90 s infusions of caproic acid caused brain edema [245]. An emulsion of triolein, a neutral triglyceride, causes BBBD following a 3 min manual carotid arterial infusion over 60 s in the rat [246]. If BBBD enables the entry of plasma proteins into brain parenchyma, this produces, by definition, the vasogenic form of brain edema [247].

#### 4.3.2. BBBD with Adenosine Analogues

Regadenoson is an adenosine analogue that is FDA approved for cardiac stress tests, and is administered as an IV dose of ~5 µg/kg [248]. Regadenoson is an adenosine receptor (AR) agonist, and the IV injection of 1 µg/kg in rats produces only a marginal increase in the brain uptake of 10 kDa dextran [249]. This may be due to the very rapid plasma clearance of regadenoson, which has a plasma T_1/2_ of only 2–4 min [250]. The IV injection of 3 sequential doses of regadenoson enhances brain uptake of 10 kDa dextran [249]. It is not clear how regadenoson causes BBBD, since this drug, 5′-N-ethylcarboxamide adenosine (NECA), has a MW of 390 Da and forms 12 hydrogen bonds with water. As discussed in Section 6.1.2, small polar molecules with these properties do not cross the BBB. An adenosine transporter is expressed on the BBB, as discussed in Section 6.2.5, but it is not clear if NECA is a ligand for the adenosine transporter. Regadenoson, like RMP-7, may be effective in the treatment of brain tumors that have a pre-existing leakage of the BBB. Rats with an F344 experimental brain tumor were treated with oral temozolomide, 50 mg/kg, in conjunction with an IV dose of Regadenoson of 0.5 µg/kg, and the BBBD increased the tumor/plasma temozolomide ratio by 55% [250].

#### 4.3.3. BBBD with Anti-Bacteria Antibodies

A MAb, designated 13.6E1, against the filamentous hemagglutin of *Bordetella pertussis* was injected intravenously in rabbits at a dose of 30 µg/kg, and this produced BBBD, as measured by the increased brain uptake of penicillin [251]. Immunohistochemistry showed the MAb bound to the vasculature of human and rabbit brain [251]. Complete Freund’s adjuvant (CFA), which is composed of inactivated mycobacteria, causes BBBD in mice, manifested by brain uptake of circulating IgG for 2–3 weeks, following a subcutaneous (SQ) injection of CFA [252]. The primary antigen of the mycobacterial cell wall is lipoarabinomannan, and an IgM anti-mannan MAb was generated [253]. Following the IV injection of 2 mg of the anti-mannan MAb in the rat, BBBD and vasogenic edema were observed, in parallel with global brain uptake of gadolinium by MRI [253]. The BBBD resolved within 24 h. BBBD is observed in experimental autoimmune encephalomyelitis (EAE), which is produced following the SQ injection of guinea pig brain extract mixed in complete Freund’s adjuvant [254]. The BBBD is this model is nearly eliminated by either neuro-intermediate pituitary lobectomy or an arginine vasopressin (AVP) receptor blocker, conivaptan [254]. This study implicates the role of the V1a or V2 AVP receptors in the BBBD associated with EAE. Similarly, the vasogenic brain edema and BBBD that follows permanent occlusion of the middle cerebral artery in experimental stroke was reduced by conivaptan administration [255].

#### 4.3.4. BBBD with Intra-Arterial Polycations

The carotid arterial infusion of 50–500 µg/mL protamine sulfate for 1–2 min in the rat results in BBBD, associated with increase brain uptake of HRP in parallel with opening of brain endothelial tight junctions [256]. A similar finding was observed in rabbits following the carotid arterial infusion of protamine sulfate, and the BBBD to albumin caused by the arterial infusion of protamine was attenuated by the co-infusion of the anionic heparin, which neutralizes the cationic protamine [257]. No BBBD was induced by protamine if the polycationic agent was administered by the intravenous route. The arterial infusion of other polycationic agents, including poly-arginine, poly-lysine [258] or histone [259] similarly caused BBBD. The BBBD induced by the intra-arterial infusion of polycations is toxic, as the intra-arterial protamine infusion led to spongiotic shrunken nerve cells in brain [260].

#### 4.3.5. BBBD with Intra-Arterial Amphipathic Agents

Amphipathic agents form micelles at concentrations above the critical micellar concentration (CMC), and can disrupt the permeability of membranes [261], including the BBB [238]. The intra-carotid arterial infusion of dehydrocholate, an oxidized bile salt, causes BBBD to albumin [262]. Similar to hyperosmolar BBBD, which causes changes in the EEG [207], BBBD due to dehydrocholate administration also causes changes in the EEG [262]. The carotid arterial infusion of 20 µM oleic acid, an 18-carbon omega-9 free fatty acid (FFA), causes BBBD to albumin [263]. The BBBD caused by the administration of alkylglycerols was developed as a new brain drug delivery strategy [264]. However, alkylglycerols, such as 1-O-hexyldigylcerol or 1-O-heptyltriglycerol, cause BBBD only after carotid arterial infusion, and not after IV administration [265]. Only high 80 mM concentrations of alkylglycerols in the arterial infusate caused BBBD to small molecules. These concentrations exceed the CMC and cause the formation of vesicles, which appear to be the mechanism of increased BBB permeability [265]. Melittin, a 26 amino acid anti-microbial peptide from bee venom, has recently been suggested as a new brain drug delivery strategy, as intra-carotid arterial infusion of 3 µM melittin causes BBBD [266]. However, melittin is unlikely to have an acceptable safety profile, as this peptide is known to alter membrane permeability by inducing the formation of membrane holes [267]. These holes form when the concentration of the peptide reaches a threshold ratio of peptide to membrane lipid [267]. Other membrane active agents that have been proposed as new brain drug delivery strategies are L-borneol [268] and NEO100 [269], which have similar organic alcohol amphipathic structures. The oral administration of 1200 mg/kg of L-borneol causes BBBD to Evans blue/albumin [268]. However, this dose is near the 50% lethal dose (LD50) of L-borneol, which is 300–5800 mg/kg in rodents [270]. NEO100 is perillyl alcohol, and the intra-carotid arterial infusion of 20 mM NEO100 causes BBBD to albumin [269]. NEO100 produces no BBBD following IV administration [269], similar to the alkylglycerols [265].

#### 4.3.6. BBBD and Free Radicals

Methamphetamine causes BBBD in association with the formation of free radicals, and the effect on the BBB is attenuated by Trolox, an anti-oxidant water-soluble analogue of vitamin E [271]. The effect of methamphetamine on BBBD is not observed in the caveolin-1 knockout mouse [272]. Methamphetamine, a highly addictive drug of abuse, has been recently suggested as a new brain drug delivery strategy [272], which follows the original suggestion of this idea made by Kast in 2007 [273]. The methamphetamine-induced formation of reactive oxygen species in cultured endothelium is blocked by the anti-oxidant, N-tertbutyl-α-phenylnitrone (PBN) [274]. PBN was originally tested in a failed phase 3 clinical trial in stroke [275], and the drug development of PBN for stroke was terminated [276]. PBN has a very low BBB PS product of only 0.1 µL/min/g [277], which approximates the BBB PS produce for sucrose [278], which indicates there is minimal, if any, BBB transport of PBN. More recently, PBN, also called OKN-007, has been shown to cause BBBD to gadolinium in the rat following the IV administration of 18 mg/kg. BBBD peaked at 2 h and returned to baseline at 4 h [279].

#### 4.3.7. BBBD and Electromagnetic Radiation

The strength of an electric field can vary widely depending on the source, e.g., television, cell phone, microwave, or radar. Exposure of male rats to ~1 GHz of radio-frequency radiation, which is comparable to the radiation emitted by a cell phone, causes BBBD [280]. The emission from a 5G cell phone is even higher [281]. Recently, the BBBD caused by exposure to pulsed electric fields (PEF) has been proposed as a new approach to brain drug delivery, which is considered to have advantages over FUS-MB, as no administration of microbubbles is required. BBBD in tumor-bearing rats was caused by repetitive electromagnetic pulses of 2.5 kV/m [282]. Focal BBBD was produced by the placement of an intra-cerebral probe that generated PEFs of ~1 kV/m [283]. Presumably, the requisite preclinical toxicologic evaluations of The PEF technology will be performed prior to human clinical trials.

In conclusion, BBBD, by any means, is a drug delivery technology that likely has a therapeutic index of 1. If the BBB is disrupted, for the purpose of brain drug delivery, then BBB is also disrupted to plasma proteins, or other agents in blood, that induce neurotoxicity.

## 5. Cell-Mediated BBB Transport

### 5.1. Stem Cells for Brain Drug Delivery

Mesenchymal stem cells (MSC), such as those derived from bone marrow, are said to cross the BBB and, therefore, to offer the potential as a conduit for brain drug delivery [284]. However, the studies cited [285,286] as evidence for BBB transport of MSCs do not support the hypothesis that stem cells cross the intact BBB. In one study of a spinal cord lesion model, the stem cells were injected directly into the spinal cord, thus bypassing the BBB [285]. In the other cited study, stem cells were injected intravenously at 1–6 weeks following a contusion spinal cord injury (SCI) model [286]. However, the blood-spinal cord barrier is disrupted for weeks in contusion SCI models [287]. These findings show there is no evidence that stem cells cross the BBB as discussed in a recent review [288]. Early work examined the distribution of stem cells in brain after IV injection, and observed that no stem cells were detected in brain parenchyma, although stem cells invade the meninges of brain [289], where there is no BBB. The delivery of stem cells for the treatment of recovery from cerebral ischemia requires BBBD with intra-arterial mannitol [290]. Stem cell transplant (SCT) with MSCs is a primary form of treatment of infants with MPSI or Hurler syndrome, which is caused by mutations in the IDUA gene [291]. SCT in MPSI reduces hydrocephalus [291], which is consistent with meningeal infiltration by the stem cells [289]. However, SCT in MPSI causes no reduction in CSF GAGs [292]. MSCs were permanently transfected with a cDNA encoding IDUA by lentiviral transfection, and injected in IDUA null MPSI mice [293]. The stem cells were transfected with lentivirus to a vector copy number (VCN) of 5–11. This VCN is considered high, whereas the FDA requires a VCN < 5 for human therapeutics, as the lentivirus is potentially mutagenic [294]. Despite the high VCN, the stem cell-lentiviral genome in brain was at the background level and log orders lower than in peripheral tissues [293]. Future forms of stem cell therapy will likely evolve toward the use of human-induced pluripotent stem cells (iPSC), which are reviewed in Section 11.7.2. However, at present, there is no evidence that iPSCs selectively cross the BBB relative to MSCs.

### 5.2. Exosomes for Brain Drug Delivery

The lack of success in brain drug delivery with stem cells has led to the development of exosomes as a brain drug delivery vehicle [295,296]. Exosomes are naturally occurring extracellular vesicles, which are released from the plasma membrane of cells, and may be taken up by neighboring cells. Exosomes are isolated from cultured cells. The cell debris is removed by centrifugation at 12,000× *g*, and the exosomes are harvested at 120,000× *g* [67]. For brain drug delivery, exosomes need a targeting mechanism so as to trigger receptor-mediated transcytosis (RMT) across the BBB. RMT is discussed below in Section 8.1. In the absence of a targeting mechanism built into the exosome, then BBB transport is minimal, unless the exosome naturally expresses a targeting ligand, as discussed below. In an early study on the brain drug delivery of short interfering RNA (siRNA), exosomes were prepared from bone marrow dendritic cells that had been permanently transfected with a cDNA encoding a fusion protein of lysosomal associated membrane protein 2b (Lamp2b) and a 29-amino acid peptide derived from the rabies virus glycoprotein (RVG). The RVG peptide is believed to trigger RMT across the BBB via the nicotinic acetylcholine receptor (nAChR) [297]. However, as discussed in Section 8.1.7, the IHC of brain shows no expression of the nAChR at the brain endothelium. The yield of exosomes from these transfected cells was 30 µg protein from 3 × 10^6^ cells [67]. Since the protein content of 10^6^ cells is 0.2 mg protein [298], the yield of exosomes from the cells was about 5%. The siRNA was encapsulated in the exosomes by electroporation [67]. The problem of encapsulating drugs into exosomes is the same as drug encapsulation in cells, and electroporation is required for a large molecule drug such as siRNA. The siRNA encapsulated in the RVG-targeted exosomes was injected into mice at a relatively large dose of siRNA of 6 mg/kg [67].

A limiting problem in exosome drug delivery is the encapsulation of the drug in the exosome. A hydrophobic small molecule can be passively encapsulated, but even a small molecule that is hydrophilic must be incorporated in the exosome by electroporation [299]. An alternative approach is to bind the drug to the surface of the exosome. This was performed for a siRNA therapeutic by engineering a fusion protein of the G58 domain of glyceraldehyde dehydrogenase, which binds the surface of exosomes and a RNA binding protein, trans-activation-responsive RNA-binding protein 2 (TARBP2), which binds double-stranded RNA, such as siRNA [300]. A three-way complex was then formed by mixing the exosomes derived from either mesenchymal stem cells or human embryonic kidney 293T cells, the G58-TARBP2 fusion protein, and the siRNA. IV administration in mice resulted in rapid clearance from the blood and a 10-fold higher uptake in peripheral organs as compared to brain [300]. The rapid clearance of exosomes from blood is the same pharmacokinetic (PK) problem that confounded early drug development of liposomes. After the IV administration of liposomes, the surface of the vesicles was coated by plasma proteins, which triggered uptake by cells lining the reticulo-endothelial system in liver and spleen. This problem of rapid clearance of liposomes was diminished by incorporation of polyethyleneglycol (PEG) in the surface of the liposome [301], which is discussed in Section 9. A post-insertion method for introducing PEG-lipids in pre-formed liposomes was developed [302], and presumably could be used with exosomes.

An alternative to the engineering of cell lines that produce targeting ligands on the exosome surface is the production of exosomes that naturally express a surface ligand that binds a receptor on the BBB. Exosomes derived from the SK-Mel-28 breast cancer cell line target the CD46 receptor [303]. CD46 is an inhibitory complement receptor that is expressed at the BBB and astrocyte foot processes [304]. Exosomes isolated from fresh mouse blood express the transferrin receptor (TfR), and loading of brain endothelial cells in culture with transferrin triggered uptake of the Tf-coated exosomes [305]. Exosomes were isolated from bone marrow macrophages, and the therapeutic, the recombinant TPP1 proenzyme, a 70 kDa lysosomal enzyme, was incorporated into the exosomes by sonication [306]. With no exosome targeting ligand, it was necessary to bypass the BBB, and to administer these exosomes by intrathecal injection [306].

Exosomes have generated considerable enthusiasm as a brain drug delivery system, and multiple review articles have been published on exosomes and brain delivery in just the last 3–4 years. However, it is not clear how this technology can be translated to human therapeutics, nor is it clear how exosomes offer advantages over synthetic nanocontainers, such as targeted pegylated immunoliposomes discussed in Section 10.2. Translation of exosome brain drug delivery technology to human therapeutics will require solutions to multiple problems including:

- Low yield of exosomes from the starting cell line. These yields are generally not provided in exosome publications, but may be on the order of only 5%, as discussed above.
- Poor PK properties, and rapid exosome removal from blood, similar to non-pegylated liposomes [301].
- Drug encapsulation in the exosomes requires procedures such as electroporation [67] or sonication [306], which is difficult to scale up for manufacturing. Passive loading will work only for hydrophobic small molecules [299]. Many therapeutics may leak out of exosomes on storage, similar to the drug leakage from liposomes [307].
- Exosomes will generally require a targeting ligand on the surface of the vesicle, so as to promote RMT across the BBB. The incorporation of such ligands will require genetic modification of the cell line used to produce the exosomes.
- The stability of exosomes is unknown. A 2-year shelf life at 4 °C typically needs to be established for biologics, and it is not clear if exosomes, which are composed of multiple membrane elements, have any significant degree of stability on storage. To what extent exosomes can be lyophilized and then re-solubilized with both high drug retention and BBB transport is not known.

## 6. Brain Drug Delivery of Small Molecules

### 6.1. Lipid-Mediated Transport of Small Molecules

#### 6.1.1. Approved Small Molecule Drugs for the CNS

A review of the 200 most-prescribed drugs in the United States shows that CNS drugs comprise 19% of these pharmaceuticals [308]. Of these 38 most-prescribed CNS drugs, 66% are for psychiatric conditions, including depression, psychosis, anxiety, and hyperactivity, and 21% are for epilepsy. Therefore, 87% of the most-prescribed CNS drugs cover only two classes of CNS disorders, neuropsychiatric conditions and epilepsy. The MW of these CNS active drugs ranges from 135 Da to 448 Da, with a mean ± SD of 276 ± 77 Da. Only 2 of the 38 drugs have a MW between 400–450 Da. The number of hydrogen bonds formed by these 38 drugs ranges from 2–6, and only 2 drugs form 6 hydrogen bonds with water. CNS drug development in 2022 has not really advanced beyond the post-World War II era of the 1950s, when the prototypes of present-day CNS pharmaceutics were developed, such as phenothiazines [40], tricyclic antidepressants [41], benzodiazepines [309], and phenytoin [310], i.e., treatments only for neuropsychiatric conditions and for epilepsy.

Only about 2% of small molecule drugs are active in the CNS [1]. This conclusion is drawn from the following reviews on small molecule CNS drugs. A survey of >6000 drugs in the Comprehensive Medicinal Chemistry database shows that only 6% of drugs are active in the CNS, and these drugs are generally confined to the treatment of psychiatric conditions and insomnia [311]. In another review of drugs, 12% were found to be active in the CNS, but if psychiatric disorders were excluded, only 1% of all drugs are active in the brain [312]. The 2% figure is a compromise between the fraction of all drugs active in the brain, 6–12%, and the fraction of drugs active in non-psychiatric conditions of brain, 1%. The reason that so few drugs are active in the CNS is that the type of small molecule that crosses the BBB via free diffusion must exhibit two necessary properties: (a) a MW < 450 Da [313], and (b) a structure that forms less than eight hydrogen bonds [314]. The vast majority of small molecule drug candidates lack these molecular properties and cannot be developed for CNS conditions.

#### 6.1.2. Mechanism of Small Molecule Diffusion through the BBB

**Figure 8:**
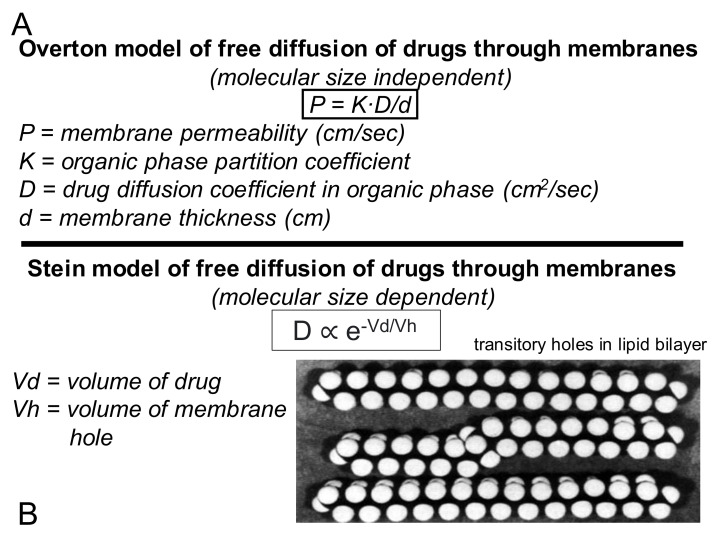
**Overton vs. Stein models of solute diffusion through membranes.** (**A**) Overton model of solute diffusion through biological membranes. Membrane permeability is independent of solute molecular size [315]. (**B**) Stein model of solute diffusion through membranes [317]. Membrane permeability is exponentially related to the molecular volume of the drug (Vd) relative to the volume (Vh) of transitory holes formed in the membrane. These membrane holes are formed by kinking of phospholipid fatty acyl side chains, as depicted in the model. Adapted from [15], Copyright© 2020 licensed under Creative Commons Attribution License (CC-BY).

The mechanism of small molecule diffusion through the BBB is the same as that which governs solute-free diffusion through biological membranes. For many years, it was believed that membrane permeability was proportional to lipid solubility, as reflected in the partition of the drug in a model solvent such as 1-octanol. Thus, measurement of the octanol partition coefficient (K) should predict membrane permeability as governed by the model of solute diffusion developed by Overton in 1901 [315]. The Overton model makes no allowance for solute size or MW. In 1980 Levin [316] observed that BBB permeability of drugs was proportional to lipid solubility providing the MW of the drug was <400 Da. This finding indicated that there was a threshold of MW governing BBB transport via free diffusion. The role of molecular size and MW in solute-free diffusion through lipid bilayers, as opposed to diffusion through a solvent, was formulated by Lieb and Stein [317]. The diffusion coefficient (D) of drug within a membrane was exponentially and inversely related to the size of the drug. The mechanism by which drug permeation through a biological membrane could be a function of solute size was put forward by Trauble in 1970 [318]. In this model, solutes penetrate a biological membrane by jumping through transitory holes in the membrane that are caused by the kinking of mobile fatty acyl side chains of membrane phospholipids, as depicted in Figure 8.

Solutes are hypothesized to traverse a biological membrane through a process of molecular “hitch-hiking”, through neighboring holes within the phospholipid bilayer until the solute traverses the membrane. The MW dependence of solute or drug free diffusion through a biological membrane is not predicted by the Overton model (Figure 8A), but is predicted by the Stein model (Figure 8B). If the MW or size of the drug is too large to fit into the membrane holes, then membrane permeation is decreased in proportion to molecular size. BBB permeability is decreased 100-fold when the cross-sectional area of the drug is increased from 52 Å^2^ to a cross-sectional area of 105 Å^2^ [319]. This exponential decrease in BBB permeation as the size of the drug increases comports with the models of Stein [317] and Trauble [318], as reviewed recently [320].

Apart from the molecular volume of the drug, as reflected in the MW, the other important factor limiting small molecule movement through membranes is polarity of the drug, as reflected in the number of hydrogen bonds formed between the drug and solvent water [317,321]. BBB permeation decreases by 1 log order for each pair of hydrogen bonds formed by functional groups on the drug as exemplified by either steroid hormones [322] or peptides [323]. The effect of hydrogen bonding on BBB transport is illustrated by the 1972 study of Oldendorf et al. [42]. The BBB transport of heroin, which is diacetylmorphine, is >10-fold faster than the BBB transport of morphine [42]. The acetylation of the 2 hydroxyl groups converts morphine to heroin, and removes a total of 4 hydrogen bonds from the parent drug.

In summary, the likelihood of BBB transport of a given small molecule can be estimated from the MW and structure of the drug. If the MW > 450 Da, and/or the structure of the drug includes polar functional groups that form >7 hydrogen bonds, then the BBB transport of the drug will be low, in the absence of carrier-mediated transport. Conversely, if the MW < 450 Da and the drug forms ≤7 hydrogen bonds with water, then the BBB transport of the drug may be significant, assuming the drug is not a substrate for an active efflux transporter, such as p-glycoprotein, as reviewed in Section 6.3.2.

#### 6.1.3. Lipid-Soluble Pro-Drugs

The BBB transport of morphine is increased nearly 100-fold by acetylation of both hydroxyl groups on morphine to form heroin [42]. Heroin is a morphine prodrug. However, the development of CNS prodrugs has proven to be difficult, and there are few FDA-approved CNS drugs wherein medicinal chemistry was used to convert a hydrophilic CNS drug, that does not cross the BBB, to a lipid-soluble prodrug that does cross the BBB [324]. The ‘lipidization’ of the hydrophilic drug by blocking hydrogen bond forming functional groups can increase the BBB permeability–surface area (PS) product, assuming the MW of the prodrug is <450 Da. However, lipidization also increases drug uptake by peripheral tissues, which reduces the plasma AUC of the drug. The increase in PS product is offset by the reduced plasma AUC, which results in only minor changes in the brain uptake of the drug, or % injected dose (ID)/g brain. The relationships between brain drug uptake, BBB PS product, and plasma AUC are given by Equation (1),

%ID/g = (BBB PS product) × (plasma AUC)(1)

Equation (1) is an approximation of Equation (7) in Section 11.4.4 (Methods), where the volume of distribution (VD) of the test drug is much greater than the brain plasma volume [1]. Equation (1) shows that drug lipidization that enhances BBB permeability, or PS product, does not translate to a parallel increase in brain uptake, or %ID/g, if there is a corresponding decrease in plasma AUC.

Xamoterol is a beta-1 adrenergic receptor agonist that is a potential treatment for AD [325]. Medicinal chemistry was used to replace a hydrogen bond forming amide functional group with a less polar ether group, and this xamoterol prodrug is designated STD-101-01 [325]. However, the oral bioavailability of the drug is low, which requires IV administration of the drug. The prodrug is rapidly removed from plasma, and the peak brain concentration of the prodrug in the rat is only 0.04%ID/g at 20 min after an IV injection of 10 mg/kg [325].

One of the few FDA-approved prodrugs for the CNS is dimethylfumarate for multiple sclerosis (MS) [326]. Monomethylfumarate activates the nuclear factor E2-related factor-2 pathway involved in oxidate stress [326]. Fumarate is a dicarboxylic acid, which do not cross the BBB [327]. Methyl esterification of both carboxyl groups reduces the hydrophilicity of the parent fumarate and enables BBB transfer. Other FDA-approved prodrugs, gabapentin enacarbil, and eslicarbazepine acetate [324], increase the oral bioavailability of drugs that already cross the BBB.

#### 6.1.4. Conjugation of Hydrophilic Drugs to Hydrophobic Carriers

A number of hydrophobic carriers have been used in an attempt to deliver hydrophilic drugs across the BBB. An early hydrophobic carrier was dihydropyridine (DHP) [328]. A hydrophilic drug, which did not cross the BBB, was conjugated to the DHP carrier. Once in brain, the DHP moiety was oxidized to a quaternary ammonium salt, which sequestered the conjugate in brain, since quaternary ammonium compounds do not cross the BBB. However, this approach does not block the hydrogen bond forming functional groups on the pharmaceutical agent, and BBB transport may not be enhanced by DHP conjugation [329]. The primary advantage of the DHP system is the sequestration in brain of a drug that is already hydrophobic such as estradiol (E2). The E2-DHP conjugate has a long residence time in brain compared to E2 alone [330]. However, the E2-DHP conjugate is highly hydrophobic and is administered IV in 100% dimethylsulfoxide (DMSO) [330]. The IV administration of 0.25 mL of 10–15% DMSO in the mouse causes BBBD to a 40 kDa protein, HRP [331]. Certain drug diluents, such as DMSO or sodium dodecylsulfate, may enable drug penetration through a BBB that is permeabilized by the detergent co-injected with the drug. Another problem with DHP conjugation is that the DHP modified drugs are labile, owing to oxidation [332].

Docosahexaenoic acid (DHA), a C22:6 essential free fatty acid (FFA), was proposed as a lipid carrier for brain drug delivery [57]. Interest in DHA as a lipid carrier was renewed by the finding that DHA is transported across the BBB via the major facilitator superfamily domain containing 2a (Mfsd2a) transporter [333], and DHA has been proposed as a ligand for the Mfsd2a-mediated transport of DHA-conjugated nanoparticles [334]. However, Mfsd2a does not transport unesterified DHA, but rather the lysolecithin form of esterified DHA [333]. Brain uptake of free DHA is not reduced in the Mfsd2a knockout mouse, and liver uptake of free DHA is 50-fold greater than the brain uptake of free DHA [333]. The brain uptake of DHA esterified as lysolecithin is nearly 10-fold greater than the unesterified form of DHA [333]. Nevertheless, the brain uptake of esterified DHA is still quite low, <<0.001%ID/g [333]. A contributing factor to the poor BBB transport of DHA is the avid binding of this FFA to albumin [335]. Owing to the very low BBB transport of DHA, it was necessary to employ BBB disruption by focused ultrasound to produce a significant brain level of LDL nanoparticles conjugated with DHA [336].

Other FFAs, such as the C18 unsaturated stearic acid, have been proposed as lipid carriers, even for large proteins such as the 40 kDa HRP [337]. HRP was conjugated with stearate and radio-iodinated by chloramine T [337]. Conjugation of a FFA to a protein such as HRP would not be expected to mediate free diffusion through the BBB, owing to the 450 Da MW threshold discussed in Section 6.1.2. Stearate conjugation of HRP had no effect on the brain uptake of the protein, measured as %ID/g, over a 3 h period after IV administration [337].

In summary, neither the use of medicinal chemistry to block polar functional groups on hydrophilic drug candidates, nor the conjugation of hydrophilic drugs to lipid carriers, has led to a significant number of new drug candidates for CNS disease that cross the BBB and can enter CNS clinical trials. An alternative approach, discussed in Section 6.2, uses medicinal chemistry to target endogenous carrier-mediated transporters (CMT) expressed at the BBB.

### 6.2. Carrier-Mediated Transport of Small Molecules

BBB carrier-mediated transporters (CMT) are members of the Solute Carrier (SLC) gene superfamily, which is the second largest gene family of membrane proteins behind G protein-coupled receptors. There are >400 genes, and >60 families of the SLC transporter gene super-family, often with extensive redundancy [338]. For example, there are >10 GLUT glucose transporters. Owing to the presence of multiple transporters for any given class of nutrients, it is necessary to confirm that the substrate transporter profile (STP) that is observed in vivo at the BBB is the same as the STP that is observed following in vitro expression of the SLC transporter that is said to function at the BBB [339]. This correlation of the in vivo/in vitro STP is especially crucial if BBB-penetrating small molecule drugs are developed to traverse the BBB via a specific CMT member of the SLC gene superfamily.

#### 6.2.1. GLUT1 Glucose Carrier

BBB glucose transport is stereospecific for D-glucose, and shows no affinity for L-glucose or fructose [48]. Multiple hexoses are transported via the BBB glucose transporter and the Km values for 2-deoxyglucose, D-glucose, 3-O-methylglucose (3OMG), D-mannose, and D-galactose, are 6 mM, 9 mM, 10 mM, 22 mM, and 42 mM, respectively [340]. The Vmax is constant for all hexoses, which means the Km is a true affinity constant for hexose binding to the carrier, and the rate-limiting step is glucose mobility through the transporter cavity [340]. The BBB glucose transporter is sodium-independent and is inhibited by phloretin, Ki = 16 µM, and phlorizin, Ki = 400 µM [340]. There are at least 14 different sodium independent glucose transporter (GLUT) genes, and at least 5 GLUT genes are expressed in brain including GLUT1, GLUT3, GLUT6, GLUT8, and GLUT13 [341]. The GLUT1 transporter (SLC2A1) is responsible for >95% of BBB glucose transport. This was demonstrated by showing the concentration of immunoreactive GLUT1 in a bovine brain capillary plasma membrane fraction, quantified with purified human erythrocyte GLUT1 as an assay standard, was identical to the concentration of D-glucose displaceable cytochalasin B binding sites in the brain capillary membrane fraction [342]. The equivalence of total glucose transporter sites and total GLUT1 sites at the BBB was confirmed with intact microvessels isolated from 70-day-old rabbits [343]. The concentrations of total D-glucose displaceable cytochalasin B binding sites, and total immunoreactive GLUT1, were 102 ± 25 pmol/mg protein and 111 ± 3 pmol/mg protein, respectively [343]. Cytochalasin B binds to all GLUTs, so the equivalence of the total GLUT1 and total glucose transporter levels at the brain capillary excludes a significant role for GLUTs other than GLUT1 as a BBB glucose carrier [342]. The concentration of GLUT1 mRNA at the brain capillary is at least a log order higher than the concentration of GLUT1 mRNA in the parenchyma of bovine brain [344], a finding confirmed with qPCR for rat brain [345].

**Figure 9:**
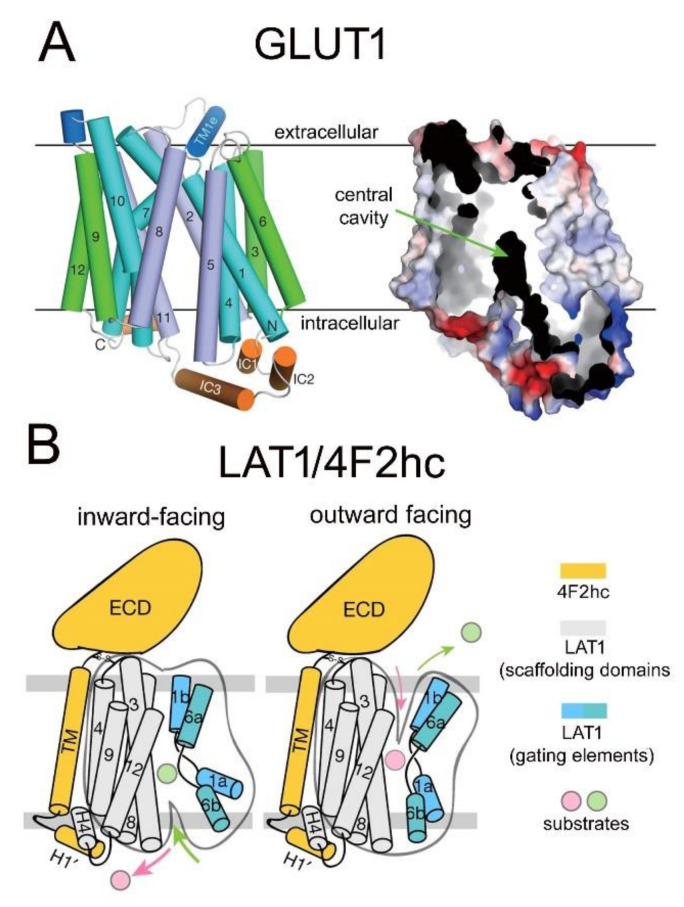
**Structure of GLUT1 and LAT1 carrier-mediated transporters.** (**A**) (Left) Model of crystal structure of human GLUT1 showing orientation of 12 transmembrane regions (TMR) in four 3-helical repeat domains composed of TMRs 1,4,7,10 (blue), TMRs 3,6,9,12 (green), and TMRs 2,5,8,11 (purple); the extracellular and intracellular helices are shown in dark blue and orange, respectively. (Right) Surface electrostatic potential model shows a central transporter cavity. Reproduced with permission from [346], Copyright© 2014 Springer-Nature. (**B**) Inward-facing and outward-facing models of the LAT1-4F2hc heteroduplex. LAT1 is composed of 12 TMRs, which form scaffolding and gating domains. The 4F2hc is formed by an extracellular domain (ECD), a transmembrane (TM) domain, which binds to TMR4 of LAT1, and an intracellular loop (H1′). Reproduced with permission from [348], Copyright© 2019 Springer-Nature.

The crystal structure of the human GLUT1 glucose transporter has been determined by x-ray crystallography [346]. The formation of GLUT1 crystals was facilitated by two point mutations: N45T and E329Q. The N45T mutation removes the single N-linked glycosylation site, and the E329Q mutation locked the transporter in an inward facing orientation [346]. The three-dimensional structure of the GLUT1 transporter shows the transporter protein forms a trans-membrane cavity that can be alternately accessed by the substrate from either side of the plasma membrane. The transporter cavity may exist in either an inward closed conformation, to mediate transport of D-glucose from blood or brain ECS to the intra-endothelial compartment, or may exist in an outward closed conformation, to mediate sugar transport from the intra-endothelial compartment to the plasma or brain ECS [346]. The GLUT1 carrier is composed of 12 transmembrane regions (TMR), which form a carboxyl terminal domain and an amino terminal domain. TMRs 1–6 form the amino terminal domain, and TMRs 7–12 form the carboxyl terminal domain TMR. The outward open conformation is largely coordinated by Aas in the carboxyl terminal domain (C), whereas the inward open conformation is largely determined by AAs in the amino terminal domain (N) [346]. The carboxyl terminal and amino terminal domains are connected by a short intracellular (IC) helical bundle. The structure of the GLUT1 transporter was also visualized by a surface electrostatic model [346]. This model reveals the central transporter cavity through which D-glucose, but not L-glucose, and certain other hexoses move to traverse the endothelial membrane. This transporter cavity is small, with dimensions of 0.8 × 1.5 nm [347], which is sufficiently large to accept D-glucose, which has a long axis of only 1 nm (10 angstroms). Therefore, as discussed below, it is dubious to expect that a conjugate of D-glucose and a drug, which does not cross the BBB, can be expected to move through the narrow, stereospecific gated cavity of the GLUT1 transporter. The TMR structure of the GLUT1 glucose transporter is shown in Figure 9A (left side), and the electrostatic model of the GLUT1 glucose transporter is shown in Figure 9A (right side).

Drugs that are designed to cross the BBB via the GLUT1 transporter, or for any BBB CMT system, fall into two categories: glucose-mimetic drugs or glucose-drug conjugates. A glucose-mimetic drug retains the basic structure of the D-glucose molecule, but certain substituents are added to the hexose that both (a) confer a pharmaceutical property on the glucose analogue, and (b) retain sufficient affinity for GLUT1 so that the glucose-mimetic drug traverses the BBB. Examples of glucose-mimetic drugs that are transported via GLUT1 include 2-deoxy [340] and 6-deoxy-6-chloro [349] analogues of D-glucose, both of which have a higher affinity for the BBB glucose carrier than D-glucose. The conjugation of a methylsulfonyl moiety to the 4-O and 6-O, but not the 3-O, hydroxyls was possible and a reasonable affinity of the glucose-mimetic for GLUT1 was retained [350].

The glucose-drug conjugate approach to brain drug delivery via GLUT1 involves the conjugation of a drug or peptide, which normally does not cross the BBB, to one of the hydroxyl groups on D-glucose. Glucose conjugates have been prepared for peptides and small molecules. Glucose was conjugated to the serine hydroxyl group of enkephalin peptides, and brain delivery of the hexa- or heptapeptide glucose conjugate was hypothesized to be mediated via GLUT1 at the BBB [351], although subsequent carotid arterial perfusion studies showed the conjugate had no affinity for GLUT1 [352]. Venlafaxine, which is a hydrophobic small molecule that crosses the BBB, was conjugated to D-glucose via an extensive linker, and this construct produced a new agent four times the size of D-glucose [353]. Studies demonstrating affinity of the conjugate for GLUT1 were not performed. A carboranylmethyl-glucose conjugate was synthesized to enable GLUT1 delivery of the boron agent for boron neutron capture therapy of cancer [354]. The borocaptate moiety conjugated to D-glucose was larger than the D-glucose molecule alone. Although the conjugate was taken up by a human cancer cell line in culture, no study showing this uptake was mediated via GLUT1 was performed [354].

#### 6.2.2. LAT1 Large Neutral Amino Acid Carrier

Large neutral amino acids (LNAA) traverse the BBB via a saturable transporter [48] that is characterized by high affinity (low Km) for the LNAAs ranging from a Km of 0.12 mM for L-phenylalanine to a Km of 0.73 mM for L-threonine [355]. These Km values approximate the LNAA plasma concentrations [356], which makes the brain selectively vulnerable to the high plasma concentrations of hyper-aminoacidemias, such as phenylketonuria, as discussed further in Section 11.4.1. The initial cloning of the major LNAA transporter, designated LAT1 (SLC7A5), from a C6 rat glioma line was enabled by the co-expression of 4F2hc (SLC3A2), which forms a hetero-duplex within the membrane with LAT1 [357]. The LNAA transporter at the BBB was cloned using frog oocyte expression of synthetic RNA produced from a bovine brain capillary cDNA library, and was shown to have an 89% amino acid identity with rat LAT1 [358]. Northern blotting showed the LAT1 mRNA was selectively expressed in brain capillary endothelial cells in vivo, and the LAT1 mRNA was at least 100-fold higher at the BBB as compared either to C6 rat glioma cells or to brain parenchyma [358]. The LAT1 mRNA was not detected in liver, heart, lung, or kidney [358]. Transport of tryptophan into oocytes expressing the cloned BBB LAT1 was characterized by high affinity with a Km of 32 µM [358], which correlates with the Km of tryptophan transport across the BBB in vivo [355].

The 3D structure of the human LAT1/4F2hc hetero-duplex has been determined by cryo-electron microscopy [348], and is depicted in Figure 9B. The 4F2hc protein is composed of an extracellular domain (ECD), a transmembrane (TM) domain and an intracellular H1′ loop (Figure 9B). The LAT1 protein is formed by 12 TMRs, and TMR4 of LAT1 has hydrophobic interactions with the TM domain of 4F2hc to stabilize the complex [348]. This interaction between LAT1 and 4F2hc occurs away from the transporter-gated cavity (Figure 9B). The narrow substrate cavity of LAT1 is adjacent to TMR1 and TMR6 (Figure 9B). BBB transport of LNAAs via LAT1 is sharply stereospecific for some amino acids [359]. The affinity of D-leucine is reduced about three-fold compared to L-leucine, but the affinity of D-tryptophan is >100-fold lower than the affinity of L-tryptophan, and the D-isomers of L-DOPA, isoleucine, valine, and threonine have no affinity for LAT1 [359]. The stereospecificity of BBB transport of LNAAs comports with the narrow-gated cavity through which amino acids traverse the membrane via LAT1 (Figure 9B).

There are major differences in the kinetics and transporter expression at the BBB for GLUT1 and LAT1 CMT systems. The Km and Vmax values for GLUT1 and LAT1 differ by more than two log orders of magnitude [339]. The brain capillary endothelial concentration (Ccap) of a BBB CMT system was first determined for GLUT1 using quantitative Western blotting, and cytochalasin B Scatchard plots, which showed the Ccap of GLUT1 was 100–110 pmol/mg protein [343]. Subsequently, the Ccap of GLUT1 was determined by quantitative targeted absolute proteomics (QTAP). In this approach, isolated brain capillaries were combined with liquid chromatography/mass spectrometry (LC-MS), along with sequence specific peptide standards, to measure the mass of GLUT1 at the brain capillary [360]. The concentration of GLUT1 in human brain capillaries (Ccap) is 139 ± 46 pmol/mg protein [360], which correlates with the Ccap of immunoreactive GLUT1 [343]. The QTAP technology has produced measurements of the Ccap for many different CMT, RMT, and AET systems, as discussed below. The Ccap values for LAT1, the cationic amino acid transporter 1 (CAT1), and the monocarboxylic acid transporter 1 (MCT1) have been measured by QTAP, and the Ccap of LAT1, CAT1, and MCT1 is much lower than the Ccap of GLUT1. The lower Ccap values for LAT1, CAT1, and MCT1, as compared to GLUT1, parallels the Vmax values of substrate transport through these CMT systems at the BBB in vivo [361]. The Vmax/Ccap ratio provides a measure of the transporter turnover rate, or number of substrate molecules transported per second at maximal velocity. The Km, Vmax, Ccap, and transporter turnover rate for GLUT1, MCT1, LAT1, and CAT1 are shown in Table 2.

The number of substrate molecules transported per second at maximal velocity, as determined from the Vmax/Ccap ratio [361], varies from 270–3000 substrates per second (Table 2). Thus, the transporter turnover rate can vary over a log order of magnitude, which explains why there is only an approximate correlation between the Vmax of the transporter in vivo, and the Ccap as measured by QTAP (Table 2).

There are a number of FDA-approved CNS drugs that penetrate the BBB via LAT1, although in all cases, this was a serendipitous finding. The first LAT1 drug developed was L-dihydroxyphenylalanine (L-DOPA) for PD, which was approved in 1970. L-DOPA crosses the BBB via LAT1 and is converted in brain to dopamine via the action of aromatic amino acid decarboxylase (AAAD). In 1959, Holtz [362] reviewed the conversion of L-DOPA into dopamine by AAAD. In 1963, Yoshida et al. [363] reported experiments showing the uptake of L-DOPA by brain slices was inhibited by LNAAs, but not by a small neutral amino acid, alanine, or an acidic amino acid, glutamate. In 1966, Hornykiewicz [45] reviewed the low production of dopamine in the striatum of PD, and the ability of L-DOPA administration to increase brain dopamine, but did not mention either the BBB or how L-DOPA gains access to the CNS. In 1975, Wade and Katzman [45], using the Oldendorf BUI technique [46], demonstrated L-DOPA crosses the BBB on an amino acid transport system, and in 2000, Kageyama et al. [364] showed that L-DOPA is a substrate for LAT1. Phenylalanine mustard (melphalan), a chemotherapeutic alkylating agent, was shown to be therapeutic in mice with experimental brain cancer [365], and subsequent arterial infusion experiments demonstrated that melphalan crossed the BBB via the LNAA transporter [366]. Melphalan was subsequently shown to be a ligand for LAT1 [367]. Gabapentin, a γ-amino acid, was developed as a new anti-convulsant in the 1990s, and cerebral microdialysis showed gabapentin crossed the BBB, although no mention was made as to mechanism of transport [368]. Using the frog oocyte expression system, the LAT1-mediated uptake of [^14^C]-phenylalanine was blocked by amino acid-like drugs, and the Ki for melphalan, L-DOPA, and gabapentin was 49 µM, 67 µM, and 340 µM, respectively [367]. The transport of gabapentin, a γ-amino acid, by LAT1 is unexpected, since LAT1 transports α-amino acids, not γ-amino acids. However, gabapentin is a cyclic compound wherein the amino and carboxyl moieties sterically resemble an α-amino acid. A perplexing example of a drug that is said to be transported by a LNAA transporter is paraquat, which is a quaternary ammonium salt, and such molecules do not cross the BBB. Paraquat is a widely used herbicide, and there is inconclusive evidence that paraquat neurotoxicity can be associated with PD [369]. Paraquat is structurally similar to the neurotoxin, 1-methyl-4-phenylpyridinium (MPP+), and MPP+ does not cross the BBB [370]. However, the SQ administration of 5–10 mg/kg paraquat results in drug distribution to brain via a process that is inhibited by a LNAA, L-valine, but not by a cationic amino acid, L-lysine [370]. Paraquat transport via LAT1 has not been tested. While the cyclic structure of gabapentin provides an explanation for gabapentin transport via LAT1, the structure of paraquat has no resemblance to an α-amino acid, and paraquat transport at the BBB may be mediated by a transporter other than LAT1.

The development of drugs that mimic the structure of a LNAA, and which are transported via LAT1, is the most advanced area of CNS drug development that targets a BBB CMT system. This work has evolved in two parallel pathways. First, LAT1 structure-based ligand discovery was initiated following the stable transfection of HEK293 cells with the full length human LAT1 cDNA, followed by screening drugs that inhibit the uptake of [^3^H]-gabapentin [371]. Subsequent to the elucidation of the 3D structure of the human LAT1/4F2hc heterocomplex [348,372], this structural information was used to assess the docking of LNAA-type drugs into the LAT1 transporter cavity [373]. To be effective in the CNS, a LNAA-type drug must not only bind to LAT1, but also undergo translocation through the membrane [373]. The second approach to the development of LNAA-type drugs that cross the BBB via LAT1 is executed without knowledge of the LAT1 binding site. Modifications to the LNAA structure are made and the affinity of the drug for LAT1 is then determined. An early example of the types of modifications to a neutral amino acid drug that can be made, and still retain LAT1 affinity, was the synthesis of 6-mercaptopurine-L-cysteine [374]. L-cysteine is a small neutral amino acid, which has a low affinity for the BBB LNAA transporter [48]. However, conversion of the free sulfhydryl group on L-cysteine to a disulfide linked therapeutic group converts L-cysteine to a LNAA, which has appreciable affinity for the BBB LNAA transporter in vivo [374]. An alternative approach to the use of medicinal chemistry to generate CNS drugs that penetrate the BBB via transport on LAT1 is the coupling of a pharmaceutical agent, ketoprofen, to the phenolic para-hydroxyl of L-tyrosine to form an ester compound [375]. The tyrosine-ketoprofen traversed the BBB in vivo via the LNAA transporter [375]. Subsequent work showed that higher affinity for LAT1 was achieved if the drug was linked to the meta position of the benzene ring of L-phenylalanine [376,377,378].

#### 6.2.3. CAT1 Cationic Amino Acid Carrier

BBB transport of the cationic amino acids (arginine, lysine, ornithine) is mediated by a saturable carrier [48] with high affinity and Km values ranging from 90 µM to 230 µM [355]. The original Rec-1 locus, which is a murine ecotropic retrovirus receptor, was shown to be a mammalian cationic amino acid transporter [379], now named CAT-1 (SLC7A1). The rat or mouse brain Rec-1 cDNA was cloned by reverse transcription of brain-derived RNA using oligodeoxynucleotides derived from the Rec-1 gene, and an RNase protection assay was used to demonstrate expression of the mRNA for CAT1 in isolated brain microvessels [380]. The crystal structure of mammalian CAT1 has not been reported, but hydropathy plots predict 14 transmembrane regions [381]. A novel use of medicinal chemistry to develop a BBB penetrating prodrug involved the conjugation of the carboxylic acid group of ketoprofen to the ε-amino acid moiety of lysine [382]. This converted the lysine into a large neutral amino acid, and the ketoprofen-lysine conjugate was transported through the BBB by LAT1 [382].

#### 6.2.4. MCT1 Monocarboxylic Acid Carrier

Monocarboxylic acids (MCA), such as pyruvate, lactate, the ketone bodies (β-hydroxybutryate and acetoacetate), are transported across the BBB by a specific MCA carrier [327,383]. The initial MCA carrier was cloned in 1994 and designated MCT1 (SLC16A1) [384]. The MCT1 mRNA was detected by PCR in rat brain microvessels [385], and MCT1 was localized to brain microvessels by immunohistochemistry [386]. MCT1 exists in the membrane as a hetero-duplex with the 60 kDa basigin protein (Bsg, CD147) [387], similar to the LAT1/4F2hc hetero-duplex (Figure 9B). The 3D structure of the MCT1-Bsg complex has recently been elucidated by cryoelectron microscopy [388]. The Bsg protein is formed by an ECD, which is composed of 2 immunoglobulin-like domains, a transmembrane (TM) domain, and a short intracellular loop. The MCT1 is composed of 12 transmembrane regions (TMR) with 6 TMRs forming the amino terminal domain (NTD) of MCT1, and 6 TMRs forming the carboxyl terminal domain (CTD) of MCT1 [388]. The TM domain of Bsg stabilizes the NTD of MCT1. Substrate translocation is proton dependent and rotation of the NTD and CTD expose the substrate binding site on each side of the membrane [388]. A prodrug transported by MCT1 was formed with an amide linkage between the ring nitrogen of 5-fluorouracil (5FU) and the carboxylic acid group of either adipic acid or suberic acid [389].

#### 6.2.5. CNT2 Purine Nucleoside Carrier and Adenine Carrier

Purine nucleosides (adenosine, guanosine), but not pyrimidine nucleosides (uridine, thymidine), traverse the BBB by a saturable carrier that is distinct from a nucleobase carrier [390]. BBB transport of adenosine is sodium-dependent and is not inhibited by nitrobenzylthioinosine (NBTI) [391]. The BBB adenosine carrier is characterized by a Km of 25 ± 3 μM and a Vmax 0.75 ± 0.08 nmol/min/g [361], which is 100-fold lower than the Vmax of MCT1 (Table 2). The substrate transporter profile (STP) of the BBB adenosine transporter in vivo, e.g., lack of affinity for pyrimidine nucleosides and sodium-dependency, is consistent with the STP of the concentrative nucleoside transporter (CNT)2 or SLC28A1 [392]. The molecular identity of the BBB adenosine transporter has been ascribed to the equilibrative nucleoside transporter (ENT)2 or SLC29A2 [393], because the abundance of CNT2 is below the limit of quantitation (LOQ) using QTAP methodology and human CMEC/D3 cultured endothelium [394]. However, the abundance of LAT1 is also below the LOQ in this cell line [394]. A low abundance of the nucleoside transporter at the BBB is expected given the 100-fold lower Vmax of the adenosine transporter compared to the MCT1 transporter [361]. Molecular cloning of the BBB adenosine transporter was performed with the frog oocyte system following oocyte injection of cloned RNA derived from a rat brain capillary cDNA library [395]. A clone was identified and DNA sequencing showed the BBB adenosine transporter was CNT2 [395]. The identification of the BBB adenosine transporter as CNT2 was consistent with the known properties of BBB adenosine transport, e.g., sodium dependency and NBTI insensitivity. The Km of adenosine transport into the oocytes expressing CNT2, 23 ± 4 µM [395], is identical to the Km of BBB transport of adenosine in vivo [361]. Adenosine transport into the oocytes was inhibited by adenosine, guanosine, and uridine, but not by cytidine or thymidine [361], and this STP of the cloned CNT2 parallels the STP of purine nucleoside transport across the BBB in vivo [390]. The sodium concentration required to produce maximal transport via the adenosine transporter expressed in frog oocytes, i.e., the K50, is 2.4 ± 0.1 mM, and the Hill coefficient is 1, indicating adenosine and sodium are co-transported in a 1:1 ratio via CNT2 [395].

The 3D structure of CNT2 has not yet been elucidated, but the structure of CNT3 has been reported using cryo-electron microscopy [396]. CNT3 has a high degree of sequence homology with CNT2 [396], although the sodium Hill coefficient for CNT2 is 2 [392]. CNT3 exists in the membrane as a homo-trimer [396]. If CNT2 also exists within the membrane as a homo-trimeric structure, this could explain the asymmetry of nucleoside transport via the BBB CNT2 expressed in frog oocytes [397]. Adenosine is transported via the BBB CNT2 on a high Vmax site, whereas dideoxyinosine (DDI) and thymidine are transported on a second low Vmax site on the CNT2 transporter [397].

The availability of the 3D structure of CNT3 enables the rational design of adenosine-based drugs that cross the BBB. One drug that may cross the BBB via CNT2 transport is cladribine, an immune-suppressive used in the treatment of multiple sclerosis [398]. Cladribine is a form of deoxy-chloro adenosine, and is transported by CNT2 [398].

An important consideration in the design of adenosine-based drugs that cross the BBB via CNT2 is the enzymatic barrier to adenosine. Although topical application of adenosine to pial vessels causes enhanced brain blood flow, the intra-arterial infusion of adenosine in dogs does not increase cerebral blood flow [399]. The lack of a pharmacologic effect of arterial administration of adenosine is due to an enzymatic BBB to adenosine transport. [^3^H]-adenosine was administered by internal carotid artery infusion for 15 s followed by microwave irradiation of the brain to cause immediate cessation of brain metabolism [391]. After 15 s of infusion, only 10 ± 3% of brain radioactivity resided in the unmetabolized adenosine pool, with 34% of radioactivity in non-adenosine nucleosides (inosine, hypoxanthine), and 32% in various nucleotide pools [391]. Therefore, adenosine-based drugs that cross the BBB must be designed not only for CNT2 affinity, but also must be resistant to the enzymatic BBB to adenosine.

In addition to the CNT2 purine nucleoside transporter, purine bases, particularly adenine, traverse the BBB via a saturable carrier that is distinct from the adenosine carrier [390]. To date, no nucleobase transporter (NBT) has been identified at the BBB.

#### 6.2.6. CTL1 Choline Carrier

BBB transport of choline, a quaternary ammonium compound, was measured with the BUI method, and found to be saturable consistent with a carrier-mediated mechanism [400]. BBB transport is inhibited by hemicholinium (HC)-3, also a quaternary ammonium molecule, and by 2-(dimethylamino) ethanol (deanol), which is a tertiary amine compound [400]. The BBB choline carrier was subsequently examined with the internal carotid artery perfusion method, and these studies reported a choline transport Km of 40 µM and a Vmax of 2.7 nmol/min/g [401]. The HC-3 Ki was 57 ± 11 µM [401]. The BBB choline carrier tolerates a number of substitutions on the choline nucleus as N-n-octylcholine and N-n-octylnicotinium both inhibit choline transport, although no inhibition is observed for N-methylpyridinium [402]. One caveat is that a given molecule may inhibit a CMT transporter, but not actually be transported by the CMT system. The BBB choline carrier could be used as a brain drug delivery system [403], and early 3D-quantitative structure activity relationships (3D-QSAR) were initiated [404]. Different 3D-QSAR models were developed to predict drugs that cross the BBB on the choline carrier [405,406]. Such models would be aided by knowledge on the molecular properties of the BBB choline carrier. Choline transporters include the choline high affinity transporter (CHT)-1, which is a member of the sodium dependent glucose transporter gene family, and is designated SLC5A7. However, CHT1 can be excluded as the BBB choline transporter, as BBB choline transport in vivo is not high affinity [400,401]. Choline transporter-like (CTL) protein-1 (SLC44A1) and CTL2 (SLC44A2) exhibit transport properties consistent with BBB choline transport in vivo, e.g., choline Km = 10–200 µM, HC-3 Ki = 10–100 µM [407]. The mRNA encoding both CTL1 and CTL2 are detected in cultured human brain endothelium [408]. However, the abundance of both CTL1 and CTL2 is <LOQ in QTAP studies of rat brain capillaries [409], similar to other CMT systems at the BBB with a low Vmax. More definitive evidence is needed that CTL1 or CTL2 mediates transport at the BBB of choline and choline-like drugs.

#### 6.2.7. Vitamin Carriers

The transport of the B vitamins across the BBB is carrier-mediated via members of the SLC transporter family. BBB transport of vitamin B1 (thiamine) is saturable [410], and the thiamine transporter (THTR)2 (SLC19A3) is expressed in brain [411]. Thiamine deficiency leads to CNS morbidity [412]. Transport of vitamin B2 (riboflavin) is saturable at the BBB in vivo [413] and in cultured endothelium [414]. The riboflavin vitamin transporter (RFVT)2 is expressed in brain, and is SLC52A2. Mutations in either RFVT2 (SLC52A2) or RFVT3 (SLC52A3) lead to neurodegeneration [415]. Vitamin B3 (niacin, nicotinic acid) is a monocarboxylic acid transported by MCT1 [416]. However, niacin is amidated to form niacinamide, which is the major form of vitamin B3 in plasma, and niacinamide traverses the BBB by non-saturable free diffusion [417]. Vitamin B4 refers alternatively to choline, adenine, or carnitine, which are no longer considered vitamins, although adenine and choline are essential nutrients, and carnitine is a conditionally essential nutrient. Vitamin B5 (pantothenic acid) traverses the BBB via a saturable process with a Km of 19 μM [418]. Both pantothenic acid and biotin (vitamin B7 or B8) are monocarboxylic acids which are transported via the sodium dependent multivitamin transporter (SMVT, SLC5A6) [419], as listed in Table 3.

SMVT is expressed in brain capillary endothelium [420]. Brain biotin uptake is saturable [421], and the brain uptake of biotin in the rat is 0.28 ± 0.03%ID/g [422]. Biotin may be transported either by MCT1 or SMVT [423]. Vitamin B6 (pyridoxine) is a small molecule with a MW of 169 Da which forms six hydrogen bonds with water, and should traverse the BBB via free diffusion. However, the brain uptake of pyridoxine is saturable [424], and transfection of cells encoding the THTR thiamine transporter (SLC19A3) leads to increased pyridoxine uptake [425]. Vitamin B9/B11 (folic acid) is transported by the folate receptor (FOLR)1, the reduced folate carrier (RFC, SLC19A1), and the intracellular proton-coupled folate transporter (PCFT, SLC46A1) [426]. The active metabolite of folic acid (FA) is 5′-methylenetetrahydrofolic acid (MTFA). The RFC has a higher affinity for MTFA than for FA, whereas the affinity of FOLR1 for MTFA and FA is comparable. The BBB transport of MTFA was equally inhibited by FA and MTFA, which suggests the major BBB folate transporter is FOLR1 [427]. Folate delivery to brain is suppressed in the FOLR1 knockout mouse [426]. However, the mRNA level of RFC exceeds the level for FOLR1 mRNA in isolated brain microvessels, which points to an important role for the RFC in BBB transport of folic acid [426]. Vitamin B10 is p-aminobenzoic acid, which is no longer considered a vitamin. Vitamin B12 (cobalamin) is transported in blood bound to the transcobalamin (TC) binding protein [428]. The B12/TC complex is endocytosed into cells via the TC receptor (TCblR), also known as CD320, and the three-dimensional structure of the B12/TCblR complex has been determined [428]. Knockout of the CD320 gene in the mouse is not lethal, although the brain concentration of B12 is >90% reduced, and metabolites associated with vitamin B12 deficiency are selectively increased in brain in the CD320 knockout mouse [429]. Expression of the TCblR/CD320 at the BBB has been confirmed [430].

The vitamin transporters are potential conduits for drug delivery to brain. In an effort to deliver neuropeptide YY to brain, this peptide was conjugated to vitamin B12 [431]. An ampakine compound was conjugated to thiamine via a disulfide bridge, which resulted in increased brain uptake of the ampakine [432].

All of the SLC transporters for nutrients or vitamins described in Section 6.2.1, Section 6.2.2, Section 6.2.3, Section 6.2.4, Section 6.2.5, Section 6.2.6 and Section 6.2.7 are potential conduits to brain of drugs that mimic the structure of the nutrient or vitamin transported by the respective SLC transporter. These numerous SLC transporters recognize a broad universe of molecular structures that can guide the medicinal chemist in creating nutrient-mimetic or vitamin-mimetic pharmaceuticals that cross the BBB via CMT.

#### 6.2.8. Thyroid Hormone Carriers

The saturable transport of the thyroid hormones, L-triiodothyronine (T3) and L-thyroxine (T4), across the BBB in vivo in the rat was demonstrated with the BUI method in 1979 [433]. The Km of T3 transport was 1.1 µM and the Ki of T4 inhibition of T3 transport was 2.6 uM. T3 transport was not inhibited by high concentrations of LNAAs, leucine or tyrosine. The Vmax of T3 transport was 0.2 nmol/min/g [433], which is 100-fold lower than the Vmax of transport via LAT1 (Table 2). Subsequently, MCT8 (SLC16A2) was shown to transport both T3 and T4 to a comparable degree [434]. The low Vmax of BBB T3 transport is consistent with the inability to detect MCT8 in brain endothelial cells by QTAP proteomics method [435], but immunohistochemistry with an antibody against MCT8 illuminated microvessels in human, rat, and mouse brain similarly to the immune staining obtained with an anti-Pgp antibody [436]. The knockout of the MCT8 gene in the mouse does not result in CNS impairment or CNS hypo-thyroidism [437], which suggests the mouse has an alternative pathway for thyroid hormone transport across the BBB, as discussed below. However, mutations in the MCT8 gene in humans causes impaired neurodevelopment, a condition known as the Allan–Herndon–Dudley syndrome [437], which indicates humans may not have an active alternative to the MCT8 pathway of thyroid hormone transport across the BBB, as discussed below.

A second thyroid hormone transporter is organic anion-transporting polypeptide (Oatp)1c1, also known as oatp14, and now designated Slco1c1. This gene was originally cloned as part of a BBB genomics investigation [438]. The new gene was named BBB-specific anion transporter 1 (BSAT1) because of a distant sequence homology with a liver specific anion transporter. The BSAT1 mRNA was not detected by Northern blotting in rat heart, lung, liver, kidney, or total brain, but was highly expressed in isolated rat brain capillaries [438]. The sequence of the full length 2736 nucleotide cDNA of rat BSAT1 was deposited in GenBank in 2001 (AF306546), and this sequence encoded for a 716-amino acid protein. Expression of the mouse oatp14 cDNA in HEK293 cells showed this transporter mediated uptake of the estradiol β-glucuronide (E_2_G) anion, but also mediated the uptake of T4 and T3 [439]. Expression of the rat BSAT1/oatp14/Slco1c1 in HEK293 cells showed the Km of transport of T4 and E_2_G via BSAT1 was 0.72 ± 0.10 µM and 6.1 ± 0.5 uM, respectively [440]. T3 inhibited the transport of T4 and E_2_G with a Ki of 50 ± 17 μM and 4.2 ± 0.7 µM, respectively. Transport of T4, T3, and E_2_G via BSAT1 (Slco1c1) was asymmetric and consistent with transport via two sites [440]. Site 1 transported T4, but not T3 or E_2_G, and site 2 transported T4, T3, and E_2_G. Using a prealbumin trap technique, the efflux of intracellular T4 was enhanced by the presence of E_2_G in the extracellular compartment [440]. Prealbumin binds T4 with high affinity and prevented reuptake of T4 following efflux from the preloaded cell [440]. Immunohistochemistry with an antibody against Slco1c1 illuminated the microvessels in rat and mouse brain, but not in human brain [436]. The absence of expression of immunoreactive Slco1c1 in microvessels of human brain was confirmed by qPCR analysis measuring the Slco1c1 mRNA in total brain and brain microvessels. The Slco1c1 mRNA was highly enriched at the brain microvessel compared to total brain for rat and mouse, but there was no enrichment of the Slco1c1 mRNA in human microvessels [436]. Similarly, the Slco1c1 gene was repeatedly isolated in rat brain vascular genomic studies [438,441], but was not detected in a similar genomics investigation using microvessels isolated from fresh human brain obtained at neurosurgery [442]. These species difference in Slco1c1 expression at the BBB in rodents vs. humans suggest that Slco1c1 may not be a suitable target for brain drug delivery in humans. The high expression of Slco1c1 at the rodent BBB, but not the human BBB, explains why MCT8 mutations cause cerebral hypo-thyroidism in humans, but not in mice [437].

#### 6.2.9. Organic Cation Carrier

Carnitine is essential to brain metabolism as a mediator of free fatty acid delivery to mitochondria [443]. Carnitine is an amino acid betaine with a quaternary ammonium terminus. Carnitine is transported via the organic cation (OCTN)2 transporter (SLC22A5) [443]. The transport of carnitine across the BBB in vivo is very low and comparable to sucrose [444], although OCTN2 mediates carnitine uptake in human brain endothelium in cell culture [445]. The SLC22 gene family includes both the organic cation transporter (OCT) and OCTN organic cation transporters as well as the organic anion transporters (OAT) [446]. A recent proteomics study of OCT expression at the human brain microvessel showed that OCT-3 (SLC22A3) is the most abundant OCT transporter at the human BBB, with an expression level of 0.15 pmol/mg capillary protein [447], a level that is about one-third the expression of LAT1 (Table 2). OCT-1 (SLC22A1) and OCT-2 (SLC22A2) were not detectable at the human BBB [447]. HEK293 cells transfected with human OCT-3 were used in a high throughput screen of over 2000 compounds that are potential OCT-3 substrates by measuring the inhibition of the cell uptake of a model OCT-3 fluorescent substrate, 4-(4-(dimethylamino)styryl)-N-methylpyridinium iodide (pinaflavol), which is a quaternary ammonium compound [447]. The investment of such a significant effort to find drugs that penetrate the BBB via transport on OCT-3 assumes that this transporter mediates the influx of drugs from blood to brain. This may not be the case as the striatal neurotoxin, MPP+, is transported via OCT-3 [448], but MPP+ does not cross the BBB [370].

In summary, multiple CMT systems are expressed at the BBB that mediate the transport of nutrients, vitamins, thyroid hormones, and organic cations from blood to brain. The experiments demonstrating saturable BBB transport of nutrients were conducted largely in the 1970s using the BUI method [48,327,340,355,383,390,400,433]. Since then, BBB transporters are now classified on a molecular basis within the context of the SLC gene superfamily. The molecular biology of the BBB CMT systems is now complex as there are >400 members of the SLC transporter family. Therefore, it is crucial to show that the substrate transporter profile (STP) of the cloned SLC transporter mirrors the STP observed at the BBB with in vivo transport investigations. The expanded knowledge base of BBB transport via SLC carriers provides targets for solving current day brain drug delivery problems for small molecules. Solutions to the problem of BBB delivery of hydrophilic small molecules has, in the past, focused on the use of medicinal chemistry for the conversion of hydrophilic small molecules into lipid-soluble prodrugs. However, this had led to few FDA-approved drugs for the CNS, as reviewed above in Section 6.1. Instead, CNS drug developers can elucidate the STP of the individual BBB CMT systems both in vivo and with cloned transporters, and then use medicinal chemistry to convert hydrophilic small molecules into drugs that mimic the structure of endogenous ligands transported by the BBB CMT systems. Such work is ongoing in academic labs, particularly for LAT1, as reviewed above in Section 6.2.2. However, the pharmaceutical industry has yet to adopt this approach, as the industry continues to focus on the development of lipid-soluble small molecules that treat primarily only psychiatric disorders and epilepsy, as reviewed in Section 6.1.1

### 6.3. Active Efflux Transport of Small Molecules

#### 6.3.1. Brain-to-Blood Efflux

The carrier-mediated SLC transporters reviewed in Section 6.2 enable the influx from blood to brain of specific classes of nutrients or vitamins. Brain-to-blood efflux across the BBB also takes place for excitatory neurotransmitters, such as the acidic amino acids, *L*-glutamate and *L*-aspartate, and for neurotransmitter metabolites, such as homovanillic acid (HVA), which is derived from catecholamine degradation. These molecules are polar and require access to specific efflux transporters in order to undergo exodus from brain-to-blood. Drugs may also be recognized by the endogenous BBB efflux transporters, which would adversely affect drug distribution to brain. An early study of drug efflux across the BBB was performed with the BUI technique, which showed the BBB permeability of valproic acid (VPA) in the brain-to-blood direction was several-fold greater than in the blood-to-brain direction [449]. However, the saturability, or cross-competition of drug efflux, cannot be accessed with the BUI method. Terasaki and colleagues developed the Brain Efflux Index (BEI) method for the study of solute efflux from brain following the direct intra-cerebral injection under stereotaxic guidance [450]. While the BEI method is generally used to study the efflux of small molecules, this method can also be used to examine the brain-to-blood transport of large molecules, such as IgGs or transferrin [451,452]. The BEI method is particularly useful to assess the BBB efflux of drugs. The brain efflux of two drugs used for HIV infection, azidothymidine (AZT) and dideoxyinosine (DDI), was measured with the BEI method [453]. Both drugs effluxed from brain with a T_1/2_ of 22–28 min, and AZT and DDI efflux was inhibited by organic anions, probenecid and p-aminohippuric acid (PAH). Other organic anions, such as the bile salt, taurocholate (TC), were demonstrated to undergo efflux across the BBB, and the TC efflux was inhibited by cholic acid and probenecid, but not by PAH [454]. Acidic amino acids, glutamate and aspartate, have the lowest rate of influx from blood to brain of any of the amino acids [455]. The high rate of efflux of the acidic amino acid from brain to blood was demonstrated with the BEI method [456]. Similarly, the BEI method characterized the efflux from brain of endogenous organic anions, such as estrone 3-sulfate (E3S), as well as the neutral estrogen, estrone (E1). The T_1/2_ of efflux of either E3S or E1 from brain was about 10 min [457]. The efflux of E3S across the BBB via free diffusion is nil, owing to the highly polar sulfate group. Therefore, efflux of the E3S would require access to a transporter, and this was demonstrated by the inhibition of E3S efflux by another endogenous organic anion, dehydroepiandrosterone sulfate (DHEAS) [457]. E1 is a hydrophobic estrogen, and such unconjugated estrogens rapidly cross the BBB via free diffusion [322], but are reversibly sequestered in brain owing to binding to cytoplasmic proteins [458]. In the absence of this sequestration, E1 should efflux from brain with a T_1/2_ comparable to water, which is 1.1 min [450]. However, the cytoplasmic binding of E1 in brain results in the prolonged brain residence time [457], as discussed further in Section 11.5.2. The brain efflux and influx of DHEAS was assessed with the BEI and internal carotid artery perfusion (ICAP) methods, respectively [459]. The rate of efflux of DHEAS was more than 10-fold faster than the rate of influx. The influx from blood to brain was restricted by the polar sulfate moiety of DHEAS, as the sulfate group converted the DHEA steroid to an organic anion and a substrate for organic anion transporters. The carrier-mediated efflux of DHEAS from brain to blood was inhibited by other organic anions, TC and E3S [459]. An organic anion generated in the degradation of catecholamines is HVA, and HVA efflux from brain to blood is inhibited by other organic anions, probenecid and PAH [460]. Anticonvulsants may undergo active efflux from brain to blood, as demonstrated for phenytoin [461]. Frog oocyte expression studies implicated MCT8 as the principal efflux transporter at the BBB for phenytoin [461]. Active efflux transporters play an important role in the distribution to brain of anticonvulsants [462].

The assignment of nutrient or vitamin CMT systems to specific members of the SLC gene superfamily is discussed above in Section 6.2. The comparable assignment of the BBB efflux transporters to specific transporter genes is more difficult owing to the large number of transporter candidates. Active efflux transporters (AET) at the BBB may arise from either the ATP-binding cassette (ABC) gene superfamily or the SLC gene superfamily. The SLC gene family includes nearly 460 genes divided over 65 sub-families [463]. The ABC gene family is composed of nearly 50 genes divided over 7 sub-families [464,465].

#### 6.3.2. ABC Efflux Transporters

ABCA1 and ABCG1 are cholesterol transporters, which mediate the efflux of cholesterol metabolites from brain to blood. Astrocytes and neurons synthesize cholesterol de novo [466]. Excess cholesterol is removed from brain by hydroxylation of cholesterol to form 24(*S*)hydroxycholesterol (24S-HC) [467], and 24S-HC is exported to blood via transport on ABCA1 and ABCG1 [466]. Brain capillary proteomics shows that ABCA1 is primarily expressed on the abluminal endothelial membrane [468]. The loss of ABCG1 leads to a toxic accumulation of 24S-HC and other oxysterols in brain [469]. All of cholesterol in blood is bound to lipoproteins, and lipoprotein-bound cholesterol does not cross the BBB [466]. The BBB transport of free cholesterol in the blood-to-brain direction was measured with the internal carotid artery perfusion (ICAP) method. BBB transport of free cholesterol was rapid, and the BBB PS product was 0.64 mL/min/g in the wild-type mouse and 1.3 mL/min/g in the abca1 knockout mouse [470]. However, these studies are difficult to interpret, because free cholesterol does not exist in plasma. The BUI of free [^3^H]-cholesterol following the carotid artery injection in either saline or serum is high 63 ± 8% [471]. However, simply mixing cholesterol with serum does not lead to incorporation of cholesterol into lipoproteins, unless the serum is incubated overnight at 37C [471]. When this is performed, the BUI of [^3^H]-cholesterol in human serum is at the background level of brain uptake [471]. The absence of transport of lipoprotein bound cholesterol from blood to brain is consistent with the absence of expression of the low-density lipoprotein receptor (LDLR) at the BBB, as discussed below in Section 8.1.6.

ABCB1, also known as p-glycoprotein (Pgp), or the multi-drug resistance (MDR) gene product, was shown, in 1989, to be expressed at the BBB with immunohistochemistry of human brain and antibodies specific for human Pgp, although no PgG was detected at the epithelium of the choroid plexus [62]. A Pgp knockout mouse was developed in 1993 [472]. Quinidine is a lipid-soluble small molecule with a MW of 324 Da, and should cross the BBB. However, quinidine is a substrate of Pgp. The brain uptake of quinidine was increased nearly 30-fold in the Pgp knockout mouse [473]. Verapamil is a lipid-soluble small molecule that should cross the BBB, but is a substrate of Pgp. Brain uptake of [^11^C]-verapamil in the rat was measured by PET, and brain uptake was increased by the co-administration of cyclosporine A (CsA), a Pgp modulator [474]. CsA has a MW of 1203 Da, and has minimal BBB transport [475]. The effect of CsA on Pgp-mediated transport suggests the Pgp is expressed at the luminal membrane of the endothelium. Proteomics studies of brain capillaries show that Pgp is exclusively expressed at the luminal endothelial membrane [468].

A total of 42 small molecules were examined for Pgp regulated brain uptake [476]. This group was composed of both “CNS drugs”, which had a mean MW of 297 Da and a mean polar surface area of 48 Å^2^, and “non-CNS drugs”, which had a mean MW of 468 Da, and a mean polar surface area of 80 Å^2^. The brain:plasma ratio of Pgp ligands, such as metoclopramide and risperidone, in the Pgp knockout mouse relative to the brain:plasma ratio in the wild-type mouse, was 7–10-fold [476]. No change in the CSF:plasma ratio was observed [476], which is consistent with the lack of Pgp expression at the choroid plexus, as originally reported in 1989 [62]. A similar finding of lack of Pgp expression at the choroid plexus was made in the primate for the HIV protease inhibitor, nelfinavir, which is a substrate for Pgp. The co-administration of nelfinavir and a Pgp-inhibitor, zosuquidar, resulted in an increase in uptake of nelfinavir into brain, but not into CSF [477]. The absence of immunoreactive Pgp at the choroid plexus has been confirmed in the rat [478] and human [479]. Brain capillary proteomics shows the level of Pgp at the brain capillary is 6.7 pmol/mg protein, which is 45-fold higher than the Pgp level at the choroid plexus, 0.15 pmol/mg protein, which is near the limit of quantitation [480]. Pgp expression in brain is generally believed to be confined to the vasculature. However, immunoreactive Pgp is expressed on astrocyte foot processes in the brain of humans [481] and primates [482].

The multi-drug resistance-associated proteins (MRP)-1 to MRP-6 are encoded by the ABCC1-ABCC6 genes. MRP1 is expressed at both the BBB and at the choroid plexus [478]. Confocal microscopy of brain shows MRP1 and MRP5 are primarily expressed at the abluminal endothelial membrane, whereas MRP4 is primarily expressed at the luminal membrane [345]. Of the MRPs, the mRNA encoding MRP6 is the most highly enriched at the microvasculature of human brain [483].

The breast cancer resistance protein (BCRP) is encoded by the ABCG2 gene, and is an important efflux transporter at the BBB [484]. Confocal microscopy of human brain and glioma shows co-localization of BCRP with GLUT1 [485]. Brain vascular proteomics shows high expression of BCRP at the BBB across multiple species [360,435,480,486]. BCRP is 7-fold enriched at the luminal capillary endothelial membrane as compared to the abluminal membrane [468]. The BCRP mRNA is highly enriched at the human brain microvasculature, relative to total brain [483]. Ivermectin is not a substrate for BCRP as the brain uptake of ivermectin is not increased in the bcrp knockout mouse [487]. Ivermectin is a Pgp substrate, and brain ivermectin uptake is increased in the Pgp knockout mouse [472,487]. However, ivermectin is a highly polar drug macrocyclic lactone with a MW of 875 Da, which are not the molecular properties of a small molecule that penetrates the BBB via free diffusion, as reviewed in Section 6.1. The high brain uptake of ivermectin in the Pgp knockout mouse suggests ivermectin traverses the BBB via an unknown transport system.

#### 6.3.3. SLC Efflux Transporters

SLC efflux systems at the BBB include transporters for both amino acids and organic anions. The acidic amino acids, glutamate and aspartate, are also excitatory amino acids [488], and CNS homeostasis is maintained by preventing changes in plasma concentrations of these amino acids causing similar changes in brain levels of the excitatory amino acids. A saturable carrier for *L*-glutamate and *L*-aspartate was identified at the BBB by the BUI method [455]. However, the rate of influx of glutamate or aspartate from blood to brain was lowest of any of the amino acids [455]. In parallel with this low rate of influx, the rate of efflux of the acidic amino acids from brain to blood was high [489]. These observations led to the hypothesis that the BBB acidic amino acid transporter was an active efflux system [490]. The active efflux of glutamate from brain to blood was confirmed with the BEI method [456]. The principle acidic amino acid transporters are the sodium-dependent excitatory amino acid transporter (EAAT)1 (SLC1A3), EAAT2 (SLC1A2), and EAAT3 (SLC1A1) [491], and these transporters are localized to the abluminal membrane of the BBB [492]. At the human brain capillary, the expression of EAAT1 is relatively high, 5.0 pmol/mg protein [409]. Small neutral amino acids may also play a role in neurotransmission. Serine is a neurotransmitter modulator, and alanine is a ligand for glycine neurotransmission [488]. Small neutral amino acids, such as alanine or serine are transported via the alanine (A)-system [493]. The A-system amino acid transporter was cloned, and this sodium dependent transporter was designated amino acid transporter 2 (ATA2, SLC38A2) [494]. ATA2 was subsequently localized to the BBB and identified as an active efflux system [495]. ATA2 is exclusively localized to the abluminal endothelial membrane [468,492].

Organic anion transporters also operate as BBB active efflux systems. The SLC22 gene family, which includes 28 transporters, comprises two parallel clades encoding for organic anion transporters (OAT) and organic cation transporters (OCT, OCTN) [446]. The mRNA encoding for OAT3 (SLC22A8) is highly expressed at the rat brain capillary [345]. Proteomics studies show species differences in the expression level of the OAT3 transporter protein as the level is 2.0 pmol/mg protein in mouse brain capillaries [435], but is less than the limit of quantitation (LOQ) for human or monkey brain microvessels [360,435]. The rodent organic anion transporting polypeptide (Oatp)1a4 is most homologous with the human OATP1A2 (SLCO1A2, previously named SLC21A3) and is expressed at the BBB [496], as well as retinal capillaries that form the blood–retinal barrier (BRB) [497]. Oatp1a4 is highly expressed at the rat arachnoid membrane [498], which is an important barrier system in brain that separates the CSF from the dura mater. Recent studies show that solute transport from CSF to the peripheral blood may take place via active transport across the arachnoid membrane. A fluorescent Oatp1a4 ligand, sulforhodamine (SR)-101, is actively transported out of CSF to blood at a rate much faster than inulin [498]. This efflux of SR-101 from CSF is blocked by taurocholate, which has broad specificity for the anion transporters, and by digoxin, which is specific for Oatp1a4 [498]. Oatp1a4 at the BBB is expressed on both luminal and abluminal endothelial membranes [499]. Unlike OAT3, which is highly expressed at the arachnoid membrane, OATP1A2 and OATP3A1 are not detectable at the arachnoid [500]. OATP1C1 (Slco1c1) is a BBB thyroid hormone transporter that can protect mouse brain deficient for MCT8 [437], although OATP1C1 expression at the human BBB is minimal [436], as discussed in Section 6.2.8. OATP3A1 is said to be another alternative thyroid hormone transporter [501]. However, OATP3A1 protein expression is <LOQ at the primate BBB [486].

In summary of Section 6 on CNS drug development of small molecules, present day efforts in the pharmaceutical industry are still largely entrenched in a 20th century model that is restricted to the development of small molecules that cross the BBB via free diffusion. Only drugs with a MW < 400–450 Da that form <8 hydrogen bonds with water can cross the BBB by free diffusion, and such drugs invariably only treat psychiatric disorders or epilepsy. Future small molecule CNS drug developers should consider re-directing medicinal chemistry away from the production of lipid-soluble pro-drugs, and toward the synthesis of drugs that mimic the structure of nutrients or vitamins that are substrates for SLC transporters expressed at the BBB. The SLC transporter family is complex and is composed of >400 transporters among >60 families [338]. Only a small fraction of the SLC transporters is expressed at the BBB. Therefore, the selection of an SLC transporter to be targeted for small molecule CNS drug delivery should consider the following:

- The substrate transporter profile (STP) that characterizes BBB transport in vivo should be replicated by the STP of the cloned transporter that is expressed in vitro. STPs determined with in vitro BBB models should not be used as a primary method, owing to the marked alteration of gene expression within brain endothelial cells grown in cell culture, as discussed in Section 11.7.2. The STP should be determined in vivo with methods discussed in Section 11.4.
- Evidence should be available that the targeted SLC transporter is expressed on both luminal and abluminal endothelial membranes in the human brain. As discussed above, there are species differences in the expression of certain transporters at the human vs. the animal BBB. Some SLC transporters are only expressed on the abluminal endothelial membrane, and these abluminal transporters would not be available to transport drug from blood to brain.
- The BBB CMT systems form trans-membrane cavities, as illustrated for GLUT1 and LAT1 in Figure 9, and these cavities can be sharply stereospecific with low tolerance for bulky structural changes to the substrate. As an example, if the GLUT1 carrier is targeted for brain drug delivery, the drug should be modified, not by conjugation of the drug to D-glucose, but rather by alteration of the drug structure so as to mimic the structure of the endogenous substrate, D-glucose.
- If the lead CNS drug candidate is a ligand for Pgp, or one of the other active efflux transporters at the BBB, then a co-drug needs to be developed that inhibits the BBB efflux transporter.

## 7. Absorptive-Mediated Transport of Cationic Proteins or Lectins

### 7.1. Cationic Proteins

#### 7.1.1. Cationized Proteins

Cationization of proteins raises the isoelectric point (pI) to the alkaline range, and this modification enhances cell uptake of the protein via a charge or absorptive-mediated endocytosis. A protein can be cationized either by conjugation of a polycation, such as poly-L-lysine (PLL) [502], or diamino agents such as ethylenediamine [503] or hexamethylenediamine [504], to surface carboxyl groups using 1-ethyl-3-(3-dimethylamino-propyl) carbodiimide (EDAC). Conjugation of PLL to either albumin or HRP enhances protein uptake into cultured fibroblasts [502]. Cationization with amine reagents and EDAC is a pH-controlled reaction, and the lower the pH of the chemical conjugation, the higher the degree of cationization. Hexamethylenediamine was conjugated to bovine serum albumin with EDAC at a pH of either 7.8 or 6.8 to produce moderately cationized bovine serum albumin (cBSA) of pI of 8.5–9 and highly cationized cBSA with a pI > 10, respectively [54]. Highly cationized albumin or IgG, with a pI > 10, is nephrotoxic [503]. Moderately cBSA, pI = 8.5–9, was both bound, and endocytosed, by isolated bovine brain microvessels via a saturable process that was 50% inhibited at a cationized albumin concentration (ED50) of 10.8 ± 0.1 µM [54]. The binding of cBSA to brain capillaries was competed by other polycations such as protamine or 70 kDa PLL. [^125^I]-cBSA was infused in the carotid artery for 10 min and the brain was removed and sectioned on a cryostat for thaw-mount emulsion autoradiography [54]. This showed the cBSA was localized to the brain microvasculature with measurable distribution into the brain parenchyma. The first use of cationized albumin for brain drug delivery was tested with the opioid peptide, β-endorphin, which was conjugated to the cBSA [54]. There was minimal uptake of the unconjugated β-endorphin by brain microvessels, but the β-endorphin was both bound and endocytosed by brain microvessels following conjugation of the peptide to the cBSA delivery system [54]. These in vitro investigations were confirmed with in vivo studies measuring the brain distribution of a metabolically stable and peptidase-resistant opioid peptide, [D-Ala^2^]–β-endorphin (DABE), which was conjugated to cBSA (pI = 8.5–9) with a disulfide cleavable linker using N-succinimidyl 3-(2-pyridyldithio(propionate)] [505]. The internal carotid artery perfusion (ICAP) method, coupled with the capillary depletion method [506], demonstrated transport of the DABE-cBSA conjugate through the BBB into brain parenchyma, whereas there was no BBB transport of the unconjugated DABE [505]. The DABE-cBSA conjugate was incubated with brain homogenate followed by gel filtration fast protein liquid chromatography (FPLC) to show cleavage in brain of the disulfide linker joining the DABE and the cBSA delivery vector [505]. This early study demonstrated that a pharmaceutical, such as an opioid peptide, could be chemically conjugated to a molecular Trojan horse, cBSA, for delivery across the BBB in vivo in confirmation of the earlier in vitro study [54].

Cationization of a protein enhances cell uptake, in general, including uptake into immune cells. Cationization of a heterologous protein enhances the immunogenicity and nephrotoxicity of the heterologous protein [503]. However, mild cationization of a homologous protein was shown to exert no toxicity in rats with chronic administration [507]. Rat serum albumin (RSA) was cationized at a pH of 7.8 to a pI of 8.5. The cationized RSA (cRSA) was bound and endocytosed by isolated rat brain microvessels with an ED50 of 2.5 ±1.1 µM [507]. The cRSA was cleared from plasma in rats with a T_1/2_ of 2.5 ± 0.4 h, and the organ uptake in rats of the cRSA was compared to the organ uptake of native RSA (nRSA). The spectrum of organ uptake of the cRSA was kidney > brain > liver, with no enhanced uptake in heart or lung [507]. The cRSA was administered chronically to rats at a dose of 1 mg/kg subcutaneous (SQ) 5 days a week for 8 weeks. The treatment produced no changes in organ histology, body weight, or clinical chemistry, and produced a low titer anti-drug antibody (ADA) response [507].

Enhanced cell uptake of IgG is also enabled by protein cationization. Bovine IgG was cationized with hexamethylenediamine to a pI > 10 [508]. The cationized bovine IgG (cIgG) was both bound and endocytosed by isolated bovine brain capillaries. The ED50 of binding was 0.90 ± 0.37 uM. The cIgG was radioiodinated and perfused via the internal carotid artery for 10 min followed by removal of brain, sectioning with a cryostat in the darkroom and emulsion autoradiography. The darkfield microscopy of the developed slides showed high sequestration of the cIgG around the brain microvessels, but also distribution into brain parenchyma. The [^125^I]-cIgG sequestration at the brain microvessels and the transport into brain parenchyma was completely inhibited by the co-infusion 25 mg/mL cationized IgG [508].

Therapeutic antibodies do not cross the BBB, and an early approach to brain delivery of a therapeutic monoclonal antibody (MAb) employed cationization of the antibody [509]. The concern with cationization of a monoclonal antibody (MAb) is the loss of affinity of the MAb for the target antigen following cationization. Different antibodies are expected to have different degrees of loss of antigen affinity following cationization. In the case of a potential therapeutic MAb for the treatment of AiDS, a MAb directed against the *rev* protein of the human immunodeficiency virus-1 (HIV1) was developed and designated MAb111 [510]. However, the HIV virus invades the CNS to cause neuroAIDS, and an anti-rev MAb would not cross the BBB. In an effort to enhance brain delivery of the therapeutic antibody, the pI of the MAb111 was raised from 6.6 to 9.5 by cationization [509]. The native MAb111 and the cationized MAb111 bound to the recombinant rev protein with comparable affinity. Incubation of [^125^I]-native MAb111 and [^125^I]-cationized MAb111 with human peripheral blood lymphocytes (PBLs) showed the native MAb111 did not enter the cells over the course of a 3 h incubation. However, the cationized MAb111 showed robust binding and endocytosis into the PBLs [509]. Incubation of PBLs with 25 µg/mL concentrations of the cationized MAb111 had no effect on thymidine incorporation over a 24 h period [509]. A potential therapeutic MAb for Alzheimer’s disease (AD) is an antibody against the amino terminal region of the Abeta peptide of AD, and one such antibody is the AMY33 antibody [511]. The pI of native AMY33 was 7.0, and this was raised to a pI of ~8 or a pI of ~9 by adjusting the molar ratios of hexamethylenediamine and EDAC, and the pH of the cationization reaction [512]. The binding of the native AMY33 and the cationized AMY33 to the Aβ^1−28^ amyloid peptide showed the dissociation constant (KD) of binding of the native and cationized antibodies was 1.4 ± 0.3 nM and 4.2 ± 0.7 nM, respectively. The cationized AMY33 also retained high affinity for binding Abeta amyloid in autopsy AD brain as shown by immunohistochemistry using either the native or cationized AMY33 antibody [512]. The use of cationized antibodies, and absorptive-mediated transcytosis for brain antibody delivery, have been reviewed [513,514]. Since these early studies on antibody cationization, a preferred method of therapeutic antibody transport into brain has emerged, which is the engineering of a bispecific antibody (BSA) that enters brain via receptor-mediated transport (RMT), as discussed in Section 8.3.4.

#### 7.1.2. Endogenous Cationic Proteins

Protamines are endogenous arginine-rich proteins with a MW of 4–7 kDa and a pI~10. Early studies showed the internal carotid artery infusion of 0.3–1.5 mg/kg of protamine over a 1–2 min period caused BBB disruption to HRP, and electron microscopy showed the BBB disruption was due to opening of tight junctions [256]. The administration of protamine via the IV route did not cause BBB disruption [257]. However, IV protamine can still enhance the delivery of macromolecules across the BBB by absorptive-mediated transport. Protamine binds anionic domains on the luminal surface of the brain endothelium. Electron microscopy shows the luminal membrane of the brain endothelium is rich in anionic sites composed of sialic acid residues on glycoproteins [515]. In parallel, protamine binds anionic domains on proteins such as albumin with a KD of 6–22 µM [516]. Therefore, protamine can act as a molecular Trojan horse for albumin delivery to brain via the non-covalent electrostatic interactions between protamine and both albumin and the luminal membrane of the BBB. Protamine enhances the binding and endocytosis of native rat serum albumin (nRSA) by isolated bovine brain capillaries with an ED50 of 70 µM protamine, but has no effect on uptake of sucrose by the microvessels [516]. The co-injection of 1.5 mg/kg of histone free protamine base and nRSA IV in rats causes a 34-fold increase in RSA uptake by liver, an 11-fold increase in RSA uptake by lung, a 3-fold increase in RSA uptake by kidney, and a 2-fold increase in RSA uptake by brain and heart [516].

Histones are endogenous lysine/arginine-rich proteins with a MW of 11–15 kDa and a pI of ~10. Early work by Ryser [517] showed that polycationic substances such as protamine or histone can acts as mediators for the cellular uptake of proteins. More recently, histones have been proposed as agents for drug delivery [518]. Similar to protamine, histones are endocytosed at the BBB in vivo, but also have toxic effects on the endothelial membrane [259]. Histone is bound and endocytosed by isolated brain microvessels with a KD of binding of 15 ± 3 µM, via a process that is inhibited by protamine and poly-L-lysine [259]. Following IV administration, histone is cleared rapidly from plasma with a T_1/2_ of 13 ± 5 sec, and is cleared primarily by kidney, lung, liver, and spleen. The volume of distribution (VD) of histone in brain is 10-fold greater than the VD for albumin at 60 min after IV injection in the rat [259]. Following 10 min of internal carotid artery perfusion in the rat, the brain VD of histone is 9-fold greater than the VD of albumin in the homogenate fraction of brain. However, capillary depletion analysis shows all of the histone taken up by brain is sequestered within the capillary endothelium without transcytosis into the post-vascular compartment of brain. The internal carotid artery infusion of a low dose of histone cause leakiness of the BBB and a seven-fold increase in the brain VD of albumin, which should be confined to the brain blood volume [259]. The toxicity of polycations such as histone at the BBB is discussed below in Section 7.3.1.

#### 7.1.3. Cell-Penetrating Peptides

The prototypic cell-penetrating peptide (CPP) is a portion of the *tat* protein of HIV1, which encompasses an 11-amino acid (AA) sequence enriched in arginine (Arg) and lysine (Lys) residues. In an early study, a 36-mer peptide derived from the HIV1 tat protein was conjugated to β-galactosidase, which resulted in increased cellular uptake of the β-galactosidase [55]. Following IV injection of the tat-β-galactosidase conjugate, the enzyme uptake was enhanced for heart, liver, and spleen, and to a lesser extent for lung and skeletal muscle, but there was no enzyme uptake by brain mediated by the tat peptide [55]. Subsequently, a fusion protein was engineered that was composed of β-galactosidase and the tat peptide domain, (GGGG)_4_YGRKKRRQRRR, which included the 11-AA tat peptide sequence following the glycine (G)_4_ linker [519]. At 4–8 h after IV administration, enzyme activity was visible histochemically in the brain parenchyma [519]. This delayed appearance of the tat-enzyme fusion protein in brain is difficult to resolve with other results showing complete inactivation of bacterial β-galactosidease enzyme in mouse brain by 4 h after IV injection of a TfRMAb–β-galactosidease conjugate [520]. Subsequent studies failed to show any enhancement of protein uptake by brain using the tat peptide. A fusion protein of tat and lysosomal enzymes, beta-glucuronidase (GUSB) [521] or arylsulfatase A (ASA) [522], showed no enzyme uptake by brain in vivo. Brain uptake of the tat peptide alone could not be detected using radiolabeling methods including PET scanning in the mouse [523].

Another early CPP was the 16-AA highly cationic penetratin, which is derived from a Drosophila protein [524]. However, following radiolabeling of penetratin with 111-indium, the brain uptake of the peptide was very low, 0.1%ID/g, in the mouse [523]. The penetratin, and other CPPs, including tat, were conjugated with (1,4,7,10-tetraazacyclododecane-1,4,7,10-tetraacetic acid), also known as DOTA or tetraxetan, for chelation of the 111-indium. This is the preferred mode of radio-labeling of rapidly cleared peptides, as opposed to radio-iodination. The small molecular metabolites generated by peripheral degradation of peptides labeled with 111-indium are sequestered in peripheral tissues. In contrast, the radio-tyrosine generated by peripheral degradation of the iodinated peptide, can cross the BBB, and lead to artifactually high brain radioactivity [525], as discussed in Section 11.4.4.

SynB1 was an 18-AA highly cationic CPP that was taken up by brain following internal carotid artery perfusion via a saturable process with an ED50 of 5.5 µM [526,527,528]. The BBB transport after internal carotid artery perfusion was inhibited by another cationic peptide, poly-L-lysine [528]. However, the addition of serum to the perfusate suppressed brain uptake [527]. The brain uptake of SynB1, labeled with 111-indium, after IV administration is at the background level, 0.1%ID/g, similar to tat or penetratin [523]. The lack of significant BBB transport of the CPPs following IV administration necessitated the brain delivery of the CPP by ICV administration [529,530].

### 7.2. Lectins

Wheat germ agglutinin (WGA) is a 36 kDa glycoprotein that is a lectin, i.e., a sugar-binding protein, with affinity for N-acetyl D-glucosamine and sialic acid [531]. The luminal membrane of the BBB expresses sugar sites including sialic acid [515], and WGA binds the luminal membrane of the brain endothelium as demonstrated by lectin-gold electron microscopy [532]. A conjugate of WGA and HRP bound to cells, which triggered absorptive-mediated endocytosis [533]. Electron microscopic histochemistry of brain following the IV administration of 50 mg/kg of an HRP–WGA conjugate in the mouse demonstrated labeling of the luminal endothelial membrane as well as some endothelial vesicles. Vesicles within vascular pericytes were also labeled, which indicates the HRP–WGA conjugate transcytosed through the endothelial barrier [170]. WGA has been used as a surface ligand on liposomes for brain delivery [534]. Apart from WGA, another lectin used for drug delivery is the ricinus communis agglutinin (RCA) [535]. RCA, which binds D-galactose groups on surface glycoproteins, avidly binds both the luminal and abluminal membranes of the brain capillary endothelium [532]. RCA is a product of *Ricinus communis* seeds, which also express a toxin, ricin. RCA and ricin are distinct proteins [536]. While there is evidence that RCA binds the BBB [532], there is no direct evidence to date that the ricin toxin binds the BBB. Ricin is composed of an A chain, which is the toxic domain, and an B chain, which binds cell surface carbohydrates to mediate endocytosis. Working on the assumption that the ricin B chain binds the luminal membrane of the brain endothelium to trigger transport into brain, ricin toxin B chain (RTB) fusion proteins were engineered for the treatment of the brain in lysosomal storage disease. A fusion protein of RTB and iduronidase (IDUA), the enzyme mutated in MPSI, or RTB and beta galactosidase 1 (GLB1), the enzyme mutated in GM1 gangliosidosis, were engineered and expressed in plants [537,538]. No evidence that the RTB fusion proteins cross the BBB in vivo was presented [537,538]. The RTB-IDUA fusion protein was administered to MPSI mice, which produce no IDUA [539]. However, brain IDUA enzyme activity was barely above background after the IV administration of 2 mg/kg of the RTB-IDUA fusion protein [539].

### 7.3. Toxicity of Cationic Proteins and Lectins

#### 7.3.1. Toxicity of Cationic Proteins

The intra-arterial infusion of protamine causes BBB disruption [258], owing to enhanced trans-endothelial vesicular transport [540]. This treatment produces toxic effects in brain including shrunken spongiotic neurons and reactive astrogliosis [260]. The IV administration of polycationic peptides can lead to death, which was demonstrated in the case of the K16ApoE peptide [541]. The K16ApoE peptide is a 36-mer composed of 16 lysine residues (Lys or K) followed by a 16-AA sequence derived from human ApoE [542]. Of the 36 AAs in this peptide, 24 are cationic amino acids (Lys, Arg). The Lys-rich domain of the peptide is intended to bind anionic domains of therapeutic proteins, which alone do not cross the BBB. The apoE peptide domain was intended to bind the ApoE receptor, to trigger receptor-mediated transcytosis through the BBB via the low-density lipoprotein receptor (LDLR). However, as discussed in Section 8.1.6, the LDLR is not expressed on the microvascular endothelium of brain. Given the highly cationic charge of the K16ApoE peptide, the likely mechanism of BBB transport is either AMT via a charge mechanism, or BBB disruption caused by the highly cationic peptide. Similar to other cationic import peptides, the K16ApoE peptide is rapidly removed from plasma in <5 min [543]. In an attempt to deliver the TPP1 enzyme to brain in TPP1 null mice, the enzyme was co-injected with the K16ApoE peptide at a dose of 40–120 nmol of the peptide. The 120 nmol dose of K16ApoE was lethal in all animals [541]. A dose of 40 nmol of the K16ApoE peptide increased brain uptake of Alexa Fluor 647-conjugated TPP1. However, fluorescent microscopy of brain revealed a highly punctate distribution of the enzyme in brain [541]. This punctate pattern was identical to that reported by Brightman in 1977 [544] following BBB disruption with intra-arterial hyperosmolar solutions. This suggests the K16ApoE peptide delivers enzyme to brain via BBB disruption, not RMT on a presumptive LDLR at the BBB. Neurotoxicty of CPPs may be a general property of these highly cationic agents. The intra-cerebral injection of 10 μg of penetratin in rat brain produces neurotoxic cell death and neuroinflammation [56]. Cellular toxicity has been reported for cells exposed to cationic CPPs [545,546].

#### 7.3.2. Toxicity of Lectins

WGA is toxic to Caco-2 epithelial cells in culture at concentrations of 0.25–2.5 µM [547,548]. Cell electrical resistance is diminished in parallel with increased permeability of the monolayer to mannitol and 3 kDa dextran. In another study, human peripheral blood mononuclear cells (PBMC) were treated with low concentrations, 14 nM, of WGA. The supernatants from these cells were toxic to Caco-2 cell monolayers, resulting in increased permeability. The toxic effects of the WGA treated PBMC supernatant were reduced with interleukin blocking antibodies [549]. These findings corroborate early results from the 1970s that WGA at concentrations of 1–5 µg/mL are toxic to cells [550]. Despite the toxicity of WGA, this molecule continues to be developed as a brain drug delivery vector [534].

In summary, delivery of drugs via absorptive-mediated transport (AMT) is problematic, particularly when compared to receptor-mediated transport (RMT) that is reviewed in the next section. First, ligands that traverse the BBB via AMT have dissociation constants (KD) of binding to the targets on the BBB in the μM range, which is up to 3 log orders of magnitude lower than the affinity of ligands that traverse the BBB via RMT. Second, ligands that cross the BBB via AMT are largely sequestered within the endothelium with minimal exocytosis into brain parenchyma. A polycationic protein, histone, was nearly completely sequestered within the vascular compartment [259]. Cationized albumin is largely sequestered in the vascular compartment of brain [54]. WGA is said to undergo transcytosis through the BBB [170], but inspection of the micrographs shows the lectin is largely sequestered within the endothelium. Third, polycationic proteins or lectins are toxic with narrow therapeutic indices. A dose of 40 nmol of the K16ApoE peptide was necessary to mediate brain uptake a K16ApoE/enzyme complex, but a dose of 120 nmol of K16ApoE peptide was lethal in all animals [541]. WGA is cytotoxic at concentrations of 1–5 µM [548,550], and generates toxins in cells at concentrations at low as 14 nM [549]. The next section will review the delivery of biologics to brain via receptor-mediated transport (RMT).

## 8. Receptor-Mediated Transport of Peptides and Monoclonal Antibodies

### 8.1. Receptor-Mediated Transporters at the Blood–Brain Barrier

#### 8.1.1. Insulin Receptor

**Figure 10:**
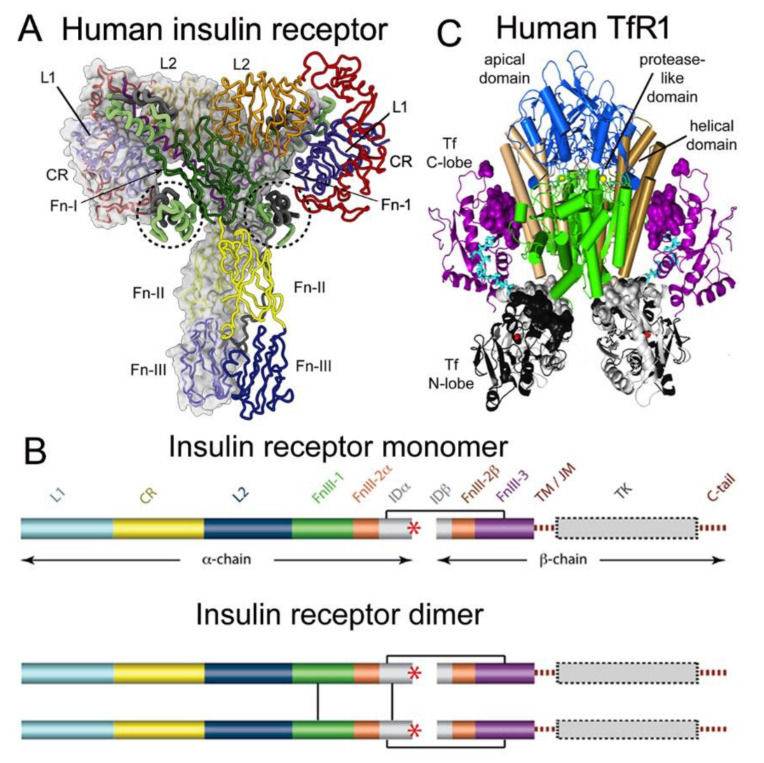
**Structure of human insulin receptor and human transferrin receptor.** (**A**) Complex of the IR tetramer and insulin is shown as determined by cryo-EM. The abbreviations of the domains are defined in the text. The structure shows a complex of 2 alpha chains, 2 beta chains, and 4 bound insulin molecules, two of which are encircled. Reproduced with permission from [551], Copyright© 2021 Elsevier, as originally reported in [552]. (**B**) 2-dimensional structure of IR monomer and dimer. The inter-chain and intra-chain disulfides are shown. Carboxyl terminus of alpha chain shown by red asterisk. Reproduced from [553], Copyright© 2011 licensed under Creative Commons Attribution License (CC-BY). (**C**) The complex of human TfR1 ECD and human holo-Tf is formed from 2 receptors and 2 holo-Tf molecules. The membrane surface is at the bottom and the apical domain (blue) is at the top. The regions shown in brown are the protease-like domains; the regions shown in brown/tan are the helical domains. The N-lobe and C-lobe of holo-Tf are shown in gray/black and purple, respectively. An Fe^+3^ atom buried in each N-lobe is red, and the N-lobe and C-lobe linker is shown in cyan. Reproduced with permission [554].

The insulin receptor (INSR or IR) is a hetero-tetrameric structure formed by two alpha chains, which bind insulin, and two beta chains, which are the tyrosine kinase domains, and the three-dimensional structure revealed by cryo-electron microscopy is shown in Figure 10A.

The long form (B form) of the human IR (HIR) is translated as a 1382-AA polypeptide, which includes a 27-AA signal peptide [555]. The receptor is cleaved into separate alpha and beta chains at the furin cleavage site, RKRR, at AA 732–735 [556]. The domains of the alpha chain include the first leucine-rich domain (L1), the cysteine-rich (CR) domain, the second leucine-rich (L2) domain, the first fibronection III domain (FnIII-1), the first part of the second FnIII domain (FnIII-2), and the first part of the insert domain (IDα); the final 12 amino acids of the alpha chain form the αCT domain, which is the high affinity insulin binding site. The beta chain is composed of the second part of the IDβ domain, the second part of the FnIII-2 domain, the third FnIII domain (FnIII-3), the transmembrane (TM) domain, the juxta-membrane (JM) domain, the tyrosine kinase (TK) domain and the carboxyl terminus (Figure 10B, top). The furin cleavage produces the ECD of the IR, which is about 900 AA in length [555]. The cryo-EM of the IR/insulin complex shows insulin binding at 2 sites: the L1/αCT interface and the FnIII-1/FnIII-2 interface [552].

Insulin is synthesized as a 110-AA prepropeptide, which includes a 24-AA signal peptide and an 86-AA propeptide (AAW83741). The 35-AA C-peptide is formed by AA 31–66. Preproinsulin is cleaved internally to release the C-peptide, as well as a separate 30-AA B chain and a 21-AA A chain. Following cleavage, the A and B chains are joined by two disulfide bonds [551]. There are two tyrosine (Tyr) residues, which are sites of radio-iodination, in both the A chain and the B chain. The fasting plasma insulin concentration is ~0.3 nM in primates and humans [557,558], which is ~100-fold lower than the IR concentration at the brain capillary, 24 nM [559].

The characterization of insulin binding to the IR at the BBB was performed with radio-receptor assays and isolated brain microvessels, as first reported in 1981 for bovine brain capillaries [560], and in 1985 for human brain capillaries [561]. Insulin binding at the human brain capillary was saturable with a KD of the high affinity binding site of 1.2 ± 0.5 nM. The saturable binding site for insulin at the human BBB was shown to be the IR, as affinity cross-linking of [^125^I]-insulin to the saturable binding site showed the MW of this site was 127 kDa, which is the size of the alpha subunit of the IR [561]. Insulin was rapidly endocytosed by the brain microvessels, and was metabolically stable over the course of a 60 min incubation at 37 °C [561]. The enrichment of the IR at the microvasculature of brain was demonstrated by immunohistochemistry for primate brain [61] and mouse brain [562]. The IR is also widely expressed in brain parenchyma, particularly in neurons [563].

Insulin is exocytosed into the brain post-vascular compartment following binding and endocytosis at the BBB. This was demonstrated with a 10 min carotid artery infusion of [^125^I]-insulin in the rabbit, followed by removal and freezing of the brain [564]. Thaw-mount autoradiography showed the distribution of insulin into brain parenchyma, and HPLC analysis of acid-ethanol extracts of brain showed the radioactivity in brain was unmetabolized insulin. The transcytosis of insulin across the BBB was completely suppressed by the co-infusion of high concentrations of unlabeled insulin [564]. Selective transport of insulin into brain, as compared to CSF, following IV administration was demonstrated in the rat using [^125^I-Tyr-A14]-insulin [565]. The latter is HPLC purified mono-iodinated insulin and is considered ‘receptor grade’ iodinated insulin [566]. [^125^I-Tyr-A14]-insulin was 95% cleared from plasma within 5 min of IV injection [565], which indicates the T_1/2_ of plasma clearance of insulin is ~2 min. Following IV administration, [^125^I-Tyr-A14]-insulin entered brain rapidly within 5 min. The brain uptake of insulin in vivo, as well as by cultured rat brain microvascular endothelial cells, was blocked by the IR antagonist, S961, which indicates that brain uptake of insulin is mediated by the BBB IR [565]. An alternative pathway of insulin transport into brain has been proposed based on studies with the EndoIRKO mouse [567], which has a targeted deletion of the IR in endothelial cells [568]. However, the study of [^125^I]-insulin transport in this mouse model was performed over a 20 min period after IV administration of radio-iodinated insulin [567]. The brain uptake of radioactivity is most likely artifact, because (a) the plasma T_1/2_ of insulin is only ~2 min [565], and (b) the insulin was labeled with 125-iodine and chloramine T, which is an oxidative reaction that iodinates insulin at multiple tyrosine residues. This form of insulin is subject to rapid degradation in vivo, which produces free [^125^I]-tyrosine that may enter brain via transport on BBB LAT1. Artifacts in the brain uptake of radio-iodinated peptides following IV administration are discussed in Methods, Section 11.4.4. Insulin transport across the BBB has also been investigated with in vitro BBB models in cell culture. As discussed in Section 11.7.2, in Methods, in vitro BBB models should be used to support primary in vivo studies, as in vitro BBB models are leaky compared to the BBB in vivo, as recently reviewed [569]. In one in vitro BBB model, insulin transport is non-saturable and occurs through the leaky para-cellular route [570]. The same model also reports non-saturable transport of transferrin through the in vitro BBB [571]. In contrast, another in vitro BBB model using primary cultures of brain microvascular endothelial cells, and receptor-grade [^125^I-Tyr-A14]-insulin, shows insulin transcytosis through the monolayer is mediated via the insulin receptor as transfer from the apical surface to the basolateral surface is blocked by the IR antagonist, S961 [572]. Heat-denatured labeled insulin was used as a control for a paracellular leak [572].

#### 8.1.2. Transferrin Receptor

There are two human transferrin receptors (TfR), TfR1 and TfR2 [573], which have 39% AA identity [574]. The TfR isoform expressed at the BBB was identified with a BBB genomics investigation as TfR1 [438]. Northern blot studies with the cloned rat TfR1 cDNA showed a primary transcript of 5.0 kb encoding the TfR1 in both brain parenchyma, and at the BBB. In addition, a BBB-specific TfR1 transcript of 6.6 kb was detected by Northern blotting of brain capillary-derived RNA [438]. The three-dimensional structure of the complex of human holo-Tf and the human TfR1 ECD has been determined [554]. The tetrameric complex is formed by a dimer of TfR1s and two molecules of holo-Tf as shown in Figure 10C. The TfR1 is synthesized as a 760-AA protein that includes an intracellular domain, AA 1–67, the transmembrane domain, AA 68–88, the stalk domain, AA 89–120, which forms two disulfide inter-chain bonds, two protease-like domains, AA 121–188 and AA 384–606, an apical domain, AA 189–383, and a helical domain, AA 607–760 [554]. The ECD is formed by AA 121–760, which is a monomeric structure that lacks the stalk domain forming the inter-chain disulfide linked dimer. Transferrin (Tf) exists in plasma in three forms: about 40% is apo-Tf, which does not bind the TfR1 at physiologic pH, about 30% is monoferric holo-Tf, and about 30% is diferric holo-Tf [554]. The affinity of diferric holo-Tf for the TfR1 is 8- to 9-fold higher than the affinity of mono-ferric Tf for the receptor [575]. The Tf concentration in plasma is about 45,000 nM [576], and the concentration of holo-Tf is about 25,000 nM. This plasma concentration of holo-Tf is nearly 1000-fold greater than the TfR1 concentration at the brain microvasculature, which is 40 nM [559].

The high expression of the TfR at the brain microvasculature was shown in 1984 by immunohistochemistry of rat brain using the murine OX26 MAb against the rat TfR [577]. In 1987, the BBB TfR was shown to mediate the transcytosis of Tf [578], and the TfR at the human BBB was characterized by radio-receptor assays and isolated human brain microvessels [579]. Subsequent work questioned whether Tf underwent transcytosis through the BBB, as opposed to a model of endocytosis of holo-Tf into the brain endothelium followed by retro-endocytosis of apo-Tf from the brain endothelium back to blood [580,581]. The evidence for this retro-endocytosis model was two-fold. First, the IV administration of dual labeled [^59^Fe, ^125^I]-Tf in rats showed that the ^59^Fe radioactivity accumulated in brain to a greater extent than the ^125^I radioactivity [580]. However, this observation is also consistent with a model of Tf transcytosis into brain followed by uptake of holo-Tf by brain cells, release of iron and reverse transcytosis of apo-Tf to blood. Support for this reverse transcytosis model was produced with the BEI method, which showed that both apo-Tf and holo-Tf undergo reverse transcytosis from brain to blood in vivo [452]. In addition to reverse transcytosis of [^125^I]-apo-Tf, any ^125^I-iodide released from [^125^I]-Tf in brain is rapidly exported from brain to blood [582]. The second line of evidence used to support the reverse-endocytosis model was pre-embedding immune electron microscopy, which identified the TfR only on the luminal endothelial membrane, and not on the abluminal membrane [583]. However, abluminal receptors are not detected with pre-embedding methods, and post-embedding techniques are required to visualize abluminal receptors [515]. Confocal microscopy of unfixed rat brain capillaries identified the TfR on both luminal and abluminal brain capillary endothelial membranes [584]. The Tf transcytosis model was further supported by electron microscopy of rat brain following a 10 min carotid artery infusion of the OX26 MAb conjugated with 5 nm gold particles [585]. The gold-labeled antibody was observed bound to the luminal membrane, packaged within 100 nm intra-endothelial transcytotic vesicles, and exocytosed into the brain interstitial space [585]. The transcytosis model was also confirmed with the internal carotid artery infusion of [^125^I]-rat holo-Tf in rats, followed by removal of the brain for thaw-mount emulsion autoradiography. This worked showed that holo-Tf penetrates well into brain parenchyma within just 5 min of arterial infusion [586]. Holo-Tf distribution into the post-vascular brain was completely suppressed by infusion of the labeled Tf in 10% rat serum, which contains 2500 nM of holo-Tf [586].

The TfR is also highly expressed at the choroid plexus epithelium, which forms the blood–CSF barrier. Proteomics studies show the TfR1 is expression at the choroid plexus is 16-fold greater than the expression of the insulin receptor at the blood–CSF barrier [480]. The high expression of the TfR1 at the choroid plexus correlates with the high distribution of an MAb against the TfR1 into the CSF. A high affinity MAb against the human TfR1, which cross reacts with the TfR1 of Old World primates, distributes into CSF of Rhesus monkeys following IV administration with a 23 h CSF/serum ratio of 4.8% at an injection dose of 3 mg/kg [587]. The TfR1 is widely expressed in the brain parenchyma, as demonstrated by film autoradiography of rat brain with [^125^I]-ferrotransferrin [588].

#### 8.1.3. IGF Receptor

The insulin-like growth factors (IGF)-1 and IGF-2 both bind with high affinity to the IGF1 receptor (IGFR), which is similar in structure to the IR [589]. IGF1 and IGF2 binding to the IGFR expressed at the human brain microvessel was reported in 1988 [590]. Both peptides are 7.5 kDa and both bind with high affinity to the BBB IGFR. The binding affinity for IGF2, KD = 1.1 ± 0.1 nM, is about twice the affinity for IGF1, KD = 2.1 ± 0.4 nM, and insulin is a very weak inhibitor of IGF1 or IGF2 binding to the human BBB IGFR [590]. The binding of either peptide to the BBB receptor is strongly inhibited by serum [590] which contains high affinity IGF binding proteins (IGFBP) [591]. Both peptides are endocytosed into the capillary endothelium [590]. Affinity cross-linking studies with either [^125^I]-IGF1 or [^125^I]-IGF2 show the MW of the saturable binding site of the human BBB IGFR is 141 kDa [590], which corresponds to the size of the alpha subunit of the IGFR [589]. Carotid arterial infusion of [^125^I]-IGF1 or [^125^I]-IGF2, in the absence of serum, shows that both peptides traverse the BBB and enter brain parenchyma via saturable process [592].

IGF-2 also binds with high affinity to the cation independent mannose 6-phosphate receptor (CI M6PR). However, the size of the CI M6PR is ~300 kDa, and the affinity cross-linking of IGF2 to the human brain microvessel shows no binding of IGF2 to a receptor larger than 141 kDa [590]. The absence of the CI M6PR on the BBB is the reason that mannose 6-phosphorylated lysosomal enzymes do not cross the BBB, as discussed in Section 8.3.1. A fusion protein of IGF2 and NAGLU, a lysosomal enzyme, does not cross the BBB in vivo, and must be administered to brain by ICV injection [89]. Unlike insulin, IGF1 and IGF2 are avidly bound by IGFBPs [591], and this is the presumptive reason for the lack of transport of an IGF2 fusion protein into brain via the BBB IGFR. The expression of the IGFR at the luminal membrane of the brain endothelium has been confirmed by pre-embedding electron microscopic histochemistry of rat brain [593]. As discussed above, detection of abluminal receptors at the brain endothelium requires a post-embedding labeling method [515]. The IGFR is also expressed in brain on both neurons and glial cells [594]. The gene expression of IGF-2, but not IGF-1, at the brain capillary endothelium was discovered with a rat brain capillary genomics program [438].

#### 8.1.4. Leptin Receptor

**Figure 11:**
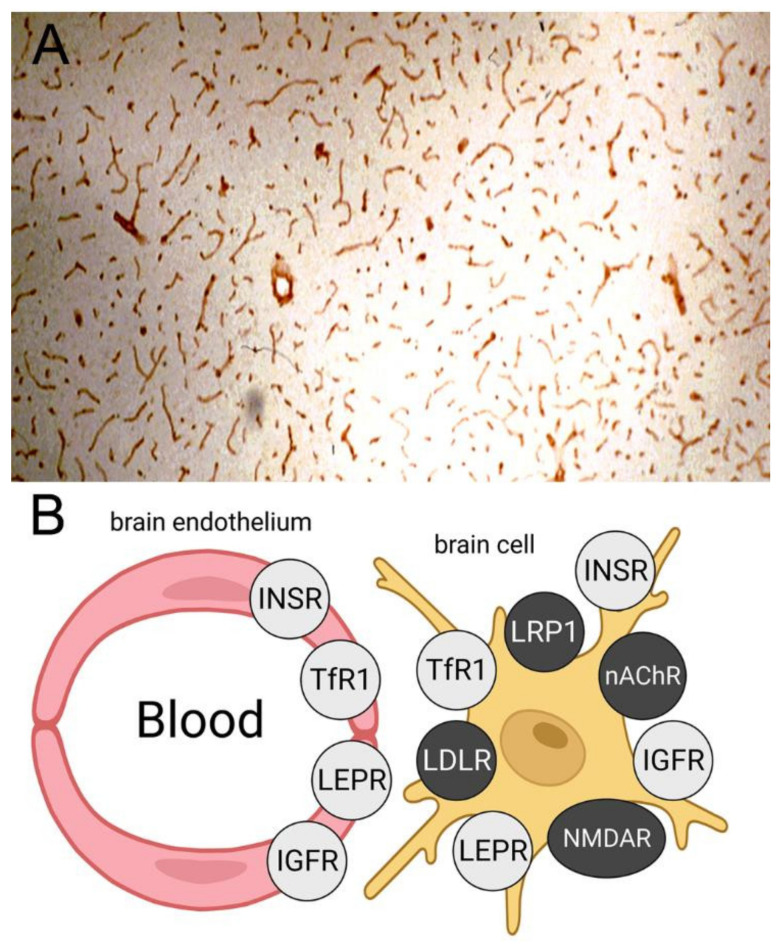
**Identification of BBB RMT targets by immunohistochemistry of brain.** (**A**) Immunohistochemistry of rat brain with an antibody to all isoforms LEPR. Reproduced with permission from [596], Copyright© 1998 John Wiley & Sons. (**B**) Expression of INSR, TfR1, IGFR, and LEPR on both the brain endothelium and on brain cells. In contrast, immunohistochemistry shows receptors such as LRP1, LDLR, NMDAR, and nAChR are expressed on brain cells but not endothelium. Reproduced from [525], Copyright© 2020 licensed under Creative Commons Attribution License (CC-BY).

A high affinity binding site for leptin was identified with radio-receptor assays and isolated human brain microvessels [595]. The KD of leptin binding was 5.1 ± 2.8 nM. Leptin binding was not inhibited by insulin or IGF-1, and leptin was endocytosed by the human brain microvessels. The Bmax of binding, in pmol/mg protein, was comparable to the Bmax of binding of insulin, Tf, or the IGFs to the human brain capillary [595]. PCR shows the predominant leptin receptor (LEPR) expressed at the BBB is the short form of the receptor [596], which has a truncated intracellular domain [597]. [^125^I]-leptin transport across the BBB in vivo in the rat has been confirmed with a carotid artery infusion method, and BBB leptin transport was saturable [598]. Leptin was also actively cleared by the choroid plexus, although there was a delay in leptin delivery into CSF [598]. Leptin distributes to CSF in humans and the CSF leptin parallels the plasma leptin concentration with a CSF/plasma ratio of 1.4–2.0% [599]. Leptin activation of cells leads to an increase in the STAT3 transcription factor, and leptin responsive cells were detected in brain in vascular endothelium, choroid plexus epithelium, and neurons [600]. Immunohistochemistry of rat or human brain showed the LEPR was highly expressed at the brain microvasculature [596], as shown in Figure 11A.

The continuous immune staining of the microvasculature in brain in Figure 11A is evidence for an endothelial origin of the microvascular LEPR.

#### 8.1.5. LRP1 Receptor

The LDL receptor related protein 1 (LRP1) has been targeted for brain drug delivery with a number of peptide-based Trojan horses, as reviewed in Section 8.2.1. Targeting LRP1 for brain drug delivery assumes this receptor is expressed on the brain capillary endothelial membrane, including the luminal endothelial membrane. Early work used an in vitro BBB model to investigate LRP1-mediated transport [601,602]. As discussed in Section 11.7.2, in vitro models of the BBB should not be used as a primary line of investigation of BBB transport, owing to the loss of multiple BBB functions when brain endothelial cells are grown in cell culture. The in vivo evidence is that LRP1 is expressed in brain, but not at the microvascular endothelium. Early immunohistochemistry (IHC) of human brain found LRP1 expression in neurons and astrocytes, but not in endothelium [603]. In situ hybridization of rat brain shows LRP1 mRNA in neurons and astrocytes, but not in endothelium [604]. Endothelial LRP1 was originally suggested as the mechanism of clearance from brain of the Abeta amyloid peptide of AD [605]. The intra-cerebral injection of [^125^I]-Abeta in brain results in rapid decline of brain radioactivity, and this was attributed to LRP1-mediated efflux of the Abeta peptide across the BBB [605]. However, subsequent work showed that LRP1 ligands, such as α_2_-macroglobulin, do not efflux from brain following intra-cerebral injection [606]. When Abeta peptide content in brain was measured not by radioactivity, but by liquid chromatography mass spectrometry (LC-MS), the decline in brain Abeta after intra-cerebral injection was shown to be due to peptide metabolism in brain, not peptide efflux across the BBB [607]. It is not possible to quantify Abeta efflux from brain across the BBB using [^125^I]-Abeta and radioactivity measurements. The labeled peptide is rapidly degraded in brain to either [^125^I]-tyrosine, which can efflux to blood via transport on LAT1, or to [^125^I]-iodide, which is rapidly exported from brain to blood [582]. More recent work implicates the role of LRP1 on astrocytes [608] or pericytes [609]. In the schematic of receptor localization in brain, LRP1 is localized to brain cells, not the brain endothelium (Figure 11B). Expression of LRP1 at brain cells beyond the BBB explains the lack of brain uptake of a MAb against LRP1 following IV antibody administration [430].

#### 8.1.6. LDL Receptor

The low-density lipoprotein (LDL) receptor (LDLR) transports lipoprotein-bound cholesterol. Protein components of lipoproteins, such as apolipoprotein B-100 (ApoB), bind to the cell surface LDLR to trigger transport of lipoprotein-bound cholesterol into the cell. As discussed in Section 8.2.1, a number of peptide Trojan horses have been developed for brain targeting of the presumptive LDLR on the luminal surface of the BBB. Expression of the LDLR at the BBB was identified in an in vitro BBB model [610]. However, in vitro models may not predict BBB function in vivo as discussed in Section 11.7.2. IHC of brain does not detect the LDLR at the microvascular endothelium, although LDLR expression is observed in neurons [562]. In this same study, the insulin receptor is highly enriched at the brain microvasculature [562], which serves as a positive control for the IHC of the LDLR. Lack of expression of the LDLR, at least at the luminal membrane of the endothelium, is consistent with the lack of transport of LDL-bound cholesterol from blood to brain [611,612,613]. An early study shows that plasma cholesterol equilibrates with brain cholesterol over a time-frame of months [614]. In the adult brain, all cholesterol in brain is synthesized de novo [613].

#### 8.1.7. Nicotinic Acetylcholine Receptor

The rabies virus gains access to the CNS via the binding of a rabies virus glycoprotein (RVG) to the nicotinic acetylcholine receptor (nAChR) in skeletal muscle [615,616]. Working on the assumption that the nAChR was also expressed at the BBB, a truncated 29-AA peptide derived from the RVG was tested as a BBB Trojan horse [297]. However, the studies cited as evidence for expression of the nAChR at the brain microvascular endothelium were only cell culture investigations [617]. IHC of brain with an antibody to the nAChR shows receptor expression in astrocytes and neurons, but not on endothelium [618,619,620]. IHC was performed on isolated rat brain microvessels with an antibody to the nAChR, but the immune staining was discontinuous suggesting the antibody was labeling abluminal elements such as remnants of astrocyte foot processes [621]. Abluminal immune staining of some microvessels was also observed in human cerebellum, and was attributed to peri-vascular astrocyte endfeet or nerve endings [622]. In contrast, there was robust expression of immunoreactive nAChR in brain parenchyma [622].

#### 8.1.8. Basigin/CD147

Basigin, also known as extracellular matrix metalloproteinase inducer, HT7, neurothelin, or CD147, is encoded by the BSG gene, and is a relatively small trans-membrane protein of 251 AA (BAC76828). A panel of MAbs against human CD147 has been produced as potential MAb BBB Trojan horses for brain drug delivery [623,624]. It is difficult to evaluate the utility of these BSG MAbs, because the only testing of BBB transport was performed with in vitro BBB models in cell culture [623,624]. As discussed in Section 6.2.2 and Section 6.2.4, BSG forms a hetero-dimer with the MCT1 lactate transporter in a manner similar to the hetero-dimer formed between the 4F2hc (CD98hc) and the LAT1 large neutral amino acid transporter. BSG facilitates the insertion of the MCT1 transporter into the plasma membrane [625]. BSG is the receptor for the malaria parasite, *Plasmodium falciparum* [626]. However, BSG does not mediate the BBB transport of *P. falciparum* [627]. It is unclear how the intra-vascular parasite triggers the CNS manifestations of cerebral malaria [628]. BSG, like 4F2hc, participates in CMT transport at the BBB, not RMT. Nevertheless, antibodies against either Bsg [623,624] or 4F2hc [430] have been proposed as BBB molecular Trojan horses. A MAb against 4F2hc was developed as a RMT candidate and the activity was said to be greater than a TfRMAb [430], although this lead was not pursued. A cryo-electron microscopy study shows the three-dimensional structure of the LAT1/4F2hc hetero-dimer bound by a Fab directed against the 4F2hc subunit [372]. However, the MCT1 and LAT1 CMT systems are trans-membrane cavities that do not undergo endocytosis into the cell. Since neither the LAT1/4F2hc complex nor the MCT1/BSG complex undergoes endocytosis, it is not clear how developing a MAb against either 4F2hc or BSG can enable transcytosis across the endothelium. One caveat in developing BSG MAbs for RMT drug delivery to brain is the species differences in BSG expression at the BBB. Proteomics studies show high expression of BSG at the rat brain capillary, 30 pmol/mg protein [409]. In contrast, BSG at the human brain capillary is undetectable [360].

#### 8.1.9. Miscellaneous Receptors

The cross-reacting material (CRM)-197 is a 58 kDa mutated, non-toxic form of the diptheria toxin (DT), which was proposed as a BBB Trojan horse [629]. The DT receptor (DTR) is the heparin binding epidermal growth factor (EGF)-like growth factor (HB-EGF). The use of CRM197 as a BBB Trojan horse assumes that the HB-EGF is expressed at the brain capillary endothelium. Early work on the IHC of brain shows expression of HB-EGF in neurons and glial cells with minimal, if any, expression in endothelium [630]. Liposomes were conjugated with CRM197, but this ligand provided no enhancement in the brain uptake of the liposomes in vivo in mice [631]. However, such in vivo studies with CRM197 should not be performed in mice, as the DTR in rats and mice is a variant. The rat DTR has low affinity for the DT or CRM197, and the mouse DTR has no affinity for DT or CRM197 [632]. Therefore, in vivo work with CRM197 must be performed in guinea pigs [629]. However, CRM197 is quite toxic in guinea pigs, as the IV administration of 50–500 µg/kg of CRM197 to guinea pigs causes BBBD and neuropathological changes to brain endothelium [633].

Iron is sequestered in the intracellular compartment by binding to ferritin (Ft) and Ft is a 24 sub-unit hetero-polymer composed of heavy chains (HFt) and light chains (LFt). In rodents, HFt, but not LFt, is endocytosed by cells via the T cell immunoglobulin and mucin domain (Tim)-2 receptor [634]. However, Tim-2 is not expressed in humans [635], although HFt binds the human Tim-1 receptor [635]. HFt also binds the human TfR1 at a site that is spatially removed from the binding site for holo-Tf [636,637,638]. Surface plasmon resonance (SPR) shows the KD of HFt binding to the human TfR1 is 7.1 ± 0.2 nM [638]. Ft is present in plasma, although most of the circulating Ft is the light chain [639]. The use of HFt as a BBB Trojan horse was evaluated with the use of iron magnetic nanoparticles [640]. HFt or LFt shells were loaded with iron oxide at 65C to form the nanoparticles (NP). The HFt-NPs, relative to the LFt-NPs, were preferentially transported across a monolayer of cultured human hCMEC/D3 endothelial cells, and preferentially taken up by rat brain vivo based on external fluorescence imaging [640]. The extent to which the brain uptake of HFt is mediated by the BBB TfR1 or BBB Tim-2 receptor in rodents is not known. There is little evidence that Tim-2 is expressed at the rodent BBB. Fluorescent microscopy of mouse brain shows expression of Tim-2 in neurons and glial cells, but not at the microvasculature [641]. The IV administration of ^59^Fe-labeled HFt to rats resulted in very high uptake in liver and spleen, moderate uptake in kidney, heart, and lung, and very low uptake by brain [642]. The brain uptake of ^59^Fe-HFt was 100-fold lower than the uptake by liver or spleen [642].

The TCblR (CD320) mediates brain uptake of the vitamin B12/cobalamin complex (Table 3 and Section 6.2.7). The mRNA encoding CD320 is enriched at the brain capillary [430]. However, a MAb against CD320 was not taken up by brain following IV administration [430]. It is possible other MAbs may undergo transport across the BBB via RMT on CD320, as not all receptor-specific antibodies are endocytosing antibodies. A panel of MAbs against human CD320 was prepared, and most antibodies did not inhibit cell uptake of the TCblR [643], and would be potential candidates for RMT across the BBB via CD320. However, some anti-CD320 antibodies inhibit the endothelial transcytosis of the B12/TC complex [644], and would not be suitable candidates for RMT delivery.

### 8.2. Trojan Horse Delivery via Blood–Brain Barrier Receptor-Mediated Transport (RMT)

#### 8.2.1. Peptide-Based RMT Trojan Horses

**Insulin receptor peptides.** Insulin was the first BBB Trojan horse developed. As described in a U.S. patent issued in 1989 [645], a neuropeptide, somatostatin, which does not cross the BBB, was covalently conjugated to insulin, a ligand for the BBB insulin receptor, which resulted in enhanced uptake of somatostatin by isolated brain capillaries. Insulin is composed of two disulfide linked chains, as discussed in Section 8.1.1. This dual chain structure is not amenable to fusion protein technology. The A and B chains of insulin were connected by a dodecapeptide linker, which converted insulin into a single chain [646]. The single chain form of insulin was genetically fused to albumin to form an insulin–albumin fusion protein, albondin. The insulin domain of albondin retained high affinity binding to the insulin receptor. The ED50 of binding to the HIR was 1.1 nM and 7.4 nM for insulin and albondin, respectively. This technology could be replicated for BBB delivery by fusion of the single chain form of insulin to another biologic that does not cross the BBB. A potential problem with such a fusion protein is that the insulin domain of the fusion protein would bind the insulin receptor in peripheral tissues, which may cause hypoglycemia.

**Transferrin receptor peptides.** Transferrin (Tf) has been used as a Trojan horse for nanoparticle delivery across the BBB, as discussed below in Section 9.5.3. The problem with using Tf as a Trojan horse is the exogenous Tf Trojan horse must compete with the endogenous holo-Tf in plasma for binding to the BBB TfR. The concentration of holo-Tf in plasma, 25,000, is nearly 1000-fold higher than the concentration of the TfR1 at the BBB [559]. Therefore, the BBB TfR1 is >99.9% saturated with endogenous holo-Tf [647]. Tf was conjugated to lysozyme in an effort to deliver this enzyme across the BBB, although no testing of the Tf-enzyme conjugate in vivo was reported [648]. Tf-mimetic peptides that bind the TfR at a site spatially removed from the Tf binding site have been developed [649]. One such peptide, designated 2DS25, bound to the human TfR1 ECD with a KD of 20 nM. However, the BBB transportability of the peptide was only tested in an in vitro model [649]. An alternative strategy to the development of Tf-mimetic peptides is the discovery of cysteine rich peptides (CDP) that have an affinity for the TfR [650]. Databases were searched for peptides of 30–50 Aas in length with 6–10 cysteine residues. Lead candidates, of 6 kDa in size, and designated TfRB1G2 and TfRB1G3, had a KD of binding to the TfR1 of 8.7 and 0.22 nM, respectively. After IV injection in the mouse, the CDP was cleared by organs with liver > kidney > spleen >> brain. The brain uptake of the CDP was 100-fold lower than the liver uptake, and measurable uptake by brain was not observed with whole body autoradiography [650]. Nevertheless, the study concluded that the low brain uptake was pharmacologically significant, and a fusion protein of the 13-AA neuropeptide, neurotensin, and the CDP was observed to activate a cyclic AMP response element in brain following IV administration of 100 nmol/mouse [650]. This is a relatively large injection dose (ID), and is equal to an ID of 24 mg/kg of the 6 kDa CDP.

**LRP1 peptides.** LRP1 binds multiple peptides, at different domains of the receptor [651], and LRP1 ligands have been proposed as a peptide-based BBB Trojan horse [652,653]. Melanotransferrin (MTf) was said to cross the BBB via LRP1 based on an in vitro BBB model [601]. However, subsequent in vivo work showed that MTf does not cross the BBB [654,655]. Angiopep-2, a 19-AA cationic peptide, was said to cross the BBB via LRP1 based on an in vitro BBB model [602]. However, the angiopep-2 peptide has little to no affinity for LRP1. The ECD of LRP1 is composed of four domains, I, II, III, and IV. The KD of angiopep-2 binding to domains II or IV was >1000 nM [656]. In another study, binding of angiopep-2 to domains II or IV was not detectable [657]. Angiopep-2 failed to increase brain uptake of either a lysosomal enzyme [522] or liposomes [631]. Lactoferrin (Lf) was said to cross the BBB via transport on LRP1 based on a cell culture model of the BBB [658]. However, when Lf transport across the BBB was measured in vivo, the brain uptake of this protein is very low in the rat, 0.016%ID/g brain [659], which is a level of brain uptake expected for a protein trapped in the brain plasma volume. In contrast, the brain uptake of the OX26 TfRMAb in the rat after IV administration is 0.44 ± 0.02%ID/g [660]. Another ligand of LRP1 is receptor associated protein (RAP), which was said to cross the BBB following IV administration of RAP labeled with ^125^I and chloramine T [654]. Based on the hypothesis that LRP1 was a RMT system expressed at the BBB, and that RAP was an endogenous ligand for this receptor, RAP-lysosomal enzyme fusion proteins were engineered for RMT delivery across the BBB [661]. The fusion proteins were only validated by cell culture models, and there was no subsequent development of the RAP-lysosomal enzyme fusion proteins. Another ligand of LRP1 is apolipoprotein E (ApoE), a 34 kDa protein associated with lipoproteins. In an effort to develop apoE peptidomimetics that are bound by LRP1, certain domains of apoE were synthesized as 15–20-AA peptides. Using an AA numbering system that does not include the signal peptide, ApoE(130–149) and ApoE(141–155) were synthesized [662]. ApoE(130–149) bound to domains II and IV of LRP1 with a KD of 51 and 129 nM, respectively; ApoE(141–155) bound to domains II and IV of LRP1 with a KD of 118 and 190 nM, respectively [662]. Both ApoE(130–149) and ApoE(141–155) are strongly cationic peptides and most likely enter cells via absorptive-mediated endocytosis, and not by RMT on LRP1. A peptide named COG-133 corresponds to ApoE(133–149) [631]. Liposomes were targeted with angiopep-2, COG-133, CRM197, and the RI7–217 TfRMAb, but only the TfRMAb Trojan horse mediated delivery to brain in vivo in the mouse [631]. The RI7-217 MAb is a rat antibody against the mouse TfR1, and is actively taken up by mouse brain in vivo [663]. The failure of the LRP1-targeted peptide Trojan horses to effectively deliver cargo to brain is consistent with the absence of expression of LRP1 on the endothelial luminal membrane (Figure 11B), as discussed in Section 8.1.5.

**LDLR peptides.** Apolipoprotein B100 (ApoB) binds the LDLR to trigger endocytosis of LDL into cells, and the LDLR is said to function at the BBB, based on cell culture studies [610]. To develop a peptide-based Trojan horse targeting the LDLR, a phage peptide library was screened for candidates [664], which led to the development of an 8-AA peptide, CMPRLRGC, designated VH434, that binds the LDLR with low affinity and a KD of 196 nM [665]. This peptide was fused to the carboxyl terminus of a human IgG1 Fc and the VH434-Fc injected intravenously at 8 mg/kg in either wild-type or ldlr^-/-^ knockout mice [665]. The brain/plasma ratio at 24 h was 2.2% and 1.1% in the wild-type and ldlr^-/-^ mice, respectively. However, the brain plasma volume is 10–30 µL/g [666], which is 1–4% of the brain volume. Any Fc-peptide conjugate confined to the blood volume of brain has not crossed the BBB. As discussed in BBB Methods (Section 11.4.4), it is important to correct brain uptake, especially for biologics, for the brain plasma volume. The primary lipoprotein that binds the LDLR is apolipoprotein B-100 (ApoB), which is a 500 kDa 4536-AA protein not counting the 27-AA signal peptide (NP_000375). An LDLR binding domain lies at AA 3371–3409, and this sequence was fused to the carboxyl terminus of a lysosomal enzyme, and this fusion gene was incorporated in a lentivirus transfection vector [667]. Subsequently, this apoB-mimetic peptide was fused to the amino terminus of secretory neprilysin, an endopeptidase that degrades the Abeta amyloid peptide of AD, and this fusion protein is designated ASN12 [668]. The ASN12 fusion protein was injected intravenously at a dose of 1 mg/kg in the mouse and the brain fusion protein was measured by ELISA. The brain fusion protein concentration at 24 h was 210 ng/g [668], which is equal to a brain concentration of 0.3%ID/g. It is difficult to attribute this low level of brain uptake to RMT via the LDLR at the BBB, since IHC shows the LDLR is not expressed at the brain microvasculature in vivo [562]. The sequence of the apoB-mimetic peptide domain of the ASN12 fusion protein is SSVIDALQYKLEGTTRLTRKRGLKLATALSLSNKFVEGS [669]. This is a highly cationic peptide with a pI of 10.2. It is likely that any BBB penetration that is achieved with this peptide is via absorptive-mediated endocytosis of a cationic peptide, as discussed in Section 7.1.

**Glutathione.** Glutathione (GSH) is a tripeptide that is said to cross the BBB to mediate the brain uptake of pegylated liposomes conjugated with GSH [670]. The basis for the use of GSH as a BBB Trojan horse is early work describing the enhanced uptake of GSH by frog oocytes injected with RNA isolated from SV40 transformed mouse brain endothelial cells in culture [671]. The presumptive sodium dependent GSH transporter was never identified. GSH is a low affinity ligand for the sodium dependent dicarboxylic acid transporter [672], but dicarboxylic acids do not cross the BBB [327]. GSH is a ligand for the N-methyl D-aspartate (NMDA) receptor (NMDAR) [673,674]. The NMDAR was localized to the brain microvasculature using a monoclonal antibody designated Glunomab [675]. This antibody was raised against synthetic peptides corresponding to the amino terminal domain of the GluN1 subunit of the NMDAR, which is a hetero-trimeric membrane protein. Fluorescent microscopy of the Glunomab immunoreactivity in brain microvessels is discontinuous [675], which is consistent with expression in either endothelium or astrocyte endfeet. The absence of the NMDAR at the brain endothelium is supported by several studies showing that brain endothelial cells lack a functional NMDAR [676]. Irrespective of what transporter or receptor GSH might access, early work on GSH transport at the BBB shows this tripeptide does not cross the BBB [677], as recently reviewed [678]. The lack of BBB transport of the GSH tripeptide is similar to the absence of BBB transport of another tripeptide, thyrotropin releasing hormone [677].

**Phage peptides.** In 1996, phage display libraries were first used to isolate peptides that bind the luminal membrane of the brain capillary endothelium [679]. Phage libraries encoding random CX_7_C octapeptide sequences were injected intravenously in mice and the brain harvested for phage recovery. After three rounds, a single phage was identified of known AA sequence. Subsequent use of the technology identified peptides that overlapped with domains of human Tf [680]. An f3 phage library with random 15-mer sequences were infused in the carotid artery of mice, which resulted in identification of a 15-AA peptide, designated the GLA peptide [681]. The GLA was conjugated to pegylated liposomes for delivery to cultured hCMEC/D3 endothelial cells, but uptake was low [682]. It was reasoned that the conformation of the 15-mer peptide differed from the conformation adopted by this sequence within the p3 phage coat protein. The 15-mer sequence was incorporated in a peptide encompassing 240 AA of the amino terminal domain of the p3 coat protein, and this new peptide was designated, p3-GLA [682]. Pegylated liposomes were targeted with either the GLA peptide or the p3-GLA peptide and incubated with cultured hCMEC/D3 endothelial cells followed by fluorescence-activated cell sorting. Liposomes targeted with the GLA peptide were not bound to the cells, whereas binding was detected with the p3-GLA peptide. It has been over 25 years since peptide phage display methods have been used to identify peptides that target the BBB. The problems with this approach are (a) the receptor targeted by the peptide is generally not known, (b) the BBB binding site identified has to be a RMT system, (c) the synthetic peptide may not have the same binding activity as the peptide sequence presented as part of the phage coat protein, and (d) oligopeptides invariably have low affinity for the targeted receptor.

In summary, peptide-based RMT Trojan horses typically work well in vitro in cell culture models, but are difficult to translate to in vivo brain delivery. The peptide may have minimal activity in vivo owing to competition with the endogenous peptide ligand, or have a poor plasma pharmacokinetics profile owing to rapid clearance by peripheral tissues. Many RMT-based peptides used for brain drug delivery are highly cationic peptides. Cationic peptides are toxic with poor safety profiles, as reviewed in Section 7.3.1. In several instances, peptides that target a presumptive BBB RMT system, such as LRP1, LDLR, nAChR or NMDAR, are ligands for receptors that are not expressed on the brain endothelium, as depicted in Figure 11B.

#### 8.2.2. Monoclonal Antibody-Based RMT Trojan Horses

Work in the early 1980s, using either isolated brain capillaries and radio-receptor assays [560], or immunohistochemistry [577], showed that the IR or TfR was highly expressed at the brain microvascular endothelium. By 1985, experiments with human brain capillaries showed the BBB IR mediated the endocytosis and exocytosis of insulin at the BBB [561]. This led to the chimeric peptide hypothesis, wherein a peptide drug, which normally does not cross the BBB, could be linked to a ligand that normally undergoes RMT across the BBB [58]. By 1987, the RMT of insulin and Tf across the BBB via the IR and TfR, respectively, was demonstrated in vivo [564,578]. In addition to endogenous ligands, early work with the LDLR showed that a monoclonal antibody (MAb) that bound an exofacial epitope on the receptor could also be endocytosed into the cell via receptor-mediated endocytosis [683]. Monoclonal antibodies (MAb) were then shown to undergo RMT across the BBB via binding to either the rat TfR [59,60] or primate IR [61]. The hypothesis that biologics could have pharmaceutical effects in the brain following linkage of the biologic to a BBB RMT ligand [58], was confirmed by in vivo pharmacologic studies using the OX26 TfRMAb [684,685]. In one model, vasoactive intestinal peptide (VIP) was conjugated to the OX26 TfRMAb with an avidin–biotin linker [684]. VIP is a potent cerebral vasodilator when applied topically to brain surface vessels. The carotid arterial infusion of VIP had no effect on cerebral blood flow (CBF) [684], because VIP does not cross the BBB. However, infusion of the OX26-VIP conjugate resulted in a 65% increase in CBF in the parenchyma of brain, whereas there was no change in CBF following the infusion of VIP alone or OX26 alone [684]. In another model, nerve growth factor (NGF) was chemically conjugated to the OX26 MAb and exerted trophic effects in an extra-cranial anterior eye transplant model [685]. The 83–14 MAb against the human insulin receptor (HIR) cross-reacted with the IR of Old World primates such as the Rhesus monkey, and was rapidly transported across the primate BBB in vivo as the brain uptake of the HIRMAb was 2.5–3.8%ID/brain [61]. The use of the HIRMAb as a BBB Trojan horse was initially tested in Rhesus monkeys with the goal of developing a BBB-penetrating peptide radiopharmaceutical for imaging the brain amyloid of AD. The Aβ^1–40^ amyloid peptide is a potential peptide radiopharmaceutical for imaging amyloid content in brain of AD [686], but the Aβ peptide does not cross the BBB [687]. [^125^I]-biotinyl Aβ^1–40^ was conjugated to the HIRMAb with a streptavidin linker [688]. Both the unconjugated [^125^I]-biotinyl Aβ^1–40^ and the HIRMAb conjugated [^125^I]-biotinyl Aβ^1–40^ avidly bound the amyloid plaques in autopsy AD brain [688]. Following IV injection of the unconjugated [^125^I]-biotinyl Aβ^1–40^ alone, no brain uptake could be detected by ex vivo brain imaging of the primate brain. In contrast, high resolution brain scans were observed following IV administration of the [^125^I]-biotinyl Aβ^1–40^ conjugated to the HIRMAb [688]. Brain radioactivity declined with a T_1/2_ of 16 h in the primate [688].

The OX26 TfRMAb is a species-specific antibody for the rat TfR and does not recognize the mouse TfR [663] or the human TfR [689]. The TfR in the mouse can be targeted with the 8D3 antibody, which is a rat MAb against the mouse TfR [690], or the RI7-217 antibody, which is a rat MAb against the mouse TfR [691]. Both the 8D3 and the RI7-217 antibodies are taken up by brain at a level of 1.6–3.1%ID/g following IV administration in the mouse [663].

Biologics can be delivered to brain following the genetic fusion of the biologic and the MAb targeting an RMT system on the BBB. The engineering and expression of recombinant forms of the antibodies targeting the TfR or IR were enabled by the determination of the AA sequence of the variable region of the heavy chain (VH) and the variable region of the light chain (VL) for the OX26 TfRMAb [692], the 8D3 TfRMAb [693], and the 83-14 HIRMAb [694]. The availability of these sequences allows for the genetic engineering of TfRMAb or HIRMAb fusion protein, as reviewed in the next section.

**Valency of TfRMAbs.** The valency of the TfRMAb domain of the fusion protein has been both bivalent and monovalent. The first monovalent TfRMAb engineered was part of a bispecific antibody (BSA) composed of one monovalent arm as the TfRMAb domain, and another monovalent arm targeting the beta secretase 1 as the therapeutic domain [695]. Engineering the TfRMAb domain in a monovalent form was obligatory since the BSA was engineered with a knob-in-hole technology [695]. In contrast, the first BBB-penetrating BSA engineered was a tetravalent BSA, where both the transporter domain targeting the HIR was bivalent, and the therapeutic domain targeting the Abeta amyloid of AD was bivalent [696]. The tetravalent BSA was engineered by fusion of a single chain form of the first antibody to the carboxyl terminus of each heavy chain of the second antibody [696]. Engineering a BSA where both the therapeutic antibody domain and the transporter antibody domain are bivalent allows for retention of high avidity binding of the BSA at both the BBB receptor and the therapeutic antibody target in brain. Conversely, in the monovalent BSA, the monovalent TfRMAb had the expected reduced binding affinity for the TfR as compared to the bivalent TfRMAb [695]. This reduced affinity was then considered advantageous and gave rise to the hypothesis that low affinity TfRMAbs were preferred BBB Trojan horses. The basis for this hypothesis was the observation that the uptake of the low affinity TfRMAb by brain was higher following the IV administration of a very high injection dose (ID) of 20–50 mg/kg of the TfRMAb. This high ID selectively saturates binding of the high affinity TfRMAb at the BBB, while not affecting binding of the low affinity TfRMAb. In another monovalent format of the TfRMAb domain of a BSA, a single chain TfRMAb was fused to the carboxyl terminus of a bivalent anti-Abeta antibody using the knob-in-hole technology [697]. In this design of the BSA, the high-affinity bivalent structure of the therapeutic antibody is retained, whereas the transporter antibody is engineered as a moderate affinity monovalent antibody. The rationale for engineering the TfRMAb domain of the BSA in a monovalent format was that the bivalent TfRMAb would trigger dimerization of the TfR, which would redirect the receptor to the lysosome resulting in reduced expression of the TfR at the cell membrane [697]. This hypothesis is curious since the TfR1 normally exists as a dimer (Figure 10C). If chronic administration of a TfRMAb led to down-regulation of the BBB TfR, then the rate of brain clearance of the antibody, as reflected by the permeability–surface area (PS) product, would be decreased after chronic treatment. This is not observed. Mice were chronically treated for 12 weeks with 2 mg/kg IV twice weekly with a fusion protein of the chimeric form of the 8D3 TfRMAb and glial-derived neurotrophic factor (GDNF), designated cTfRMAb–GDNF [698]. The BBB PS product of the cTfRMAb–GDNF fusion protein was unchanged from the start of treatment to the end of 12 weeks of treatment [698]. In addition, the rate of plasma clearance of the fusion protein was unchanged with 12 weeks of treatment [698], which indicates the TfR is not down regulated in peripheral tissues. In yet another monovalent format of a TfRMAb, the CH3 region of the antibody heavy chain was engineered by mutagenesis of multiple amino acids to create a new TfR binding site in the CH3 region [699]. A lysosomal enzyme was fused to the amino terminus of a second antibody chain, and the two chains were combined by knob-in-hole technology [699]. The monovalent format of the TfRMAb was said to be advantageous so as to avoid intracellular clustering and degradation of the TfR, which is observed following exposure of cultured hematopoietic cells to a bivalent TfRMAb [700,701]. However, these cell culture studies purporting to show intracellular clustering of a bivalent TfRMAb did not use a bivalent TfRMAb, per se, but rather a bivalent TfRMAb–avidin fusion protein. Such IgG–avidin fusion proteins form 400 kDa tetramers, owing to the dimerization of the avidin domain [700,702]. The apoptosis induced by the tetrameric TfRMAb–avidin fusion protein was not observed with the bivalent TfRMAb alone [700]. Chronic administration to mice with a high affinity bivalent TfRMAb causes no down-regulation of brain TfR or brain iron [703].

Apart from antibodies that target the TfR or IR, antibodies that target other BBB RMT systems, such as the LEPR or IGFR (Figure 11B), are also potential new BBB MAb-based Trojan horses. Recently, single domain VHH antibodies against the ECD of the human IGF1R were isolated following Llama immunization [704]. The VHH was fused to mouse Fc to generate either a monovalent or a bivalent format. Affinity for the human IGF1R was determined by SPR and the KD values ranged from 0.3 nM to 1.3 nM. BBB transcytosis was measured with an in vitro culture model as the primary model, although transcytosis was confirmed with carotid artery infusion and capillary depletion [704]. The IGF1R antibody cross reacted with the antibody in rats and mice and IV administration of the bivalent form of the antibody showed distribution into both CSF and post-vascular brain [704]. Leptin receptor (LEPR) antibodies are yet to be tested as BBB RMT agents. A panel of antibodies against the human LRPR ECD was isolated by panning a single chain Fv (ScFv) phage library with the human LEPR ECD [705]. For isolation of a LEPR antibody that does not inhibit leptin binding to the LEPR, panning of phage libraries can be performed with complexes of the LEPR ECD and leptin so as to eliminate capture of antibodies that bind the leptin binding site on the LEPR.

In summary, receptor-specific MAbs are more effective BBB RMT Trojan horses than are peptides. Virtually any research lab can custom order their own lot of recombinant 8D3 TfRMAb for brain delivery in mice, recombinant OX26 TfRMAb for brain delivery in rats, or recombinant 83-14 HIRMAb for brain delivery in Old World primates or human cells, because the sequences of the VH and VL for these antibodies have been published [692,693,694]. The production of recombinant antibodies based on these sequences has recently been described for a recombinant 8D3 TfRMAb [706] or recombinant 83-14 HIRMAb [707].

### 8.3. IgG Fusion Proteins for Blood–Brain Delivery of Biologics

**Figure 12:**
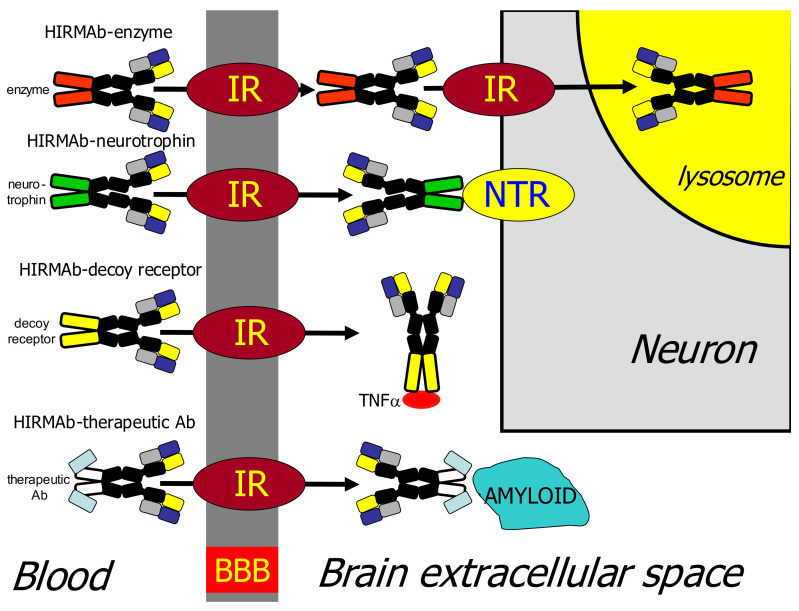
**BBB receptor-mediated transport of IgG fusion proteins of lysosomal enzymes, neurotrophins, decoy receptors, and therapeutic antibodies.** Model of RMT of 4 classes of biologics (lysosomal enzyme, neurotrophin, decoy receptor, or therapeutic antibody) across the BBB following fusion of the biologic to a BBB Trojan horse such as the HIRMAb. The IgG domain of the fusion protein targets the insulin receptor (IR) on the BBB and, if necessary, as in the case of lysosomal enzymes, on the brain cell membrane. In the examples depicted in this figure, the therapeutic domain is fused to the carboxyl terminus of each heavy chain of the HIRMAb. Reproduced with permission from [708], Copyright© 2015 John Wiley & Sons.

All four classes of biologics have been reduced to practice as either TfRMAb and HIRMAb fusion proteins, including therapeutic antibodies, lysosomal enzymes, neurotrophins, and decoy receptors [708]. In the case of delivery of a therapeutic antibody to brain, the problem is engineering of a bispecific antibody (BSA), which includes a transporter antibody domain and a therapeutic antibody domain. There are multiple approaches to the genetic engineering of BBB-penetrating BSAs, as discussed in Section 8.3.4. In the case of brain delivery of a lysosomal enzyme, it is necessary to deliver the enzyme across both the BBB and the brain cell membrane, followed by triage of the IgG-lysosomal enzyme fusion protein to the lysosomal compartment [709]. A lysosomal enzyme can be delivered across both the BBB and the brain cell membrane (BCM) with an antibody targeting the IR, TfR, LEPR, or IGFR, as these receptors are expressed on both the BBB and the BCM, as depicted in Figure 11B. The engineering of bi-functional HIRMAb or TfRMAb lysosomal enzyme fusion proteins is discussed in Section 8.3.1. In the case of neurotrophin delivery to brain, the neurotrophin receptor (NTR) is generally expressed on the plasma membrane of brain cells, so the IgG–neurotrophin fusion protein need only traverse the BBB to access the target neurotrophin receptor in brain. The engineering of bi-functional HIRMAb or TfRMAb neurotrophin fusion proteins is discussed in Section 8.3.2. In the case of decoy receptor delivery to brain, the cytokine target of the decoy receptor is generally secreted to the extracellular space of brain, so the IgG-decoy receptor need only cross the BBB to come in contact with the target inflammatory cytokine. The engineering of bi-functional HIRMAb or TfRMAb decoy receptor fusion proteins is discussed in Section 8.3.3. The delivery of therapeutic antibodies, lysosomal enzymes, neurotrophins, or decoy receptors to brain with an HIRMAb Trojan horse is depicted in Figure 12.

#### 8.3.1. Lysosomal Enzymes

There are over 70 lysosomal storage diseases and over 50 of these affect the CNS [710,711]. The principal treatment for these conditions is Enzyme Replacement Therapy (ERT) with weekly IV infusions of the recombinant enzyme. However, ERT does not treat the brain because the enzymes do not cross the BBB, owing to the absence of the cation independent mannose-6 phosphate (M6P) receptor (M6PR) on the BBB [709]. Mucopolysaccharidosis (MPS) Type I (MPSI), also called Hurler syndrome, is caused by mutations in the gene encoding the lysosomal enzyme, α-L-iduronidase (IDUA). The cDNA encoding human IDUA was cloned [712], and Chinese hamster ovary (CHO) lines expressing recombinant IDUA [713] were developed over 30 years ago. However, in 2022, the primary treatment of infants <16 months of age diagnosed with neuronopathic MPSI, also called Hurler syndrome, is stem cell transplant [291]. The problem with stem cell transplant as a treatment of the brain, as discussed in Section 5.1, is that stem cells do not cross the BBB.

**Figure 13:**
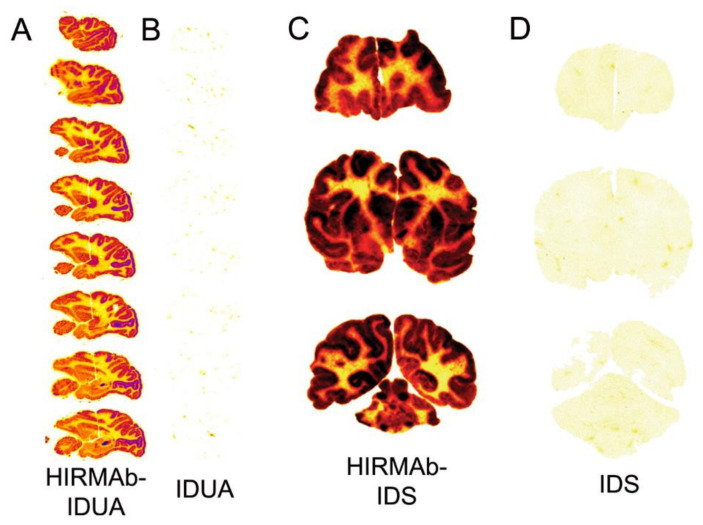
**Brain delivery in the primate of lysosomal enzymes fused to the HIRMAb.** Phosphorimager scans of sagittal sections of Rhesus monkey brain removed 2 h after the IV administration of [^125^I]-Bolton–Hunter labeled HIRMAb–IDUA fusion protein (**A**) or IDUA alone (**B**). Reproduced with permission from [716], Copyright© 2017 American Chemical Society. Film autoradiograms of coronal sections of Rhesus monkey brain removed 2 h after the IV administration of [^125^I]-Bolton–Hunter labeled HIRMAb-IDS fusion protein (**C**) or IDS alone (**D**). Reproduced with permission from [717], Copyright© 2013 American Chemical Society.

In an effort to develop a treatment of the brain in MPSI, the IDUA lysosomal enzyme was re-engineered to enable BBB transport via RMT on the brain capillary insulin receptor. A fusion protein of human IDUA and the chimeric HIRMAb was engineered, wherein the IDUA enzyme, minus the enzyme signal peptide, was genetically fused to the carboxyl terminus of each heavy chain of the chimeric HIRMAb [714]. The structure of the HIRMAb–IDUA fusion protein, now named valanafusp alfa [715], is shown in Figure 12. The HIRMAb–IDUA fusion protein retained high affinity binding to the HIR and high IDUA enzyme activity [714]. The fusion protein was triaged to the lysosomal compartment of Hurler fibroblasts based on confocal microscopy and co-localization of the fusion protein with lysosomal associated membrane protein-1, a lysosome marker [714]. Treatment of Hurler fibroblasts with the HIRMAb–IDUA fusion protein normalized intracellular IDUA enzyme activity, and caused a decrease in the intracellular content of sulfated glycsoaminoglycans [714]. The impact on brain uptake of fusion of IDUA to the BBB-penetrating HIRMAb was examined in the adult Rhesus monkey. The recombinant IDUA (laronidase) and the HIRMAb–IDUA fusion protein (valanafusp alfa) were separately radio-iodinated with the [^125^I]-Bolton–Hunter reagent, and injected intravenously into separate Rhesus monkeys. The plasma concentration of each protein was determined over a 2 h period, followed by euthanasia of the primates, freezing, and determination of the organ distribution of each protein by whole body imaging of radioactivity using a phosphorimager. The brain was processed by sagittal sectioning. The impact of the IDUA fusion to the HIRMAb on the brain uptake of the lysosomal enzyme is shown in Figure 13A,B.

These ex vivo brain scans show (a) that IDUA does not cross the BBB (Figure 13B), and (b) that global distribution of IDUA to brain is possible after fusion of the enzyme to the HIRMAb BBB Trojan horse (Figure 13A). HIRMAb–IDUA and IDUA were radio-iodinated with the [^125^I]-Bolton–Hunter reagent [716]. This is the preferred method of radiolabeling biologics for the study of BBB transport in vivo, because Bolton–Hunter radiolabeled metabolites do not cross the BBB [717], as discussed in Methods, Section 11.4.4.

The pharmacologic efficacy of Trojan horse–IDUA fusion proteins was tested in the MPSI Hurler mouse. The HIRMAb cannot be tested in the mouse, because the HIRMAb does not recognize the murine insulin receptor [709]. Therefore, for treatment of the brain of the Hurler mouse, the murine IDUA was fused to the carboxyl terminus of each heavy chain of the recombinant 8D3 TfRMAb [718]. Aged MPSI mice were treated intravenously with 1 mg/kg of the TfRMAb–IDUA fusion protein weekly for 8 weeks. This treatment reduced lysosomal inclusion bodies in brain by 73% [718]. The safety pharmacology of chronic treatment with an IgG–IDUA fusion protein was evaluated in primates. Chronic treatment of Rhesus monkeys with weekly IV infusions of 3, 9, or 30 mg/kg of the HIRMAb–IDUA fusion protein for 6 months showed the only adverse effect was hypoglycemia following rapid IV infusion of the high dose, 30 mg/kg, of the fusion protein in saline [709]. The hypoglycemia was eliminated by the addition of 5% dextrose to the infusion solution [709]. Chronic treatment of primates had no effect on glycemic control [709]. The HIRMAb–IDUA fusion protein was the first BBB Trojan horse pharmaceutical to enter human clinical trials, and a 1-year phase I-II trial was performed in children with MPSI [715]. Treatment stabilized the decline in cognitive impairment and cerebral atrophy. Over the course of 1 year, >500 IV infusions of the fusion protein were administered, and adverse events included a 1.7% incidence of infusion related reactions, treated with diphenhydramine, and a 2.1% incidence of mild hypoglycemia reversed with a snack [715]. The formation of anti-drug antibodies (ADA) against the valanafusp alfa was comparable to the ADA response to recombinant IDUA, laronidase [715].

In addition to IDUA, eight other lysosomal enzymes have been re-engineered for BBB transport by enzyme fusion to the HIRMAb or the TfRMAb, as recently reviewed [709], and these are listed in Table 4.

In the case of the nine IgG-lysosomal enzyme fusion proteins listed in Table 4, high affinity binding of the fusion protein to the insulin or transferrin receptor and high lysosomal enzyme activity was retained.

MPSII, also known as Hunter syndrome, is caused by mutations in the gene encoding the lysosomal enzyme, iduronate 2-sulfatase (IDS). The cDNA encoding human IDS was cloned in 1990 [745], and recombinant IDS was produced in HT-1080 fibrosarcoma cells for clinical trials [746]. About two-thirds of MPSII subjects have CNS disease [747], and ERT with recombinant IDS does not treat the brain because the enzyme does not cross the BBB. Human IDS was re-engineered for BBB transport by fusion of the enzyme to the carboxyl terminus of each heavy chain of the HIRMAb [722] (Table 4). The HIRMAb–IDS fusion protein retained high affinity binding to the human insulin receptor and high IDS enzyme activity [722]. The impact of fusion of the IDS to the HIRMAb BBB delivery agent is demonstrated in Figure 13, which shows the film autoradiograms of coronal sections of Rhesus monkey brain removed 2 h after the IV injection of either the HIRMAb–IDS fusion protein (Figure 13C), or IDS alone (Figure 13D). The HIRMAb–IDS and IDS were radio-iodinated with either the [^125^I]-Bolton–Hunter reagent or with 125-iodine and Iodogen for comparison of these different protein radio-labeling methods [717]. The data showed that low-MW metabolites formed by the degradation of the fusion protein in peripheral tissues, and released to blood, do not cross the BBB and enter brain when the protein is labeled with the [^125^I]-Bolton–Hunter reagent, whereas such metabolites do enter brain following Iodogen radiolabeling [717]. Labeling with the [^125^I]-Bolton–Hunter reagent is a non-oxidative process that conjugates the Bolton–Hunter reagent to ε-amino groups of surface lysine residues [748,749], and this modified lysine does not cross the BBB [717]. Labeling with 125-iodine and Iodogen is an oxidative process that places the iodine atom on tyrosine residues, and the iodo-tyrosine does cross the BBB [717]. To eliminate artifacts of brain uptake caused by peripheral metabolism, biologics should be iodinated with the Bolton–Hunter reagent or chelation of 111-indium, as discussed in Methods, Section 11.4.4. The safety pharmacology of chronic treatment with the HIRMAb–IDS fusion protein was evaluated in primates, and no adverse events were observed in a 6-month chronic GLP dosing study in 42 juvenile Rhesus monkeys [721].

The human IDS enzyme has also been fused to a TfRMAb that targets the human TfR1 [719]. This TfRMAb-IDS fusion protein, now designated pabinafusp alfa, has completed a successful phase 3 clinical trial [720]. As discussed in Section 13, pabinafusp alfa (IZCARGO^®®^) is the first BBB Trojan horse fusion protein to receive market approval, as this TfRMAb–IDS fusion protein was approved in 2021 by the Ministry of Health, Labor, and Welfare (MHLW) in Japan for treatment of the brain in MPS-II.

#### 8.3.2. Neurotrophins

Nerve growth factor (NGF) was discovered in 1951 [750]. Over the next 40 years, more than 30 neurotrophic factors were identified as potential new treatments of CNS disease [751], and in 1994, *Science* magazine hailed the entry of neurotrophic factors into clinical trials for CNS disorders, although the issue of how neurotrophins crossed the BBB was not discussed [752]. Nearly 30 years later, in 2022, and 70 years after the NGF discovery, there is not a single neurotrophin that is FDA approved for CNS disease. This lack of neurotophin approval is not for the lack of trying. However, neurotrophin drug development for brain proceeded down a path that either ignored or avoided the BBB. The first neurotrophin clinical trials tested the effects of either brain-derived neurotrophic factor (BDNF) or ciliary neurotrophic factor (CNTF) for amyotrophic lateral sclerosis (ALS), wherein the neurotrophin was administered by subcutaneous (SQ) administration. The issue of BBB delivery of the neurotrophin was *in absentia*. Both trials failed, since neither neurotrophin reached the brain. In the report of the failed trial, the issue of BBB transport of the neurotrophin was not discussed for BDNF [753] or CNTF [754]. Having discovered that SQ administration of neurotrophins may not lead to clinical success in a CNS disease, the next neurotrophin to enter clinical trials, glial-derived neurotrophic factor (GDNF), was administered by ICV injection for the treatment of PD [107]. The ICV delivery route was used, even though the sponsor’s own data demonstrated very limited penetration into brain following ICV administration [79], as shown in Figure 5C. For the reasons discussed in Section 2.1.4, the failure of the ICV trial of GDNF for PD was expected. Following the failure of the ICV delivery for PD, the next clinical trial for GDNF therapy of PD used convection-enhanced diffusion (CED) for brain drug delivery [130]. The limitations of CED for brain drug delivery are reviewed in Section 2.2.2. The CED clinical trial failed for GDNF in PD [130], and subsequent to the CED trial failure, a primate study showed that GDNF entry into brain tissue following CED was very limited [131], as shown in Figure 6A, and discussed in Section 2.2.2. Some 14 years after the failed CED trial of GDNF in PD, a panel discussed the future of GDNF therapy in PD [755]. Not much had changed in 30 years, as the role of the BBB in neurotrophin drug development for brain disorders was not mentioned in either 1994 [752] or in 2020 [755]. CNS drug developers are reluctant to discuss the BBB if they have no solution to the intractable problem of brain drug delivery.

Apart from chronic disease such as ALS or PD, neurotrophins could also be potent drugs for acute brain disease, such as acute stroke. Neurotrophins can rescue dying neurons if the neuroprotective agent is administered to the ischemic brain within 5 h after the stroke event [756,757]. Therefore, the neurotrophin stroke trials were designed to administer the neurotrophin by IV injection within 5 h of the stroke. The problem with this approach is that the BBB is intact in the early hours after acute cerebral ischemia in either experimental stroke [758,759] or human stroke [757,760]. Since no attempt was made to re-engineer the neurotrophin for BBB delivery, the trials met with the expected failed results for either the intravenous fibroblast growth factor (FGF)-2 stroke trial [761] or the intravenous erythropoietin (EPO) stroke trial [762]. The rationale for the EPO trial was that the IV administration of EPO results in EPO delivery to CSF, which was taken as evidence that EPO crosses the BBB [763]. However, EPO does not cross the BBB [733]. As discussed in Section 1.1, drug entry into CSF is evidence for drug transfer across the choroid plexus, which forms the blood–CSF barrier, but is not evidence for drug transfer across the brain endothelium, which forms the BBB (Figure 3). The use of biologic entry into CSF as confirmation of BBB transport, and as a rationale for a CNS clinical trial, leads predictably to clinical trial failure [762]. An approach to CNS drug development that relies on drug entry into CSF as a measure of BBB transport is reminiscent of concepts from over 100 years ago. As reviewed in Section 1.2, the prevailing view in 1913 was that CSF is an obligatory compartment between blood and brain [26].

Working on the hypothesis that neurotrophin drug development requires re-engineering of the neurotrophin for BBB transport, BDNF was conjugated to the OX26 TfRMAb. To optimize plasma pharmacokinetics, the cationic BDNF was pegylated on carboxyl groups [764]. The PEG-BDNF-TfRMAb conjugate was neuroprotective in both transient forebrain ischemia [765] and middle cerebral artery occlusion (MCAO) [766] following delayed IV administration. The reduction in stroke volume in the MCAO model correlated with a functional motor improvement using the rotarod test [767]. A BDNF–HIRMAb fusion protein was engineered, which retained high affinity binding to both the BDNF trkB receptor and the HIR [728]. A HIRMAb–GDNF fusion protein was engineered, which retained high affinity binding for the GDNF receptor and the HIR [730]. For preclinical studies in a mouse PD model, a TfRMAb–GDNF fusion protein was engineered [768], and this fusion protein was neuroprotective in both experimental PD [731], and experimental stroke following delayed IV administration in the mouse [732]. The HIRMAb-GDNF produced no adverse events in a GLP toxicology evaluation in 56 Rhesus monkeys that were administered up to 50 mg/kg of the fusion protein over 60 h [729].

A HIRMAb–EPO fusion protein was engineered that retained high affinity binding to both the HIR and the human EPO receptor (EPOR) [733]. The brain uptake and plasma pharmacokinetics of either EPO or the HIRMAb–EPO fusion protein was measured in the Rhesus monkey following radiolabeling of either EPO or the HIRMAb–EPO fusion protein with the [^125^I]-Bolton–Hunter reagent [733]. Fusion of the EPO to the HIRMAb had 2 beneficial effects. First, fusion to the HIRMAb enabled EPO to enter the primate brain with a brain uptake of 2.1 ± 0.1%ID/brain. Conversely, the brain uptake of EPO alone was comparable to a brain plasma volume marker, an IgG1 isotype control antibody, which indicated EPO is retained in the brain plasma volume and does not cross the BBB [733]. Second, fusion of EPO to the HIRMAb resulted in a 13-fold reduction in the plasma AUC of EPO [733]. Since the hematopoietic effect of EPO is proportional to the plasma AUC [769], EPO fusion to the HIRMAb reduces the hematopoietic effect of the EPO domain by 13-fold. For preclinical studies in a mouse PD or AD model, EPO was fused to the 8D3-derived TfRMAb, and the TfRMAb–EPO fusion protein retained high affinity binding for the mouse EPOR and mouse TfR1 [770]. Chronic treatment of mice with experimental PD with the TfRMAb–EPO fusion protein was neuroprotective and had only a minor effect on hematocrit [734]. Sumbria and colleagues have demonstrated the therapeutic effects of chronic treatment of AD transgenic mice with the TfRMAb–EPO fusion protein [735].

#### 8.3.3. Decoy Receptors

The leading decoy receptor pharmaceutical, etanercept, is a biologic formed by fusion of the ECD of human TNFR2 to the amino terminus of human IgG1 Fc, as originally described in 1991 [771]. Etanercept is a biologic tumor necrosis factor (TNF)-α inhibitor (TNFI). Other widely used biologic TNFIs are MAbs including adalimumab and rituximab. The global annual revenue for adalimumab in 2019 was USD 19 billion [772], and the combined revenues for etanercept and rituximab were comparable. The biologic TNFIs are only used for systemic disease of chronic inflammation, and none of these agents are FDA approved for treatment of CNS disease. The biologic TNFIs are not approved for the brain because these agents do not cross the BBB. This is an unfortunate situation, since TNFα plays a pro-inflammatory role in both acute brain disease, such as stroke [773,774], and chronic brain disease, such as AD [775] and PD [776,777]. In experimental models of brain disease, the intra-cerebral injection of the TNFR2 ECD is neuroprotective in experimental stroke [778]. The intra-cerebral, but not the intravenous, injection of etanercept is neuroprotective in traumatic brain injury (TBI) [779]. The use of the biologic TNFIs for brain disease will require re-engineering of these biologics as BBB-penetrating drugs. In the case of adalimumab and rituximab, these therapeutic antibodies can be fused to transporting antibodies for the engineering of new bi-specific antibodies (BSA) as discussed in the next section.

A BBB-penetrating form of etanercept was engineered by fusion of the human TNFR2 ECD to the carboxyl terminus of each heavy chain of the HIRMAb [736]. The IgG-decoy receptor orientation of this HIRMAb-TNFR fusion is opposite that of etanercept. With etanercept, the TNFR2 is fused to the *amino* terminus of the IgG Fc. In contrast, with the HIRMAb–TNFR fusion protein, the TNFR2 ECD is fused to the *carboxyl* terminus of the HIRMAb. This design of the fusion protein fixes the TNFR2 in a dimeric configuration (Figure 12), which enables high affinity binding for TNFα [780]. A dimeric configuration is the preferred orientation of the TNFR2, which crystallizes as a dimer [781]. The HIRMAb–TNFR fusion protein retained high affinity binding for both the HIR and TNFα [736,782]. The HIRMAb–TNFR fusion protein and etanercept were radiolabeled with the [^125^I]-Bolton–Hunter reagent and injected intravenously in the Rhesus monkey [782]. The brain uptake of etanercept was comparable to the IgG1 isotype control, which indicates etanercept is retained in the blood volume of brain without transport across the BBB. However, the HIRMAb–TNFR fusion protein rapidly crossed the BBB with a brain uptake of 3.0 ± 0.1%ID/brain [782]. For treatment in preclinical models of stroke or PD in the mouse, an 8D3-derived TfRMAb–TNFR fusion protein was engineered and expressed, and this fusion protein retained high affinity binding to both the mouse TfR1 and TNFα [783]. The therapeutic effect of the TfRMAb–TNFR fusion protein was evaluated in experimental Parkinson’s disease (PD) induced by the intra-cerebral injection of 6-hydroxydopamine [737]. PD mice were treated with saline, 1 mg/kg etanercept, or 1 mg/kg TfRMAb–TNFR fusion protein IV every other day for 3 weeks. Treatment with the TfRMAb–TNFR fusion protein resulted in an 83% reduction in apomorphine-induced rotation behavior, an 82% increase in vibrissae-elicited forelimb placing, and a 130% increase in striatal tyrosine hydroxylase enzyme activity. In contrast, neither saline nor etanercept treatment had any therapeutic effect in the PD mice [737]. Chronic treatment of mice with the fusion protein induced only a low titer ADA response [737].]. The TfRMAb–TNFR fusion protein was also neuroprotective in experimental stroke, which was induced with a reversible middle cerebral artery occlusion (MCAO) method [738]. The mice were treated with delayed IV administration of either 1 mg/kg etanercept or 1 mg/kg TfRMAb-TNFR fusion protein. Neuroprotection was assessed at both 1 and 7 days after the 60 min MCAO. Treatment with the TfRMAb–TNFR fusion protein caused a 45%, 48%, 42%, and 54% reduction in hemispheric, cortical, and subcortical stroke volume, and neural deficit, respectively. Conversely, treatment with etanercept had no therapeutic effect [738]. The neuroprotective effects in the reversible MCAO model of combined treatment with the TfRMAb-GDNF and TfRMAb-TNFR fusion proteins were additive, illustrating the advantages of combination biologic therapy in a brain disease [732]. Similar to etanercept alone [738], GDNF alone had no neuroprotective effect in experimental stroke [732]. IV etanercept or GDNF are not neuroprotective in experimental stroke because these biologics do not cross the BBB, and because the BBB is intact in the early hours after stroke [758,759], when neuroprotection in stroke is possible [756,757].

#### 8.3.4. Bispecific Antibodies

Biologic drugs, which are mainly monoclonal antibodies, are increasingly receiving FDA approval for non-CNS indications, and in 2019, biologics accounted for 43% of total prescription drug revenues [772]. The development of therapeutic antibodies, particularly for AD, has accounted for significant investment in clinical trials by the pharmaceutical industry. These trials involved the monthly IV infusion of anti-Abeta amyloid antibodies (AAA) on the assumption that a small amount of the antibody in blood would penetrate the BBB to enter brain tissue. It was, and is, commonly assumed that about 0.1–0.2% of the injected antibody reaches the brain [784,785]. This assumption is derived from the observation that the CSF concentration of IgG is about 0.1–0.2% of the plasma concentration [12]. However, as discussed in Section 1.1, it is expected than any antibody in plasma will enter into the CSF compartment owing to the leakiness of the choroid plexus, which forms the blood–CSF barrier, and that antibody penetration into CSF provides no information on BBB transport of the antibody. The important predictor of success in a CNS trial is not whether the biologic enters CSF, but whether the biologic crosses the BBB to enter brain, as demonstrated by in vivo methods reviewed in Section 11. When the brain uptake of a therapeutic antibody is measured in vivo, the brain/plasma ratio of a therapeutic antibody is <0.01%, not 0.1–0.2% [786].

The first AAA to fail in a large phase 3 trial in AD was bapineuzumab [787,788]. Bapineuzumab entered clinical trials even though the preclinical data showed the brain uptake of the antibody in the mouse was no higher than 0.07%ID/g [789], which indicates the antibody is confined to the blood volume of brain [739]. Following the failure of the bapineuzumab trial, another AAA, aducanumab, was developed [790]. Aducanumab was said to cross the BBB because the brain concentration increased as the injection dose (ID) was increased. However, this is expected for an antibody that is confined to the brain blood volume. The measurement of aducanumab in brain was determined after washout of the brain vasculature [790]. However, the brain/plasma ratio of aducanumab was 1 µL/g, which is 5–10% of the brain blood volume, and is indicative of incomplete washout of the brain [786]. Nevertheless, in clinical trials of AD subjects, the entry of aducanumab into the brain of these patients could be inferred, because antibody treatment reduced the amyloid plaque in brain [790]. The mechanism of aducanumab entry into the brain of AD subjects appears to be BBB disruption. A known side effect of AAA therapy in AD is amyloid related imaging abnormalities of edema (ARIA-E) as determined by MRI [790]. ARIA-E is a form of vasogenic edema that follows BBB disruption. In the aducanumab clinical trial, there is a direct relationship between the reduction in amyloid plaque and the ARIA-E, which suggests the aducanumab enters brain through a disrupted BBB [786]. The hypothesis that ARIA-E is required to cause a reduction in amyloid plaque is consistent with the clinical effects of another AAA, crenezumab, which does not cause ARIA-E [791] and does not reduce brain amyloid plaque [792]. Reduction in brain amyloid, as shown by PET, is a surrogate marker. The primary endpoint in the two large aducanumab phase 3 trials was the Clinical Dementia Rating-Sum of Boxes (CDR-SB) [793]. Although neither trial met the endpoint, a post hoc analysis showed a statistically significant improvement in CDR-SB in one of the trials [793]. On the basis of this post hoc analysis, the FDA-approved aducanumab in 2020 for treatment of patients with AD despite the near unanimous rejection of the aducanumab application by the FDA Advisory Committee [793]. Aducanumab was denied approval by the European Medicines Agency (EMA) in late 2021 and in early 2022 the Centers for Medicare and Medicaid Services (CMS) restricted aducanumab reimbursements only for patients in clinical trials. The road to FDA approval of the AAAs for AD proved to be as tortuous as the road to approval of neurotrophins, as reviewed in Section 8.3.2. Both AAAs and neurotrophins, as well as lysosomal enzymes or decoy receptors, need to be re-engineered for BBB transport prior to entry into costly human clinical trials.

When the BBB Trojan horse is a MAb and the neurotherapeutic is a MAb, the re-engineering of the therapeutic antibody requires the production of a bi-specific antibody (BSA). The first BSA engineered for BBB transport was reported in 2007 and involved production of a tetravalent BSA [696]. An AAA was re-engineered as a single chain Fv (ScFv) antibody, and this ScFv was fused to the carboxyl terminus of each heavy chain of the HIRMAb. The HIRMAb-ScFv retained high affinity binding to both the HIR and to soluble Aβ^1–40^ as well as amyloid plaque in brain and amyloid fibrils [696]. The HIRMAb–ScFv fusion protein entered the brain of the Rhesus monkey following IV administration, whereas the AAA alone was confined to the brain blood volume [696]. To enable preclinical studies in AD transgenic mice, the anti-Abeta ScFv was fused to the carboxyl terminus of each heavy chain of the 8D3-derived TfRMAb, and this BSA retained high affinity binding to both the mouse TfR and Aβ^1–40^ [794]. The brain uptake of the TfRMAb-ScFv BSA in the mouse was 3.5 ± 0.7%ID/g following IV administration of [^125^I]-Bolton–Hunter radio-labeled TfRMAb–ScFv fusion protein [794]. Double transgenic APPswe, PSEN1dE9 mice at 12 months of age were treated for 12 weeks with daily SQ injections of saline or 5 mg/kg of the TfRMAb–ScFv BSA [739]. Abeta fibrils in brain were measured by immunohistochemistry with the 6E10 MAb and total amyloid plaque in brain was measured by thioflavin-S fluorescent microscopy. Treatment reduced total plaque in cortex and hippocampus by 49% and 43%, respectively, and reduced Abeta fibrils in cortex and hippocampus by 57% and 61%, respectively [739]. The ARIA-E in AD subjects treated with an AAA [790] is equivalent to the cerebral microhemorrhage in mice treated with an AAA [795]. Mice treated chronically with the TfRMAb-ScFv BSA did not develop cerebral microhemorrhage based on Prussian blue staining of brain, and developed only a low titer ADA response [739].

Subsequent to the description of the BBB-penetrating BSA derived from either with the HIRMAb [696] or the TfRMAb [794], a variety of BBB-penetrating BSAs were engineered that used a multitude of formats for BSA design. A BSA was engineered that targeted both the TfR and BACE1 as a treatment for AD [695,742]. This BSA was engineered with the knob-in-hole technology which placed both the TfRMAb transporting antibody and the BACE1 therapeutic antibody each in a monovalent format. A BSA that targeted both the TfR and the Abeta amyloid peptide was engineered with knob-in-hole technology by fusion of a single chain Fab form of the TfRMAb to the carboxyl terminus of one heavy chain (HC) of a hetero-tetrameric antibody against Abeta [697]. This design placed the TfRMAb in a monovalent form and the AAA in a bivalent format. In a modified tetravalent BSA format, the 8D3 TfRMAb was engineered as a ScFv, which was then fused to the carboxyl terminus of each light chain (LC) of the AAA, mAb158 [796], or an α-synuclein MAb [744]. The mAb158 is the murine precursor to the BAN2401 AAA for AD [797]. Several therapeutic antibodies were re-engineered as a TfRMAb-based BSA using a dual variable domain format where the VH and VL for each antibody was placed in tandem at the amino terminus of each HC and LC [798]. Owing to steric hindrance by the outer domain antibody, the affinity of the inner domain antibody was reduced [798]. A similar tetravalent tandem BSA was engineered with a TfRMAb and an AAA that targeted the protofibrillar form of the Abeta peptide [740]. In another monovalent TfRMAb format, a BSA was engineered that targeted the TfR as a monovalent antibody and BACE1 as a bivalent antibody using knob-in-hole technology [743]. The TfR binding site was created in the CH3 region of one heavy chain by mutagenesis of multiple amino acids [743]. In yet another format for BSA engineering, a single domain shark variable domain of new antigen receptor antibody with high affinity binding to the TfR was fused to the amino terminus of the heavy chain of the bapineuzumab antibody [741].

In summary, since the initial report of the engineering of a tetravalent BSA that targets either the insulin receptor [696] or transferrin receptor [794], at least eight different formats have been used for engineering a BBB-penetrating BSA [695,697,740,741,742,743,744,796,798]. The antibodies range from monovalent for both arms of the BSA, to bivalent for both arms of the BSA, and to monovalent for one arm and bivalent for the other arm. The affinity of the BBB transporting arm of the BSA ranges from high affinity to moderate affinity to low affinity [559,647]. The final test of these BSAs is whether the BSA goes all the way to FDA approval for treatment of AD or another CNS disease. The one BBB-penetrating BSA that is currently in clinical trials is the Roche BSA [697], designated RO7126209, which is in a phase 2 clinical trial for AD [NCT04639050].

### 8.4. Avidin-Biotin Technology

There are classes of biologics that cannot be delivered across the BBB with fusion protein technology, and these include small peptide drugs and nucleic acid pharmaceuticals. Oligopeptides may no longer bind the cognate receptor after fusion to a BBB Trojan horse even if a long linker is employed. Nucleic acid pharmaceuticals cannot be fused to a polypeptide. It is possible to deliver oligopeptides or antisense agents across the BBB with RMT technology that is combined with a linker system, such as avidin–biotin technology. In this approach, the pharmaceutical is formulated in two vials. The first vial contains the mono-biotinylated peptide or antisense agent. The second vial contains a fusion protein of avidin and the IgG RMT Trojan horse, which is generated by the genetic engineering of an IgG–avidin fusion protein. The use of RMT Trojan horse–avidin fusion proteins and peptide or antisense radiopharmaceuticals is particularly amenable to the development of neuro-diagnostics using positron emission tomography (PET) or single photon emission computed tomography external brain imaging.

#### 8.4.1. Peptide Radiopharmaceuticals for Brain Imaging

The first peptide radiopharmaceutical used as a diagnostic agent and external scanning was [^111^In]-octreotide, an 8-AA somatostatin (SST) analogue with a MW of 1395 Da, which enabled the external imaging of neuroendocrine tumors [799]. SST receptors (SSTR) are over-expressed in such tumors [800]. The SSTR is expressed in the CNS, as are receptors for >100 other neuropeptides [801]. However, since neuropeptides do not cross the BBB [802], the development of peptide radiopharmaceutical neuro-diagnostic agents is not possible in the absence of a BBB delivery technology. Epidermal growth factor (EGF) was used to develop a prototype of a BBB-penetrating peptide radiopharmaceutical. The EGF receptor (EGFR) is over-expressed in primary brain cancer [803]. The use of a targeted EGF peptide radiopharmaceutical as an imaging agent in experimental brain cancer was evaluated with an experimental intra-cranial U87 human glioma in nude rats [804].

**Figure 14:**
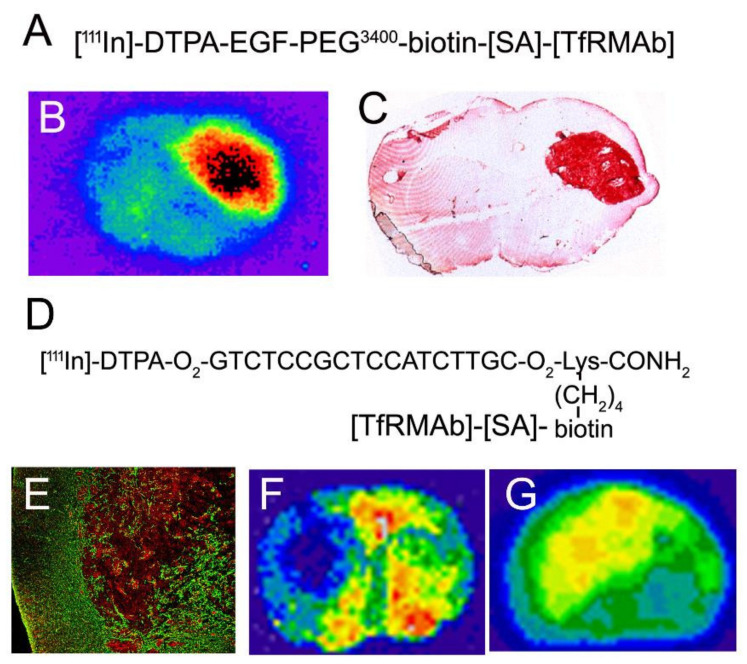
**Imaging brain cancer with peptide or antisense radiopharmaceuticals and BBB drug delivery technology.** (**A**) Structure of [^111^In]-DTPA-EGF-PEG^3400^-biotin bound to the OX26/SA conjugate. (**B**) Film autoradiogram of coronal section of nude rat brain bearing a U87 glioma removed 2 h after IV injection of the BBB-targeted EGF peptide radiopharmaceutical. (**C**) A section parallel to that shown in (**B**) was examined by immunohistochemistry using the 528 MAb against the human EGFR. Panels B and C reproduced with permission [804], Copyright© 1999 American Association Cancer Research. (**D**) Structure of [^111^In]-DTPA-O_2_-18-mer PNA antisense to nucleotides 20–37 of the rat GFAP mRNA. The carboxyl terminus of the PNA incorporates a lysine (Lys) residue and biotin is conjugated to the ε-amino group of the Lys; O = 9-atom linker. The biotinyl PNA is bound by the OX26/SA conjugate. (**E**) Confocal microscopy of an intra-cranial RG-2 tumor in rats that is immune-stained with an antibody to caveolin-1α (red) and an antibody to GFAP (green). (**F**) Film autoradiogram of coronal section of tumor-bearing rat brain removed 6 h after the IV injection of the biotinyl GFAP-PNA-OX26/SA conjugate. (**G**) Film autoradiogram of coronal section of tumor-bearing rat brain removed 6 h after the IV injection of the biotinyl CAV-PNA-OX26/SA conjugate. Panels (**E**–**G**) reproduced with permission [806], Copyright© 2004 SNMMI.

The EGF was re-formulated for BBB delivery in a two-vial approach. Vial A contained EGF that was both radiolabeled and biotinylated. Vial B contained a conjugate of the OX26 TfRMAb specific for the rat TfR and streptavidin (SA). Prior to IV administration in EGFR expressing brain-tumor-bearing rats, the two vials were mixed. Owing to the very high affinity binding of biotin to SA, there was immediate formation of a complex of the EGF peptide radiopharmaceutical and the TfRMAb BBB Trojan horse [804]. The EGF was radiolabeled with 111-indium, which was chelated by a diethylenetriamine pentaacetate (DTPA) group attached to the peptide. In addition, the EGF was conjugated with 3400 Da polyethyleneglycol (PEG^3400^)-biotin. The placement of the PEG^3400^ linker between the EGF and the biotin was necessary to eliminate steric hindrance on EGF binding to the EGFR caused by EGF capture by the OX26/SA conjugate [805]. When a 14-atom linker was placed between the EGF and the biotin, the high affinity binding of the 6 kDa biotinyl-EGF to the EGFR was sterically hindered by the 60 kDa SA [805]. The use of the PEG^3400^ linker created a spacer >200 atoms in length between the EGF and the biotin/SA complex and this extended linker removed the steric hindrance of the SA complex on EGF binding to the EGFR on human glial tumor cells [805]. The [^111^In-DTPA, PEG^3400^-biotinyl]-EGF/SA-OX26 was injected intravenously in nude rats bearing an intracranial human U87 glioma, which over-expresses the EGFR [804]. As a control, the tumor-bearing rats were also injected with the [^111^In-DTPA, PEG^3400^-biotinyl]-EGF without attachment to the TfRMAb Trojan horse. The design of the BBB-penetrating EGF radiopharmaceutical is shown in Figure 14A.

The BBB-targeted EGF peptide radiopharmaceutical shown in Figure 14A enabled brain imaging of an intra-cranial human glial tumor in the nude rats, as shown in Figure 14B. The over-expression of the tumor EGFR was confirmed by immunohistochemistry of the tumor at post-mortem (Figure 14C). No imaging of the tumor was possible following the IV injection of the [^111^In-DTPA, PEG^3400^-biotinyl]-EGF not bound to the OX26 TfRMAb [804]. Recent reviews have discussed the use of peptide radiopharmaceuticals for either therapeutic [807] or diagnostic [808] agents for brain disease. Peptides have the potential for many medical applications in the CNS as there are >100 peptide systems in the brain [801,809]. Such therapeutic and diagnostic applications of peptide radiopharmaceuticals for the brain will require the re-engineering of the peptides with BBB peptide delivery technology.

#### 8.4.2. Antisense Radiopharmaceuticals for Brain Imaging

Over 20 years ago, there was promise for imaging CNS gene expression with sequence specific antisense radiopharmaceuticals [810,811]. Antisense agents are either phosphodiester antisense oligodeoxynucleotides (ASO), phosphorothioate ASOs, or peptide nucleic acids (PNA). Phosphodiester ASOs are not suitable agents for in vivo use, owing to the rapid degradation of phosphodiester ASOs by endo- and exo-nucleases in vivo. Phosphorothioate ASOs are more resistant to nucleases, but are not suitable imaging agents because binding of a phosphorothioate ASO to a target mRNA forms a DNA:RNA heteroduplex, which triggers mRNA degradation by RNase H [812]. PNAs are the preferred antisense imaging agents, as the PNA polypeptide backbone is not degraded by nucleases, and PNAs do not activate Rnase H [813]. However, PNAs are highly polar molecules that do not cross the BBB [814]. PNAs can be re-formulated to cross the BBB and retain affinity for the target mRNA sequence using BBB RMT and avidin–biotin technology. An 18-mer PNA was synthesized with a nucleobase sequence that was antisense to either nucleotides (nt) 10–27 of the rat caveolin (CAV)-1α mRNA (AF439778) or to nt 20–37 of the rat GFAP mRNA (NM_017009) [806]. The sequence of the GFAP PNA is shown in Figure 14D. The amino terminus of the PNA was conjugated with DTPA, which chelates the 111-indium radiotracer. A double 9-atom linker (O) was placed at both the amino terminus between the DTPA and the PNA, and at the carboxyl terminus between the PNA and the terminal lysine (Lys) as shown in Figure 14D. The ε-amino group of the Lys amino acid was biotinylated, which allowed binding of the biotinyl PNA to a conjugate of SA and the OX26 rat TfRMAb (Figure 14D). Northern blotting with synthetic GFAP or LAT1 mRNA, produced by in vitro transcription, showed the GFAP PNA bound to the GFAP mRNA, but not the LAT1 mRNA, despite being bound by the OX26/SA conjugate [806]. Fischer CD344 rats bearing an intra-cranial RG-2 tumor were studied for brain imaging with either the CAV PNA or the GFAP PNA. Confocal microscopy showed the experimental brain tumor over-expressed the CAV protein and under-expressed the GFAP protein (Figure 14E). Loss of GFAP expression is typical in high grade glial tumors [815]. GFAP mRNA in the RG-2 glioma cells in culture was not detectable by Northern blotting even after over-exposure of the film [806]. In vivo brain imaging of the RG-2 intra-cranial tumors with the CAV PNA or the GFAP PNA antisense radiopharmaceutical demonstrated the under-expression of the GFAP mRNA (Figure 14F) and the over-expression of CAV mRNA (Figure 14G) in the intra-cranial brain tumor, providing the PNA was conjugated to the TfRMAb Trojan horse [806]. Only blank brain scans were produced when the GFAP PNA alone or the CAV PNA alone were injected intravenously in the tumor-bearing rats [806]. Antisense agents had been proposed as new approaches to the in vivo imaging of gene expression in the brain [816]. This is still possible, providing the antisense agents are re-formulated with a BBB Trojan horse brain delivery technology [806].

#### 8.4.3. IgG–Avidin Fusion Proteins

TfRMAb–avidin fusion proteins were generated from the OX26 rat TfRMAb as either a ScFv-SA fusion protein produced in E. coli [692], or as a bivalent TfRMAb–avidin fusion protein produced in myeloma cells [817]. However, the problem with production of IgG–avidin fusion proteins in mammalian expression systems is the high concentration of biotin in tissue culture medium [818], coupled with the very slow dissociation of biotin from avidin. Biotin binding to avidin is characterized by a KD of 10^−15^ M, and a dissociation T_1/2_ of 3 months [819]. The IgG–avidin fusion protein produced with standard expression media is fully loaded with biotin [702], and only harsh denaturing conditions can separate the avidin and biotin [819]. An IgG–avidin fusion protein that is saturated with biotin has little utility for brain drug delivery of biotinylated agents. Therefore, it was necessary to develop culture conditions that produce a gradual depletion of medium biotin. In standard culture medium, the biotin concentration is 800 nM, which greatly exceeds the concentration of the avidin fusion protein. The CHO cells stably transfected with the HIRMAb–avidin fusion protein were re-suspended in custom biotin-free medium supplemented with 20 nM biotin. The medium biotin concentration was reduced to 10 nM, 3 nM, 1 nM, and 0.3 nM, at days 8, 14, 18, and 21 of growth in serum free medium [702]. The HIRMAb–AV fusion protein produced under these conditions retained 1 unoccupied biotin binding site per hetero-tetrameric IgG–avidin fusion proteins, which is composed of 2 avidin monomeric chains fused to the carboxyl terminus of each HIRMAb heavy chain [702].

An 8D3-derived TfRMAb–avidin fusion protein was produced in stably transfected CHO cells under conditions of biotin depletion, and the fusion protein retained high affinity binding for the mouse TfR and 1 biotin binding site per tetramer [820]. The biologic activity of the fusion protein was tested in vivo in mice with respect to brain delivery of [^125^I, biotinyl]-Aβ^1–40^, a potential peptide radiopharmaceutical for imaging the brain amyloid burden of AD [686,687]. Currently, the brain amyloid burden in AD is imaged with small molecules such as florbetapir [790], which has a brain uptake of 2–3%ID/g at 60 min after IV administration in the mouse [821]. However, lipid-soluble small molecules such as florbetapir are subject to rapid efflux from brain to blood [822], and a significant amount of florbetapir can efflux from brain during a 2 h scanning period. A peptide radiopharmaceutical, such as Aβ^1–40^, may be a preferred imaging agent owing to a longer brain residence time [688], should the peptide be made transportable through the BBB. Aβ^1–40^ alone does not cross the BBB [687,820]. The delivery of N-biotinyl Aβ^1–40^ to mouse brain was assessed following radio-iodination of the peptide with the [^125^I]-Bolton–Hunter reagent, and conjugation to the 8D3-derived TfRMAb–avidin fusion protein [820]. The brain uptake of [^125^I, biotinyl]-Aβ^1–40^ conjugated to the TfRMAb–avidin fusion protein is 2.1 ± 0.2 %ID/g in the mouse following IV injection [820]. Therefore, the use of BBB RMT delivery technology increases the brain uptake of a large molecule peptide radiopharmaceutical to the same level of brain uptake observed with a lipid-soluble small molecule such as florbetapir [820].

## 9. Nanoparticles

### 9.1. Nanoparticle Formulations

There are three broad classes of nanoparticles (NP) [823]:

- **Polymer-based nanoparticles**, which include polymeric NPs (PNP), dendrimers, micelles, and protein nanoparticles, such as albumin nanoparticles;
- **Lipid-based nanoparticles**, which include liposomes, which have an aqueous interior, and solid lipid nanoparticles (SLN), which lack an aqueous interior; exosomes, which are reviewed in Section 5.2, can be considered natural liposomes;
- **Non-polymeric nanoparticles**, which include carbon nanotubes (CNT), graphene oxide (GO) fullerenes or quantum dots, and metallic nanoparticles produced from metals such as iron, gold, silver, or silica. Iron nanoparticles are magnetic.

Nanoparticles may be functionalized by conjugation of ligands on the surface of the nanoparticle, where such ligands are intended to mediate endocytosis of the nanoparticle following binding to either a carrier-mediated transporter (CMT) or a receptor-mediated transporter (RMT). Nanotechnology, which includes nanoparticles, liposomes, dendrimers, and exosomes, constitute 32% of all brain drug delivery research (Table 1). Despite the development of nanoparticles over the last 30 years, there have been few nanoparticle formulations to enter into CNS clinical drugs, and there have been no FDA approvals of nanoparticles for brain disease [824], as reviewed below. Nanoparticles are complex structures that can exert toxic effects in brain with neuropathologic changes, as discussed below. However, owing to the very few Investigational New Drug (IND) applications to the FDA for brain-targeted nanoparticles, these agents have not been subjected to the rigorous preclinical GLP safety pharmacology and toxicology studies in two species required for an IND. An IND application also requires a proven and scalable plan for manufacturing of the drug product under Good Manufacturing Practice (GMP). A PubMed search for ‘nanoparticle GMP manufacturing’ lists no entries (January 2022).

### 9.2. Polymer-Based Nanoparticles

#### 9.2.1. Polymeric Nanoparticles

The first use of polymeric nanoparticles (PNP) to deliver drug across the BBB was reported in 1995 [64]. The opioid hexapeptide, dalargin, was adsorbed to the surface of PNPs prepared from poly(butyl cyanoacrylate) or PBCA. The dalargin was added to the PBCA and the suspension was sonicated and either 0 or 1% polysorbate-80 (PS80, Tween-80), a non-ionic detergent, was added, and the suspension immediately injected intravenously in mice. Analgesia was measured with the tail-flick method [64]. Analgesia was not induced by the peptide alone, the peptide adsorbed to the PNPs, or the peptide plus the PS80, but was induced by the combination of the peptide, the PNPs, and PS80 [64]. Subsequently, it was shown that plasma proteins are adsorbed to the surface of the PS80-coated PNPs, including apolipoprotein B (apoB) and apolipoprotein E (apoE), and it was hypothesized that apoB or apoE on the NP surface acted as ligands and attached the NP to the LDLR on the BBB to trigger transport of the PNP into brain [825]. However, as discussed in Section 8.1.6, the LDLR is not localized to the BBB by immunohistochemistry. The absence of the LDLR on the BBB is consistent with the lack of transport across the BBB of LDL cholesterol [611,612,613]. The model of BBB transport of the PNP was revised following the demonstration of binding of apoA-I to the surface of the nanoparticle, and it was then hypothesized that the apoA-I triggered transport not via the LDLR, but via the scavenger receptor (SR)-B [826], which is also known as CD36. CD36 is localized to the microvasculature in brain by immunohistochemistry [827]. The SR-B is a ligand for oxidized LDL, such as acetylated LDL [828]. Acetylated LDL is endocytosed, but not transcytosed, across the BBB in vivo as demonstrated by internal carotid artery infusion with capillary depletion [506]. Therefore, the BBB SR-B is only an endocytosis system, and does not mediate transcytosis through the BBB [506]. The microvascular SR-B/CD36 is believed to participate in phagocytosis at the neurovascular unit [829]. The presence of PS80, or other surfactants, in the PNP formulation is essential for BBB transport [830]. Given that the SR-B is only an endocytosis system at the BBB, the mechanism is unclear by which PS80 enables PBCA PNPs to cross the BBB in vivo. Toxic effects of the PS80-coated PBCA PNP at the BBB are discussed below in Section 9.7 on nanoparticle toxicity.

PNPs are also stabilized by the addition of a corona of polyethyleneglycol (PEG) on the surface of the nanoparticle [831]. PNPs were prepared from 45 kDa poly(lactic acid) (PLA) and PEG conjugated poly(lactic coglycolic acid) (PLGA). The size of the PEG varied from 2 to 20 kDa [831]. Pegylation of PNPs minimizes adsorption of plasma proteins to the surface of the PNP. In the absence of the PEG surface coating, this serum protein adsorption triggers rapid uptake by the reticulo-endothelial system in vivo and accounts for very rapid plasma clearance of the non-pegylated PNP [832].

#### 9.2.2. Dendrimers

Dendrimers are tree-like branching structures that can vary in MW from ~1 kDa to ~1000 kDa, and can have a net neutral or cationic charge. A poly(amidoamine) (PAMAM) dendrimer was tritiated and injected intravenously in mice [65]. The cationic dendrimer had a higher organ uptake than the neutral dendrimer. The organ with the highest uptake was the lung and the organ with the lowest uptake was the brain. Dendrimers alone do not cross the intact BBB [833]. Therefore, dendrimers need to be targeted to brain. A PAMAM-PEG-Tf or PAMAM-PEG-Lf conjugate was prepared and injected intravenously in mice [834]. The organs with the highest uptake were liver, lung, spleen, and kidney and uptake by heart and brain was low. Exogenous Tf is not expected to act as a TfR-directed Trojan horse at the BBB in vivo, because the concentration of endogenous Tf fully saturates the BBB TfR [647], as discussed in Section 8.2.1. Lf, a ligand for LRP1, is not expected to act as a BBB Trojan horse, since the LRP1 is not expressed on the endothelium, as discussed in Section 8.2.1. The ultimate utility of amine-terminated dendrimers may be limited by the cytotoxicity of these agents and the aggregation of the cationic dendrimers when mixed with serum [834]. The serum aggregation of cationic dendrimers is similar to the saline-induced aggregation of cationic liposome/DNA complexes [835], which is discussed in Section 10.2.

#### 9.2.3. Micelles

Amphiphilic sodium alginate cholesterol derivatives were synthesized and self-assembled into 200 nm micelles, which were loaded with a polar neuroprotective oxysteroid [836]. The micelles alone did not cross the BBB, so the micelles were targeted with lactoferrin (Lf). However, Lf is not a useful ligand for brain targeting. Although Lf is a ligand for the LRP1, this receptor is not localized to the brain endothelium as discussed in Section 8.1.5. Additionally, Lf is not a ligand for the TfR [837]. The brain uptake of the Lf-targeted micelles was very low, 0.05%ID/g [836], which indicates the micelles are confined to the blood volume of brain. In another application, micelles were formed with distearoylphosphatidylethanolamine (DSPE)-PEG^2000^-CREKA, where CREKA is a pentapeptide that binds fibrin deposits at the tumor vasculature [838]. The micelles were formed at 80 °C, cooled and injected intravenously in mice. The micelles were primarily cleared by liver and kidney and demonstrated minimal, if any, transport across the BBB [838]. Micelles were formed by 24 h incubation in water of GM1 monosialogangliosides, which formed micelles with a mean diameter of 226 nm [839]. It was hypothesized that the GM1 gangliosides form a complex with serum albumin, which then mediates RMT across the BBB via the gp60 albumin receptor expressed in cultured endothelium [840]. However, the albumin receptor is not expressed on brain microvessels [841].

#### 9.2.4. Albumin Nanoparticles

Human serum albumin (HSA) is converted into nanoparticles by an ethanol desolvation/glutaraldehyde cross-linking method [842]. In an effort to deliver the HSA NPs across the BBB, apoE3 was chemically cross-linked to the HSA NPs [843]. As discussed in Section 9.2.1, PBCA NPs coated with PS80 were said to bind apoE to trigger RMT across the LDLR on the BBB [825]. However, the BBB transport of the apoE3-HSA NPs was only evaluated by electron microscopic identification of 200–250 nm electron dense particles in selected fields of mouse brain, and it is difficult to interpret this small sampling. Endocytosis was demonstrated by electron microscopy of bEND.3 brain endothelium in cell culture [843]. There is a rationale for endocytosis in the cultured endothelium, because cultured cells express the LDLR [610]. However, the LDLR is not expressed in brain in vivo at the brain microvasculature [562]. Lipid-free ApoE does bind the SR-B scavenger receptor [844], but this receptor at the BBB only mediates endocytosis, not transcytosis, across the BBB [506]. HSA NPs were produced by the ethanol desolvation/glutaraldehye method as well as by an ethanol desolvation/thermal (90 °C) denaturation method. No coupling of lipoprotein or use of PS80 was used in this investigation [845]. BBB transport was estimated in rats by fluorescent microscopy following the IV injection of a large dose, 50 mg/kg, of the HSA NPs. On the basis of this qualitative microscopy method, the HSA NP was said to cross the BBB by a proposed mechanism of absorptive-mediated transport [845]. However, these HSA NPs were neither cationic or conjugated with lectins, which are the primary mechanisms of BBB absorptive-mediated transport (Section 7).

### 9.3. Lipid-Based Nanoparticles

#### 9.3.1. Liposomes

Liposomes are nanoparticles formed from lipids with an aqueous interior. In contrast, solid lipid nanoparticles (SLN) have a solid lipid interior. The first investigation of drug delivery to brain with liposomes was reported in 1990 [63]. Phosphatidylcholine/cholesterol liposomes were prepared and injected in the carotid artery of Fisher 344 rats with an intra-cranial 9 L glioma. The liposomes did not enter the hemisphere of brain contralateral to the tumor, which indicated liposomes do not cross the BBB in normal brain. There was uptake of the liposomes by the tumor and the brain adjacent tumor [63], because the blood–tumor barrier is leaky in the 9 L glioma model [846]. In this early study, the liposomes were infused into the carotid artery. It was not possible to use the IV route of administration, because liposomes are rapidly cleared from the blood similar to PNPs in the absence of a PEG corona [847]. Stealth liposomes have a PEG corona which produces a longer blood residence time, and Doxil^®®^ stealth liposomes were evaluated for brain uptake in 1995 [847]. Doxil is doxorubicin encapsulated in liposomes prepared from phosphatidylcholine/cholesterol/PEG^1900^, and was injected intravenously in rats with an intra-cranial glioma [847]. The Doxil liposomes were delivered to the experimental glioma, as this tumor was shown to have a leaky BBB, but the Doxil liposomes were not taken up by the contralateral brain [847]. This study shows that pegylated liposomes do not cross the intact BBB, similar to the lack of BBB transport of pegylated HSA NPs [843].

#### 9.3.2. Solid Lipid Nanoparticles

Solid lipid nanoparticles (SLN) have a solid lipid interior, as opposed to liposomes, which have an aqueous interior. The drug loading capacity of SLNs is not high, and the loading capacity is higher for nano-structured lipid carriers (NLC) [848]. Lipid nanoparticles (LNP) is a generic term that includes liposomes, SLNs, NLCs, and cationic lipoplexes [849]. The early work on SLNs for brain tested BBB transport only in cell culture models [848]. SLNs are particularly suited for drugs with low aqueous solubility. In one SLN application, a drug that is insoluble in water, camptothecin, was incorporated in cetyl palmitate SLNs with and without stabilization by PS80 [850]. The SLNs were formed by heating at 60 °C followed by homogenization and sonication. Brain uptake of the SLN/camptothecin was low unless the PS80 was added to the formulation. The explanation for the PS80 effect was taken from prior work with PBCA NPs [825,826], and it was assumed lipoproteins are bound to the PS80, which triggers uptake via the presumptive LDLR on the BBB. However, in this SLN study [851], as in the PBCA NP work [825,826], no evidence was provided that the LDLR is expressed at the BBB. SLNs require targeting agents to mediate delivery of the particles across the BBB [850], as reviewed below.

### 9.4. Non-Polymeric Nanoparticles

#### 9.4.1. Carbon Nanotubes

Carbon nanotubes (CNT) are needle-like structures and may be single walled nanotubes (SWNT) or multi-walled nanotubes (MWNT), which have diameters of 0.4–2 nm and 10–100 nm, respectively, and can be 50 nm to >1000 nm in length [852]. CNTs are allotropes of carbon; CNTs have a tube structure, fullerenes have a cage structure, and graphene is flat. CNTs are produced by electric arc discharge and laser ablation or by passage of carbon-containing vapors in a furnace with a metal catalyst [852]. CNTs are insoluble in water and have to be functionalized by chemical modifications to the carbon lattice for biomedical applications. The ‘needle’ structure of CNTs is believed to facilitate the piercing of cell membranes so that CNTs may gain access to the intracellular compartment [852]. CNTs are toxic to cells [852,853]. SWNTs were loaded with acetylcholine by adsorption of the drug to the walls of the SWCNT and injected intravenously into mice at a dose of 20–50 mg/kg of SWNT, which corresponds to an acetylcholine dose of 4–10 mg/kg [853]. The SWCNTs were said to cross the BBB based on an improved performance by AD transgenic mice in a shuttle box test [853]. SWCNTs were said to cross the BBB based on experiments performed solely in cell culture [854]. MWCNTs were functionalized with surface cationic, anionic, or non-ionic groups and transport across the monolayer of cultured hCMEC/D3 endothelial cells was determined [855]. Cationic and non-ionic MWCNTs were largely confined to the cell glycocalyx. Anionic MWCNTs had the highest rate of transport in cell culture, and no in vivo studies of BBB transport of CNTs were performed [855]. Similar to PNPs and SLNs, CNTs must incorporate surface ligands to stimulate endocytosis without cell damage [856].

#### 9.4.2. Graphene Oxide, Fullerenes, and Quantum Dots

Graphene is a two-dimensional carbon nanosheet, which is oxidized to form graphene oxide (GO), where the graphene surface is decorated with carboxyl or hydroxyl groups. Certain drugs, such as doxorubicin (Dox), were bonded non-covalently to the to GO sheet by π-π stacking [857]. The GO surface was also conjugated with PEG, and the Dox-GO-PEG, as well as free Dox, were injected intravenously in rats. Binding of the Dox to the GO-PEG had no effect on the brain uptake of the Dox [857]. The authors concluded that GO nanosheets need to be modified with receptor ligands to facilitate BBB transport, as discussed below.

Fullerenes are 60-carbon caged carbon structures, which are not water soluble. Chemical linking of water soluble groups such a tris-malonic acid produces a water soluble fullerene designated *C3*, which is a superoxide dismutase (SOD)-mimetic [858]. The C3 fullerene was injected intravenously in mice, and the brain uptake of the fullerene appeared confined to the blood volume of the mouse, although no corrections for blood volume were performed [858].

Graphene oxide quantum dots are spherical structures with a diameter of about 10 nm [859]. GO carbon dots were conjugated to glucose to facilitate CMT across the BBB on the GLUT1 glucose carrier [860]. The carbon dot was also conjugated with fluorescein, and the glucose/GQD/fluorescein structure was designated GluCD-F. The complex was injected intravenously and BBB transport assessed qualitatively by fluorescent microscopy. It is difficult to confirm BBB transport of the GlutCD-F in this small field sample. The GluCD-F dots had a mean diameter of 3.8 nm [860]. However, as shown in Figure 9A, the glucose cavity in the GLUT1 transporter is a highly confined space that has a diameter of only 1.2 nm [347]. Therefore, the GlutCD-F structure has a diameter >3-fold greater than the diameter of the GLUT1 cavity, so it is difficult to see how this complex can traverse the BBB via GLUT1. The GLUT1 carrier can be expressed in transfected cells or frog oocytes for direct examination of whether the GlutCD-F is transported via GLUT1, but this has not been performed.

#### 9.4.3. Metallic Nanoparticles

Nanoparticles have been produced from several metals including gold (Au), iron oxide (FeO), silver (Ag), and silica.

**Gold nanoparticles (AuNP).** AuNPs of 15 nm diameter were coated with albumin and poly(allylamine), a highly cationic polymer, and injected intravenously in mice [861]. The AuNPs were said to cross the BBB based on fluorescent microscopy, but the fluorescent signal may have been due to the aggregation of the AuNPs at the microvascular surface. In another study, AuNPs did not cross the BBB in the absence of BBB disruption caused by external laser irradiation of the brain [862]. Severe combined immune-deficient (Scid) mice with intra-cranial U87 human gliomas were treated with 13 nm AuNps conjugated with siRNA, and the AuNPs were observed to cross the leaky blood–tumor barrier but not the BBB in normal brain [863]. A recent review of nearly 40 studies on the use of AuNPs for brain delivery showed an average brain uptake of 0.06%ID/g [864], which is very low and could be explained on the basis of AuNPs residing in the brain blood volume. AuNPs were hypothesized to cross the BBB via calcium or potassium channels [865]. Even very small AuNPs with a diameter of 2.5 nm are large compared to the diameter of the pore size of calcium or potassium channels, which is 0.9–1.5 nm [866,867,868]. Moreover, AuNPs conjugated with siRNA have a diameter of 19–34 nm [863].

**Silver nanoparticles (AgNP).** AgNPs were combined with PLGA polymeric nanoparticles (PNP), which produced AgPNPs of 191 nm in diameter [869]. The AgPNPs were conjugated with chlorotoxin, a 36-AA scorpion toxin that binds the matrix metalloproteinase 2 (MMP2) of glioma cells, and was sequestered in a flank glioma in mice after IV administration [869]. No studies of AgPNP transport across the intact BBB in brain were performed.

**Silica nanoparticles (SiNP).** SiNPs were prepared from tetraethylorthosilicate [870], which is (CH_3_CH_2_O)_4_-Si. The SiNPs were infused in the carotid artery of rats, and the brain was stained with a silica selective fluorescent compound used to detect silica in soils. Based on fluorescent microscopy [870], the SiNPs were said to cross the BBB, although the micrographs suggest the SiNPs are largely sequestered within the vasculature. In another study [871], SiNPs were produced from tetraethylorthosilicate and the surface was coated with PEG-poly(ethyleneimine), or PEI, which is a cationic polymer. The functionalized SiNPs were injected in the mouse and brain uptake visualized by two-photon microscopy through a cranial window. The SiNPs were said to cross the BBB [871], although inspection of the micrographs suggest the SiNPs are largely sequestered within the vasculature.

**Magnetic iron nanoparticles.** Iron NPs (FeNP) are magnetic and designated superparamagnetic iron oxide nanoparticles or SPION [872]. SPIONs were functionalized by adsorption of PEG and PEI to the surface and stabilized by the addition of PS80. The SPIONs were injected intravenously in the rat, which was subjected to an external magnetic field (EMF) by fixation of a magnet over the skull. In the absence of either the EMF or the PS80, there was no brain uptake of the SPIONs; however, brain uptake of the PS80-stabilized SPIONs was observed in the rats subjected to an EMF [872]. This work was said to provide the basis for therapeutic applications of Tween-SPIONs under EMF [872]. Iron oxide NPs were mixed with a complex of PEI and DNA to form FeNPs. These were added to cultured cells for the assessment of gene expression, a process named magnetofection [873]. However, exposure of cultured cells to a magnet did not enhance gene expression with the FeNPs. The diameter of the FeNPs in water was ~150 nm. However, when the FeNPs were added to tissue culture medium, the FeNPs aggregated to a diameter >1 micron [873]. Such aggregation stimulates phagocytosis [874], which appears to be the principal mechanism for cell entry of the FeNPs coated with PEI/DNA.

In summary, despite the diversity of nanoparticle formulations that have evolved over the last 25 years, the data reviewed above show that nanoparticles do not cross an intact BBB. The one exception to this rule may be PBCA polymeric nanoparticles that are coated with the surfactant, PS80 [825,833]. An early study of PS80-coated PBCA nanoparticles and brain delivery showed pharmacologic effects could be attributed to the toxic effects of the PBCA polymer, which were augmented by the PS80 detergent [875], and the toxicity of PS80/PBCA PNPs is discussed further in Section 9.7. Given the lack of nanoparticle transport across the BBB, nanoparticles need to be re-formulated to access endogenous transport systems within the BBB, similar to classic small or large molecules discussed in Section 6, Section 7 and Section 8. As reviewed in Section 9.5, nanoparticles have been functionalized in a variety of ways so as to undergo transport through the BBB via CMT, AMT, or RMT mechanisms. In addition, nanoparticles have been delivered to brain with BBB avoidance strategies, such as BBBD with focused ultrasound, intra-cerebral delivery with convection-enhanced diffusion, or trans-nasal delivery.

### 9.5. Mediated Blood–Brain Barrier Delivery of Functionalized Nanoparticles

#### 9.5.1. Carrier-Mediated Transport of Nanoparticles

To facilitate nanoparticle transport across the BBB, micelles were produced with a PEG^2000^-poly(α,β-polyaspartic acid) co-block polymer, which included a terminal D-glucose moiety, so as to enable BBB passage via the GLUT1 glucose transporter [876]. Fluorescent microscopy showed the micelles were largely trapped in the intra-vascular compartment of brain and no quantitative measurement of brain uptake was reported. Pegylated PLGA nanoparticles were conjugated with ascorbic acid to facilitate transport of the PNPs across the BBB via the sodium dependent vitamin C (SVCT) carrier [877]. However, ascorbic acid does not cross the BBB; instead dehydroascorbate, the oxidized form of vitamin C, crosses the BBB via the GLUT1 transporter [878]. Similarly, dehydroascorbate, rather than ascorbate, is the form of vitamin C that crosses the blood–retinal barrier (BRB) on the GLUT1 carrier [879]. Transport of the ascorbate-targeted PEG-PLGA nanoparticles was demonstrated only in a cell culture model or with neurobehavior tests, without in vivo measurements of BBB transport [877]. AuNPs were targeted with L-DOPA to enable transport via the LAT1 large neutral amino acid carrier at the BBB, and BBB transport was only assessed with cell culture models [880]. Dendrimer-based micelles encapsulating doxorubicin (DOX) were conjugated with choline to enable transport on the BBB choline carrier, which is presumed to be CTL1, as discussed in Section 6.2.6. Whereas DOX uptake in a leaky experimental brain tumor was increased, the uptake of DOX across the intact BBB was negligible [881]. Pegylated liposomes were conjugated with the tripeptide, glutathione (GSH), on the assumption that a GSH transporter is expressed at the BBB [882]. However, as discussed in Section 8.2.1, GSH does not cross the BBB [677], and a GSH transporter at the BBB has not been identified [678].

The available data show that targeting CMT systems at the BBB for nanoparticle delivery is not advisable. Nutrients traverse the SLC CMT systems via narrow, stereospecific cavities, as illustrated for the GLUT1 or LAT1 carriers in Figure 9. The width of these cavities is about 1 nm, as discussed in Section 6.2.1. There is no direct evidence that even small nanoparticles can fit through these CMT cavities. The SLC carriers do not mediate endocytosis, as is the case for the RMT systems, which means there is no plausible mechanism by which nanoparticles can be transported by the CMT systems. The counter argument is that certain viruses, which have a size comparable to a nanoparticle, enter cells by first binding a CMT system. As discussed in Section 6.2.3, the murine ecotropic retrovirus binds CAT1 [379], as does the bovine leukemia virus (BLV) [883]. The human T cell leukemia virus binds GLUT1 [884]. However, virus endocytosis into cells is a two-step process of binding to a cell membrane receptor, e.g., a CMT system, followed by membrane fusion, which then triggers endocytosis. This two-step process is illustrated with the Severe Acute Respiratory Syndrome Coronavirus 2 [885]. The S1 domain of the virus spike protein binds the cell membrane receptor, angiotensin converting enzyme 2 (ACE2). Binding to ACE2 alone does not trigger endocytosis of the virus. After ACE2 binding, the spike protein is cleaved by furin into the separate S1 and S2 subunits, and the cleaved S2 subunit fuses with the cell membrane to enable virus endocytosis [885]. Nanoparticles targeting a CMT system would need to be further functionalized with a membrane fusion domain.

#### 9.5.2. Absorptive-Mediated Transport of Nanoparticles

Nanoparticles have been modified by the addition of cationic agents, such as protamine or cationized albumin to facilitate BBB transfer via the AMT mechanism reviewed in Section 7. Cisplatin loaded PLGA PNPs were cationized by loading protamine to the surface of the nanoparticle [886]. However, BBB transfer was only evaluated in cell culture. Pegylated poly(lactic acid) (PLA) PNPs were prepared and surfaced conjugated with cationized bovine serum albumin (cBSA) with a thio-ether bond [887]. However, BBB transport was only assessed with an in vitro model, where transport of the cBSA-PNPs moved across the endothelial monolayer faster than PNPs conjugated with native bovine serum albumin [887]. Pegylated liposomes were covalently conjugated with cBSA, and brain uptake was assessed in vivo [888]. However, after IV injection in rats, the cBSA-liposomes were confined to the vascular wall without significant transport into brain parenchyma [888]. Cationized bovine serum albumin was conjugated to a PAMAM dendrimer, which was mixed with a DOX loaded PLGA PNP so as to enhance BBB transport of the DOX chemotherapeutic agent. However, BBB transport was only assessed in vitro [889]. As discussed in Section 7, AMT at the BBB can also be mediated by lectins such as WGA. This lectin was conjugated to PEG-PLA PNPs and cell uptake and toxicity was assessed in cell culture. The higher the WGA content of the nanoparticle, the higher the cell uptake, but the greater the cell toxicity [890]. The toxicity of the WGA nanoparticle is not unexpected given the known toxicity of this lectin as discussed in Section 7.3.2. In summary, the delivery of nanoparticles across the BBB via AMT pathways used by cationic proteins or lectins is not promising, owing to the sequestration of the complex in the endothelial compartment and to the toxicity of cationic proteins or lectins (Section 7.3).

#### 9.5.3. Receptor-Mediated Transport of Nanoparticles

The RMT delivery of nanoparticles across the BBB is similar to the delivery of large molecule biologics. Nanoparticles alone do not cross the non-disrupted BBB, as reviewed in Section 9.2, Section 9.3 and Section 9.4. Similar to biologics, the BBB transport of nanoparticles is not possible using CMT systems, as these transporters generally do not undergo endocytosis. Additionally, similar to biologics, the re-formulation of nanoparticles to access RMT systems within the BBB provides greater brain uptake as compared to nanoparticles that access AMT systems. As reviewed below, both peptides and receptor-specific MAbs have been used as Trojan horses to enable RMT of nanoparticles across the BBB.

**Figure 15:**
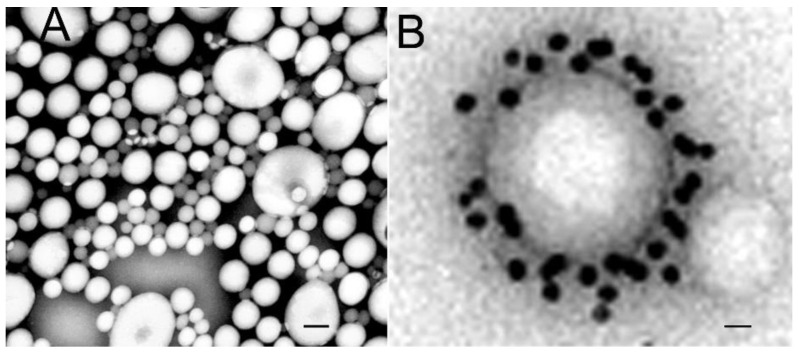
**Pegylated immuno-nanoparticles.** (**A**) Transmission EM of pegylated PLA nanoparticles counter-stained with phosphotungstic acid. Magnification bar = 100 nm. (**B**) Transmission EM of the complex of the OX26 antibody-pegylated immunonanoparticles bound by a 10 nm gold conjugated secondary antibody. Magnification bar = 15 nm. Reproduced with permission [892], Copyright© 2002 Springer-Nature.

**Transferrin receptor antibody-targeted nanoparticles.** The first nanoparticle formulated for RMT across the BBB was reported in 1996 in the form of pegylated immunoliposomes [891], also called Trojan horse liposomes (THL). Pegylated liposomes were surface conjugated with the mouse OX26 MAb against the rat TfR, and BBB transport was demonstrated in vivo in the rat [891]. The optimal use of THLs is the brain delivery of plasmid DNA for non-viral brain gene therapy, as discussed in Section 10.2. Subsequent to the description of BBB-penetrating pegylated immunoliposomes, pegylated PLA nanoparticles conjugated with the OX26 TfRMAb for brain delivery was described in 2002 [892]. The PNPs were produced from methoxy-PEG^2600^-PLA^40000^ and maleimide-PEG^3500^-PLA^40000^ with an emulsion/solvent evaporation method with 1% sodium cholate as a surfactant. The diameter of the pegylated PLA nanoparticles was 121 ± 5 nm, based on dynamic light scattering. Transmission EM showed most of the PNPs had a diameter of ~100 nm, although a few were 200–300 nm in size, as shown in Figure 15A.

The OX26 antibody was thiolated with 2-iminothiolane in parallel with production of the pegylated nanoparticles. The thiolated OX26 antibody was conjugated to the PEG-extended maleimide group on the surface of the nanoparticle to form a stable thio-ether linkage. The conjugation of the OX26 antibody to the surface of the pegylated nanoparticle is shown in Figure 15B. The relationship of the OX26 antibody and the nanoparticle surface was examined by binding a 10 nm gold conjugated secondary antibody to the pegylated immunonanoparticle, followed by washing and electron microscopy (Figure 15B). The number of OX26 antibodies conjugated per pegylated immunonanoparticle was 67 ± 4 [892].

The OX26 TfRMAb is specific only for the rat TfR, and is not active in mice [663]. TfR antibodies that react with the mouse TfR that could be used for BBB delivery in the mouse were described in 2000, as it was shown the rat 8D3 MAb against the mouse TfR, or the rat RI7-217 MAb against the mouse TfR, penetrated the BBB in the mouse [663]. Just as the OX26 antibody is active in the rat, but not the mouse [663], the RI7-217 antibody is active in the mouse, but not in other species [691]. The species specificity of the TfRMAbs has not been considered in several studies of NP targeting to brain with a MAb against the TfR. For example, the BBB transport of OX26-targeted liposomes was evaluated in a human endothelial hCMEC/D3 culture model [893], but the OX26 TfRMAb does not recognize the human TfR [689]. If human cells are used as a model system, then a TfRMAb specific for the human TfR should be used, such as the 128.1 TfRMAb against the human TfR [894]. Pegylated chitosan nanoparticles were conjugated with the OX26 antibody [895]. However, these in vivo transport studies are difficult to interpret, since the studies were conducted in the mouse [895], and the OX26 antibody is not active in the mouse [663]. In another report confounded by species specificity of the TfRMAbs, pegylated PLA PNPs were conjugated with the OX26 antibody, and BBB transport was tested in cell culture with human cells, and in vivo in the mouse [896]. The OX26 antibody is not active in humans [689] or mice [663]. OX26-targeted pegylated immunoliposomes were prepared and the TfRMAb was bound to the surface of the liposome with a biotin-streptavidin bridge [893], as originally described by Huwyler and colleagues [897]. However, the BBB transport of the OX26-targeted liposomes was then evaluated in the human CMEC/D3 endothelial line in cell culture [893], where the OX26 antibody does not react with the human TfR [894].

If the TfRMAb is matched to the correct species, then successful RMT delivery of NPs to brain is possible. PLGA PNPs encapsulating the opioid tetrapeptide, endomorphin, were conjugated with the OX26 antibody, and the effect on analgesia was tested in rats [898]. Pegylated PLA PNPs were conjugated with the OX26 antibody and BBB transport was demonstrated by cerebral microdialysis in rats [899]. Pegylated mesoporous silica nanoparticles were conjugated with the rat RI7-217 antibody against the mouse TfR, and BBB transport was demonstrated in cell culture using the mouse bEND5 cells and in vivo in the mouse using fluorescent microscopy [900]. BBB transport of nanoparticles in the mouse was investigated with AuNPs conjugated with the rat 8D3 antibody against the mouse TfR [901]. The AuNPs could be visualized by electron microscopy at the luminal endothelial membrane, within intra-endothelial vesicles, and at the abluminal endothelial membrane [901]. It was hypothesized that the 8D3 conjugated AuNPs were not released by the abluminal TfR into the brain parenchyma [901]. However, it is not expected that gold particles in the post-vascular brain could be visualized at the EM level, owing to the volumetrics of the brain. As discussed in an early 1994 study on the EM of rat brain following carotid arterial infusion of a 5 nm gold-OX26 conjugate [585], the volume of the brain extracellular space, 200 µL/g, is 250-fold greater than the volume of the brain endothelial compartment, 0.8 µL/g. Therefore, the gold particles undergo a 250-fold dilution subsequent to exocytosis at the abluminal membrane.

**Insulin receptor antibody-targeted nanoparticles.** THLs were targeted with a MAb against the human insulin receptor (HIR), which cross reacts with the insulin receptor of Old World monkeys, and the HIRMAb-targeted THLs were encapsulated with a β-galactosidase expression plasmid. IV administration of these THLs produced widespread expression of the transgene in Rhesus monkey brain [902], as discussed in Section 10.2. The same HIRMAb was conjugated to polymersomes produced from an amphiphilic diblock copolymer, and endocytosis of the nanoparticles was followed by fluorescent microscopy in human hCMEC/D3 endothelial cells [903]. Another antibody against the HIR, the 29B4 antibody, was incorporated in albumin nanoparticles, and the 29B4 conjugated nanoparticles produced analgesia following IV administration in mice [904]. These results are difficult to interpret, because the 29B4 antibody is directed against the tyrosine kinase (TK) domain of the beta subunit of the HIR [905], and this TK domain is localized within the intracellular region of the HIR (Figure 10B). An antibody in plasma cannot access the epitope on the receptor that is localized within the intracellular compartment. Antibodies used as BBB RMT Trojan horses bind exofacial epitopes on the extracellular domain of the receptor, which are accessible to the blood-born antibody. The extracellular domains of either the HIR or the human TfR1 are shown in Figure 10.

**Transferrin- and lactoferrin-targeted nanoparticles.** Nanoparticles have been conjugated with transferrin (Tf) for brain drug delivery [906,907,908,909]. The problem with using Tf as a Trojan horse is that the exogenous Tf, conjugated to the nanoparticle, must compete with the endogenous Tf in plasma. The concentration of holo-Tf in plasma, 25,000 nM, is nearly 1000-fold greater than the concentration, 40 nM, of the TfR1 at the BBB [559]. Therefore, the BBB TfR is >99.9% saturated with endogenous Tf. For this reason, a TfRMAb Trojan horse, which binds the apical region of the TfR (Figure 10C), a site spatially removed from the holo-Tf binding site, is the preferred type of TfR Trojan horse. The other factor to consider in the use of Tf as a Trojan horse is the iron content of the Tf. Apo-Tf does not bind the TfR at physiological pH, and mono-ferric Tf binds the TfR with an 8- to 9-fold lower affinity than the di-ferric form of Tf [575]. Iron loading of Tf to produce di-ferric Tf has been described [586]. Lactoferrin (Lf) has been conjugated to polymeric nanoparticles for brain drug delivery [908]. The problem with using Lf as a Trojan horse is that Lf is a ligand for LRP1, and LRP1 is not expressed at the luminal endothelial membrane, as discussed in Section 8.2.1.

**Folic acid-targeted nanoparticles.** Stearic acid SLNs were incorporated with a conjugate of stearic acid and folic acid [910]. The folate conjugated SLNs incorporated docetaxel and ketoconazole, both highly water insoluble chemotherapeutic agents, which are favored agents for SLN delivery. The use of folic acid as a BBB Trojan horse assumes the folate receptor (FOLR1, Table 3) is the principal folate transporter at the BBB. Folate is also transported by the reduced folate carrier (FRC). There is evidence for expression at the BBB of both the FOLR1 [427], which is an RMT system, and the FRC [426], which is a CMT system, as discussed in Section 6.2.7. If FOLR1 is the primary folate transporter at the BBB, then folate conjugated nanoparticles may undergo RMT across the BBB. However, the FRC is a CMT system, and if FRC is the primary BBB transporter for folic acid, then it is unlikely that folate conjugated nanoparticles may traverse the BBB via very narrow cavity of a CMT system, as discussed in Section 9.5.1.

**Dual receptor targeting of nanoparticles.** The dual receptor targeting of pegylated liposomes was reported in 2002 using both the 8D3 antibody, for targeting the mouse TfR, and the 83-14 antibody, for targeting the HIR in an experimental intra-cranial human glioma model in the mouse [911]. These dual targeted THLs are discussed further in Section 10.2. Subsequently, sphingomyelin/cholesterol liposomes were dual targeted with the RI7-217 rat antibody against the mouse TfR, and phosphatidic acid, which targets the Abeta amyloid peptide of AD [912]. However, BBB transport of the liposomes was evaluated in culture with human hCMEC/D3 endothelial cells, which express the human TfR, and the human TfR is not recognized by the RI7-217 antibody [691]. Pegylated liposomes were dual targeted with the OX26 antibody against the rat TfR and the 19B8 antibody against the Abeta amyloid peptide of AD [913]. One antibody was thiolated and conjugated to a PEG terminal maleimide moiety, and the other antibody was biotinylated and conjugated with a streptavidin bridge to a PEG terminal biotin group [913].

**Peptide targeting of nanoparticles.** The 29AA rabies virus glycoprotein (RVG) peptide was conjugated to PLGA nanoparticles [914], based on the assumption the RVG peptide targets the nAChR on the BBB as suggested by Kumar et al. [297]. However, CNS uptake of the PLGA nanoparticles was not enhanced by the RVG peptide [914], which is consistent with the lack of expression of the putative RVG receptor, the nAChR, at the brain endothelium as discussed in Section 8.1.7. Pegylated SLNs were targeted with the apoE141-150 peptide, which corresponds to AA 141-150 of the human apolipoprotein E (P02649), on the assumption this ligand would trigger RMT across the LDLR at the BBB [915]. However, fluorescent microscopy showed the apoE-targeted SLNs did not cross the BBB [915], which is consistent with the lack of expression of the LDLR at the BBB in vivo, as discussed in Section 8.1.6. The same apoE141-150 peptide was conjugated to PLGA nanoparticles, and brain uptake of the nanoparticles was monitored by fluorescent microscopy [916]. The sequence of the apoE141-150 peptide is LRKLRKRLLR, which has a pI of >10. To the extent this peptide mediates BBB transport, the mechanism is most likely not RMT via a LDLR, but rather AMT owing to the highly cationic charge of the peptide.

In summary, NP functionalization with ligands that trigger RMT across the BBB is necessary because NPs alone do not cross the BBB, as reviewed in Section 9.2, Section 9.3 and Section 9.4. The importance of functionalization of nanomedicines for transport across vascular barriers has been recently reviewed [917]. Functionalization with ligands that promote NP transport via CMT is not effective, as the NPs cannot fit within the narrow transport cavities of a CMT, as reviewed in Section 9.5.1. Delivery of NPs via AMT is not optimal, owing to the high degree of sequestration of the NP within the endothelial compartment of brain following endocytosis via an AMT process, as reviewed in Section 9.5.2. The optimal approach to NP functionalization for brain drug delivery is the incorporation on the NP surface of receptor-specific MAbs that trigger RMT of the NP across the BBB. However, the TfR-specific MAbs, e.g., the rat 8D3 MAb against the mouse TfR, the rat RI7-217 MAb against the mouse TfR, or the mouse OX26 MAb against the rat TfR, are species-specific. The OX26 TfRMAb does not recognize either the mouse TfR [663] or the human TfR [689]. The RI7-217 TfRMAb does not recognize the human TfR [691], and has not been shown to bind with high affinity to the rat TfR. Therefore, it is important to match the species specificity of the TfRMAb with the species of the animal model or the species of origin of cells in culture.

#### 9.5.4. Brain Delivery of Nanoparticles with BBB Avoidance Strategies

Nanoparticles do not cross the BBB in the absence of a BBB delivery technology (Section 9.2, Section 9.3 and Section 9.4). In the absence of re-formulating the nanoparticles with a receptor-specific ligand to enable RMT at the BBB, one of the BBB avoidance strategies must be employed. These include convection-enhanced diffusion (CED) (Section 2.2.2), trans-nasal delivery (Section 3), and BBBD with focused ultrasound (Section 4.2). In an early study of CED and nanoparticles, camptothecin loaded PLGA PNPs were infused into the brain for 30 min by CED 7 days after implantation of a 9 L intra-cranial glioma in Fischer CD344 rats [918]. The infusion of a dose of 0.25 mg of the camptothecin had no effect on survival, although the infusion of a dose of 0.5 mg camptothecin in the PNPs increased median survival from 17 to 22 days. As reviewed in Section 2.2.2, clinical trials with the CED delivery system for brain conditions have failed. Therefore, in an effort to enhance drug distribution into the brain following CED, PLGA nanoparticles, with or without pegylation, were infused in the brain via CED in either isotonic (0.9%) or hypertonic (3%) saline [919]. The combination of pegylation of the PLGA PNPs, drug administration via CED, and the infusion of 3% hypertonic saline increased the distribution of the infusate in brain. This effect is presumed to be due to shrinkage of brain cells owing to osmotic fluid shifts in brain caused by the hypertonic saline infusion. Brain delivery of nanoparticles via the trans-nasal route was examined in mice following the nasal instillation of 5 µL/nostril of a microemulsion of clobazam [920]. However, clobazam is a lipid-soluble small molecule with a MW of 301 Da and low hydrogen bonding, and is a benzodiazepine that crosses the BBB [921]. As reviewed in Section 3, such molecules may gain access to brain following nasal instillation by first passing from the nose to the blood compartment and then entry into brain across the BBB. In another study of nasal delivery of nanoparticles, paclitaxel was formulated in PLGA PNPs and infused into the nose in large volumes of 25 µL in each nostril in the mouse with a pressurized olfactory device [922]. This volume of drug instilled is equal to the entire nasal volume in the mouse [148]. As reviewed in Section 3.2, this instillation of such a large volume causes local injury to the nasal mucosa. AuNPs were administered to mice in conjunction with BBBD induced by focused ultrasound (FUS) [923]. The size of the AuNPs varied from 3 nm to 15 nm to 120 nm. The dose of the microbubbles was 8 × 10^7^ per mouse IV, and the acoustic pressure used was 0.5–0.7 MPa. The highest distribution of AuNPs in brain was produced with the 15 nm AuNPs and an acoustic pressure of 0.7 MPa [923]. As discussed in Section 4.2, the FUS/microbubble form of BBBD creates pores at the BBB owing to opening of tight junctions. The 120 AuNPs may have a diameter that exceeds the transitory pores in the BBB caused by the FUS/microbubble procedure. BBBD caused by the FUS/microbubble treatment can lead to neuropathology (Section 4.2). As discussed below, nanoparticle administration is not without toxicity, as discussed below. Therefore, the NP toxicity would be additive with a toxicity profile of the BBB avoidance strategy, such as CED, nasal administration of large volumes, or FUS/microbubble BBB disruption.

### 9.6. Nanoparticle Clinical Trials for the Brain

The NP-based pharmaceuticals that are FDA approved are nearly all liposomal formulations, and none are approved for the CNS [924]. FDA-approved formulations include:

- Pegylated and non-pegylated liposomes encapsulating cancer chemotherapeutic agents including doxorubicin, cytarabine/daunomycin, vincristine, irinotecan;
- Liposomes encapsulating amphotericin B for fungal infections;
- Liposomes encapsulating verteporfin for macular degeneration;
- Cremophor-free paclitaxel re-formulated as albumin nanoparticles for cancer;
- siRNA in cationic pegylated liposomes for hereditary transthyretin amyloidosis;
- Iron replacement therapies;
- Imaging agents.

Additional liposomal agents are in clinical trials as reviewed recently [924]. However, few of these clinical trials are designed for CNS indications, with some exceptions [925]:

- SGT-53 was developed as a treatment for brain cancer [926]. SGT-53 is a plasmid DNA encoding the p53 tumor suppressor gene that is adsorbed to cationic liposomes conjugated with a ScFv antibody against the human TfR [926]. This ScFv was derived from the 5E9 antibody [927], also known as the HB21 antibody [928]. The ScFv against the human TfR was chemically conjugated to the liposomal lipids with a thio-ether linkage. SGT-53 was administered to patients with recurrent glioblastoma multiforme (GBM) concurrent with temozolomide treatment (NCT02340156). Only one patient was enrolled and the trial was terminated. The SGT-53 formulation is a cationic lipoplex of DNA, and such agents demonstrate aggregation problems, as discussed in Section 10.2.
- MTX-110 is a complex of panobinostat, a histone decarboxylase inhibitor, and hydroxylpropyl β-cyclodextrin [929]. MTX-110 is a soluble form of panobinostat, which is poorly soluble in water. MTX-110 does not cross the BBB, and this formulation has been administered to rats by CED [929] and to primates by infusion in the fourth ventricle [930]. MTX-110 was administered to patients with a pontine glioma by CED; the phase 1 trial in 7 patients concluded in February 2022, with no advancement to phase 2 (NCT03566199).
- ARCT-810 is a mRNA encoding ornithine transcarbamylase (OTC) formulated in a LNP for the treatment of late onset OTC deficiency. This condition can lead to seizures, brain edema, and death [931]. The ARCT-810 clinical trial was initiated in 2020 and is ongoing (NCT04442347). The details of ARCT-810 manufacturing are not available, and it is not clear if this was formulated as a lipoplex/RNA mixture or if the mRNA was fully encapsulated in the LNPs.
- CNM-Au8 is a preparation of gold nanocrystals which are daily administered orally at a dose of 30 mg, and were tested in a phase 2 trial for ALS [932]. The trial was completed in 2022 and no results were yet reported (NCT04098406). It is not clear how such AuNPs, which are not functionalized, can cross the BBB in ALS. The BBB is intact in ALS [933].
- ABI-009 is a preparation of albumin NPs complexed with the macrolide antibiotic, rapamycin, an anti-tumor agent, which is administered to patients with newly diagnosed GBM (NCT03463265). The trial was first posted in 2018, and no results have been reported. Since the albumin NPs are not functionalized, no transport across the intact BBB is expected. The BBB may be leaky in the tumor area of GBM to small molecules [191]. However, much of the GBM tumor retains an intact BBB, and tumor eradication is not possible unless all cancer cells within the tumor are exposed to the therapeutic [934]. Therefore, new treatments for GBM need to be formulated or engineered to enable transport across an BBB.
- NU-0129 is an AuNP conjugated with siRNA and designated a spherical nucleic acid (SNA) [935]. The siRNA targets the Bcl1Like12 oncogene [935]. NU-0129 is said to be BBB-penetrating, but the AuNP is not functionalized. Only the gold part of this NU-0219 was tested for brain penetration, not the siRNA part. The siRNA was simply adsorbed to the surface of the AuNP, and there is immediate separation of the AuNP and the siRNA following IV administration [935]. The plasma T_1/2_ of the siRNA is 5.4 ± 5.1 min, whereas the plasma T_1/2_ of the gold is 17 ± 6 h [935]. A phase 1 trial in recurrent GBM was initiated for NU-0129 in 2017 with the last posting in 2020 and no study results are available (NCT03020017).

In summary, there are no nanoparticle formulations FDA approved for CNS diseases, and based on the ongoing clinical trials reviewed above, this situation is not likely to change in the near future. The challenge with nanoparticles for the brain is the same as that for biologics for the brain—FDA approval is unlikely unless the biologic, or the nanoparticle, is re-engineered to enable transport across an intact BBB, preferably via an endogenous BBB RMT pathway.

### 9.7. Nanoparticle Neurotoxicology

There are several reviews on the neurotoxicology that follows from the accumulation of nanomaterials in the brain [936,937,938,939,940]. The greatest toxicity is observed with the chronic administration of either metallic NPs or CNTs/fullerenes. With respect to metallic NPs, toxicity is found after the administration of AuNPs [941,942,943], AgNPs [944,945], iron NPs [946], silica NPs [947], and titanium NPs [948]. Pregnant mice were fed AgNPs orally from the first to last day of gestation [944]. The silver content of brain increased 14-fold and 22-fold following the feeding of 30 nm and 10 nm AgNPs, respectively, which was associated with increased gene expression of inflammatory cytokines and impaired cognition [944]. Similar findings were made in rats fed AgNPs [945]. Silver ions may gain access to brain from blood similar to mechanisms that mediate the brain uptake of copper ions. Internal carotid perfusion studies show that copper gains access to brain via carrier mediated transport at the BBB of the free copper ion [949]. The SLC31 sub-family encodes for copper transporters (CTR) [950].

Pegylated graphene oxide (GO) nanosheets are toxic to cells following partial insertion in the cell membrane, which triggers an inflammatory response [951]. The intra-cerebral injection of C60 fullerenes into the hippocampus reduces neurotrophic factors and causes neuro-behavioral changes [952]. CNTs, including SWCNTs or MWCNTs, are particularly toxic to vascular cells both in brain and peripheral tissues [938]. Reduced graphene oxide NPs with an average diameter of 340 nm caused BBB disruption following an IV injection of 7 mg/kg in rats, and electron microscopy showed leaky tight junctions [953].

In addition to metallic NPs, and CNT/fullerene/GO nanoparticles, polymeric NPs may also cause toxicity in brain. PBCA NPs coated with polysorbate-80 (PS80) were the first NPs to be shown to cross the BBB [64], as reviewed in Section 9.2.1. The PBCA NPs were said to cross the BBB on the basis of analgesia induced by dalargin loaded nanoparticles [64]. Nanoparticle administration induced dalargin analgesia only if PS80 was added to the formulation. Subsequently, it was shown that the addition of PS80 causes rapid desorption of the opioid peptide from the PBCA NPs [875]. The impact of PS80-coated PBCA NPs on BBB integrity was examined with an in vitro BBB model [954]. The addition of PS80-coated PBCA NPs to the endothelium caused a dose-dependent disruption in the BBB, as measured by trans-endothelial electrical resistance (TEER). BBB disruption induced by the PS80-coated PBCA NPs caused enhanced flux across the endothelial monolayer of sucrose or albumin, which do not cross an intact BBB [954]. Significant toxicity was observed following the IV administration of PS80-coated chitosan NPs [955]. The PS80-coated chitosan NPs were demonstrated to cross the BBB by external fluorescent microscopy. Daily administration of 3–30 mg/kg of the NPs caused a dose-dependent decrease in body weight in rats [955]. A microscopic examination of the brain showed apoptotic and necrotic neurons and reduced GFAP reactive cerebellar astrocytes [955].

In summary, both polymeric and non-polymeric NPs may prove to have a significant toxicity profile with chronic administration. The toxicity of a pharmaceutic following long-term, e.g., 6 months, administration is generally not examined in detail until an Investigational New Drug (IND) application is submitted to the FDA to seek approval for a human phase 1 clinical trial [956]. The safety pharmacology and toxicology performed under Good Laboratory Practice (GLP) procedures in either a primate, or two lower species, is a required component of an IND. Only a few nanoparticle formulations for the brain have been tested in a clinical trial, as reviewed in Section 9.6. Therefore, few GLP safety pharmacology and toxicology evaluations of long-term nanoparticle administration have been performed.

An IND application also requires demonstration of a scalable manufacturing process under Good Manufacturing Practice (GMP) or ‘clean room’ conditions [957]. Few nanoparticle formulations for the brain have passed the rigors of a scalable GMP manufacturing process, or the demonstration of long term, e.g., 2-year stability with storage. The challenges in scalability, process development, fill/finish, in-process testing, and release testing required for human pharmaceutics will also have to be mastered for nanoparticle drugs for the brain, as recently reviewed [958]. A limiting problem for nanoparticles, which also limits development of liposomal formulations, is both (a) poor loading of the nanoparticle with drug, and (b) leakage of loaded drug from the nanoparticle on storage [959]. Doxorubicin is one of the few small molecules to be commercialized as a liposomal formulation, and a reason for this is that doxorubicin precipitates inside the liposome [960], which eliminates leakage on long term storage. Doxorubicin forms covalently bonded dimers in aqueous solution, which causes precipitation of the drug in the aqueous interior of the liposome [961].

## 10. Gene Therapy of the Brain

### 10.1. Viral Gene Therapy of Brain

#### 10.1.1. Lentiviral-Transfected Stem Cells

The lentivirus (LV) is a retrovirus, which permanently integrates into the host genome. Therefore, there is a risk of long-term cancer with this virus [962]. Following introduction of the expression cassette encoding the therapeutic gene into the LV genome, hematopoietic stem cells (HSC) are permanently transfected ex vivo, and this transfection is quantified by determination of the vector copy number (VCN), which is the number of LV genomes inserted into the HSC. So as to reduce the risks of insertional mutagenesis, the FDA requires a VCN <5 [294]. Mutations in the gene encoding the lysosomal enzyme, arylsulfatase A (ASA), causes metachromatic leukodystrophy (MLD), and nine MLD patients were treated with the LV-HSC-ASA gene therapy [963]. Long term follow-up showed normal ASA enzyme activity in peripheral blood lymphocytes and in CSF [964], as measured with an enzymatic fluorometric assay using 4-methylumbelliferyl sulfate (4-MUS) as the substrate [965]. These enzyme activity results are difficult to evaluate, because 4-MUS is hydrolyzed by arylfulfatase B and other O-sulfatases, and is not specific for ASA. A preferred surrogate marker would be immunoreactive ASA as determined by ELISA or similar methodology. The CSF data are difficult to assess, as any sulfatase in plasma would be expected to pass the blood–CSF barrier to enter CSF, as discussed in Section 2.1.2. The underlying difficulty with the HSC-LV approach is the lack of stem cell transport across the BBB, as discussed in Section 5.1.

#### 10.1.2. Adenovirus

Adenovirus (AV) is a common cold virus, and the majority of the human population has a pre-existing immunity to AV [966]. AV was evaluated as a vector for delivery of a transgene gene to the brain following intra-cerebral injection of the virus. In 1993, replication deficient AV encoding β-galactosidase was injected directly into rat brain, and gene expression was followed by β-galactosidase histochemistry [967]. The β-galactosidase transgene expression persisted for 45–60 days. However, by 1999, the AV virus was shown to produce an inflammatory reaction in brain, including microglial activation, astrogliosis, and demyelination in rats [968] and primates [969]. Additionally, in 1999, a patient with partial ornithine transcarbamylase (OTC) deficiency received a high intravenous dose, 3.8 × 10^13^ particles, of AV encoding the OTC gene, and this treatment proved to be fatal [970]. These events related to the toxicity of AV gene therapy suppressed the enthusiasm for the use of the AV vector in gene therapy.

#### 10.1.3. Herpes Simplex Virus

Herpes simplex virus type 1 (HSV1) is a common virus causing cold sores [971], and the majority of humans have a pre-existing immunity to the virus [972]. In 1989, a recombinant HSV-1 encoding the gene for human hypoxanthine-guanine phosphoribosyl transferase (HPRT) was injected into mouse brain, and this resulted in human HPRT expression in the brain [973]. However, by 1994, the toxicity of HSV1 administration to brain was demonstrated, as the intra-cerebral injection of a replication deficient HSV1 amplicon in the rat caused a robust neuro-inflammatory reaction [974]. This was confirmed with intra-cerebral injections in either rats or mice of replication deficient HSV1, and the neuro-inflammation was associated with systemic illness and significant weight loss [975]. Subsequently, HSV1 was engineered as an oncolytic virus, which selectively replicated in tumor cells, but not normal cells, and this variant was designated the G207 HSV1 [976]. A phase 1b clinical trial for recurrent GBM tested the tumoricidal effects of the G207 variant, and in this application, the G207 virus carried no transgene [977]. A dose of 10^8^ plaque forming units (pfu) of the virus was injected in a volume of 1 mL in the cavity of brain created by the tumor resection in patients with brain cancer [977]. The trial did not proceed further, but a genomic analysis of tumor biopsies was recently reported [978]. A potential problem with this approach is that the penetration of the virus into brain from the tumor cavity is limited by diffusion. GBM is notorious for microscopic spread into normal brain beyond the tumor cavity [979]. This microscopic extension to distant parts of brain, which cannot be visualized by imaging methods, is a major reason for the dismal prognosis of GBM.

#### 10.1.4. Adeno-Associated Virus

**Brain gene delivery via intra-cerebral injection.** Adeno-associated virus (AAV) expressing the lacZ β-galactosidase gene was injected directly into the brain of rats and expression persisted for about 4 months [980]. The problems pertaining to diffusion limitation with the intra-cerebral (IC) route for brain drug delivery are discussed in Section 2.2.1. The same problem pertains to brain gene delivery via intra-cerebral route, which is that the transgene is only expressed at the injection site [980,981]. In an attempt to increase transgene penetration into brain following intra-cerebral injection, the AAV was co-injected in 1.1 M mannitol [981], which causes osmotic shrinkage of brain cells. This potentially toxic formulation caused a modest improvement in the penetration of the transgene into brain from the injection site [981]. The futility of the IC route was manifest in the design of a 2010 clinical trial of CLN2 disease, where the AAV2 encoding the TPP1 enzyme was injected into 12 sites of the cerebrum through 6 burr holes [982]. This multiple IC-injection approach to brain gene delivery is reminiscent of the multiple catheter bundles proposed for either IC [126] or CED [140] drug delivery to brain, discussed in Section 2.2.1 and Section 2.2.2, respectively.

**Figure 16:**
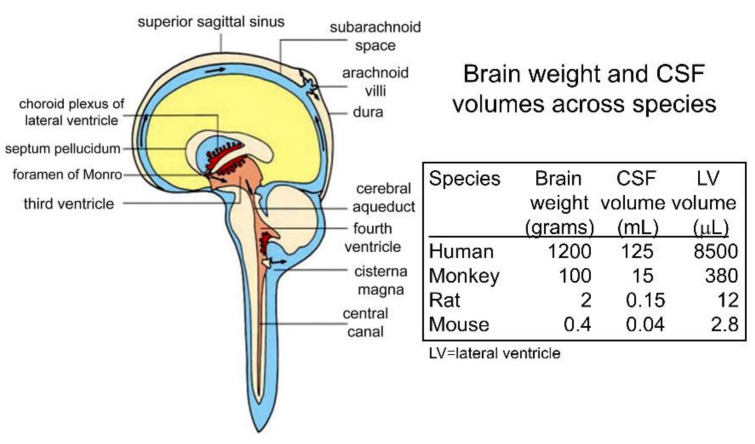
**CSF flow and volume in humans and animals.** (**Left**) CSF, shown in blue or brown, is produced at the choroid plexus lining the ventricles (red) and flows around the surface of the brain or spinal cord, and is absorbed into the venous blood of the superior sagittal sinus at the arachnoid villi. The septum pellucidum separates the 2 lateral ventricles into separate compartments. The cisterna magna is at the base of the cerebellum next to the brain stem. Reproduced with permission from [984], Copyright© 2016, Elsevier. (**Right**) The brain weights, total CSF volume, and lateral ventricle (LV) volumes for humans, monkeys, rats, and mice are shown. CSF volumes are from [985], and the LV volumes are from [74], for the rat, from [986], for the mouse, from [987], for the monkey, and from [988], for humans.

**Brain gene delivery via intra-thecal injection.** In an attempt to achieve broader distribution of the transgene to brain, the intra-thecal injection into CSF was performed [983]. The CSF flow tracts in the brain are shown in Figure 16.

AAV injection via the intrathecal route was proposed so as to “inject viral vectors directly into the cerebral lateral ventricles and allow the natural flow of the CSF to deliver the virus throughout the CNS” [983]. One could draw support from this hypothesis from the work of Mott 1913 [26], as the prevailing view over a 100 years ago was that nutrients in blood passed first into CSF from blood and then to brain, as discussed in Section 1.2. It is now known that drugs injected into the CSF do not flow throughout the CNS, as reviewed in Section 2.1.1 and illustrated in Figure 5. For ICV brain gene delivery, an AAV2 vector encoding GUSB was injected in a volume of 2 µL in both lateral ventricles of mice on the day of birth [983]. The volume of the lateral ventricle of an adult mouse is 2.8 µL [986], as shown in Figure 16. The brain weight of an adult mouse, 433 mg, is 6-fold greater than the brain weight of a newborn mouse, 73 mg [989]. A conservative estimate of the volume of the lateral ventricle in the newborn mouse is 0.6 µL. Therefore, the injection of 2 µL into each lateral ventricle of a newborn mouse is >300% of the ventricular volume. As discussed in Section 2.1.1, the injection of such large volumes of fluid in the lateral ventricle forces fluid into the brain via perivascular spaces, which is an artifact of the high injection volume.

Intrathecal (IT) delivery of viral gene therapy vectors can access the CSF via a lumbar injection, an ICV injection into a lateral ventricle, or injection into the cisterna magna (CM) at the base of the brain (Figure 16). MRI in primates shows an ICV injection preferentially delivers drug to the surface of the cerebrum with minimal distribution to the caudal portion of the spinal cord [990]. Injection into the lumbar CSF compartment results in distribution to the surface of the spinal cord with minimal distribution to the cerebrum [990]. Injection into the CM produces maximal distribution to the surface of both the cerebrum and the spinal cord [990]. However, an intra-cisternal injection in humans poses significant safety considerations, owing to the proximity of the CM to the vital structures of the brain stem (Figure 16). Another factor complicating the intra-cisternal route of brain gene delivery is that the volume of the CM in humans varies depending on individual neuroanatomy, and can range from near 0 to a maximal mean volume of 1.1 mL [991]. The CM volume in 60% of humans is only 0.35 mL [991].

Intra-thecal delivery of a AAV8 or AAV9 encoding IDUA was performed in primates via an intra-cisternal injection [992]. The injection volume, 0.5 mL, greatly exceeds the volume of the CM of the primate, which has a brain weight <10% of the human brain. As discussed in Section 2.2.1, drug injection into the CSF is akin to a slow IV infusion, and it is expected that AAV will rapidly move from the CSF compartment to the blood. The diameter of AAV is only 25 nm [993], and particles up to 7 microns pass the arachnoid villi to move from CSF to blood [68]. Injection of AAV into the CSF results in the formation of antibodies against both the AAV capsid protein, as well as to the protein product of the transgene. Intra-thecal delivery of AAV-IDUA in monkeys caused the formation of anti-IDUA antibodies that were found in both plasma and CSF [992]. The antibody formation in the periphery follows from movement of the AAV from CSF to blood. The peripheral anti-IDUA antibodies then move from plasma to CSF similar to any IgG in blood, as discussed in Section 8.3.4. The percent of cells transfected with the IDUA gene in brain varied from 1% to 30% depending on the region of brain [992]. Intra-thecal gene delivery is said to be advantageous over intravenous delivery of AAV9 to brain, as the injection dose of the AAV is lower with the intra-thecal route as compared to IV administration [992]. The ID of intravenous AAV9 for SMA is 1.1 × 10^14^ vg/kg in humans [994], and there is potential hepatotoxicity from such a high dose, as discussed below. The ID used in an ongoing clinical trial of MPS2 with intrathecal AAV-IDS is 6.5 × 10^10^ vg/g brain (NCT04571970). Given a 20 kg child with a 1000 g brain, this ID is equivalent to 3.3 × 10^12^ vg/kg, which is more than a log order reduction in virus exposure to the patient, as compared to the IV route. However, the problem with the CSF route of AAV delivery to brain is the same as the CSF route for any pharmaceutical. The intrathecal route results in AAV delivery only to the CSF surface of the brain, as discussed in Section 2.1 (Figure 5), and results in AAV movement from CSF to the blood compartment, as discussed in Section 2.1.2.

**Brain gene delivery via intravenous injection.** In 2009, AAV9 was shown to penetrate the BBB following IV administration in the 1-day-old and 70-day-old mouse [995]. The form of AAV9 used in this study was the self-complementary AAV or scAAV. The ID in the newborn mouse, which weighs 1–2 g, was 4 × 10^11^ vg, which is equivalent to 2.7 × 10^14^ vg/kg. Although neurons were transduced in brain of the newborn, astrocytes were the principal site of transduction in the 70-day-old mouse [995]. These findings were replicated in the primate following the IV injection of 1–3 × 10^14^ vg/kg of a scAAV9 encoding green fluorescent protein (GFP) [996]. GFP expression was higher in grey matter as compared to white matter [996], which is consistent with the higher vascular density in gray matter as compared to white matter [997]. The survival motor neuron 1 (SMN1) gene is mutated in spinal muscular atrophy (SMA). An scAAV9 encoding SMN1, and designated Zolgensma^®®^, or onasemnogene abeparvovec-xioi, was FDA approved in 2019 for treatment of SMA as a one-time IV administration of 1.1 × 10^14^ vg/kg of [994]. Zolgensma^®®^ is a scAAV as opposed to a single stranded AAV or ssAAV. The size of the expression cassette encoding the therapeutic gene, which includes the promoter, any 5′-untranslated region (UTR) and the 3′-UTR, is limited to <2.3 kb for scAAV, but is limited to <4.7 kb for ssAAV [998]. The scAAV is more effective as a brain delivery vector than the ssAAV [998]. An IV injection dose of 4 × 10^13^ vg/kg of scAAV or ssAAV transduces only 12% and 2% of neurons in brain, respectively [998]. These results indicate the transport of AAV9 across the BBB is not very efficient, which is why a high ID of Zolgensma, 10^14^ vg/kg, is required for the IV treatment of SMA. New variants of AAV9 are being developed, which produce higher rates of transduction in brain following IV administration.

**New AAV variants.** AAV9 with mutated capsid protein, and designated ssAAV9-PHP.B, produce higher rates of neuronal expression of a GFP transgene, as compared to non-mutated ssAAV9 in the mouse [999]. The intravenous ID required for broad transgene expression in brain is still high, at 4 × 10^13^ vg/kg [999]. The new variants, PHP.B or PHP.eB, gain access to the mouse brain via a novel AAV receptor, lymphocyte antigen 6 family member a (Ly6a) [1000]. However, the injection dose of the new variants is still high at 2 × 10^14^ vg/kg [1000]. These new variants of AAV may be only effective in the mouse, as humans lack the Ly6a receptor [1001]. Recently, the Ly6a human homologue, Ly6e, has been proposed as a novel AAV9 receptor at the human BBB [1002], although this has yet to be experimentally confirmed.

**AAV immunogenicity.** AAV is a small 25 nm DNA parvovirus, which is non-pathogenic, but is infectious, and 60–70% of the human population has a pre-existing immunity to AAV [1003,1004]. The anti-AAV antibodies in humans include neutralizing antibodies (NAb), which can lead to rapid inactivation and clearance of the virus. A single injection of AAV in humans produces a high titer NAb response with long-lasting immunity [1004]. The immune response can cross react with the protein produced by the transgene inserted in the AAV backbone, and this immune response against the therapeutic protein is observed following intravenous or intrathecal administration of the AAV. Monkeys were injected intravenously with AAV9 encoding human NAGLU, and an immune response against the viral capsid protein developed over the ensuing weeks [1005]. A NAb response also developed against the endogenous NAGLU enzyme, which caused a >10-fold decrease in serum NAGLU enzyme activity in some animals [1005]. In another primate study, the AAV was administered by intrathecal injection in the cisterna magna. If the AAV encoded a foreign protein, then a severe immune response was generated, which resulted in ataxia and pathologic changes in the nearby cerebellum [1006], which is contiguous with the cisterna magna (Figure 16). Conversely, if a self-protein was encoded in the AAV, no immune response was observed [1006]. The AAV capsid protein is effectively acting as an immune adjuvant for the transgene product. This could be a problem in the treatment of children with genetic disease secondary to nonsense mutations, wherein no endogenous protein is ever produced. The immune response against the AAV could trigger an immune response against the transgene product, which the immune system recognizes as a foreign protein. The intrathecal injection of AAV encoding human IDUA in monkeys produced an immune response against the IDUA enzyme, if the animals were not pre-tolerized by prior exposure of a liver directed AAV8-IDUA [992]. IDUA enzyme activity in CSF was reduced in the non-tolerized animals [992].

The issue of AAV immunity will become a primary concern when AAV-mediated gene expression terminates at some period following the initial single administration of the AAV. AAV exists within the cell as an episome, and while AAV gene expression may last for some years, it is expected that the patient will need subsequent courses of treatment in future years. Zolgensma^®®^ is approved only for a single use in the treatment of SMA. Significant questions remain in this area. When will a second and third dose be required? What type of immune response will the second or third doses generate? Will long last immunity against AAV result in prompt neutralization of future doses? Persistent T cell immunity against the NAGLU enzyme, which is mutated in MPSIIIB, has been confirmed in subjects treated with a single course of AAV-NAGLU by intra-cerebral injection [1007].

**AAV hepatotoxicity.** AAV is a hepatotropic virus [1008]. Patients treated with IV Zolgensma^®®^ develop abnormal liver function tests in 90% of subjects treated, and many require corticosteroid treatment [1009]. Zolgensma^®®^ is administered as a high injection dose of 10^14^ vg/kg [994]. This dose, 10^14^ vg/kg, when administered to newborn mice, produces hepatocellular carcinoma in about 70% of mice observed long term [1010]. These findings confirm an early study describing the generation of hepatocellular carcinoma in AAV-treated mice [1011].

**AAV neurotoxicity.** AAV induces an inflammatory response in the CNS as recently reviewed [1012]. The injection of AAV9-IDUA into the cisterna magna of primates produces a mononuclear pleocytosis in CSF and degenerative changes in the dorsal root ganglion [1013]. AAV8 was injected bilaterally in the thalamus in primates at a dose of 10^11^–10^12^ vector genomes, and this produced severe white matter and gray matter necrosis along the injection track [1014]. In an important study pointing to the role of the inverted terminal repeats (ITRs) of the recombinant AAV, cerebellar neurotoxicity was observed in primates following AAV injection into this region of brain [1015]. This cerebellar toxicity was not observed in rodents [1015], which points to the importance of primate studies in the safety pharmacology and toxicology evaluation of new AAV gene products. The ITRs are 145 bp elements placed at both the 5′- and 3′-ends of the expression cassette [1016]. The ITRs can exert promoter activity on cross-packaged material present in the AAV formulation [1015,1016].

The use of AAV in brain gene therapy is a global enterprise with over 3000 citations in PubMed using the search term, ‘adeno-associated virus and brain’ (March 2022). The development of AAV serotypes that cross the BBB following IV administration is an advance over intrathecal or intra-cerebral routes of administration. Nevertheless, this is a field shadowed by potential long-term complications, including potential liver cancer, severe immune reactions from future repeat treatments, and neuropathologic side effects. Given these issues, it is important to develop, in parallel, non-viral plasmid DNA-based gene therapy of the brain.

### 10.2. Non-Viral Gene Therapy of Brain

#### 10.2.1. Cationic Liposomes and Cationic Polyplexes

Lipofection is the process of delivery of plasmid DNA into cultured cells following the mixing of the anionic DNA with a cationic lipid, and this was first described in 1987 [1017]. The cationic liposomes were formed from a 1:1 mixture of a cationic lipid, N-[1-(2,3,-dioleyloxy)propyl]-N,N,N-trimethylammonium chloride, also known as DOTMA, and a helper lipid, dioleoyl phosphatidylethanolamine, also known as DOPE, and these agents, or variants, are still used today, and widely known as Lipofectamine^®®^. Lipofection is performed in many labs to produced transgene expression in cultured cells. However, the translation of in vitro lipofection to gene therapy in vivo proved to be difficult. Following IV administration of a reporter gene complexed with cationic liposomes, the transgene was effectively expressed only in the lung, as transgene expression in this organ was several log orders greater than transgene expression in liver or other organs [1018,1019,1020,1021]. Plasmid DNA lipoplexes are formulated in water, or saline-free buffered solutions of low tonicity, and have a diameter of ~100 nm. However, when DNA lipoplexes are transferred to physiologic saline, the structures aggregate into micron-sized particles, and precipitate overnight [835]. The saline-induced aggregation explains why lipofection is so successful in cultured cells, and why lipofection is unsuccessful in vivo. The saline induced aggregation triggers uptake by cultured cells via phagocytosis [1022]. Some cultured cell lines are difficult to lipofect if the cell line has a low level of phagocytosis [1023]. While this aggregation of DNA lipoplexes is useful for cell culture, the aggregation limits the utility of in vivo gene therapy with cationic liposomes. Following the in vivo injection of the DNA lipoplexes, these aggregate immediately and embolize in the first vascular bed encountered after an IV administration, which is the pulmonary circulation [1018,1019,1020,1021]. A histological exam of lung shows the transgene is only expressed in the pulmonary endothelium [1018]. The IV administration of DNA lipoplexes produces an inflammatory response and elevated cytokines [1024], which is due largely to the DNA component [1025]. Cationic liposomes do not cross the BBB [1026], and must be injected directly into the brain to produce transfection of brain cells [1027].

The cationic lipid can be substituted with cationic polymers, such as polyethylenimine (PEI) [1028]. PEI DNA polyplexes have the same properties as lipid DNA polyplexes, and transfect essentially only the lung after IV administration, where gene expression is 2–3 log orders higher than in liver [1028]. In cell culture PEI-mediated transfection correlates with the size of the aggregates formed when PEI/DNA complexes are added to physiologic saline [874]. Aggregate size is larger if linear PEI is used as compared to branched PEI [874].

The first example of receptor-targeting of a DNA polyplex described the complexation of a chloramphenicol acetyltransferase expression plasmid DNA to a polycationic polymer, poly-L-lysine (PLL), which was conjugated to asialoorosomucoid (ASOR), a ligand for the liver asialoglycoprotein receptor [1029]. A large dose of plasmid DNA, 5 mg/kg, complexed to the PLL-ASOR was injected intravenously in rats, and chloramphenicol acetyltransferase gene expression in liver was observed. However, as with all cationic polyplex/DNA complexes, the PLL/DNA complex aggregated in physiologic saline [1030], which aborted further development of this form of non-viral gene therapy for humans.

#### 10.2.2. Pegylated Liposomes

A detergent dialysis method was used to encapsulated plasmid DNA in the interior of pegylated liposomes, also called stabilized plasmid–lipid particles, or SPLP [1031]. The SPLPs do not aggregate in vivo, and do not target the lung [1032]. However, SPLPs lack any targeting ligand, and do not produce efficient gene expression in vivo. A luciferase expression plasmid DNA was encapsulated in SPLPs and injected in mice bearing a Neuro-2a flank tumor. Luciferase gene expression in the flank tumor was 2 log orders of magnitude higher than in liver, spleen, or lung [1032], which is consistent with the known accumulation of pegylated liposomes in mouse flank tumors. These tumors have a leaky vasculature with open endothelial clefts and discontinuous basement membrane [1033]. Luciferase gene expression in the flank tumor was 100 pg/g [1032], which is equivalent to 1 pg/mg protein, given 100 mg protein per gram tissue [1034]. Luciferase gene expression in mouse organs (lung, spleen, liver) was very low, ≤0.01 pg/mg protein. In contrast, as discussed in the next section, Trojan horse liposomes (THLs), which are receptor-targeted pegylated liposomes, produce much higher levels of transgene expression in vivo following IV administration. A THL, also called a pegylated immunoliposome, is a pegylated liposome, where the tips of some of the polyethyleneglycol (PEG) strands are conjugated with a receptor-specific MAb. THLs targeted with a MAb against the human insulin receptor (HIR), and encapsulating a luciferase expression plasmid, produced levels of luciferase enzyme activity in the liver, brain, spleen, and lung of 16, 9, 3, and 2 pg/mg protein, respectively, in the Rhesus monkey [902].

#### 10.2.3. Trojan Horse Liposomes

The in vivo delivery and brain expression of a plasmid DNA was reported in 2000 using pegylated immunoliposomes, also called Trojan horse liposomes (THLs) [1035]. In this approach, a plasmid DNA is encapsulated in the interior of a pegylated liposome, and the tips of some of the PEG strands on the surface of the liposome are conjugated with a MAb that targets a RMT system at the BBB such as the TfR or insulin receptor. The incorporation of the receptor targeting ligand or MAb on the surface of the liposome is essential for delivery into brain in vivo following IV administration, as non-functionalized pegylated liposomes do not cross the BBB [891,1035].

Despite the requirement for a receptor targeting ligand on the surface of pegylated liposomes for plasmid DNA delivery [1036], the field of liposome-mediated delivery of nucleic acid therapeutics has evolved without a major emphasis on the incorporation of a targeting ligand in the liposome. A recent review of nucleic acid delivery with lipid nanoparticles (LNP), which is a generic term for pegylated liposomes, makes no reference to the need for functionalization of the LNP with a targeting ligand to enable receptor-mediated uptake into cells, apart from the presumed coating of the surface of the LNP with apoE in plasma [1037]. The incorporation of short chain pegylated lipids on the surface of the LNP is advocated, so as to facilitate rapid dissociation of this PEG-lipid in vivo in plasma [1037]. The dissociation of the pegylated lipid from the surface of the liposome is said to enable fusion of the liposome with the plasma membrane, which then initiates endocytosis of the nucleic acid encapsulated in the liposome into the cell [1037]. This approach of deploying short chain pegylated lipids, so as to enhance dissociation of the PEG lipid in vivo, is opposite of the design of THLs, where long chain pegylated lipids are incorporated on the surface of the liposome for conjugation of the targeting MAb at the tip of the pegylated lipid [1036]. Early dissociation of the lipid-PEG-MAb from the surface of the THL would eliminate RMT of the THL across the BBB. The incorporation of the receptor targeting ligand or MAb on the surface of the liposome is essential for delivery into brain in vivo following IV administration, as non-functionalized pegylated liposomes do not cross the BBB [891,1035].

**Figure 17:**
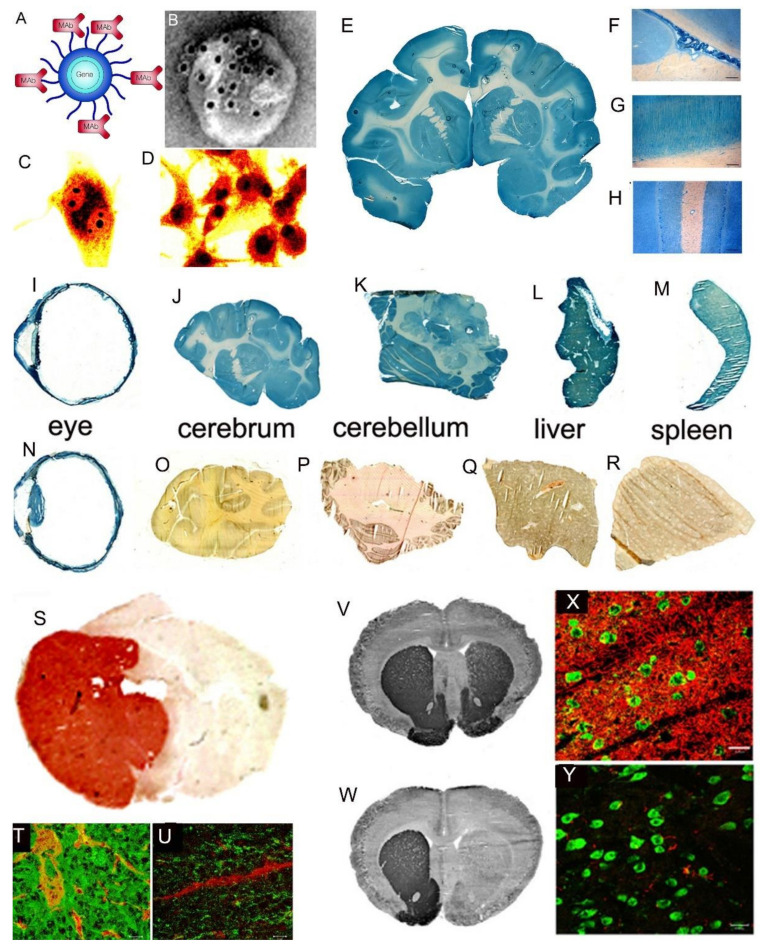
**Trojan horse liposomes and non-viral gene therapy of the brain.** (**A**) Model of a THL showing single plasmid DNA encapsulated in interior of pegylated liposome, where the tips of a small fraction of the surface PEG strands are conjugated with a receptor-specific MAb. Reproduced with permission from [1038], Copyright© 2002 Springer-Nature. (**B**) Electron micrograph of a THL co-incubated with secondary antibody conjugated with 10 nm gold particles. Reproduced from [1039]. (**C**,**D**) Confocal microscopy of U87 human glioma cells after 6 h (**C**) or 24 h (**D**) incubation with HIRMAb-targeted THLs encapsulating fluorescein conjugated plasmid DNA. Reproduced with permission from [1040], Copyright© 2002 John Wiley & Sons. (**E**) Beta galactosidase histochemistry of coronal section of brain from Rhesus monkey removed 48 h after the IV administration of 12 ug/kg of pSV-lacZ expression plasmid DNA encapsulated in HIRMAb-THLs. (**F**–**H**) Beta galactosidase histochemistry of choroid plexus (**F**), occipital lobe (**G**), and cerebellum (**H**) of brain shown in (**E**). (**I**–**R**) Beta galactosidase histochemistry of Rhesus monkey eye (**I**), cerebrum (**J**), cerebellum (**K**), liver (**L**), and spleen (**M**) at 48 h after the IV administration of HIRMAb-targeted THLs encapsulating a lacZ expression plasmid DNA under the influence of the widely expressed SV40 promoter, and of Rhesus monkey eye (**N**), cerebrum (**O**), cerebellum (**P**), liver (**Q**), and spleen (**R**) at 48 h after the IV administration of HIRMAb-targeted THLs encapsulating a lacZ expression plasmid DNA under the influence of the eye-specific opsin promoter. (**E**–**M**) reproduced from [902], Copyright© 2003 licensed under Creative Commons Attribution License (CC-BY-NC-ND 4.0); (**I**,**N**,**O**,**Q**,**R**) reproduced from [1041], Copyright© 2003 licensed under Creative Commons Attribution License (CC-BY-NC-ND 3.0); (**P**) reproduced with permission from [1042], Copyright© 2007 Elsevier. (**S**) Intracranial U87 human glioma in the brain of a severe combined immunodeficient (scid) mouse removed at autopsy and stained immunohistochemically with an anti-EGFR antibody. Reproduced from [911], Copyright© 2002 licensed under Creative Commons Attribution License (CC-BY-NC-ND 4.0). (**T**,**U**) Confocal microscopy of scid mouse intra-cranial U87 human glioma at autopsy stained with antibodies against the mouse TfR (red) and the human EGFR (green); the mice in (**T**) were treated with saline and the mice in (**U**) were treated with doubly targeted HIRMAb/8D3-TfRMAb THLs encapsulating a plasmid DNA encoding a short hairpin RNA (shRNA) directed against nucleotides 2525–2557 of the human EGFR mRNA. (**T**,**U**) reproduced from [1043]. (**V**,**W**) Coronal sections of rat brain stained immunohistochemically with an antibody to tyrosine hydroxylase. Brains removed 3 days after a single IV injection of THLs encapsulating a plasmid DNA encoding rat tyrosine hydroxylase under the influence of a brain specific glial fibrillary acidic protein promoter and conjugated with either the OX26-TfRMAb (**V**) or a mouse IgG2a isotype control (**W**). The THLs were administered 7 days after the intra-cerebral injection of a neurotoxin, 6-hydroxydopamine, in the right median forebrain bundle. (**V**,**W**) from [1044]. (**X**,**Y**) Confocal microscopy of striatum ipsilateral to toxin lesion and double immune stained with antibodies against tyrosine hydroxylase (red) and neuronal neuN (green). Confocal micrograph in (**X**) corresponds to histochemistry in (**V**), and confocal micrograph in (**Y**) corresponds to histochemistry in (**W**). (**X**,**Y**) from [1044].

A THL is a 100–150 nm pegylated liposome that encapsulates a single plasmid DNA, and is functionalized for RMT delivery to brain by conjugation of a receptor-specific MAb on the surface PEG strands of the liposome (Figure 17A). 

THLs are formed by first encapsulating the plasmid DNA in the interior of pegylated liposomes, followed by conjugation of the targeting MAb to the surface of the liposome. THLs were initially produced from 96% 1-palmitoyl-2-oleoyl-glycero-3-phosphocholine (POPC), 3% 1,2-distearoyl-sn-glycero-3-phosphoethanolamine (DSPE)-polyethyleneglycol (PEG) 2000 Da (DSPE-PEG^2000^), 1% dimethyldioctadecylammonium (DDAB), and 0.15% DSPE-PEG^2000^-maleimide [1035]. The DDAB has a cationic charge, but the DSPE-PEG^2000^ has an anionic charge, so the THL has a net negative charge. In parallel, the targeting MAb is thiolated with a reagent such as 2-iminothiolane. The thiolated receptor-specific MAb is conjugated to the maleimide group of the PEG^2000^ on the liposome surface to form a stable thio-ether bond. Typically, each THL incorporates ~50 MAb molecules per liposome, and each THL encapsulates a single double stranded plasmid DNA in the interior of the liposome, such that the DNA is protected from nucleases. For dual receptor targeting, two MAb molecules of different receptor specificity may be conjugated to the THL surface so as to engage two different cell membrane receptors, as discussed below. Binding of the MAb domain of the THL to the cell membrane receptor triggers receptor-mediated transcytosis of the THL across the BBB, followed by receptor-mediated endocytosis into brain cells. As discussed in Section 8.1, certain receptors, such as the insulin or transferrin receptors, are expressed at both the BBB and the brain cell membrane (Figure 11B).

The PEG linked MAb extended from the surface of the THL is shown with electron microscopy (EM) in Figure 17B. In this study a 10 nm gold conjugated secondary antibody was mixed with the THL prior to EM. The size of the 10 nm gold is about the same size as the MAb, and the micrograph shows there are multiple MAb molecules conjugated to the surface of the THL [1039].

**THLs target plasmid DNA to the nuclear compartment of cells.** Lipofection of cells with plasmid DNA bound to cationic liposomes is an inefficient process, as the majority of the DNA that enters the cell is retained in the cytoplasm aggregated within pre-lysosomal vesicles [1045]. However, in the case of THLs, the majority of the endocytosed plasmid DNA is incorporated in the nuclear compartment. This was demonstrated with the confocal micrographs shown in Figure 17C,D. In this study, a plasmid DNA encoding an antisense RNA against the human epidermal growth factor receptor (EGFR) was labeled with fluorescein by nick translation and fluorescein-12–2′-deoxyuridine-5′-triphosphate [1040], and incorporated in THLs targeted with a MAb against the human insulin receptor, and designated the HIRMAb. The HIRMAb-THLs were incubated with human U87 glioma cells in culture and confocal microscopy was performed at 6 h (Figure 17C), and 24 h (Figure 17D). By 6 h, the majority of the DNA is in the cytosolic compartment, although transgene is visible in the nucleolus of the nucleus at 6 h. By 24 h, virtually all of the cellular transgene is localized to the nuclear compartment (Figure 17D). The insulin receptor is expressed on both the BBB and the brain cell membrane as discussed in Section 8.1.1. Following RMT of the THL across the BBB, and following insulin receptor-mediated endocytosis of the THL into brain cells, the liposome cargo must then be delivered to the nuclear compartment for gene expression. A MAb targeting the insulin receptor may be particularly suited to nuclear delivery of plasmid DNA, because the insulin receptor normally serves to deliver insulin to the nuclear compartment [1046,1047].

**THL brain delivery of reporter genes.** Plasmid DNAs encoding reporter genes such as the lacZ and luciferase genes have been encapsulated in HIRMAb-THLs for gene expression in the primate [902], in OX26 TfRMAb-THLs for gene expression in the rat [1035,1048], and in 8D3 TfRMAb-THLs for gene expression in the mouse [1049]. The global expression of the lacZ transgene in the Rhesus monkey brain following IV administration of HIRMAb-THLs was confirmed by X-Gal histochemistry. Histochemistry of un-injected primate brain showed no reaction [1050]. However, global expression of the lacZ transgene was observed in the primate brain at 48 h after the IV administration of the HIRMAb-targeted THLs encapsulating the lacZ expression plasmid DNA as shown in Figure 17E. The injection dose (ID) of lacZ plasmid DNA in this primate study, 12 μg/kg, is more than 2 log orders of magnitude lower than the ID, 5 mg/kg, of CAT plasmid DNA required for gene delivery to liver in the rat with the PLL-ASOR conjugate [1029], or luciferase gene delivery in the mouse with SPLPs [1032]. In a primate study with a luciferase reporter gene, the expression plasmid DNA was encapsulated in HIRMAb-THLs for IV administration in the Rhesus monkey and the injection dose of the luciferase plasmid DNA was also 12 μg/kg [902]. The brain luciferase gene expression at 48 h was 9–10 pg/mg protein [902], and qPCR analysis showed the luciferase plasmid DNA content in primate brain declined with a T_1/2_ of 1.3 ± 0.3 days, which correlated with the T_1/2_ of decline of brain luciferase enzyme activity of 2.1 ± 0.1 days [1051]. The qPCR quantitation of luciferase plasmid DNA content in brain at 2 days following the IV administration of the HIRMAb-THLs indicated that ~3 plasmid DNA molecules was delivered to every cell in brain [1051]. This finding on the global delivery to brain of a luciferase expression plasmid DNA correlates with the global expression of the lacZ transgene expression in the primate brain (Figure 17E). Light microscopy of regions of primate brain from the lacZ study showed transgene expression in the choroid plexus epithelium and the capillary endothelium of white matter (Figure 17F), in the cortical columns of the occipital cortex (Figure 17G), and in the molecular and granular layers and the intermediate Purkinje cells of the cerebellum (Figure 17H). Similar findings of global expression in brain of the lacZ gene were made in the mouse and rat following the IV administration of 8D3-THLs and OX26 TfRMAb-THLs, respectively [1048,1049]. Significant levels of lacZ gene expression were visible by histochemistry at 6 days following IV administration of THLs in the rat [1048]. The average number of HIRMAb, OX26 TfRMAb, or 8D3 TfRMAb antibodies conjugated per THL can be computed [891], and in the primate, rat, and mouse studies was 35–50 [902,1048,1049]. In both the rat and mouse study, no lacZ expression was detected in brain following the IV administration of THLs conjugated with the isotype control antibody, which is mouse IgG2a for OX26, and rat IgG for 8D3 [1048,1049]. The absence of gene expression with THLs targeted with the isotype control IgG shows that gene expression is determined by the receptor specificity of the MAb domain of the THL.

**Tissue-specific promoters and encapsulation of large sized plasmid DNA in THLs.** The lacZ gene encapsulated in the HIRMAb-THLs used for the primate study shown in Figure 17E–H was under the influence of the widely expressed SV40 promoter [902]. HIRMAb-THLs encapsulated with the SV40-lacZ produced transgene expression in the eye, the cerebrum and the cerebellum of the primate (Figure 17I–K), but also produced lacZ expression in peripheral organs such as liver (Figure 17L) and spleen (Figure 17M) in the primate [902,1041]. A lacZ expression plasmid under the influence of 2 kb of the 5′-flanking sequence (FS) of the bovine opsin gene, and designated pLacF [1052], was encapsulated in HIRMAb-THLs and injected intravenously in the Rhesus monkey. This resulted in lacZ expression in the eye (Figure 17N), but no lacZ gene expression in the cerebrum, cerebellum, liver or spleen (Figure 17O–R). IV administration of a lacZ plasmid encapsulated in HIRMAb-THLs produced global expression of the transgene in all layers of the retina (Figure 17I,N). There is greater expression of a lacZ transgene in the multiple layers of the primate retina following IV administration of HIRMAb-THLs [1041], as compared to lacZ expression in the layers of the retina of the mouse following IV administration of 8D3 TfRMAb-THLs [1053], and this is attributed to the widespread expression of the insulin receptor in human ocular tissues [1054].

An expression plasmid encoding the rat tyrosine hydroxylase (TH) cDNA under the influence of the SV40 promoter was encapsulated in OX26 TfRMAb-THLs and injected intravenously in the rat. This treatment produced off-target TH expression in the liver [1044]. The SV40 promoter was replaced with a GFAP promoter [1044], which was taken from the 2 kb of the 5′-FS of the human GFAP gene [1055]. IV administration of the GFAP-TH plasmid encapsulated in OX26 TfRMAb-THLs in the rat produced no TH transgene expression in the liver [1044], which is consistent with the lack of expression in liver of GFAP, a brain-specific gene.

The 1.5 kb 5′-FS of the human platelet-derived growth factor B (PDGFB) gene is a neuron specific promoter [1056]. An expression plasmid composed of the 4 kb cDNA encoding for the human Niemann Pick disease type 1 (NPC1) cholesterol transporter, and under the influence of the 1.5 kb human PDGF-B promoter, was engineered and this 8 kb plasmid was encapsulated in 8D3 TfRMAb-THLs for treatment of the NPC1 mouse [773], as described below.

Tissue-specific gene expression can be enabled by the administration of chromosomal-derived transgenes, as compared to cDNA-derived transgenes. The largest size plasmid DNA encapsulated in THLs is a 21 kb plasmid encoding the entire 18 kb rat TH gene, which is composed of 8.4 kb 5′-FS, 7.3 kb coding region with 13 exons and 12 introns, and 1.9 kb of 3′-FS [1057]. Following encapsulation of this 21 kb plasmid DNA in OX26 TfRMAb-THLs, the THLs were injected intravenously in the rat with experimental PD, and this treatment produced a >10-fold increase in striatal TH enzyme activity [1057]. TH gene therapy in PD only replaces the TH deficiency, but does nothing to abort the neurodegeneration of the nigral-striatal tract in PD. GDNF is a potent nigral-striatal neurotrophin [1058]. So as to restrict GDNF gene expression in the brain to the nigral-striatal tract, the cDNA encoding human prepro GDNF was placed under the influence of the 8.4 kb 5′-FS of the rat TH gene, and the size of the expression plasmid was 13 kb [1059]. This plasmid was encapsulated in OX26 TfRMAb THLs for treatment of the rat with experimental PD [1059], as described below. The expression in brain of the TH gene is restricted primarily to the nigral-striatal tract that degenerates in PD, so the TH promoter allows for region-specific GDNF gene expression in this region of brain.

In summary, tissue-specific gene expression following IV administration of plasmid DNA is possible with the combined use of tissue-specific gene promoters and THL plasmid DNA delivery technology. Some tissue-specific promoters are large, e.g., the 8.4 kb TH promoter used to restrict GDNF expression to the nigral-striatal tract of brain [1059]. In addition to the use of tissue-specific promoters, another goal in gene therapy is production of high levels of expression of the transgene. Gene expression can be enhanced by insertion of 5′-untranslated region (UTR) and 3′-UTR elements flanking the open reading frame of the transgene. The interaction of the 5′-UTR and 3′-UTR elements can have synergistic effects on gene expression. When a 171 nt 5′-UTR or a 200 nt 3′-UTR from the GLUT1 mRNA was inserted 5′ and 3′ in a luciferase expression cassette, transgene expression was increased 10-fold and 6-fold, respectively [1060]. However, when the luciferase expression cassette contained both GLUT1 mRNA 5′-UTR and 3′-UTR elements, transgene expression was increased 59-fold [1060]. The more the therapeutic gene expression cassette is modified with tissue-specific promoters or 5′-UTR or 3′-UTR elements, the greater the size of the expression cassette. The construction of advanced expression cassettes of larger size is not possible with AAV gene therapy, given the limited size of the expression cassette that can be inserted in the AAV backbone, e.g., <4.7 kb for incorporation in ssAAV, and <2.1 kb for incorporation in scAAV [998], as discussed in Section 10.1.4. In contrast, plasmid DNA up to 21 kb in size is expressed in vivo in brain following encapsulation and brain delivery with THLs [1057].

**THL brain delivery of therapeutic genes for brain cancer.** Human glial tumors over-express the EGFR gene, which plays an oncogenic role in these tumors [1061]. Treatment of glial tumors aim to suppress the expression of the tumor EGFR. To determine the effect of EGFR suppression, a human intracranial experimental glioma was produced by implantation of human U87 glioma cells in the caudate nucleus of scid mice [911]. The size of the tumor at autopsy is shown by the EGFR immunohistochemistry (Figure 17S). A plasmid DNA encoding an antisense RNA corresponding to nucleotides (nt) 2317–3006 of the human EGFR mRNA was inserted in a 11 kb plasmid under the influence of the SV40 promoter, and encapsulated in THLs [911]. These THLs were dual conjugated with both the HIRMAb, to target the HIR on the human glial cells, and the 8D3 TfRMAb, to target the mouse TfR on the tumor vascular endothelium, which originates from mouse brain. These antibodies are species specific, and the HIRMAb does not recognize the mouse insulin receptor on the tumor vascular endothelium, which originates from mouse brain, and the 8D3 TfRMAb does not recognize the tumor cell human TfR [911,1043]. Prior to treatment of the tumor-bearing mice, the dual Mab-targeted THLs were produced that encapsulated a luciferase expression plasmid DNA, which was injected intravenously in the mice with the U87 gliomas. The luciferase enzyme activity in the human tumor, targeted by the HIRMAb, was >10-fold higher than in normal mouse brain, targeted by the TfRMAb [911]. This greater degree of gene expression using the HIRMAb as compared to the TfRMAb was also observed with comparison of luciferase gene expression in the primate, targeted with the HIRMAb, as compared to luciferase gene expression in the rat, targeted with the TfRMAb [902]. The higher gene expression with the HIRMAb is attributed to the selective triage of the insulin receptor to the nucleus [1046,1047]. At 5 days following implantation of 500,000 U87 glioma cells in the caudate-putamen, mice were treated weekly by IV administration of (a) saline, (b) HIRMAb/TfRMAb-THLs encapsulating the luciferase expression plasmid, or (c) HIRMAb/TfRMAb-THLs encapsulating the EGFR antisense RNA expression plasmid. The time of 50% mortality, ED50, was 18 days for either the saline treated mice or the mice treated with THLs encapsulated with the luciferase plasmid DNA. However, the survival ED50 was increased 100% to 36 days for the mice treated with the THLs encapsulated with the plasmid DNA encoding the EGFR antisense RNA [911].

RNA interference (RNAi) therapeutics can be either short interfering RNA (siRNA) or short hairpin RNA (shRNA). The siRNA is administered as a short RNA duplex, and the shRNA is produced in target cells following the delivery of an shRNA expression plasmid DNA under the influence of a U6 promoter. Biotinylated siRNA is delivered to brain with a MAb Trojan horse coupled with avidin–biotin technology [1062]. For shRNA therapy, an expression plasmid was engineered that encoded a shRNA targeting nt 2529–2557 of the human EGFR under the influence of the U6 promoter [1043]. The intracranial tumor model used for the RNAi treatment was the U87/scid mouse model (Figure 17S) applied previously for testing the therapeutic effects of THL-mediated antisense gene therapy [911]. Treatment of U87 cells in culture with the HIRMAb-THLs encapsulating the shRNA expression plasmid produced a >90% suppression of EGF-mediated intracellular calcium flux [1043]. At 5 days following implantation of 500,000 U87 human glioma cells in the caudate-putamen, mice were treated weekly by IV administration of (a) saline or (b) 5 μg DNA per mouse of HIRMAb/TfRMAb-THLs encapsulating the anti-EGFR shRNA expression plasmid. The time of 50% mortality, ED50, was 17 days for the saline treated mice, and the ED50 was increased 88% to 32 days for the mice treated with THLs encapsulating the plasmid DNA encoding the shRNA [1043]. Confocal microscopy of the tumor at autopsy for the saline treated mouse and the THL/RNAi treated mice is shown in Figure 17T and 17U, respectively, where the immunoreactive human EGFR is shown in green and the immunoreactive mouse TfR is shown in red [1043]. This study shows RNAi treatment against the EGFR caused a knockdown in both the level of immunoreactive EGFR in the human tumor and the vascular density of the tumor, as shown by the level of immunoreactive mouse TfR at the vasculature. The capillary density, as assessed by immunohistochemistry of vascular TfR, was 35 ± 1 capillaries/mm^2^ in either the saline or the RNAi treated mice in the non-tumor mouse brain. However, the capillary density was reduced to 15 ± 2 and 3 ± 0 capillaries/mm^2^ in the center of the tumor in the saline and RNAi treated mice, respectively. The capillary density was 29 ± 4 and 9 ± 1 capillaries/mm^2^ in the periphery of the tumor in the saline and RNAi treated mice, respectively [1043]. The EGFR has a pro-angiogenic effect in brain tumors [1063]. Therefore, the knockdown of the tumor EGFR by the THL-mediated RNAi therapy caused a suppression of the vascular density of the tumor. Since the RNAi therapeutic is delivered to the brain tumor via the tumor vasculature, the knockdown of tumor EGFR has a self-limiting effect on the ultimate survival outcome. THL delivery of plasmid DNA encoding either antisense RNA [911] or shRNA [1043] directed against the EGFR has a therapeutic effect in brain cancer, but needs to be combined with other therapies that halt tumor progression before the sharp decline in tumor vasculature that occurs in the terminal stages of the tumor growth.

**THL brain delivery of therapeutic genes for Parkinson’s disease.** Parkinson’s disease (PD) is caused by degeneration of the nigral-striatal tract, resulting in reduced TH enzyme activity and dopamine production in the striatum. In an effort to develop a non-viral gene therapy of PD, an expression plasmid encoding the rat TH cDNA was engineered under the influence of either the widely expressed SV40 promoter [1039] or the brain-specific GFAP promoter [1044]. Experimental PD was induced by the intra-cerebral injection of 6-hydroxydopamine in the median forebrain bundle of one side of the brain in rats. At 1 week after toxin injection, rats were treated with a single dose of OX26 TfRMAb-THLs encapsulating 10 ug/rat of either the SV40-TH plasmid or the GFAP-TH plasmid. As a control, THLs were targeted with the mouse IgG2a isotype control antibody instead of the OX26 TfRMAb. PD rats were treated with 1 μg, 5 μg, or 10 μg of SV40-TH plasmid DNA encapsulated in the TfRMAb-THLs. Treatment at the 1 μg DNA/rat dose had no therapeutic effect; treatment at the 5 μg DNA/rat dose caused a partial restoration of striatal TH enzyme activity, while treatment at the 10 μg DNA/rat dose caused a complete normalization of the striatal TH enzyme activity. The therapeutic effect of the THLs was due singularly to the TfRMAb targeting, as THLs targeted with the mouse IgG2a isotype control antibody had no therapeutic effect [1039]. The striatal TH enzyme activity produced with the single THL treatment of 10 μg DNA/rat dose declined with a T_1/2_ of 6 days [1039]. The normalization of striatal TH enzyme activity was correlated with an improvement in motor function measured by the number of 360° rotations/min (RPM) induced by apomorphine treatment. In the study with the SV40-TH treatment, the apomorphine RPM was reduced from 20 ± 5 to 6 ± 2 [1039], and in the study with the GFAP-TH treatment, the apomorphine RPM was reduced from 22 ± 3 to 4 ± 3 [1044]. The increase in striatal TH enzyme activity caused by THL treatment correlated with the immunoreactive TH in the striatum. The treatment with the GFAP-TH plasmid encapsulated in OX26 TfRMAb-THLs normalized the immunoreactive TH in striatum of the PD rats (Figure 17V,X), whereas there was no therapeutic effect in PD rats treated with the GFAP-TH plasmid encapsulated in IgG_2a_-THLs (Figure 17W,Y).

The therapeutic effect of THL-mediated TH enzyme replacement in experimental PD lasts only 1 week [1039], owing to degradation of the plasmid DNA in brain [1051]. Therefore, treatment of brain with THL gene therapy requires chronic repeat administration. However, TH replacement gene therapy of PD does not address the underlying problem in PD, which is degeneration of the nigral-striatal tract. What is needed for PD is neurotrophin gene therapy that reverses the degeneration of the nigral-striatal region of brain, and GDNF is a potent neurotrophin for this region of brain. A human prepro GDNF expression plasmid DNA under the influence of the 8 kb rat TH promoter was engineered [1059], and encapsulated in OX26 TfRMAb-THLs and injected into PD rats at 1, 2, and 3 weeks after toxin administration at a dose of 10 μg DNA/rat per injection [1064]. The rats were then tested at 6 weeks after toxin administration, which was 3 weeks after the third and final dose of THLs, for apomorphine-induced rotation, for amphetamine-induced rotation, and for striatal TH enzyme activity. By 6 weeks after toxin administration, apomorphine-induced 360° rotation was increased to 25 ± 2 RPM in the saline treated PD rats, but was reduced 87% to 3 ± 1 RPM in the THL treated rats. Amphetamine-induced 360° rotation at 6 weeks after toxin injection was 11 ± 1 RPM in saline treated rats, and this was reduced 90% to 1.1 ± 0.2 RPM in the THL treated PD rats. The striatal TH enzyme activity was reduced 99% at 6 weeks after toxin injection in the saline treated rats, but was reduced only 23% at 6 weeks after toxin injection in the THL treated rats [1064]. These results indicate a more durable therapeutic effect in experimental PD is achieved with GDNF gene therapy as compared to TH gene therapy. Placement of the GDNF transgene under the influence of the TH promoter restricts GDNF expression only to sites where the TH gene is transcriptionally active [1059].

**THL brain delivery of a therapeutic gene for Niemann-Pick C1 disease.** Niemann-Pick C1 (NPC1) disease is an inherited disorder caused by mutations in the NPC1 gene, which encodes an intracellular membrane transporter of non-esterified cholesterol [1065]. The NPC1 cholesterol transporter is a large protein with an open reading frame of 3.9 kb. Therefore, the NPC1 cDNA can only be inserted in a ssAAV and with a small promoter and 3′-UTR, so that the expression cassette is <4.7 kb. It would be desirable to place the NPC1 gene under a neuron-specific promoter, such as the 1.5 kb human PDGF-B promoter [1056]. However, such a construct would be too large to insert into an AAV vector. An 8 kb expression plasmid was engineered, designated pPDGFB-NPC1, which placed the 3.9 kb NPC1 open reading frame under the influence of the 1.5 kb PDGFB promoter and a bovine growth hormone poly A sequence [706]. NPC1 mice replicate the neuropathology of human NPC1 [1066,1067]. Owing to the severe neuropathology, NPC1 mice die young at about 10 weeks with reduced body weight [706]. So as to encapsulate the pPDGFB-NPC1 plasmid in THLs active in the mouse, the recombinant 8D3 TfRMAb was expressed [706], based on the previously reported amino acid sequence for the heavy and light chains of this antibody [693]. THLs were produced from the recombinant 8D3 TfRMAb and encapsulated the pPDGFB-NPC1 plasmid DNA. Weekly intravenous THL treatment of NPC1 mice began at the age of 6–7 weeks. After euthanasia, the mass of the pPDGFB-NPC1 plasmid DNA was measured by qPCR in brain, liver, and spleen removed at 4 days following the last dose of THLs. The plasmid concentration in brain, liver, and spleen was 10.1 ± 3.1, 107 ± 9, and 40 ± 8 pg plasmid DNA per mg wet tissue [706]. High plasmid DNA content in spleen is attributed to the high expression of the TfR1 in spleen in the mouse [1068]. Based on the number of brain cells per mg wet brain, these qPCR studies indicate ~4 plasmid DNA molecules are delivered to each cell in brain [706]. The expression of the NPC1 mRNA in brain, spleen, and liver, relative to the mRNA of glyceraldehyde 3′-phosphate dehydrogenase (GAPDH), was measured by reverse transcriptase PCR [706]. The ΔCq parameter is the difference in qPCR Cq value for NPC1 and GAPDH for brain, spleen, or liver. The ΔΔCq is the difference in ΔCq for the vehicle treated mouse and the THL treated mouse. The change in NPC1 mRNA abundance in the organs of the THL treated mouse was computed from the base 2 antilog of the ΔΔCq [706]. The ΔΔCq values between THL and vehicle treatment groups showed the NPC1 mRNA, relative to the GAPDH mRNA, was increased 338-fold, 8192-fold, and 238-fold in brain, spleen, and liver, respectively [706], which indicates THL treatment resulted in a significant increase in NPC1 transcript in brain in the NPC1 null mice. THL treatment caused a reduction in cholesterol inclusion bodies in brain, and peripheral organs, but did not increase lifespan in these mice [706]. The lack of effect on lifespan was attributed to the delay in initiation of treatment until 6–7 weeks of age. By this time, the NPC1 mice already have developed pathologic changes in brain including autophagic lysosomal inclusion bodies, astrogliosis, microglia activation, and suppressed myelin production [1069,1070,1071,1072]. Future treatment of the NPC1 mouse should initiate THL gene therapy at birth.

**Safety pharmacology of chronic THL administration.** The safety of chronic administration of THLs was tested by treating rats with THLs encapsulated with a 7 kb TH expression plasmid under the influence of the SV40 promoter and conjugated with either the OX26 TfRMAb or its isotype mouse IgG_2a_ control [1073]. A dose of 20 μg/kg of THL encapsulated DNA was administered by weekly IV injections for 6 consecutive weeks. A third group of rats were treated with weekly saline and served as a control. Delivery of the TH expression plasmid to brain was confirmed by Southern blot. There was no change in body weight, 14 serum chemistries or histology of brain and peripheral organs (liver, spleen, heart, lung, kidney) following chronic THL administration. Immunohistochemistry of brain using primary antibodies against multiple markers of inflammation showed no inflammatory reaction in brain.

**THL manufacturing.** THLs described above were produced at scale of 1–5 mL using sonication, extrusion, and purification by gel filtration chromatography [1036]. These procedures are not scalable for commercial manufacturing. A scalable manufacturing process for pegylated liposomes, which can be adapted to THLs, uses an ethanol dilution method, which eliminates sonication, extrusion, and gel filtration [1074]. This scalable manufacturing approach, as recently reviewed [1037], adapts previously developed methods of liposome production by an ethanol injection method [1075], and use of a T-shaped device for rapid mixing of lipids and aqueous plasmid DNA [1076]. Ethanol dilution has two favorable effects on the encapsulation of plasmid DNA in pegylated liposomes. First, the ethanol dilution causes condensation of the plasmid DNA [1077], which is necessary to enable encapsulation of the plasmid DNA within the 100–150 nm vesicles. The gyration radius of 10 kb supercoiled plasmid DNA is 460 nm [1078], which greatly exceeds the radius of the small vesicles. Second, ethanol dilution induces the conversion of large multivesicular vesicles into small 100–150 nm vesicles [1079]. THL manufacturing at a 10 L level has been outlined using ethanol dilution and tangential flow filtration [525]. A relatively small volume of THL manufacturing of 15 L could provide an amount sufficient to treat an orphan disease with 50% market penetration for a year at a weekly IV infusion dose of 10 μg/kg of THL encapsulated plasmid DNA [525]. The primary problem in the manufacturing of THLs is product formulation to ensure a shelf life of 1–2 years, and this is possible with THL lyophilization. Recent work shows that THLs fully conjugated with MAb and encapsulating plasmid DNA can be lyophilized, stored, and re-hydrated providing the proper lyoprotectant is used [707]. Lyophilized and re-hydrated THLs passed manufacturing specifications when hydroxypropyl-gamma-cyclodextrin is used as the lyoprotectant [707]. The lyophilized, re-hydrated HIRMAb-THLs encapsulating a 5 kb plasmid DNA was injected intravenously in Rhesus monkeys at injection doses of 12 and 58 μg/kg of THL encapsulated plasmid DNA. The pharmacokinetics (PK) of plasma clearance of the plasmid DNA was determined by qPCR [707], and these PK parameters were comparable to those reported previously for HIRMAb plasma clearance in the primate [61]. IV administration of the lyophilized, hydrated THLs to Rhesus monkeys produced no adverse clinical events and no change in 25 serum chemistries [707].

**Variety of THL formulations.** Polymeric NPs (PNPs) were prepared with 45 kDa PLA and PLA-PEG-maleimide [1080], similar to the PLA NPs shown in Figure 15. The PNPs were mixed with an expression plasmid encoding TNF related apoptosis-inducing ligand (TRAIL), which is cytotoxic in tumors. The PLA-PEG, PLA-PEG-maleimide, and plasmid DNA were mixed and PNPs produced with an emulsion/solvent evaporation. The primary and secondary emulsions were produced by sonication [1080]. There is concern about the integrity of the DNA using such a procedure, since sonication can nick super-coiled plasmid DNA [1081,1082], which would reduce or eliminate gene expression. The Trojan horse used in these THLs was cationized bovine serum albumin (cBSA) and the thiolated cBSA was conjugated to the PEG extended maleimide [1080]. The Trojan horse PNPs were tested in vivo with a rat C6 intra-cranial glioma implanted in the striatum of BALB/c mice. Mice were treated at 7 days following implantation of the rat glioma cells, and were treated every 2–3 days for 2 weeks with a large dose, 100 μg/kg, of PNP encapsulated plasmid DNA. This treatment extended the median survival from 20 days to 42 days [1080]. In another study, OX26 TfRMAb THLs encapsulated a LacZ expression plasmid under the influence of either a cytomegalovirus or GFAP promoter, were injected intravenously in rats, and THL-mediated transgene expression in brain was confirmed [1083]. THLs were prepared by double conjugation with the OX26 TfRMAb, for delivery of liposomes across the rat BBB, and with chlorotoxin (CTX), a 4 kDa peptide from scorpion venom, which binds glioma cells [1084]. The plasmid DNA, pC27, encodes a 27 kDa carboxyl terminal peptide of human telomerase reverse transcriptase, which suppresses glioma growth. For production of THLs, the lipids, which included a PEG-maleimide, and plasmid DNA were initially sonicated prior to liposome formation by extrusion. The thiolated OX26 and thiolated CTX were then conjugated at the maleimide group on the THLs. Since sonication can damage super-coiled DNA [1081,1082], it is not advisable to sonicate after addition of DNA to the preparation. When DNA is exposed to sonication, the retention of the super-coiled conformation of the plasmid DNA should be confirmed by agarose gel electrophoresis, which typically elutes as multiple bands [707]. The therapeutic effects of the double conjugated THLs encapsulating the pC27 plasmid DNA were tested with a C6 intra-cranial glioma model in rats. The THLs conjugated only with the OX26 TfRMAb extended median survival of the tumor-bearing rats from 13 days to 29 days, and THLs double conjugated with the OX26 TfRMAb and CTX extended median survival of the rats to 46 days [1084]. In another approach, THLs were conjugated with an antibody against the IGF2 receptor (IGF2R), and encapsulated a plasmid DNA encoding p11, a 10–12 kDa protein implicated in depression [1085]. The results of these studies are difficult to interpret, since no information on the IGF2R MAb is provided. There are two types of IGF2R, and only one of these is expressed at the BBB. The IGF1R, which has high affinity for both IGF1 and IGF2, is expressed at the BBB, as discussed in Section 8.1.3. IGF2 also binds the 300 kDa cation independent M6PR, but this receptor is not expressed at the BBB, as discussed in Section 8.1.3, and recently reviewed [709]. Glutathione (GSH) has been conjugated to pegylated liposomes to trigger transport across the BBB by a putative GSH transporter at the brain endothelium (672). A pharmacokinetic analysis of brain transport of the GSH-pegylated liposome concluded the data did not support a model of transcytosis through the brain endothelium [1086]. This finding is expected for GSH conjugated THLs, since there is no known receptor for GSH at the BBB, and GSH does not cross the BBB, as discussed in Section 8.2.1.

**Figure 18:**
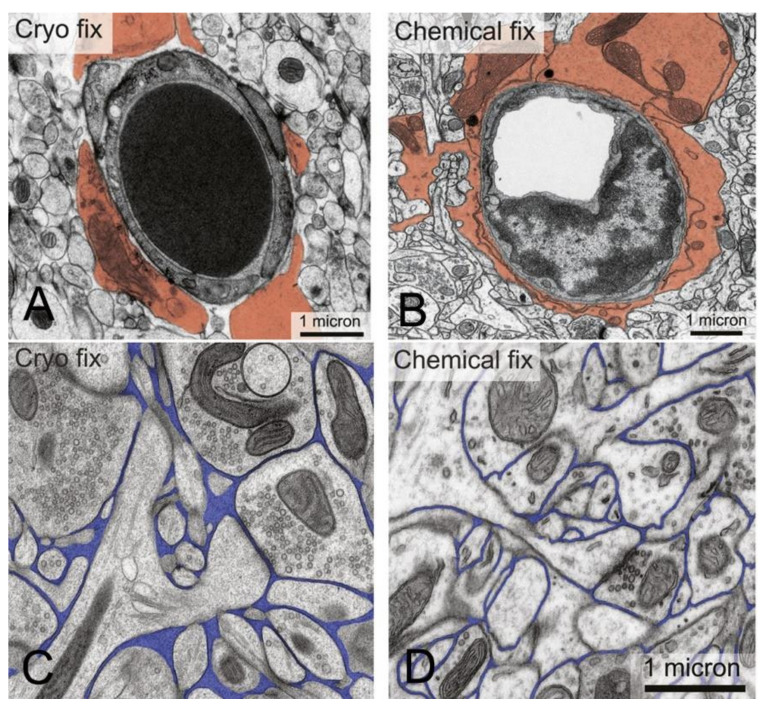
**Astrocyte endfeet and brain extracellular space in cryo-fixed and chemical-fixed brain.** (**A**,**B**) Brain capillary ultrastructure after cryo-fixation (**A**) and chemical fixation (**B**). The astrocyte endfeet are pseudo-colored in orange. An erythrocyte is present within the capillary lumen in (**A**). (**C**,**D**). Brain extracellular space after cryo-fixation (**C**) and chemical fixation (**D**). The extracellular space is pseudo-colored in blue. Reproduced from [22], Copyright© 2015 licensed under Creative Commons Attribution License (CC-BY).

**THL diffusion in brain.** Pegylated immunoliposomes were conjugated with the rat RI7-217 MAb against the mouse TfR [1087], as this ‘RI7’ TfRMAb undergoes RMT across the mouse BBB in vivo [663]. The transport of the RI7 TfRMAb-THLs across pial microvessels in mouse brain was investigated with two-photon microscopy through a cranial window [1087]. The findings led to the conclusion that while receptor-mediated endocytosis of the TfRMAb-THLs was an active process at the brain capillary, the exocytosis of the THL from the capillary endothelium into brain ECS was limited, as compared to a much higher rate of exocytosis into the brain ECS at post-capillary venules [1087]. The differential transport of THLs across the endothelium of the capillary vs. the post-capillary venule was said to be due to the lack of a peri-vascular space (PVS) at the capillary, and the presence of a PVS at the post-capillary venule [1087]. The hypothesis of an absent capillary PVS is derived from the assumption that the brain capillary is >90% invested by astrocyte foot processes. Electron microscopy of rat brain removed following aldehyde perfusion fixation shows an essentially complete ensheathment of the abluminal surface of the brain capillary by astrocyte foot processes [1088]. Owing to this complete investment of the capillary by astrocyte foot processes, it is assumed there is a fusion of the capillary basement membrane and the separate basement membrane lining the glial limitans or astrocyte foot processes, thus eliminating any PVS at the brain capillary [1089]. Conversely, at the post-capillary venule, it is said there is a separation of the capillary basement membrane and the glial limitans basement membrane, which creates a PVS at the post-capillary venule [1089]. The problem with this theory of complete investment of the capillary by the astrocyte endfeet, thus obliterating a PVS at the capillary, is that the theory is based on an artifact derived from electron microscopy of chemically fixed brain. A different picture of the brain PVS and astrocyte endfeet emerges with cryo-fixation of brain, as shown in Figure 18 and discussed below.

Electron microscopy of chemically fixed brain performed in the 1950s found a very small ECS in brain of only 3%, which did not comport with physiologic measurements of a brain ECS of ~25% of brain, as reviewed by Van Harreveld and colleagues in 1965 [1090]. A brain ECS volume of 24% was estimated by electron microscopy following cryo-fixation of brain [1090]. More recently, the investment of the capillary by astrocyte endfeet has been examined with electron microscopy following cryo-fixation of brain in comparison with chemical fixation of brain [22]. Chemical fixation causes astrocyte swelling and ECS shrinkage, which leads to an over-estimation of the capillary endothelial coverage by astrocyte foot processes [22]. Following cryo-fixation, only 63% of the capillary abluminal surface is invested by astrocyte foot process [22], whereas 94% coverage of the abluminal surface of the brain capillary by astrocyte endfeet is found with chemical fixation of brain [22]. The ECS volume of brain is estimated to be 15% following cryo-fixation, but only 2.5% following chemical fixation [22]. The brain capillary ultrastructure and brain ECS revealed by electron microscopy and cryofixation, which demonstrates the incomplete investment of the brain capillary by astrocyte endfeet, and the expanded brain ECS, is reproduced in Figure 18.

Following exocytosis at the abluminal membrane of the endothelium, the THL must diffuse 5–20 microns through the brain ECS to the neighboring neuron or glial cell body. The extent to which nanoparticles with a diameter of 100–150 nm can diffuse through the porous structure of the brain ECS was examined in living mouse brain with fluorescent microscopy through a cranial window following the intra-cerebral injection of pegylated polystyrene nanoparticles [1091]. Diffusion of pegylated nanoparticles was also measured with slices of fresh human brain removed at neurosurgery [1091]. Surface pegylation of the nanoparticle enhanced nanoparticle diffusion through the brain ECS [1091]. Functional pores in the ECS of human brain as large as 200 nm were observed, and 25% of human ECS pores had a diameter ≥100 nm [1091]. A definitive proof that THLs transcytose through the BBB, diffuse through brain ECS, and are taken up by brain cells is the histochemistry of brain showing global expression of a transgene in brain following IV administration of THLs encapsulating the plasmid DNA, as illustrated in Figure 17E–H for primate brain. Similar studies show global expression in brain of the transgene following THL delivery in the rat [1048] and mouse [1049].

## 11. Blood–Brain Barrier Transport Methodology

**Log BB.** The BBB is the limiting factor in the development of small molecule drugs for the CNS. In an effort to predict which small molecule drugs cross the BBB, the log BB parameter has been used for decades [1092], where BB is the brain/blood ratio of total drug in each compartment. A linear correlation between log BB and log P, where P = the drug partition coefficient in 1-octanol and water, was observed [1092]. The log BB is still used today [1093], despite the fact that the limitations of this parameter have been known for many years [1094]. The BB parameter, or brain/blood ratio, is a measure of the brain volume of distribution (VD), which is driven largely by brain tissue binding of drug. Thus, two drugs could have comparable rates of BBB transport, but widely disparate BB values, because one of the two drugs was avidly bound by brain tissue binding proteins, which increase the brain VD or BB ratio. The log BB parameter is being replaced by a new parameter, K_p,uu_ [1095], which is the concentration of *unbound* drug in brain, designated here as L_M_, divided by the *unbound* drug in plasma, designated here as L_F_. The problem with the K_p,uu_ parameter, i.e., the L_M_/L_F_ ratio, relates to how one experimentally measures the concentration of unbound drug in brain and the concentration of unbound drug in plasma, and whether the in vitro methods used to compute K_p,uu_ are reliable indices of the L_M_/L_F_ ratio in brain in vivo. An understanding of the factors controlling L_M_ and L_F_ in brain in vivo can be aided with a physiologic-based mathematical model of free drug in brain and plasma in vivo.

### 11.1. Physiologic Model of Free Drug in Brain and Plasma

**Figure 19:**
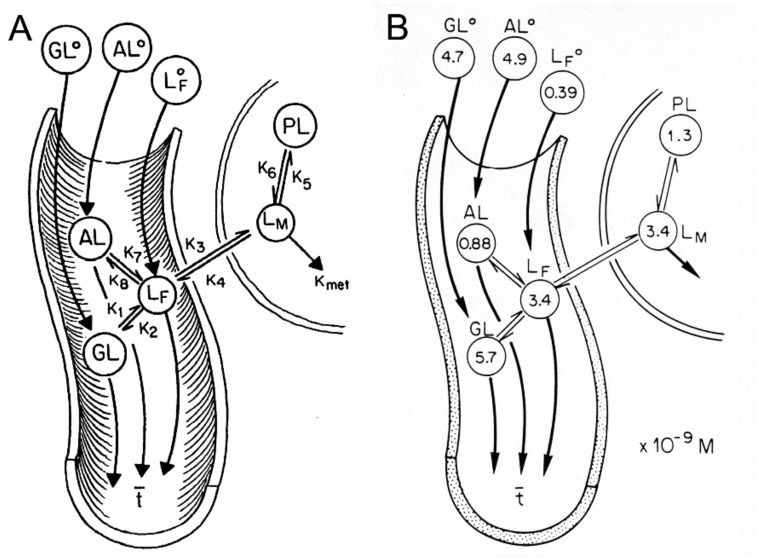
**Partly flow-partly compartmental model of drug influx and efflux at the BBB and drug binding to plasma proteins and brain tissue proteins.** (**A**) Drug in plasma may be bound to a plasma globulin, such as α_1_-acid glycoprotein (AAG), which is GL° and GL in the arterial and capillary compartments, respectively, or bound to albumin, which is AL° and AL, or may be free, which is L_F_° or L_F_, in the arterial and capillary compartments, respectively. The free drug in brain is L_M_, and the tissue bound drug in brain is PL. The rate constant of drug metabolism is K_met_. The rate constants of drug dissociation with AAG, albumin, and the tissue binding protein are K_1_, K_7_, and K_6_, respectively. The products of the rate constants of drug association and the concentration of the respective protein are given by K_2_, K_8_, and K_5_, respectively, for AAG, albumin, and the tissue binding protein. The rate constants of drug influx and efflux across the BBB are K_3_ and K_4_, respectively. The brain capillary transit time is denoted as $\bar{t}$. (**B**) Model predictions for testosterone concentrations in plasma and brain pools shown in (**A**). Model simulation is based on plasma sex hormone binding globulin and albumin concentrations of 28 nM and 640 μM, respectively. Reproduced with permission from [1096], Copyright© 1985 American Physiological Society.

A physiologic partly flow/partly compartmental mathematical model of brain transport of circulating drugs or hormones was developed to understand the relationship between the free drug in brain in vivo and the free drug in the brain capillary plasma compartment in vivo [1096], and this model is shown in Figure 19A.

The mathematical model of Figure 19A was solved analytically, and yielded the relationship for the ratio of free drug in brain (L_M_) and free drug in plasma (L_F_) given in Equation (2),

(2) $$\frac{L_{M}}{L_{F}}=(K_{3}\cdot \mathrm{Vp})/\left[(K_{4}+K_{\mathrm{met}}\right)\mathrm{VT}]$$

where Vp is the plasma volume of brain, 10 μL/g, VT is tissue volume of brain, 700 μL/g, K_3_ is the rate constant of drug influx from blood to brain across the BBB, K_4_ is the rate constant of drug efflux from brain to blood across the BBB, and K_met_ is the rate constant of drug metabolism in brain. Drug metabolism in brain may also take place within the endothelial compartment, owing to an enzymatic BBB, such as in the case for adenosine [391]. Since K_3_·Vp is equivalent to the permeability–surface area (PS) product of influx, PS^influx^, from plasma to brain across the BBB, and K_4_·VT is equivalent to the PS product of efflux, PS^efflux^, from brain to plasma across the BBB, then in the absence of significant drug metabolism in brain, where K_met_ = 0, then Equation (2) reduces to,

(3) $$K_{p,\mathrm{uu}}=\frac{L_{M}}{L_{F}}=\frac{{\mathrm{PS}}^{\mathrm{influx}}}{{\mathrm{PS}}^{\mathrm{efflux}}}\;\mathrm{or},{\;L}_{M}=\left[\frac{{\mathrm{PS}}^{\mathrm{influx}}}{{\mathrm{PS}}^{\mathrm{efflux}}}\right]\cdot L_{F}$$

The approximation of K_p,uu_ by the PS^influx^/PS^efflux^ ratio, given in Equation (3), has been recently proposed by Huttenen and colleagues [1097]. A PS^influx^/PS^efflux^ ratio < 1 is indicative of active efflux transport at the BBB. A PS^influx^/PS^efflux^ ratio = 1 is indicative of symmetric BBB transport, e.g., for a lipid-soluble drug that traverses the BBB via free diffusion.

The complexity of the factors controlling L_M_ and L_F_ in brain in vivo arises from the fact that many CNS drugs are bound by proteins in both plasma and in brain [1098]. Drug binding plasma proteins include albumin and globulins. The major drug binding globulin is α_1_-acid glycoprotein (AAG) also called orosomucoid. The physiologic model in Figure 19A accounts for the kinetics of drug binding to albumin and globulin plasma proteins, drug binding to brain tissue proteins, drug influx and efflux across the BBB, and drug metabolism in brain [1096].

The model in Figure 19A treats the brain interstitial and intracellular spaces as a single extravascular pool, owing to a much greater brain cell membrane surface area as compared to the surface area of the BBB, which is 120 cm^2^/g [11]. There are approximately 200 billion neuronal and non-neuronal cells in the 1000 g human brain [1099]. Modeling each cell as a 10-micron cuboidal structure yields a total brain cellular surface area of 1200 cm^2^/g, which is 10-fold greater than the surface area of the BBB.

The model in Figure 19A was evaluated for testosterone transport from blood to brain, and the results of the model simulation for testosterone are shown in Figure 19B, which gives the testosterone concentrations in the various pools of plasma and brain, as reported previously [1096]. As discussed in the next section, there is enhanced dissociation of testosterone from albumin within the brain capillary compartment in vivo [1100]. This enhanced dissociation produces a 9-fold elevation in the concentration of free (bioavailable) testosterone in the brain capillary in vivo (L_F_), 3.4 nM, relative to the concentration of free (dialyzable) drug in vitro, 0.39 nM, which is represented by the drug concentration in the arterial compartment (L_F_°) in Figure 19. The concentration of free drug in brain, L_M_, is identical to the bioavailable drug in the brain capillary compartment, L_F_ (Figure 19B). This identity between L_M_ and L_F_ is predicted by Equation (3) when drug transport across the BBB is symmetrical, i.e., PS^influx^ = PS^efflux^, and the drug is not metabolized in brain [1096].

The sections below review the methods available for determination of the 4 principal factors controlling in vivo K_p,uu_, which are the free (bioavailable) drug in plasma (L_F_), the free drug in brain (L_M_), PS^influx^, and PS^efflux^, with the assumption that drug metabolism in brain is nil, i.e., K_met_ = 0.

### 11.2. Free Drug in Plasma In Vivo and Role of Plasma Protein Binding

The major drug binding plasma proteins are albumin and α_1_-acid glycoprotein (AAG), also called orosomucoid. AAG is a 42 kDa heavily glycosylated plasma protein and the 3D structure of human AAG has been determined [1101]. AAG binds many drugs, and together with albumin, plays a major role in plasma protein binding of drugs [1101]. AAG is an acute-phase reactant, and the plasma AAG concentration can vary over 7-fold depending on the clinical condition [1102]. The concentration of AAG in plasma, about 0.1 g/dl, is 50-fold lower than the plasma albumin concentration [1102]. However, the affinity of AAG for many drugs can be 100-fold higher than the affinity of the drug for albumin binding. Therefore, many CNS drugs are bound by both albumin and by AAG in plasma.

The number of drugs that are highly bound by plasma proteins, e.g., >95% bound, is increasing. Before 2003, only about 30% of FDA-approved small molecules were classified as highly bound, but this number has increased to 45% of all drugs by 2019 [1103]. Therefore, it is important to develop a model of how plasma protein binding impacts the brain uptake of small molecule CNS drugs. The free drug hypothesis posits that the fraction of drug in plasma that is bioavailable for transport across the BBB, i.e., pool L_F_ in Figure 19 and Equation (3), is the same fraction that is free in plasma in vitro, as determined by a variety of methods, one of which is equilibrium dialysis [1104]. The free drug hypothesis is equivalent to the assertion that the dissociation constant, KD, governing the binding of drug to the plasma protein that is measured in vitro, or KD^in vitro^, is identical to the KD of drug binding to the plasma protein at the brain endothelial interface in vivo, or KD^in vivo^. If the guiding principle is, “to measure is to know” [1105], then this assumption of the free hormone hypothesis should be subjected to direct empiric testing in vivo. That is, the KD of the drug–plasma protein binding should be measured in vivo. If the drug–plasma protein KD^in vivo^ is >> than the KD^in vitro^, then enhanced ligand dissociation from the plasma protein occurs in vivo within the brain capillary compartment, relative to the dissociation rates observed in vitro. Conversely, if the KD^in vivo^ = KD^in vitro^, then the precepts of the free drug hypothesis are upheld with in vivo testing of the hypothesis.

**Figure 20:**
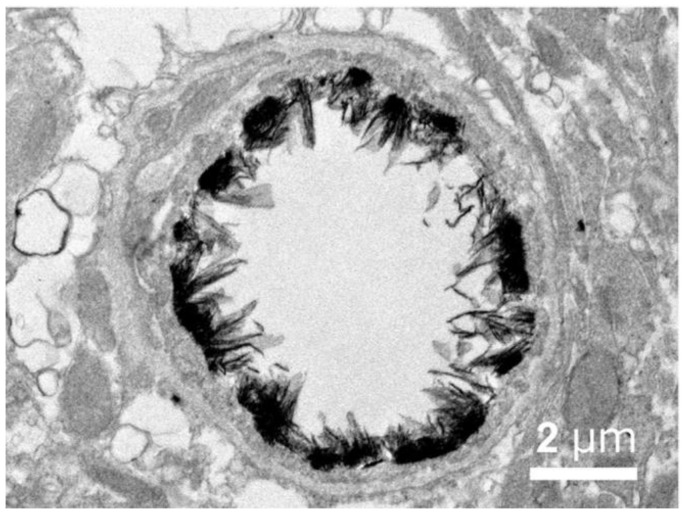
**Glycocalyx at the blood–brain barrier.** Brain capillary endothelial glycocalyx is visualized with lanthanum nitrate staining in the mouse. Reproduced from [20], Copyright© 2018 licensed under Creative Commons Attribution License (CC-BY).

Enhanced drug dissociation from plasma proteins such as albumin or AAG would be caused by conformational changes about the drug binding site on the plasma protein that takes place in vivo at the brain endothelial surface. Surface-mediated conformational changes have been documented for both albumin and AAG [1106,1107]. As plasma courses through the cerebral microcirculation, plasma proteins such as albumin or AAG transiently and reversibly bind the endothelial luminal glycocalyx [1108]. The brain endothelial glycocalyx is shown in Figure 20.

The glycocalyx covers 40% of the luminal surface of the brain capillary endothelium [20]. The thickness of the glycocalyx of the brain endothelial luminal membrane is up to 400 nm based on two-photon microscopy [19], and this finding on the thickness of the brain endothelial glycocalyx is confirmed by the electron micrograph in Figure 20. Therefore, the thickness of the endothelial glycocalyx is actually greater than the thickness of the brain capillary endothelium, 300 nm [17], as illustrated in Figure 20. Albumin binds reversibly to the glycocalyx surface of both the endothelium [1109] and the hepatocyte [1110]. The glycocalyx is composed of glycosaminoglycans (GAGs), which bind plasma proteins, including albumin and AAG [1108].

Albumin conformational changes have been observed following albumin binding either to GAGs [1107] or to the liver cell surface [1110]. The binding of AAG to GAGs [1111], or to biomembranes [1112], triggers conformational changes within the AAG protein that results in enhanced dissociation of drugs bound to AAG [1112]. If enhanced dissociation does occur at the brain endothelial interface, then the plasma protein-bound drug is operationally available for transport into brain, although there is no egress of the plasma protein, per se, from the plasma compartment of brain.

The KD^in vivo^ of drug binding to AAG, human serum albumin, or bovine serum albumin within the brain capillary has been measured using the Brain Uptake Index (BUI) carotid artery injection technique [1100], described below in Section 11.4.1. The KD^in vivo^ in brain, and the KD^in vitro^, as measured by equilibrium dialysis, is shown in Table 5 for multiple drugs and hormones.

The KD^in vivo^ was measured with the Kety–Renkin–Crone equation of capillary physiology, as described in Equation (4).

(4) $$E=1-e^{-f\cdot \frac{\mathrm{PS}}{F}},\;\mathrm{where}\;f=\left(\frac{{\mathrm{KD}}^{\mathrm{in}\;\mathrm{vivo}}}{n}\right)/\left(A+\frac{{\mathrm{KD}}^{\mathrm{in}\;\mathrm{vivo}}}{n}\right)$$

The extraction (E) of unidirectional influx of drug across the BBB following the carotid injection of the radiolabeled drug in the presence of different concentrations of the plasma protein is fit to Equation (4), where F = cerebral blood flow, f = the fraction of bioavailable drug in vivo in the brain capillary, A = the plasma protein concentration in the carotid artery injection solution, and n = the number of binding sites on the plasma protein [1100]. Curve fits are performed with non-linear regression analysis to estimate two parameters: the PS/F ratio and the KD^in vivo^/n. The ‘A’ parameter is technically the concentration of unbound plasma protein (A_F_). However, the concentration of total albumin or total AAG in the carotid artery injection solution is >10-fold greater than the drug concentration. Therefore, the unoccupied plasma protein concentration is approximated by the total plasma protein concentration in the carotid arterial injection solution. The extraction € of the drug by brain is plotted on the *y*-axis, vs. the protein concentration (A) on the *x*-axis, and non-linear regression analysis allows for computation of the PS/F ratio and the KD^in vivo^/n, as described by Equation (4) [1100]. In parallel, the KD^in vitro^ is determined by equilibrium dialysis.

The KD^in vivo^ at the brain capillary, as measured with the BUI technique, and the KD^in vitro^, as measured by equilibrium dialysis, have been determined in several studies on the binding of CNS drugs to AAG and to albumin, and these are summarized in Table 5. The experimental findings show that several albumin-bound drugs do not undergo enhanced dissociation at the brain capillary in vivo, and that the KD^in vivo^ is equal to the KD^in vitro^, e.g., propranolol, bupivacaine, and domitroban [1098]. However, drugs such as piroxicam, diazepam, devazepide, isradipine, darodipine, the amino acid, L-tryptophan, L-triiodothyronine, and a steroid hormone such as testosterone, do undergo enhanced rates of ligand dissociation from albumin in vivo within the brain capillary, which is indicated by the finding of KD^in vivo^ >> KD^in vitro^ (Table 5). With respect to drugs that bind AAG, propranolol, bupivacaine, imipramine, isradipine, and darodipine undergo enhanced dissociation within the brain capillary in vivo, whereas the binding of piroxicam to AAG in vivo within the brain capillary conforms to the binding of this drug to AAG in vitro [1098]. The measurement of the KD^in vivo^ with the BUI method can confirm the predictions of the free drug hypothesis, i.e., KD^in vivo^ = KD^in vitro^. However, in many instances, the free drug hypothesis is not confirmed in vivo, and the KD^in vivo^ is >> KD^in vitro^.

The finding of KD^in vivo^ >> KD^in vitro^ is evidence for enhanced dissociation of ligand from the plasma protein binding site in vivo within the brain capillary, which nullifies the key assumption of the free drug hypothesis. The only way to reconcile the in vivo brain drug uptake results with a hypothesis that asserts the KD^in vivo^ = KD^in vitro^ is a dissociation-limited model [1098]. The dissociation-limited model must posit that *both* drug re-association with the plasma protein in vivo *and* drug dissociation from the plasma protein in vivo are very slow compared to membrane permeation. Using the parameters in Figure 19A, the dissociation-limited model assumes both K_7_ << K_3_ and K_8_A_F_ << K_3_. Given the parameters of drug binding to the plasma protein, membrane permeation (K_3_) would have to be more than 2–3 log orders greater than the transit time of plasma through the brain capillary [1098], which would be equivalent to a BBB PS product that is 2–3 log orders higher than the rate of cerebral blood flow (F). The PS/F ratios for the drugs studied in the reports cited in Table 5 ranged from 0.14–1.35 with a mean of 0.88. These PS/F ratios are far too low to allow for a dissociation-limited mechanism of plasma protein-bound drugs across the BBB in vivo.

The discordance between drug binding to a plasma protein in vitro vs. drug binding in vivo within the capillary extends to organs other than brain. As an example, warfarin is bound by albumin, and albumin-bound warfarin, similar to some other albumin-bound drugs (Table 5), does not undergo enhanced dissociation at the BBB in vivo [1120]. However, warfarin undergoes enhanced dissociation from albumin in the microcirculation of liver, where the KD^in vivo^ of albumin binding of warfarin is 403 μM as compared to the KD^in vitro^ of albumin binding of warfarin, 20 μM [1121]. Therefore, warfarin undergoes a 20-fold enhanced dissociation from albumin at the liver microcirculation [1121], but there is no enhanced dissociation at the warfarin binding site on albumin at the brain microcirculation [1120].

Albumin bound steroid hormones, e.g., testosterone, albumin bound thyroid hormones, e.g., L-triiodothyronine (T3), and albumin bound L-tryptophan are available for transport across the BBB [322,433,1122], as exemplified by the high KD^in vivo^ relative to the KD^in vitro^ (Table 5). Conversely, globulin-bound steroid and thyroid hormones are generally not available for transport into brain, but are available for transport into liver in vivo [322,1098,1122,1123]. The plasma protein, per se, does not undergo transport into brain or liver. The plasma protein-bound drug or hormone is said to undergo transport into the organ in vivo, because the fraction of drug or hormone that is free (bioavailable) in the organ capillary in vivo is much greater than the free fraction of drug or hormone measured in vitro, e.g., with equilibrium dialysis. The enhanced dissociation of drug or hormone from albumin and/or globulin binding sites in vivo within the brain capillary compartment increases the free drug in the brain capillary plasma compartment, L_F_, relative to the free drug measured in vitro, L_F_° (Figure 19B). The fact that the KD^in vivo^ is often many-fold higher than the KD^in vitro^, as shown in Table 5, means the use of in vitro measurements of free drug in plasma, e.g., with equilibrium dialysis, may not provide an accurate representation of the drug–plasma protein binding interactions that take place in vivo at the brain endothelial surface. Under these conditions, the free drug in plasma in vivo within the brain capillary compartment, L_F_, is much greater than the free drug in plasma in vitro in a test tube.

### 11.3. Measurement of Free Drug in Brain

#### 11.3.1. CSF as a Measure of Free Drug in Brain

CSF is frequently used as a surrogate measure of drug transfer across the BBB, particularly in humans. This is based on ideas from the first half of the 20th century that CSF represents brain interstitial fluid (ISF) [26,32]. However, early dialysis fiber experiments showed that atenolol did not appear in the intra-cerebral micro-dialysate following ICV injection [88], and subsequent reviews commented on the lack of suitability of use of CSF drug penetration as an index of BBB transport [1124,1125]. In addition to the lack of equilibration between CSF and ISF, the CSF and ISF compartments are separated from blood by different membrane systems. The ISF is separated from the blood by the BBB and the CSF is separated from the blood by the choroid plexus. These anatomically distinct barriers are also functionally distinct and transporters that exist at the BBB may not exist at the choroid plexus and vice versa. As reviewed in Section 6.3.2, P-glycoprotein (Pgp) is expressed at the BBB [62], but is not expressed at the choroid plexus [62,478,479]. Co-administration of a Pgp inhibitor, zosuquidar, with nelfinavir, a Pgp substrate, produced an increase in brain distribution into brain, but not into CSF [477]. While the use of CSF as a surrogate for BBB transport has declined over the years for small molecule CNS drugs, developers of biologic drugs for the CNS still use CSF as a surrogate measure of BBB penetration, as discussed in Section 8.3.4. The CSF concentration of a therapeutic antibody is 0.1–0.2% of the plasma concentration, and this is said to indicate a small, but significant passage of the antibody across the BBB [784,785]. What is overlooked is that all IgG in plasma penetrates into CSF via passage across a leaky choroid plexus, and that the ratio of any IgG in CSF/plasma is normally 0.1–0.2% [12], which exists in the absence of any IgG transport across the BBB.

#### 11.3.2. Free Drug in Brain with Cerebral Microdialysis

Brain ISF is a protein-free compartment, so drug concentration in brain ISF is considered a measure of the free drug concentration in brain. Brain ISF is experimentally accessible with the implantation of an intra-cerebral dialysis probe [1126]. The experimental limitations of cerebral microdialysis were recognized early [88], and they include the lack of correlation of drug recovery across the dialysis membrane in vivo vs. in vitro, role of infusate temperature, and changes in the local brain environment triggered by what is effectively a stab wound of brain. The neuropathologic changes that are induced by insertion of a dialysis probe into brain was shown by an early study, which detected BBB disruption to circulating albumin by immunohistochemistry of brain following probe insertion [1127]. The entry of albumin into brain triggers an astrogliosis and microglia reaction in brain following insertion of the fiber [1128]. Placement of the microdialysis fiber in brain induces BBB disruption to small molecules as well as to albumin [1129,1130]. Film autoradiography was used to follow the BBB disruption to sucrose following insertion of the dialysis fiber. BBB permeability to sucrose was increased in a biphasic manner, and was increased 19-fold immediately after fiber insertion, and then 17-fold at 2 days after fiber insertion. BBB disruption persisted for at least 28 days after fiber implantation [1129].

#### 11.3.3. Free Drug in Brain In Vitro with Brain Slices or Homogenates

An alternative to cerebral microdialysis was developed using brain slices or homogenates. Drug is mixed with either the brain slice or brain homogenate and the volume of distribution of drug is measured from the ratio of drug concentration in the slice or homogenate relative to the medium drug concentration [1095,1131]. Drugs may be avidly bound by brain proteins, and this sequestration by brain can be examined with either brain homogenate or brain slice preparations. However, many CNS drugs are lipophilic amines, which are sequestered within the acidic intracellular lysosomal compartment, which can have a pH of 4.5–5.5. This acidity will trap a drug with a high pKa, where pKa is the pH at which 50% of the drug is ionized. The brain slice method is superior to the homogenate method as intracellular organelles, as well as brain cell membrane transporters, are intact in the brain slice preparation [1131]. The data provided with the brain slice or brain homogenate method is very useful in understanding the mechanisms by which drugs are bound and sequestered in brain. The problem with this in vitro methodology is that the free drug that is measured with a brain slice or homogenate in vitro, which is dominated by brain binding/sequestration mechanisms, is said to represent the free drug in brain in vivo [1095,1131]. However, the concentration of free drug in brain in vivo, which is shown as L_M_ in Figure 19A, is fully independent of brain binding/sequestration [1096]. As shown in Equation (2), the concentration of unbound drug in brain, L_M_, is a function of the PS^influx^, PS^efflux^, K_met_, and the bioavailable drug in plasma (L_F_), and is independent of tissue binding [1096]. The continuous flow of bioavailable drug in plasma, L_F_, acts as a forcing function in vivo [1132], and this forcing function of the continuous flow of plasma is non-existent with in vitro preparations of brain. The tissue bound drug in brain in vivo (PL, Figure 19) contributes to the total brain drug concentration, and determines the brain VD or brain/blood ratio or the log BB. However, the free drug in brain in vivo is independent of tissue binding, and is controlled by bi-directional BBB transport (PS^influx^ and PS^efflux^), the plasma bioavailable drug (L_F_), and brain drug metabolism, K_met_, as shown by Equation (2).

### 11.4. In Vivo Measurement of PS^influx^

#### 11.4.1. Brain Uptake Index Method

The BBB permeability–surface area (PS) product of influx from blood to brain can be measured with the Brain Uptake Index (BUI) method of Oldendorf [46]. An ~0.2 mL buffered solution of a [^14^C]-test molecule, and a [^3^H]-water reference is rapidly injected into the common carotid artery of an anesthetized rat through a 27-gauge needle, followed by decapitation at 15 s. The BUI is the ratio of extraction of the unidirectional influx of the test molecule (E_test_), divided by the extraction of the water reference (E_ref_), and is computed from the ratio of ^14^C-DPM/^3^H-DPM in brain divided by the same ratio in the injection solution. Since the BUI is a ratio of ratios, no measurements of brain weight or volume of injection solution are required. Since the test solution is injected into the common carotid artery, most of the injection solution is dispersed to organs other than brain via the external carotid artery [46]. However, this does not impact the measurement of E_test_, because an identical fraction of the test and references molecules are distributed to brain. The E_test_ = (BUI)·(E_ref_), and the PS product can be computed from E_test_ using Equation (4), which is the Kety–Renkin–Crone equation, where f = 1 when there is no plasma protein binding. If the test molecule is [^3^H]-labeled, then a [^14^C]-butanol reference can be used [1133]. Alternatively, three isotopes (^3^H, ^14^C, ^125^I) can be injected followed by triple isotope liquid scintillation counting [1133]. BUIs may be performed in conscious rats by placement of a PE-10 catheter into the external carotid artery a day before the study [1133]. The co-injection of [^3^H]-water, [^14^C]-butanol, and [^125^I]-N-isopropyl-p-iodoamphetamine allows for computation of the E_ref_ for either the [^3^H]-water or [^14^C]-butanol reference, which is 0.55 ± 0.01 and 0.87 ± 0.01, respectively, in the conscious rat [1133]. The cerebral blood flow (F) in the conscious, ketamine-anesthetized, and pentobarbital anesthetized rat is 1.64 ± 0.11, 0.93 ± 0.03, and 0.81 ± 0.09 mL/min/g, respectively [1133]. With the E_ref_ value, the BUI is converted to E_test_, and, the PS^influx^ is computed from the E_test_ and F with the Kety–Renkin–Crone equation (4). The unidirectional clearance (CL) from blood to brain is defined as CL = E_test_·F, or CL = (BUI)·E_ref_·F. When cerebral blood flow (F) is greater than the PS^influx^, then the Kety–Renkin–Crone equation, given in Equation (4) where f = 1.0, is approximated by E = PS^influx^/F, and unidirectional CL≈PS^influx^ [490].

**Carrier-mediated transport.** The Michaelis–Menten kinetics of carrier-mediated transport across the BBB in vivo can be determined with the BUI method, and Km and Vmax values for representative substrates of CMT systems are listed in Table 2. The relationship between PS, Km, Vmax, and K_NS_, where K_NS_ is the constant of non-saturable transport (μL/min/g), is defined in Equation (5),

(5) $$\mathrm{PS}=\left[\mathrm{Vmax}/\left(\mathrm{Km}+S\right)\right]+K_{\mathrm{NS}}$$

where Vmax is the maximal transport velocity (nmol/min/g), and Km (nmol/mL) is the absolute Km, which is the concentration (μM) of substrate (S) that causes 50% inhibition of transport. The Vmax, Km, and K_NS_ are determined by non-linear regression analysis, where the substrate clearance (CL) is plotted on the *y*-axis and the substrate concentration in the injection solution (S) is plotted on the *x*-axis, and CL = (BUI)·(E_ref_)·(F).

In the case of a CMT system that transports multiple competing substrates, e.g., LAT1 or CAT1, then the Km is an apparent Km, or Km^app^, which is defined by Equation (6),

(6) $${\mathrm{Km}}^{\mathrm{app}}=\mathrm{Km}\cdot \left[1+\sum \left(\frac{S_{i}}{\mathrm{Ki}}\right)\right]$$

The Km^app^ is derived from the absolute Km of the substrate, which is determined in the absence of competing inhibitors, the absolute Km for each inhibitor (Ki), and the concentration of competing inhibitor, Si, as shown in Equation (6) [1134]. The affinity of the CMT system is defined by the relationship between the absolute Km and the substrate concentration (S) in plasma. If the plasma S approximates Km, then the CMT system is high affinity, and Km^app^ > Km, which indicates substrate competition effects take place in vivo. If the plasma S << Km, then the CMT system is low affinity, and Km^app^ = Km, which indicates there are no competition effects in vivo. The transport of LNAAs across the BBB via LAT1 is the classic high affinity system, as the plasma concentrations of LNAAs approximate the absolute Km values for the individual LNAAs [355]. The high affinity (low Km) of the BBB LAT1 system, and the approximation of LAT1 Km values by the concentrations of the individual LNAAs in plasma, is the physical basis of the selective vulnerability of the CNS to hyperaminoacidemias [356]. The hyperphenylalaninemia of phenylketonuria (PKU) saturates the BBB LAT1 system with phenylalanine, and this saturation inhibits the brain uptake of other LNAAs, which are needed in brain to sustain cerebral protein synthesis. Conversely, LNAA transport in peripheral tissues is mediated by low affinity transporters with high Km values, and peripheral tissues are not exposed to LNAA starvation in the case of a hyperaminoacidemia such as PKU. Any drug, e.g., L-DOPA or gabapentin, that crosses the BBB via transport on LAT1 is subject to competition effects for BBB transport by the LNAAs in plasma.

In summary, the BUI technique is a versatile methodology that allows for quantitation of the kinetics of substrate influx from blood to brain via a CMT system, as defined by Equation (5). The BUI method also allows for the determination of the KD of binding of drugs or hormones to plasma proteins in vivo within the brain microcirculation, as defined by Equation (4). This is possible because the injection solution traverses the brain microcirculation as a first pass bolus with only ~5% mixing with rat plasma [1134].

#### 11.4.2. Internal Carotid Artery Perfusion Method

The BUI method is less sensitive when the E^test^ < 3–5%. In this case, BBB PS^influx^ can be determined with an internal carotid artery perfusion (ICAP) method [1135]. A PE-10 or PE-50 catheter is inserted in the external carotid artery, and the common carotid and pterygopalatine arteries are closed by ligation. Buffered fluid is perfused at rates of 1.2–5 mL/min for up to 5 min. At the end of the perfusion, the brain is removed for determination of brain radioactivity. A brain volume of distribution of the test molecule (VD^test^) is computed from the ratio of (DPM/g)/(DPM/μL perfusate). The perfusate also contains a second radiolabeled plasma volume (pv) marker, such as sucrose, and the VD of the plasma volume, VD^pv^, is also determined [1135]. The PS^influx^ = (VD^test^ − VD^pv^)/T, where T = the length of the perfusion. The ICAP method is more labor-intensive than the BUI method.

#### 11.4.3. Capillary Depletion Method

The ICAP method was modified to allow for study of the kinetics of AMT or RMT of biologic large molecules [506]. In this approach, the perfusion rate was 1.0–1.2 mL/min and the perfusion time was extended up to 10 min. For perfusion times >2.5 min, the rat blood volume was maintained constant by withdrawal of blood from a femoral artery catheter at the same rate as the infusion [506]. In the case of AMT or RMT of large molecules, it is important to separate endocytosis at the endothelium from transcytosis through the endothelial barrier, and this was performed with the capillary depletion method [506]. At the end of the perfusion, the brain is homogenized in cold 13% 60 kDa dextran, followed by centrifugation at 4 °C for 15 min at 5400× *g* in a swinging bucket rotor, and the post-vascular supernatant is carefully separated from the vascular pellet. Radioactivity is measured in each of the three fractions: total homogenate, post-vascular supernatant, and vascular pellet. Measurement of the activity of vascular specific enzymes, γ-glutamyl transpeptidase (γGTP) and alkaline phosphatase, showed the post-vascular supernatant was 94–95% depleted of brain vasculature [506]. Therefore, test molecules that distributed to the post-vascular supernatant had undergone transcytosis through the BBB during the perfusion period. The capillary depletion method was validated by the perfusion of acetylated LDL, a molecule that is only endocytosed into the capillary endothelium, and not transcytosed, as discussed in Section 9.2.1. Acetylated LDL was recovered only in the vascular compartment and not in the post-vascular supernatant [506]. During the homogenization of brain, the acetylated LDL was retained in the vascular pellet owing to the high affinity binding of this ligand to the scavenger receptor, which has a binding KD = 3 nM [1136]. Owing to this high affinity binding of acetylated LDL to the scavenger receptor, and also to the performance of the capillary depletion method at 4 °C at all steps, the acetylated LDL stays retained in the vascular compartment despite the homogenization of brain. Since the description of the capillary depletion method in 1990 [506], the method has been described in >300 publications in PubMed. In several of these studies, the capillary depletion method has not been used as originated, because the method has been applied to ligands with low affinity binding to the putative receptor at the BBB. Such low affinity ligands have rapid dissociation rates, and will most likely dissociate from the brain vasculature and appear in the supernatant during the homogenization process. This ligand dissociation from the BBB receptor during the homogenization will produce an artifact of ligand distribution to the post-vascular supernatant, and this artifact will be ascribed to BBB transcytosis. The capillary depletion method was developed only for ligands that bind to the target receptor on the BBB with high affinity, such that there is no dissociation from the capillary receptor during the homogenization process.

#### 11.4.4. Intravenous Injection Methods

The PS^influx^ can be determined following the IV co-injection of the labeled test molecule and a radiolabeled plasma volume marker, such as albumin. The drug concentration in brain (nmol/g), divided by the drug concentration in plasma (nmol/uL), is the brain VD (μL/g) of the test molecule, VD^test^. The brain VD of the plasma volume marker, VD^pv^, also with units of μL/g, is measured in parallel. The terminal plasma concentration, Cp(T), (nmol/μL), and the plasma area under the curve concentration (pAUC) (nmol·min/uL) during the time period (min) between IV injection and removal of brain, are also measured, as shown by Equation (7). The PS^influx^ is computed as follows [1094],

(7) $${\mathrm{PS}}^{\mathrm{influx}}=\frac{\left[\left({\mathrm{VD}}^{\mathrm{test}}-{\mathrm{VD}}^{\mathrm{pv}}\right)\cdot \mathrm{Cp}\left(T\right)\right]}{\mathrm{pAUC}}.$$

There are several caveats associated with determination of the PS^influx^ by IV injection methods, and these include (a) limitation of the time period of the influx measurement so that there is minimal efflux from brain back to blood; (b) measurement of the brain plasma volume (VD^pv^); (c) elimination of artifacts of brain uptake caused by peripheral degradation of the radiolabeled test molecule, and (d) determination of the plasma AUC or pAUC.

**Figure 21:**
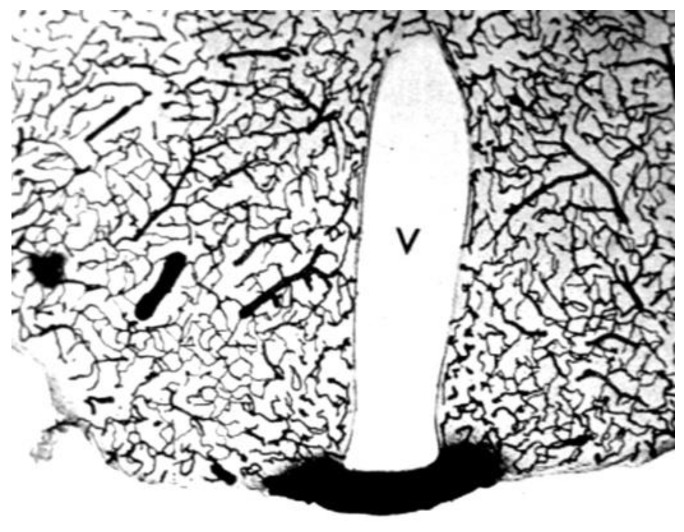
**Brain plasma volume.** Histochemistry of mouse brain following the IV administration of horseradish peroxidase (HRP), a 40 kDa enzyme. The enzyme is retained in the plasma volume of brain, except for the median eminence at the base of the third ventricle (V). Image provided as a gift from Dr. Milton W. Brightman. Reproduced from [709], Copyright© 2022 licensed under Creative Commons Attribution License (CC-BY).

**Brain plasma volume.** The brain plasma volume is visualized by the histochemistry of mouse brain removed after the IV administration of HRP, as shown in Figure 21.

It may seem paradoxical that a CNS drug, particularly a biologic drug, such as a therapeutic antibody, could be measurable in a homogenate of brain following IV administration, yet have not crossed the BBB. This paradox is visualized by the HRP histochemistry in Figure 21. The histochemistry shows the HRP in brain is confined to the brain plasma volume, except for the median eminence at the base of the third ventricle. The median eminence is a circumventricular organ (CVO), which are four tiny regions of brain that have no BBB [1137]. If the brain shown in Figure 21 was homogenized and HRP enzyme activity was measured, one might conclude that HRP crosses the BBB, when, in fact, the HRP does not cross the BBB, but is confined to the plasma volume of brain.

It is necessary to determine *both* the VD^test^ of the test molecule and the brain plasma volume (VD^pv^), which is explicitly included in Equation (7), as an experimental variable in the measurement of drug uptake by brain. The brain plasma volume is determined with either albumin or a non-specific IgG, neither of which cross the BBB. If the brain uptake of the test molecule is low, and VD^test^ approximates VD^pv^, then (VD^test^ − VD^pv^) = 0, and there is no BBB transport of the test molecule, as described in Equation (7). When there is no difference between VD^test^ and VD^pv^, then the BBB PS^influx^ = 0. In this setting, the test molecule is solely retained in brain within the plasma volume, as illustrated in Figure 21. The measurement of drug distribution in the brain plasma volume is particularly germane to the determination of the brain uptake of biologics following IV administration. As discussed in Section 8.3.4 for therapeutic antibodies for brain, antibodies have been injected intravenously and antibody was detected in homogenates of brain. If the injection dose of the antibody was increased, then a higher amount of antibody was detected in brain homogenate, because the antibody in plasma is increased at the higher injection dose. However, if no correction for brain plasma volume is made, then the higher antibody concentration in brain homogenate at the higher injection dose will be erroneously interpreted as evidence that the therapeutic antibody crossed the BBB [786].

**Figure 22:**
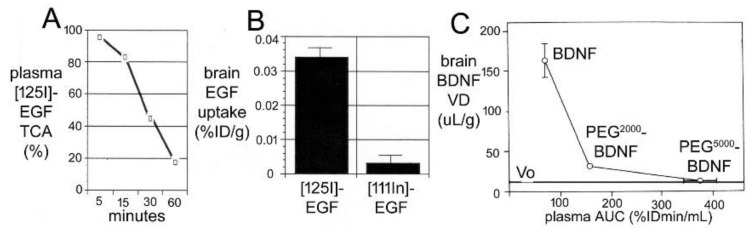
**Peptide metabolism and artifacts of brain uptake of radiolabeled peptide.** (**A**) Rapid a of trichloroacetic acid (TCA)-precipitable plasma radioactivity following the IV injection of [^125^I]-EGF in the rat. (**B**) Brain uptake of radioactivity is increased >10-fold following the IV injection of [^125^I]-EGF as compared to brain uptake after the IV injection of [^111^In]-EGF. (**A**,**B**) drawn from data reported in [805]. (**C**) The brain/plasma ratio of radioactivity is equal to the brain volume of distribution (VD), and this is plotted against the plasma AUC for 3 forms of radio-iodinated BDNF: [^125^I]-BDNF, [^125^I]-PEG^2000^-BDNF, and [^125^I]-PEG^5000^-BDNF. The progressive pegylation of BDNF with PEG^2000^ and then PEG^5000^ blocks the peripheral metabolism of BDNF, as reflected in the increasing plasma AUC. As the BDNF metabolism is progressively inhibited, the brain VD of BDNF decreases. The Vo, 13 ± 1 μL/g, shown by the horizontal bar is the brain plasma volume measured with [^14^C]-rat albumin. The brain VD of BDNF following pegylation with PEG^5000^ completely suppresses peripheral metabolism of the BDNF and the brain VD = Vo, which shows that BDNF does not cross the BBB. Reproduced with permission from [1139], Copyright© 1997 Springer-Nature.

**Artifacts caused by metabolism.** The second confounding variable in the measurement of the PS^influx^ using an IV injection technique is metabolism of the radiolabeled test molecule following uptake by peripheral tissues. The metabolic degradation of the [^125^I]-labeled test molecule leads to the release into the plasma of low molecular [^125^I]-metabolites that can cross the BBB. This brain uptake of metabolites produces radioactivity in brain that is not representative of the brain uptake of the test molecule. Instead, the brain uptake of the radioactivity is an artifact of metabolism of the test molecule. Such artifacts are exemplified in the case of the brain uptake of radioactivity following the IV injection of [^125^I]-EGF or [^125^I]-BDNF. The standard method of radio-iodination of a biologic is an oxidative reaction with 125-iodine and either chloramine T or Iodogen. This reaction places the ^125^I radiolabel on the aromatic ring of tyrosine residues on the protein or peptide. Following uptake and metabolism of the [^125^I]-peptide by peripheral tissues, there is a gradual increase in the plasma concentration of TCA-soluble radiolabeled metabolites, such as [^125^I]-tyrosine [1138]. The [^125^I]-tyrosine then enters the brain via CMT on BBB LAT1 to give an artifactual picture of brain uptake of the original radio-iodinated biologic, such as the [^125^I]-EGF (Figure 22A,B).

Conversely, when the EGF is conjugated with DTPA and chelated with 111-indium, the amount of radioactivity that enters the brain is decreased >10-fold (Figure 22B). The [^111^In]-EGF is taken up and metabolized by peripheral tissues to the same extent as the [^125^I]-EGF, but the ^111^In radioactivity is sequestered in the intracellular compartment of peripheral tissues and is not released to plasma [1138]. In another example of brain uptake artifact caused by peripheral metabolism of chloramine T/Iodogen labeled peptides, TCA-soluble metabolites appear in plasma soon after the IV injection of [^125^I]-BDNF radio-iodinated with chloramine T, and this produces a high brain uptake of radioactivity (Figure 22C). However, the peripheral degradation of [^125^I]-BDNF is progressively blocked by pegylation with either PEG^2000^ or PEG^5000^, as demonstrated by comparison of the plasma AUC for [^125^I]-BDNF, [^125^I]-PEG^2000^-BDNF, and [^125^I]-PEG^5000^-BDNF shown in Figure 22C. The pegylation of BDNF reduced the amount of TCA-soluble radiolabeled metabolites in plasma and reduced the brain uptake of radioactivity to the extent that the radioactivity was confined solely to the brain plasma volume following injection of [^125^I]-PEG^5000^-BDNF (Figure 22C). The data in Figure 22B shows that the preferred form of radio-labeling of a biologic is chelation of 111-indium. Alternatively, biologics can be radio-iodinated with the [^125^I]-Bolton–Hunter reagent, which conjugates the radiolabeled reagent to surface lysine residues in a non-oxidative reaction. Lysine conjugated with the [^125^I]-Bolton–Hunter reagent that is released to plasma does not cross the BBB [717].

**Plasma AUC.** The third caveat in the quantitation of PS^influx^ with IV injection methods is the need to determine the plasma AUC, pAUC, which is explicitly included in equation (7) as an experimental variable. The measurement of pAUC can be performed with standard pharmacokinetic methods when the experimental study period is long, e.g., >30 min between IV injection and harvesting of brain, which is typically the case for biologics. If brain uptake is measured during short experimental time periods between IV injection and organ harvesting, which is the case for small molecules, then the plasma AUC can be measured with an external organ method. In this approach, a femoral artery is catheterized and blood is withdrawn with a syringe pump during the experimental period [1140]. The plasma AUC (nmol·min/mL) is the drug concentration in the syringe (nmol/mL) multiplied by the experimental time period (minutes).

### 11.5. Measurement of PS^efflux^

The PS^efflux^ is the product of K_4·_VT, where K_4_ is the rate constant of drug efflux from brain to blood across the BBB, as illustrated in Figure 19 and Equation (2), and VT is the brain water space, 700 μL/g [1096]. The measurement of PS^efflux^ is more challenging than the estimation of PS^influx^, because a number of variables contribute to the rate of efflux of test molecules across the BBB from brain to blood. These variables include brain metabolism of the test molecule, brain binding of the test molecule, or active uptake of the test molecule by brain cells. Both the Brain Uptake Index (BUI) and Brain Efflux Index (BEI) methods can be used to measure the rate constant (K_4_, Figure 19) of test molecule efflux across the BBB.

#### 11.5.1. Brain Uptake Index Method

The Brain Uptake Index (BUI) method was first used to estimate solute efflux from brain to blood in 1975 [340,1141]. To use the BUI method to measure efflux, the time between carotid arterial injection and decapitation is prolonged from the usual 0.25 min to 1, 2, and 4 min. The brain is pulsed with solute within 5 s of the arterial injection, and efflux from brain to blood may then be monitored over the time period up to 4 min. Beyond 4 min, there is a loss of linearity of the efflux from brain owing to recirculation [1141]. Any metabolism of the test molecule during the 4 min will prevent reliable estimates of efflux, so studies are generally restricted to solutes not metabolized within 4 min of administration. Both the influx and the efflux of the non-metabolizable glucose analogue, 3-O-methyl D-glucose (3OMG), were measured with the BUI technique [340]. The PS^influx^ and PS^efflux^ were not significantly different, which indicated the BBB glucose carrier was a symmetrical transporter [340], as originally suggested by Crone [1142]. The kinetic analysis of 3OMG efflux from brain was based on the earlier theoretical analyses of solute efflux from skeletal muscle [1143].

#### 11.5.2. Brain Efflux Index Method

The Brain Efflux Index (BEI) method has advantages over the BUI method for the study of solute efflux from brain to blood. First, efflux of solute that has a low rate of influx from blood to brain can be measured with the BEI method. The study of efflux with the BUI method requires a significant influx of the test solute into brain from blood so that efflux from brain can be measured. In the BEI method, the radiolabeled test solute is injected directly into brain under stereotaxic guidance [450]. The second advantage of the BEI method is that the effects of cross-competition between substrates or drugs and the radiolabeled test solute can be measured, as reviewed in Section 6.3.1. Solute or drug efflux from brain is typically measured over time periods of 20–60 min with the BEI method. Under these conditions, it is important to confirm there is no metabolism of the test solute during the experimental period. If the test molecule was metabolized in brain, then efflux of the radiolabeled metabolite would produce an artifact, and lead to erroneous conclusions about solute efflux from brain. The classic example of artifacts of solute efflux caused by brain metabolism is the case of the Abeta amyloid peptide, as discussed in Section 8.1.5. In the original study [1144], [^125^I]-Aβ^1–40/42^ was injected into brain, and efflux of radioactivity from brain to blood was observed, and ascribed to LRP1-mediated efflux of the Aβ^1–40^ peptide from brain to blood. This model had important implications for the understanding of the formation of Aβ amyloid plaques in AD, and the extent to which receptor-mediated efflux of Aβ peptides from brain to blood had on this process. However, the efflux of radioactivity from brain was shown to be an artifact caused by rapid degradation of [^125^I]-Aβ^1–40/42^ in brain following intra-cerebral injection of the amyloid peptide [607]. The suppression of degradation of [^125^I]-Aβ^1–40^ in brain eliminates the efflux of radioactivity [607], which indicates the Aβ amyloid peptide of AD does not efflux from brain across the BBB.

In addition to metabolism, interpretation of BEI data is also confounded by brain tissue binding/sequestration of the ligand. The rate constant of efflux (K_eff_) of estrone, a highly lipid-soluble sex steroid that freely crosses the BBB [322], is only 0.069 min^−1^ as measured with the BEI method [457]. This K_eff_ for estrone is not a measure of BBB permeability on the brain side of the barrier, i.e., the K_4_ parameter in Figure 19A. The K_eff_ measured with the BEI method is much less than the K_4_ of estrone efflux from brain to blood across the BBB, owing to sequestration by brain tissue binding proteins of sex steroids such as estrone. A mathematical model, similar to that developed for analysis of efflux across the BBB and brain tissue binding in vivo, for either steroid hormones [458] or drugs [1145], must be developed to discern how both efflux across the BBB and brain tissue binding/sequestration influences the K_eff_ determined with the BEI method.

### 11.6. Measurement of Drug Sequestration in Brain In Vivo

**Figure 23:**
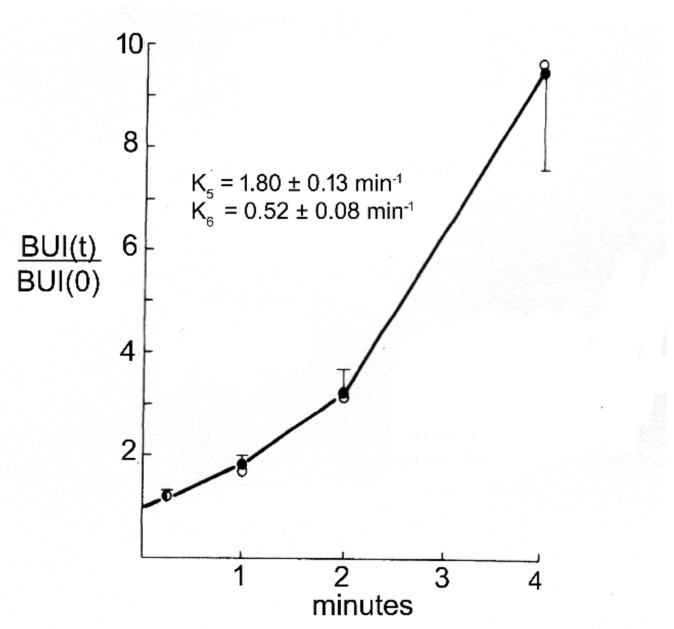
Propranolol binding to brain tissue proteins. The BUI at 1, 2, or 4 min after injection, BUI(t), relative to the BUI at T = 0, is plotted against the time after carotid artery injection. The data were fit to a compartmental model of efflux and tissue binding similar to that shown in Figure 19, which allowed for determination of the rate constant of drug association (K_5_) and the rate constant of drug dissociation (K_6_) from tissue binding proteins. The closed circles are the experimentally determined BUI values, and the open circles are the BUI values predicted from fitting these data to the model of drug efflux and binding in brain. Reproduced in part with permission from [1145], Copyright© 1984 American Physiological Society.

The ratio of propranolol concentration in brain (B), relative to plasma (P), in humans is high, e.g., the BP ratio is 17 [1145]. A BB or BP ratio greater than 1 is indicative of either active transport of drug into brain, or more likely sequestration of the drug in brain, e.g., by brain tissue binding. The BUI method was used to compute the rate constants of binding of drugs, such as propranolol or lidocaine [1145], to brain tissue in vivo, as shown in Figure 23 for propranolol.

Fitting BUI data to a mathematical model allowed for estimation of the rate constants of drug association (K_5_) and dissociation (K_6_) of binding to brain tissue in vivo, where these rate constants are defined in Figure 19A. The BUI of [^3^H]-propranolol, relative to [^14^C]-butanol, was measured for up to 4 min after common carotid artery injection (Figure 23). Thin layer chromatography of brain showed there was no metabolism of the [^3^H]-propranolol during the 4 min experimental period [1145]. The differential equations and analytic solutions of the mathematical model used to derive from BUI data the K_5_ and K_6_ parameters of propranolol binding in brain in vivo have been reported previously [458,1145]. The increase in the BUI with time after carotid arterial injection shown in Figure 23 is due to (a) binding/sequestration of [^3^H]-propranolol by brain, and (b) the rapid efflux from brain of the freely diffusible [^14^C]-butanol reference, which is not sequestered by brain. Similar to propranolol, sex steroid hormones are also avidly sequestered by brain [458]. Unlike the sex steroid hormones, the corticosteroid, corticosterone, is not sequestered in brain in vivo [458]. The selective sequestration of sex steroids, but not corticosteroids, by brain in vivo explains the high brain VD, i.e., high BB ratios, of the sex steroids relative to a much lower BB ratio for the corticosteroids [458]. Developmental regulation of brain sequestration of sex steroid hormones is observed, as no brain tissue binding is found in newborn rabbits [458]. The selectivity of the brain binding of sex steroid hormones, but not corticosteroids, and the developmental regulation of this process for steroid hormones, suggests that specific binding proteins are responsible for the sequestration in brain of hormones and drugs. However, mechanisms other than tissue binding may account for a high BB or BP ratio, particularly for lipophilic amine drugs such as propranolol or lidocaine, which may exist in protonated forms. The effect of plasma pH on BBB transport of propranolol or lidocaine in vivo was investigated with the BUI method [1145]. Influx of either drug across the BBB in vivo was inhibited 40–50% when the pH of the injection solution was lowered from 7.5 to 5.5. The lower transport of the protonated form of the drug was attributed to preferential transport of the unprotonated drug across the BBB in vivo. Similarly, CNS drugs with a high pKa may be protonated in the acidic compartment of the lysosome, which would contribute to the sequestration of the drug by brain, as demonstrated with the brain slice preparation [1131] discussed in Section 11.3.3.

### 11.7. In Vitro Models of BBB Transport

#### 11.7.1. Isolated Brain Microvessels

**Figure 24:**
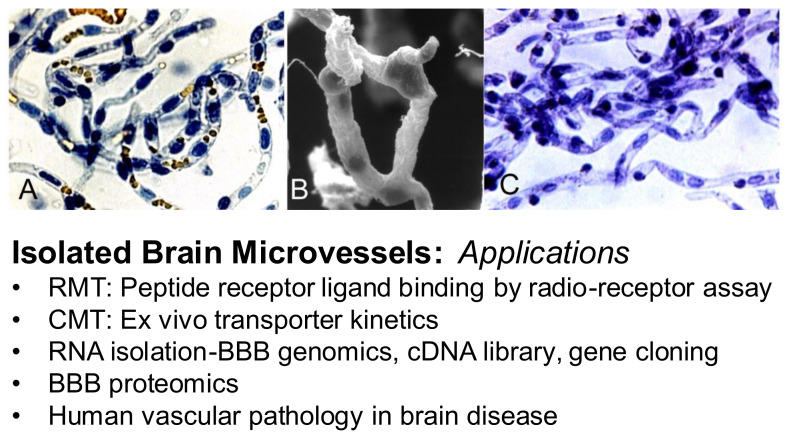
**Isolated brain microvessels.** (**A**) Trypan blue stain of freshly isolated bovine brain microvessels. (**B**) Scanning electron micrograph of bovine brain capillaries with attached nerve endings. (**C**) Trypan blue stain of microvessels isolated from human autopsy brain. Reproduced from [569], Copyright© 2020 licensed under Creative Commons Attribution License (CC-BY).

Brain microvessels were first isolated in 1969 from bovine and human brain [1146], and subsequently from rat brain [1147,1148]. The microvessels are isolated free of adjoining brain tissue as shown in Figure 24.

Isolated brain microvessels were originally proposed as models for the investigation of brain endothelial metabolism [1148,1149]. The original methods for isolation of brain microvessels used a mechanical homogenization technique. Subsequently, microvessels were isolated with an enzymatic homogenization method, and it was said that brain capillaries isolated with the enzymatic method excluded trypan blue, whereas capillaries isolated with the mechanical homogenization method failed to exclude trypan blue [1150]. Microvessels stained with trypan blue are shown in Figure 24A,C for bovine brain or human brain, respectively. The failure of cells to exclude trypan blue is an index of cellular metabolic dysfunction. The cellular ATP levels of brain microvessels isolated with either a mechanical or enzymatic homogenization procedure have ATP concentrations <10% of normal [1151]. The cause of the metabolic dysfunction, and severe loss of cellular ATP, in microvessels freshly isolated from brain has not been elucidated. Despite the metabolic impairment of isolated brain microvessels, these structures have proven over the last 50 years to be a versatile in vitro model for the study of the cellular and molecular biology of the BBB and neurovascular unit [569], and the major areas of study include (a) radio-receptor assays for characterization of BBB receptor-mediated transport (RMT) systems; (b) ex vivo kinetic studies of the uptake of nutrients and vitamins via BBB carrier-mediated transport (CMT) systems; (c) isolation of brain microvessel RNA, which allows for BBB genomics and an analysis of the brain microvascular transcriptome; (d) quantitative absolute targeted proteomics (QTAP) determinations of brain microvessel concentration of RMT and CMT systems; (e) vascular pathology in human brain disease, as outlined in Figure 24.

**Radio-receptor assays, isolated brain capillaries, and BBB RMT systems.** Several of the RMT systems at the BBB discussed in Section 8.1 were identified with radio-receptor assays and microvessels isolated from human autopsy brain, including the human BBB insulin receptor [561], the human BBB transferrin receptor [579], the human IGF receptor [590], and the human leptin receptor [595].

**Ex vivo kinetics of BBB CMT systems.** Ex vivo kinetics of transport of nutrients via BBB CMT systems, discussed in Section 6.2, have been determined with isolated brain capillaries. The isolated human brain capillary preparation was used in 1985 to describe the kinetics of BBB transport of [^3^H]-phenylalanine, and the selective inhibition of phenylalanine transport by other LNAAs [1152]. The use of isolated brain capillaries to characterize multiple BBB CMT and AET transporters has been recently reviewed [569], as have methods for experimental design of ex vivo transport with isolated brain capillaries [1153]. The isolation of plasma membranes from brain capillaries was first reported in 1980 [1154], and in 1992, these membrane vesicles were used to characterize luminal and abluminal amino acid transport at the BBB [1155].

**BBB genomics.** The field of BBB genomics was first described in 2001 using RNA purified from capillaries isolated from fresh rat brain [438] and in 2002 using RNA purified from capillaries isolated from fresh human brain removed at neurosurgery [442]. The microvessel-derived RNA was used to produce cDNA libraries, which facilitated the molecular cloning of multiple BBB transporters, including GLUT1 [1156], LAT1 [358], CNT2 [395], and BSAT1/Slco1c1 [440]. The BBB transcriptome has been characterized with multiple experimental approaches [1157,1158,1159,1160], as recently reviewed [569].

**BBB proteomics.** The combined used of the isolated brain capillary preparation and liquid chromatography-mass spectrometry (LC-MS) allowed for quantitative targeted absolute proteomics (QTAP), which was first described in 2011 for the human brain capillary [360]. The QTAP methodology subsequently was described for the hCMEC/D3 human cultured endothelium [394], the rat brain capillary [409], the monkey brain capillary [435,486], the choroid plexus [480], the arachnoid membrane [500], and isolated luminal and abluminal capillary membranes [468]. The QTAP programs allowed for quantitation of the multiple CMT and RMT systems at the BBB discussed in Section 6.2 and Section 8.1.

**Vascular pathology in human neural disease.** The brain microvasculature plays a primary role in the pathogenesis of AD, as all extracellular amyloid plaques arise from the peri-vascular surface [1161]. Cortical microvessels were first isolated from AD cortical brain in 1987, which allowed for the purification and AA sequencing of the microvascular Aβ amyloid peptide of AD [1162]. These studies confirmed earlier results on the sequence of the Aβ amyloid peptide isolated from meningeal vessels of AD brain [1163]. Microvessels have since been isolated from AD autopsy brain for a variety of experimental applications [1164,1165,1166,1167]. One of the earliest lesions in brain in multiple sclerosis (MS) is a peri-vascular cuffing of lymphocytes [1168]. Microvessels were first isolated from human MS brain in 1989, which showed selective expression of the class II histocompatibility DR antigen in microvascular pericytes in MS brain [1169], a finding subsequently confirmed [1170]. Capillaries can be isolated from frozen human autopsy brain stored in brain banks [1171]. The brain microvasculature plays a primary role in multiple neural diseases, apart from AD or MS. The molecular analysis of capillaries isolated from brain bank specimens of human neural disease represents a unique, yet currently under-developed, area of the neurosciences. Caution should be used for any study that generates RNA from capillaries isolated from brain bank specimens, as the time period between death, autopsy, and freezing of the brain specimen is generally not known, and degradation of capillary RNA may take place. For RNA work, it is preferable to isolate microvessels from fresh human brain. Methods for isolation of microvessels from fresh human brain have been recently reviewed [1172].

#### 11.7.2. In Vitro Models of BBB Transport in Cell Culture

**History of in vitro BBB model development.** The development of an in vitro model of the BBB that was suitable for high throughput screening of multiple compounds for BBB permeability has been long sought by the pharmaceutical industry, and the development of such in vitro BBB models have a history covering the last 40 years. In 1983, bovine brain microvessel endothelial cells were grown in tissue culture as “a model for the study of blood-brain barrier permeability” [1173]. However, in vivo/in vitro BBB permeability comparisons showed the in vitro BBB model was leaky and over-estimated BBB permeability for small molecules that crossed the BBB by free diffusion, and under-estimated BBB permeability for small molecules that crossed the BBB by CMT [1174]. No specific CMT transport of L-DOPA across the in vitro BBB could be measured [1174], which was due to the marked down-regulation of LAT1 gene expression when brain endothelial cells are grown in cell culture. Detection of the LAT1 transcript by Northern blot using 2 μg polyA + RNA purified from either isolated brain capillaries or cultured brain endothelial cells required development of the film autoradiogram for 2 h vs. 7 days, respectively [358,1175], which indicates LAT1 gene expression is down-regulated ~100-fold when brain endothelial cells are grown in culture. A similar level of down-regulation of GLUT1 gene expression was observed when brain endothelial cells were grown in cell culture [1156]. Early studies showed down-regulation of a BBB-specific enzyme, γGTP, in cell culture, and the partial up-regulation of γGTP expression by co-culture of endothelial cells with astrocytes [1176]. In 1990, the transwell model was developed, where primary cultures of bovine brain endothelial cells were grown on one side of a transwell, and primary cultures of newborn rat brain astrocytes were grown on the other side of Millicell-CM filter with a pore size of 0.4 microns [1177]. The transwell model lacks the shear stress caused by capillary blood flow. The shear stress on brain endothelial cells in vivo is 5–20 dyne/cm^2^ [1178]. This flow-related shear stress was produced with an in vitro BBB model by co-culture of bovine aortic endothelial cells and rat C6 glioma cells in a hollow fiber cartridge; fluid flow through the cartridge at 4 mL/min produced a shear stress of 4 dyne/cm^2^ [1179]. This dynamic in vitro BBB model was a precursor to a microfluidic ‘BBB on a chip’ models where a silicone chip was fabricated to allow for fluid flow through an outer endothelial chamber with astrocytes cultured in an inner chamber [1180]. The BBB-on-a-chip model was first described in 2005, although this model had no fluid flow component [1181]. It is not clear that in vitro BBB models require continuous flow of culture medium. The hypothesis that fluid flow induces BBB properties in brain endothelial cells is at odds with the lack of barrier properties in endothelia of non-brain organs, which are also exposed to flow induced shear stress [569]. In 2012, human-induced pluripotent stem cells (iPSC) were used to produce an in vitro BBB model [1182], and expression of tight junction proteins in the iPSC in vitro models was enhanced by the addition of 5 μM retinoic acid to the medium [1183]. Retinoic acid increases tight junctions via the Wnt signaling pathway [1184], which plays a special role in the differentiation of brain endothelium [1185]. The in vitro BBB model has undergone significant improvements over the last 40 years [1186]. However, the central issue is the extent to which even modern in vitro BBB models replicate the properties of the BBB in vivo. The in vitro/in vivo comparisons address the trans-endothelial electrical resistance (TEER), the permeability coefficient (Pe, cm/s) of sucrose, and the tissue-specific gene expression at the BBB in vitro and in vivo.

**Trans-endothelial electrical resistance.** The TEER has been measured for pial vessels on the surface of the brain and is 1600 ohm·cm^2^ [7], which is high compared to the TEER across the choroid plexus, 26 ohm·cm^2^ [6]. However, pial vessels are not representative of intra-parenchymal vessels in brain, as pial vessels lack an astrocyte ensheathment found in parenchymal vessels [8]. The TEER across intra-parenchymal vessels has been estimated at 8000 ohm·cm^2^ [10]. The TEER is very low, <50 ohm·cm^2^, with a human vitro BBB model using the hCMEC/D3 line [1187]. The TEER in the transwell co-culture model increases to 600–800 ohm·cm^2^ [1177,1188]. The TEER is 1700–3000 ohm·cm^2^ in cultures derived from iPSCs exposed to 5 μM retinoic acid [1183]. TEER values approximate 8000 ohm·cm^2^ when iPSC-derived endothelial cells are grown on transwells opposite co-cultures with pericytes followed by neurons/astrocytes [1189]. Although TEER values in these advanced co-culture models are approximating the TEER at intra-parenchymal vessels in brain in vivo, the in vitro models are still leaky compared to the BBB in vivo, when Pe values for sucrose are measured.

**Sucrose permeability in the in vitro BBB models.** The high and low ranges of BBB permeability coefficients (Pe, cm/s) may be defined by diazepam and sucrose. Based on the in vivo PS product for diazepam [1115], a brain capillary endothelial surface area in vivo of 120 cm^2^/g [11], the in vivo diazepam Pe = 1.8 × 10^−4^ cm/s [569]. The in vivo PS product for [^13^C]-sucrose is 0.04 uL/min/g [1190,1191], which corresponds to a sucrose Pe of 5.5 × 10^−9^ cm/s, a value 5 log orders lower than the in vivo Pe for diazepam. The sucrose Pe value is 5 × 10^−6^ cm/s in either a flow-based dynamic in vitro BBB model [1179] or a transwell co-culture model [1188], which is 1000-fold higher than the sucrose Pe value in vivo. The sucrose Pe, 5 × 10^−7^ cm/s, in a retinoic acid differentiated iPSC in vitro model that produces a TEER up to 3000 ohm·cm^2^ [1183], is still 100-fold higher than the sucrose Pe value in vivo [1190,1191].

**BBB-specific gene expression in in vitro BBB models.** BBB-specific gene expression is down-regulated when brain capillary endothelial cells are grown in cell culture [1192]. The mRNA encoding BBB-specific transporters such as GLUT1 or LAT1 was decreased at least 100-fold when transporter mRNA levels in freshly isolated brain capillaries was compared to cultured endothelium [358,1156,1175]. Gene expression in freshly isolated rat brain microvessels was up to 3 log orders of magnitude higher than expression of the same BBB-related genes in primary cultures of rat brain endothelium [1193]. Gene expression in primary cultures of human brain endothelium was down-regulated up to 6 log orders of magnitude [1194], when compared to BBB gene expression in vivo [1158]. The cause of the down-regulation of BBB specific gene expression in cell culture is not known, but may be related to the breakdown of the neuro-vascular unit in cell culture. There is a close apposition of astrocyte foot processes and the brain microvascular endothelium in vivo as these cellular structures are separated by a distance of only 20 nm [1088]. Given this close proximity of endothelial cells, astrocyte foot processes, and pericytes, which share the same basement membrane with the endothelium, it may be that current co-culture models do not replicate the proximity between these cells that exist in vivo.

**Cellular proximity with the in vitro BBB model.** Early work showed that the induction of BBB properties in cultured endothelium was observed only in mixed cultures, not co-cultures of endothelium and astrocytes [1195]. BBB properties could be induced by co-cultures of endothelium and astrocytes if the pore size of the transwell was 3.0 microns, but not if the pore size was 0.45 microns [1196]. Similarly, if the channel size is only 0.4 microns in a BBB-on-a-chip model, there is no spread of astrocyte processes into the endothelial chamber [1181]. The larger pore size of 3 microns enabled astrocyte cellular processes to extend through the pore to come in contact with the endothelium [1196]. The pore size of standard in vitro BBB co-cultures is 0.4 microns [1177,1179,1197], which prevents cell-to-cell contact between endothelium and the cells on the other side of the filter. The close proximity of neurovascular unit cells is produced in spheroid mixed cultures [1198], but transport through such cultures cannot be measured. In a more recent BBB-on-a-chip model, the size of the channels connecting the inner and outer chambers is 3 microns [1199]. However, these channels are long, 100 microns, as compared to the thickness of transwell chambers, 10 microns. Astrocyte processes do not extend over a distance of 100 microns in the in vitro BBB-on-a-chip model [1199].

In summary of cell culture models of BBB transport, considerable progress has been made in the development of in vitro BBB models since these were first introduced 40 years ago. However, these models have not been fully validated with in vivo/in vitro comparisons of solute and drug permeability via either lipid-mediated free diffusion, carrier-mediated transport, or receptor-mediated transport. Therefore, in vitro models should not be used as a primary method of determining drug transport across the BBB. In vitro models need to be validated and confirmed with in vivo measurements of BBB permeability. It may be that the real value of in vitro BBB models is not for the screening of drug transport across the BBB, but rather as a model that elucidates the mechanisms responsible for the induction of tissue-specific gene expression at the brain capillary endothelium, and the neuro-vascular unit.

### 11.8. BBB Transport Methods from Perspective of Pharmaceutical Industry

The methods reviewed above for the determination of the PS product of drug transport across the BBB in vivo, in either the blood-to-brain or brain-to-blood direction, are not widely employed within the pharmaceutical industry. Instead, industry seeks a unified parameter of drug distribution into brain, such as the CSF concentration, for biologics, or the log BB for small molecules. The ‘BB’ parameter, which is the ratio of total drug in brain divided by the total drug in blood (or plasma), has given way to the K_p,uu_ [1200], which is the ratio of *free* drug in brain divided by the *free* drug in blood (or plasma), as defined in Equation (3). Underlying the use of the K_p,uu_ is the likely supposition that the concentration of drug in brain that drives receptor occupancy is the free drug in brain, not the tissue-bound drug in brain. The problem with the interpretation of data on the K_p,uu_ parameter relates to how the ‘free drug in plasma’, and the ‘free drug in brain’, are experimentally determined. The ‘free drug’ methods advocated by industry allow for the measurement of the free drug in plasma and brain with in vitro methods, such as equilibrium dialysis of an aliquot of plasma in parallel with an aliquot of brain homogenate [1200].

**Free drug in plasma.** The measurement of free drug in plasma in vitro with equilibrium dialysis assumes the KD governing the binding of the drug to the plasma protein in vitro in a test tube is the same as the KD of binding of the drug to the plasma protein in vivo at the glycocalyx surface of the brain capillary endothelium. If this assumption is never subjected to direct empiric testing in vivo, then there is no adherence to the principle of “to measure is to know” [1105]. The KD^in vivo^ can be measured with in vivo BBB methods as described in Section 11.2. In many, although not all, instances the KD^in vivo^ >> KD^in vitro^ (Table 5). In this case, the measurement of free drug in vitro with equilibrium dialysis significantly underestimates the fraction of drug in plasma that is bioavailable for transport into brain. The lack of a reliable measure of the bioavailable drug in plasma, or L_F_, impacts on estimates of the free drug in brain in vivo, or L_M_, as the latter is directly related to the former, as shown by Equation (3). The measurement of free drug in plasma in vitro with a method such as equilibrium dialysis is a useful screen of the extent to which a given drug is plasma protein bound. However, if the bioavailable drug in brain in vivo is not measured, and in vitro free drug is extrapolated to the in vivo condition, then only confirmation bias is supporting the free drug hypothesis.

**Free drug in brain.** The measurement of free drug in brain in vitro with equilibrium dialysis of a homogenate of brain is useful in predicting the brain volume of distribution, or total drug BB ratio. However, the use of in vitro equilibrium dialysis of brain homogenate does not yield reliable estimates of the free drug in brain in vivo, because this in vitro homogenate approach measures free drug in brain in the absence of the continuous flow in vivo of bioavailable drug in plasma. The in vivo bioavailable drug in plasma, the L_F_ parameter in Figure 19, acts as a forcing function controlling the free drug in brain (L_M_), along with PS^influx^ and PS^efflux^, as described in Equation (3). The concentration of free drug in brain, which determines metabolic clearance and receptor occupancy in brain, is independent of brain tissue binding [1096]. This is a re-statement of pharmacokinetic principles, developed over 40 years ago, that tissue binding of drug affects tissue volume of distribution, e.g., the BB ratio, but has no effect on the tissue concentration of free drug [1201].

## 12. Summary

This review has covered the diverse array of brain drug delivery technologies that have emerged over the last three decades, and which are outlined in Figure 2, and these are highlighted below:

**ICV drug delivery to brain:**

- Drug injected into the CSF enters brain by diffusion, and diffusion decreases exponentially with the diffusion distance. Consequently, following ICV delivery, drug traverses a distance of only 1–2 mm from the CSF surface of the brain (Figure 5), as reviewed in Section 2.1.1.
- An intrathecal injection of drug is akin to a slow intravenous infusion of drug, as noted by Fishman and Christy in 1965 [83]. Therefore, the control group in a clinical trial of a drug administered by ICV injection, e.g., with an Ommaya reservoir, should be a cohort of patients administered the same drug by IV infusion, as suggested by Aird in 1984 [85], and reviewed in Section 2.1.4.

**Intra-cerebral implants:**

- Drug enters brain from an intra-cerebral implant by diffusion, which decreases exponentially with the distance from the implant. The maximal distance from the implant covered by the drug is 0.2–2 mm [118].
- To overcome the limitations of diffusion, viral vectors have been delivered to brain via multiple Burr holes drilled in the skull [982]. However, the virus penetration into the brain is limited to the area around the tip of the injection needle [980,981].

**Convection-enhanced diffusion:**

- Convection-enhanced diffusion (CED) attempts to overcome the limitations of diffusion in brain. A catheter inserted in the brain is connected to a pump [53]. A clinical trial of GDNF delivery to brain with bilateral CED failed in PD [130]. A primate study demonstrated the GDNF concentration in brain decreases exponentially with each mm of distance from the catheter [131], as illustrated in Figure 6A. Such an exponential decay in drug distribution in brain is indicative of diffusion, not convection.
- The maximum volume covered by CED in the cat brain was 100 mm^3^ [53], or 300 mm^3^ in the primate brain [120], which is only a fraction of the volume of the putamen in the human brain, 6000 mm^3^, on each side of the brain [127].

**Trans-nasal drug delivery to brain:**

- There are >1000 publications in PubMed on trans-nasal delivery to brain (Table 1). However, all clinical trials of drug delivery to brain via the nose have failed, as reviewed in Section 3.3.
- The olfactory region covers 50% of the nasal mucosa in the rat, but only 3% in humans [146].
- Drug delivery to olfactory CSF following nasal administration in preclinical studies is generally performed in rodents wherein large volumes are instilled in the nose, and these large volumes cause local injury to the nasal mucosa. The volume of the nasal mucosa in humans and mice is 20 mL and 0.03 mL, respectively [148]. Instillation of a volume >100 μL in the human naris causes local injury [147,148].

**Blood–brain barrier disruption (BBBD):**

- BBBD has been induced by intra-carotid artery hyperosmolar mannitol (ICAHM), by focused ultrasound with IV microbubbles (FUS-MB), and by a variety of methods such as opening tight junctions with an anti-claudin-5 antibody, or even electromagnetic radiation, as reviewed in Section 4.
- Disruption of the BBB to drugs also opens the BBB to plasma proteins, which are toxic to brain [197,198].
- BBBD with either ICAHM or FUS-MB causes a sterile inflammatory response in brain [200,201], vasculopathy [202], and chronic neuropathologic changes in the brain [203,228].

**Stem cell delivery to brain:**

- Stem cells do not cross the BBB [288], nor enter brain parenchyma [289] as reviewed in Section 5.1.
- Stem cells do invade the meninges of brain [289], where there is no BBB.
- Stem cells were permanently transfected with lentivirus (LV) and injected into mice, but the LV genome in brain was near the background of the method and log orders lower than in peripheral tissues [293].

**Exosome delivery to brain:**

- Exosomes are liposome-like membrane vesicles derived from cultured cells, as reviewed in Section 5.2.
- Similar to liposomes, exosomes do not cross the BBB in the absence of a surface ligand that triggers RMT across the BBB.
- The future translation of exosomes to human neurotherapeutics is limited by low encapsulation of drug in exosomes, drug efflux from exosomes on storage, the lack of stability of exosomes on long-term storage required for commercialization, the low yield of exosomes from cultured cells, and the unfavorable pharmacokinetic profiles of exosomes following IV administration.

**Small molecule delivery to brain via free diffusion:**

- All CNS drugs on the market have a MW < 450 Da and form <8 hydrogen bonds with solvent water. Only about 2% of all small molecules have these molecular properties of MW and hydrogen bonding, and these drugs typically treat only neuropsychiatric conditions or epilepsy, as reviewed in Section 6.1.1.
- The model of MW dependence of small molecule diffusion through biological membranes was developed by Stein decades ago [317], and is reviewed in Section 6.1.2, and in Figure 8.
- Water-soluble drugs have been conjugated to lipid-soluble carriers, including dihydropyridine, free fatty acid, or docosahexaenoic acid (DHA), but with little success as reviewed in Section 6.1.4.
- The 20th century model of CNS drug development of lipid-soluble small molecules needs to be expanded to include drugs that cross the BBB via carrier-mediated transport.

***Small molecule delivery to brain via BBB carrier-mediated transport:***

- Several carrier-mediated transporters (CMT) are expressed at the BBB for transport of nutrients, including GLUT1, LAT1, CAT1, MCT1, CTL1, and CNT2.
- The genes encoding these CMT systems are members of the Solute Carrier (SLC) superfamily, which includes >400 transporters and >60 families [338].
- There are >10 glucose transporters (GLUT) genes in the SLC superfamily. Therefore, if a given CMT system is being targeted as a conduit for brain drug delivery, it is important to first confirm the Substrate Transporter Profile (STP) of the CMT system that exists in vivo at the BBB correlates with the STP of the cloned transporter expressed in vitro.
- In addition to the CMT systems for nutrients, there are also several SLC transporters that mediate vitamin transport across the BBB, as reviewed in Section 6.2.7 and Table 3.
- The 3D structure of some CMT systems have been elucidated, as shown in Figure 9 for GLUT1 and LAT1. The dimensions of the transporter cavity are only 0.8–1.5 nm [347]. Therefore, drugs, which do not cross the BBB, should not be conjugated to an endogenous CMT substrate, as the transporter cavity will most likely reject the conjugate.
- Medicinal chemistry can be used to create a dual-purpose pharmaceutical that has affinity for both the CMT cavity as well as for the drug receptor in brain.

**Small molecule transport via active efflux transporters:**

- Active efflux transporters (AET) mediate the transport of molecules in the brain-to-blood direction and include members of both the SLC and the ATP-binding cassette (ABC) gene families. There are ~50 genes and 7 families in the ATP superfamily, and many of these AET systems are expressed at the BBB, as reviewed in Section 6.3.
- The model AET system is P-glycoprotein (ABCB1), but there are multiple other ABC transporters expressed at the BBB.
- Drug efflux via either ABC or SLC transporters can be assessed with the Brain Efflux Index (BEI) method, as reviewed in Section 11.5.2.

**Absorptive-mediated transport:**

- Cationic proteins or lectins traverse the BBB via absorptive-mediated transport (AMT), as reviewed in Section 7.
- Cationic proteins include cationized proteins, endogenous cationic proteins, e.g., protamine or histone, and cell-penetrating peptides (CPP), such as the tat or penetratin peptides. Wheat germ agglutinin (WGA) is the model lectin that undergoes AMT at the BBB.
- AMT ligands are not preferred delivery systems, as these tend to have low affinity for BBB binding sites, are largely sequestered within the brain endothelium, and have unacceptable toxicity profiles.

**Receptor-mediated transport:**

- Receptor-mediated transporters at the BBB include the endogenous receptors for insulin, transferrin, leptin, and the IGFs, as reviewed in Section 8.1.
- Localization of a putative BBB RMT system should be confirmed by brain immunohistochemistry (IHC), as exemplified by Figure 11A. Brain IHC for several receptors targeted for RMT shows these receptors are localized at brain cells, not at the capillary endothelium, including LRP1, LDLR, nAChR, and the NMDAR (Figure 11B).
- Receptor-specific MAbs act as molecular Trojan horses to ferry across the BBB a biologic drug that is genetically fused to the MAb. IgG fusion proteins for biologics drug delivery to brain have been engineered and validated in vivo for lysosomal enzymes, neurotrophins, decoy receptors, and therapeutic antibodies (Figure 12, Table 4).
- Avidin-biotin technology, and the engineering of IgG–avidin fusion proteins, allows for the BBB delivery of peptide or antisense radiopharmaceuticals for neuro-imaging as shown in Figure 14.

**Nanoparticles:**

- Nanoparticles (NP) are reviewed in Section 9, and they include polymer-based nanoparticles (polymeric NPs, dendrimers, micelles, and protein NPs, such as albumin NPs), lipid NPs (solid lipid NPs, liposomes), and non-polymeric NPs (magnetic NPs, carbon nanotubes).
- NPs do not cross the BBB without surface functionalization of the NP with a ligand that triggers RMT across the BBB.
- NPs have been functionalized with ligands that target CMT systems, but the narrow cavities of the CMT systems do not allow for transport of the 100 nm NP, as reviewed in Section 9.5.1.
- Apart from vaccines, NP have been slow to enter clinical trials, and no successful CNS clinical trials have been performed to date with NP formulations, as reviewed in Section 9.6.
- NPs have significant toxicity profiles, particularly for magnetic NPs, carbon nanotubes, and PBCA polymeric NPs, as reviewed in Section 9.7. Detailed safety pharmacology and toxicology studies of the effects of long-term NP administration are lacking. Such 6-month GLP toxicology studies are required for an IND application, but few IND applications have been submitted for CNS clinical trials with NPs.

**Gene therapy of the brain:**

- Viral gene therapy and non-viral gene therapy of the brain are reviewed in Section 10.1 and Section 10.2, respectively.
- Zolgensma^®®^ is an intravenous AAV gene therapeutic, and was FDA approved in 2019 as a single-dose treatment for spinal muscular atrophy (SMA) at an IV dose of 1.1 × 10^14^ vg/kg [994]. Zolgensma is a self-complementary (sc) form of adeno-associated virus (AAV)-9, which undergoes BBB transport following IV administration [995].
- AAV is a hepatotropic virus [1008], and Zolgensma treatment causes abnormal liver function tests in 90% of subjects [1009]. The IV injection of 10^14^ vg/kg of AAV to newborn mice induces hepatocellular cancer in 70% of mice observed long-term [1010].
- AAV treatment induces a strong immune response against both the viral capsid protein, as well as the protein product of the therapeutic gene [992,1005]. Long term T cell immunity against the NAGLU lysosomal enzyme was observed in subjects receiving an intra-cerebral injection of AAV-NAGLU [1007].
- Non-viral gene therapy of brain is possible with Trojan horse liposomes (THLs) as described in Figure 17. THLs are produced by conjugation of a receptor-specific MAb to the tips of polyethyleneglycol strands on the surface of 100–150 nm pegylated liposomes. Both reporter genes and therapeutic genes have been delivered to mice, rats, and monkeys with antibodies that target either the insulin receptor or the transferrin receptor at the BBB.

## 13. Perspective

Brain drug delivery science is important to the overall mission of CNS drug development because ~100% of biologics do not cross the BBB, and ~98% of small molecules do not cross the BBB. The absence of drug transport across the BBB is the singular reason that CNS drug development is so difficult. Yet, CNS drug developers practice their craft by adhering to two conflicting beliefs: (a) drugs for CNS disease can be developed, and (b) CNS drug development can take place in the absence of any consideration of the blood–brain barrier. These contradictory beliefs are illustrated by recent reviews of drug development for AD [1202,1203,1204,1205], PD [755,1206], stroke [1207], brain cancer [1208], Huntington’s disease [1209], ALS [1210], ataxia [1211], spinal cord injury [1212], traumatic brain injury [1213], or addiction [1214]. In none of these reviews on drug development for specific brain diseases was the BBB even mentioned, so the crucial issue of brain drug delivery was uniformly *in absentia* in the CNS drug development process. If the drug does not cross the BBB, and delivery to the target in brain is not possible, then drug development will lead to clinical trial failure.

**Figure 25:**
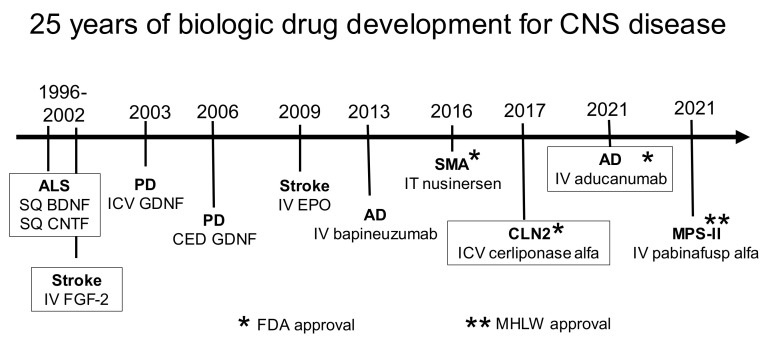
**Biologics drug development for the CNS over the last 25 years.** See Abbreviations section.

The futility of CNS drug development in the absence of BBB delivery technology over the course of the last 25 years is illustrated by a review of failed clinical trials of biologics for CNS disease. New drug approvals are increasingly biologics, and by 2019, 43% of all prescription revenues were generated by biologics [772]. The earliest biologics to enter CNS clinical trials were recombinant human BDNF or CNTF, which were developed as new treatments for neuro-degeneration, such as AD, PD, or ALS. The initial neurodegenerative condition targeted for clinical testing with neurotrophic factors was ALS. Both BDNF and CNTF were administered by SQ injection to patients with ALS in large phase 3 clinical trials [753,754]. Neither BDNF nor CNTF cross the BBB, and cannot reach the therapeutic targets within the brain following SQ administration of the neurotrophin. Both the BDNF and the CNTF clinical trials for ALS ended in failure [753,754], and are depicted in Figure 25 as the beginning of 25 years of CNS biologics drug development.

In neither the report of the failed BDNF clinical trial [753] nor the report of the failed CNTF clinical trial [754] was the issue of BBB delivery discussed. Another neurotrophin, GDNF, was developed as a new treatment for PD. Since GDNF does not cross the BBB, the neurotrophin was administered in one phase 3 trial for PD by ICV injection, and in another phase 3 trial for PD by CED. These BBB avoidance strategies do not result in adequate drug delivery to brain, as reviewed in Section 2.1.4 and Section 2.2.2, respectively, and both phase 3 trials ended in failure [107,130]. Neurotrophins were also developed as new treatments of acute stroke, and FGF-2 and EPO were administered as IV infusions within the first 5 h of the stroke. These neurotrophins do not cross the BBB, and the BBB is intact in the early hours after stroke when neuroprotection is still possible, as reviewed in Section 8.3.2. Both phase 3 clinical trials for neurotrophin treatment of acute stroke ended in failure [761,762]. Anti-Abeta amyloid antibodies (AAA) were developed as new treatments for AD, and the first AAA developed for AD, bapineuzumab, was followed by over a half dozen AAAs that entered clinical trials for AD. Bapineuzumab does not cross the BBB, as reviewed in Section 8.3.4, yet bapineuzumab was administered by IV infusion to AD patients. Since the BBB is intact in AD [786], the therapeutic antibody could not reach the amyloid targets in brain, and the bapineuzumab phase 3 trial ended in failure [787,788]. Aducanumab, another AAA for AD, also does not cross the intact BBB [786]. However, aducanumab reduces brain amyloid in AD [790]. The mechanism for aducanumab entry into brain of AD subjects appears to be BBB disruption, since there is a linear correlation between plaque reduction and ARIA-E [786], as discussed in Section 8.3.4. Aducanumab was approved in 2021 for AD amid controversy [793], and its use has been rejected by the health care community [1215]. Aducanumab is most likely a superior form of treatment for AD, should the antibody be enabled to enter the brain without BBB disruption. Aducanumab, and other AAAs, can be re-engineered with BBB Trojan horse technology to enable RMT across the BBB, as reviewed in Section 8.3.4. Nusinersen, a phosphorothioate-ASO, was approved for treatment of SMA by injection into the lumbar CSF [95]. As reviewed in Section 2.1.3, SMA is amenable to lumbar intrathecal delivery, because the drug targets, spinal cord motor neurons, lie near the surface of the spinal cord contiguous with the intra-lumbar injection. Despite the unique spatial relationship of motor neurons in the lumbar spinal cord to a lumbar CSF injection, attempts were made to replicate the nusinersen/SMA model for brain diseases of the parenchyma of brain or spinal cord. Tominersen is a phosphorothioate-ASO for Huntington’s disease (HD), and tofersen is a phosphorothioate-ASO for ALS, and both drugs entered phase 3 trials where the phosphorothioate-ASO was administered by monthly intrathecal injection in the lumbar CSF. As discussed in Section 2.1, this route of brain drug delivery is not expected to deliver drug to the parenchyma of brain. The phase 3 trials for tominersen for HD and tofersen for ALS were terminated in 2021 [1216,1217]. Recombinant TPP1 (cerliponase alfa) was approved for treatment of the brain in CLN2 disease, an inherited lysosomal storage disorder, and the enzyme was infused into one lateral ventricle bimonthly via an Ommaya reservoir [110]. The control group in the pivotal clinical trial was historical controls, perhaps from a natural reluctance to subject young patients to a chronically implanted Ommaya reservoir for the purpose of placebo infusion. However, as emphasized over 40 years ago by Aird [85], the proper control group for an ICV drug trial is IV administration of the study drug. This is because drug injected into the CSF is rapidly exported to blood as noted by Fishman and Christy over 50 years ago [83]. Therefore, the study drug may exert therapeutic effects owing to drug action in peripheral organs, which are falsely attributed to a drug effect on the CNS, as discussed in Section 2.1.4.

In all of the clinical trials outlined in Figure 25, and discussed thus far, the CNS drug development program either was silent on the issue of BBB delivery, or employed a BBB avoidance strategy. The first drug to be re-engineered with a BBB drug delivery technology and receive market approval following a phase 3 clinical trial is the IgG-iduronate 2-sulfatase fusion protein, pabinafusp alfa (IZCARGO^®®^) [720], which received MHLW approval in Japan in 2021 for treatment of the brain with MPSII (Figure 25). The IgG domain of this fusion protein is a TfRMAb, which enables RMT of the IgG-lysosomal enzyme fusion protein across the BBB in vivo, followed by receptor-mediated endocytosis into brain cells, as discussed in Section 8.3.1. This model of re-engineering biologics with BBB Trojan horse antibodies for RMT across the BBB can be replicated for all classes of biologics, including enzymes, neurotrophins, decoy receptors, and therapeutic antibodies. However, the CNS drug developer must first engineer a BBB drug delivery technology.

The re-engineering of the biologic for BBB delivery must take place in the earliest phases of preclinical drug development, and well before entry into clinical trials for brain disease. Should the CNS drug developer choose to go forward with a drug for brain that is not a lipid-soluble small molecule, and without BBB drug delivery technology, then the clinical trial failures of the past 25 years will only be replicated. Such a decision would be reminiscent of the choice of Fitzgerald’s Gatsby, “so we beat on, boats against the current, borne back ceaselessly into the past”.

## Figures and Tables

**Table 1:** Brain drug delivery citations in PubMed.

No.	Delivery Technology Keyword ^a^	Citations	No.	Delivery Technology Keyword ^a^	Citations
1	Nanoparticles (1995)	4169	11	Cationic (1987)	437
2	Ultrasound (2001)	1472	12	p-glycoprotein (1989)	382
3	Cerebral implant (1994)	1417	13	Transferrin receptor (1991)	373
4	Liposomes (1990)	1285	14	Dendrimers (2004)	364
5	Nasal (1982)	1024	15	Carrier-mediated transport (1975)	327
6	Lipid carrier (1996)	814	16	Cell-penetrating peptide (2000)	263
7	Cerebrospinal fluid (1963)	666	17	Exosomes (2011)	224
8	BBB disruption (1979)	627	18	Tat (1994)	155
9	Small molecules (1954)	598	19	Insulin receptor (1995)	134
10	Receptor-mediated transport (1986)	566	20	Convection-enhanced diffusion (1994)	124

^a^ PubMed search term is ‘brain drug delivery and keyword’, and each of the 20 keywords is listed in Table. The first year of publication of the brain drug delivery technology is given in parenthesis next to the technology keyword.

**Table 2:** Blood–brain barrier carrier-mediated transport system.

Carrier	SLC Gene	Substrate	Km (µM)	Vmax (nmol/min/g)	Ccap (pmol/mg_p_)	Molecules per S *
Hexose (GLUT1)	2A1	D-glucose	11,000 ± 1400	1420 ± 140	139 ± 46	600
Monocarboxylates (MCT1)	16A1	L-lactate	1800 ± 600	91 ± 35	2.3 ± 0.8	2300
Large neutral AAs (LAT1)	7A5	L-phenylalanine	26 ± 6	22 ± 4	0.43 ± 0.09	3000
Cationic AAs (CAT1)	7A1	L-arginine	40 ± 24	5 ± 3	1.1 ± 0.2	270

AA = amino acid; Km, Vmax and molecules/s from [361]; brain capillary transporter concentration (Ccap) from [360]; calculation of number of molecules transported per s derived from Vmax/Ccap ratio, assuming 0.14 mg capillary protein per gram brain, and that 50% of the capillary transporter is distributed to the luminal endothelial membrane [361]. * Molecules/s is the number of substrate molecules that flux through the transporter per second at maximal velocity (Vmax).

**Table 3:** Vitamin transporters at the blood–brain barrier.

Vitamin	MW	Polarity	Transporter	SLC
Thiamine (B1)	265	charged	THTR2	19A3
Riboflavin (B2)	376	hydrophilic	RFVT2	52A2
Niacin (B3)	123	carboxylate	MCT1	16A1
Pantothenic acid (B5)	219	carboxylate	SMVT	5A6
Pyridoxine (B6)	169	hydrophobic	THTR2	19A3
Biotin (B7, B8)	244	carboxylate	SMVT	5A6
Folic acid (B9, B11)	441	hydrophilic	FOLR1	receptor
Cobalamin (B12)	1355	hydrophilic	TCBLR	receptor

**Table 4:** IgG fusion proteins for blood–brain barrier delivery of biologics.

Class	Biologic	Disease	Reference
Lysosomal enzyme	IDUA	MPSI	[714,715]
	IDS	MPSII	[699,719,720,721,722]
	SGSH	MPSIIIA	[723,724]
	NAGLU	MPSIIIB	[725]
	ASA	MLD	[726]
	PPT1	CLN1	[727]
	ASM	NPDA	[727]
	HEXA	TSD	[727]
	GLB1	GM1 gangliosidosis	[727]
Neurotrophin	BDNF	Neurodegeneration	[728]
	GDNF	PD, stroke	[729,730,731,732]
	EPO	PD, AD	[733,734,735]
Decoy Receptor	TNFR2	PD, AD, stroke	[736,737,738]
Therapeutic antibody	Abeta amyloid MAb	AD	[696,697,739,740,741]
	BACE1 MAb	AD	[695,742,743]
	α-synuclein MAb	PD	[744]

**Table 5:** Drug binding to plasma proteins in vitro and in vivo within the brain capillary.

Drug	Plasma Protein	KD^in vitro^ (μM)	KD^in vivo^ (μM)	Reference
propranolol	bovine albumin	299 ± 25	220 ± 40	[1102]
	AAG	3.3 ± 0.1	19 ± 4	
bupivacaine	bovine albumin	141 ± 10	211 ± 107	[1113]
	AAG	6.5 ± 0.5	17 ± 4	
piroxicam	human albumin	10.9 ± 0.1	910 ± 105	[1114]
	AAG	29 ± 1	35 ± 3	
diazepam	bovine albumin	2	13,900	[1115]
	human albumin	6.3 ± 0.1	156 ± 35	[1116]
devazepide	human albumin	8.2 ± 0.8	266 ± 38	
imipramine	AAG	4.9 ± 0.3	90 ± 9	[1117]
isradipine	human albumin	62 ± 8	221 ± 7	[1118]
	AAG	6.9 ± 0.9	35 ± 2	
darodipine	human albumin	94 ± 5	203 ± 14	
	AAG	2.5 ± 0.5	55 ± 7	
domitroban	bovine albumin	35	36 ± 4	[1115]
L-tryptophan	bovine albumin	130 ± 30	1700 ± 100	[1119]
L-T3	bovine albumin	4.7 ± 0.1	46 ± 4	[1100]
testosterone	bovine albumin	53 ± 1	2520 ± 710	[1100]

AAG = human α_1_-acid glycoprotein; assumes 1 drug binding site on each plasma protein.

## Data Availability

Data sharing not applicable.

## Abbreviations

AA, amino acid; AAA, anti-amyloid antibody; AAAD, aromatic amino acid decarboxylase; AAG, α_1_-acid glycoprotein; AAV, adeno-associated virus; ABC, ATP binding cassette; ACE, angiotensin converting enzyme; AD, Alzheimer’s disease; ADA, anti-drug antibody; AET, active efflux transport; AIDS, acquired immune deficiency syndrome; ALS, amyotrophic lateral sclerosis; AMT, absorptive-mediated transcytosis; ARIA-E, amyloid-related imaging abnormalities of edema; ASA, arylsulfatase A; ASM, acid sphingomyelinase; ASO, antisense oligodeoxynucleotide; ASOR, asialoorosomucoid; AUC, area under the concentration curve; AVP, arginine vasopressin; AZT, azidothymidine; BACE, beta secretase; BB, brain:blood ratio; BP, brain:plasma ratio; BBB, blood–brain barrier; BBBD, BBB disruption; BCM, brain cell membrane; BCNU, 1,3-bis(2-chloroethyl)-1-nitroso urea; BCRP, breast cancer resistance protein; BDNF, brain-derived neurotrophic factor; BEI, brain efflux index; bFGF, basic fibroblast growth factor; BK, bradykinin; BSA, bispecific antibody; BSAT, BBB-specific anion transporter; BSG, basigin; BTB, blood–tumor barrier; BUI, brain uptake index; CAT, cationic amino acid transporter; CAV, caveolin; cBSA, cationized bovine serum albumin; Ccap, capillary transporter concentration; CCK, cholecystokinin; CDP, cystine-dense peptide; CDR-SB, Clinical Dementia Rating–Sum of Boxes; CED, convection-enhanced diffusion; CFA, complete Freund’s adjuvant; CHO, Chinese hamster ovary; CI, cation independent; CL, clearance; CLDN, claudin; CM, cisterna magna; CMC, critical micellar concentration; CMT, carrier-mediated transport; CNT, carbon nanotube; CNT2, concentrative nucleoside transporter 2; CPP, cell-penetrating peptide; CRM, cross reacting material; cRSA, cationized rat serum albumin; CsA, cyclosporine A; CSF, cerebrospinal fluid; CTD, carboxyl terminal domain; CTL, choline transporter-like protein; CTX, chlorotoxin; Da, Dalton; DABE, [D-Ala^2^]-b-endorphin; DDI, dideoxyinosine; DHA, docosahexaenoic acid; DHEAS, dehydroepiandrosterone sulfate; DHP, dihydropyridine; DMSO, dimethylsulfoxide; Dox, doxorubicin; DSPE, distearoylphophatidylethanolamine; DTPA, diethylenetriamine pentaacetate; E2, estradiol; E2G, estradiol glucuronide; E3S, estrone-3 sulfate; EAAT, excitatory amino acid transporter; ECD, extracellular domain; ECS, extracellular space; EDAC, 1-ethyl-3-(3-dimethylamino-propyl) carbodiimide; EEG, electroencephalogram; EGF, epidermal growth factor; EGFR, EGF receptor; EM, electron microscopy; EMF, external magnetic field; EPO, erythropoietin; EPOR, EPO receptor; ERT, enzyme replacement therapy; F, cerebral blood flow; FA, folic acid; FDA, Food and Drug Administration; FDG, fluorodeoxyglucose; FFA, free fatty acid; FOLR, folic acid receptor; FRC, reduced folate carrier; FS, flanking sequence; FUS, focused ultrasound; GABA, gamma aminobutyric acid; GAG, glycosaminoglycan; GAPDH, glyceraldehyde 3′-phosphate dehydrogenase; GBM, glioblastoma multiforme; GDNF, glial cell derived neurotrophic factor; GFAP, glial fibrillary acidic protein; GFP, green fluorescent protein; GLB, beta galactosidase; GLP, Good Laboratory Practice; GLUT, glucose transporter; GMP, Good Manufacturing Practice; GO, graphene oxide; GSH, glutathione; GST, glutathione S-transferase; GTP, glutamyl transpeptidase; HB-EGF, heparin binding EGF-like growth factor; HC, hemicholinium; HD, Huntington’s disease; HFt, ferritin heavy chain; HIR, human IR; HIV, human immunodeficiency virus; HPRT, hypoxanthine-guanine phosphoribosyl transferase; HRP, horseradish peroxidase; HSC, hematopoietic stem cells; HSV, herpes simplex virus; IC, intra-cerebral; ICAHM, intra-carotid arterial hyperosmolar mannitol; ICAP, internal carotid artery perfusion; ICV, intra-cerebroventricular; ID, injection dose; IDS, iduronate 2-sulfatase; IDUA, α-L-iduronidase; IGF, insulin-like growth factor; IGFBP, IGF binding protein; IGFR, IGF receptor; IHC, immunohistochemistry; IND, Investigational New Drug; INSR, insulin receptor; iPSC, induced pluripotent stem cells; IR, insulin receptor; ISF, interstitial fluid; IT, intrathecal; ITR, inverted terminal repeat; IV, intravenous; KD, dissociation constant; lacZ, bacterial β-galactosidase; LAT, large neutral amino acid transporter; LC-MS, liquid chromatography mass spectrometry; LDL, low-density lipoprotein; LDLR, LDL receptor; LEPR, leptin receptor; Lf, lactoferrin; LFt, ferritin light chain; LNAA, large neutral amino acid; LNP, lipid NP; LOQ, limit of quantitation; LRP, LDL related protein receptor; LV, lentivirus; M6P, mono-6-phosphate; M6PR, M6P receptor; MAb, monoclonal antibody; MB, microbubble; MCAO, middle cerebral artery occlusion; MCT, monocarboxylic acid transporter; MHLW, Ministry of Health, Labor and Welfare; MDR, multidrug resistance; MPP, 1-methyl-4-phenylpyridinium; MPS, mucopolysaccharidosis; MRI, magnetic resonance imaging; MRP, multi-drug resistance protein; MS, multiple sclerosis; MSC, mesenchymal stem cell; MTf, melanotransferrin; MTFA, 5′-methylenetetrahydrofolic acid; MUS, methylumbelliferone sulfate; MW, molecular weight; MWCNT, multi-walled CNT; NAb, neutralizing antibody; nAChR, nicotinic acetylcholine receptor; NAGLU, N-acetyl-α-glucosaminidase; NBT, nucleobase transporter; NLC, nano-structured lipid carriers; NMDAR, N-methyl D-aspartate receptor; NP, nanoparticle; NPC, Niemann Pick disease, type C; nt, nucleotide; NTD, amino terminal domain; NTR, neurotrophin receptor; OAT, organic anion transporter; OCT, organic cation transporter; OCTN, organic cation/carnitine transporter; ODC, ornithine decarboxylase; OR, olfactory receptor; OTC, ornithine transcarbamylase; PAMAM, poly(amidoamine); PBCA, poly(butyl cyanoacrylate); PBL, peripheral blood lymphocyte; PBN, N-tertbutyl-α-phenylnitrone; PCNSL, primary CNS lymphoma; PD, Parkinson’s disease; PDGF, platelet-derived growth factor; Pe, permeability coefficient; PEF, pulsed electric field; PEG, polyethyleneglycol; PEI, poly(ethyleneimine); PET, positron emission tomography; Pgp, P-glycoprotein; pI, isoelectric point; PK, pharmacokinetics; PLA, poly(lactic acid); PLGA, poly(lactic coglycolic acid); PLL, poly-L-lysine; PNA, peptide nucleic acid; PNP, polymeric NP; PPT, palmitoyl protein thioesterase; PS, permeability–surface area; PVS, peri-vascular space; QSAR, quantitative structure activity relationship; RAP, receptor associated protein; RCA, ricinus communis agglutinin; RCT, randomized controlled clinical trial; RFVT, riboflavin vitamin transporter; RMP, receptor-mediated permeabilizer; RMT, receptor-mediated transport; RPM, revolutions per minute; RTB, ricin toxin B chain; RVG, rabies virus glycoprotein; SA, streptavidin; scAAV, self-complementary AAV; ScFv, single chain Fv; SCI, spinal cord injury; scid, severe combined immunodeficient; SGSH, N-sulfoglucosamine sulfohydrolase; shRNA, short hairpin RNA; siNP, silica NP; SIR, sterile inflammatory response; siRNA, short interfering RNA; SLC, solute carrier; SLN, solid lipid NP; SMA, spinal muscular atrophy; SMVT; sodium dependent multivitamin transporter; SOD, superoxide dismutase; SPION, superparamagnetic iron oxide nanoparticles; SPLP, stabilized plasmid–lipid particles; SQ, subcutaneous; SR, scavenger receptor; ssAAV, single stranded AAV; SST, somatostatin; SSTR, SST receptor; STP, substrate transporter profile; SWCNT, single-walled CNT; TARBP, trans-activation-responsive RNA-binding protein; T3, triiodothyronine; TBI, traumatic brain injury; TC, transcobalamin; TcBLR, transcobalamin receptor; TEER, trans-endothelial electrical resistance; Tf, transferrin; TfR, Tf receptor; TH, tyrosine hydroxylase; THL, Trojan horse liposome; THTR, thiamine transporter; TK, tyrosine kinase; TM, transmembrane; TMR, transmembrane region; TNF, tumor necrosis factor; TNFI, TNF inhibitor; TNFR, TNF receptor; TPP, tripeptidyl tripeptidase; UPDRS, Unified Parkinson Disease Rating Scale; VCN, vector copy number; VD, volume of distribution; VIP, vasoactive intestinal peptide; VPA, valproic acid; Vp, brain plasma volume; VT, brain water volume; WGA, wheat germ agglutinin.
